# Safety and immunogenicity of rAd26 and rAd5 vector-based heterologous prime-boost COVID-19 vaccine against SARS-CoV-2 in healthy adolescents: an open-label, non-randomized, multicenter, phase 1/2, dose-escalation study

**DOI:** 10.3389/fimmu.2023.1228461

**Published:** 2023-08-01

**Authors:** Amir I. Tukhvatulin, Inna V. Dolzhikova, Alina S. Dzharullaeva, Daria M. Grousova, Anna V. Kovyrshina, Olga V. Zubkova, Ilya D. Zorkov, Anna A. Iliukhina, Artem Y. Shelkov, Alina S. Erokhova, Olga Popova, Tatiana A. Ozharovskaia, Denis I. Zrelkin, Fatima M. Izhaeva, Dmitry V. Shcheblyakov, Ilias B. Esmagambetov, Elisaveta A. Tokarskaya, Natalia A. Nikitenko, Nadezhda L. Lubenets, Elizaveta A. Khadorich, Vladimir A. Gushchin, Svetlana N. Borzakova, Anna V. Vlasova, Ismail M. Osmanov, Valerii V. Gorev, Boris S. Naroditsky, Denis Y. Logunov, Alexander L. Gintsburg

**Affiliations:** ^1^ Federal State Budget Institution “National Research Centre for Epidemiology and Microbiology Named After Honorary Academician N. F. Gamaleya”, Ministry of Health of the Russian Federation, Moscow, Russia; ^2^ Children’s City Clinical Hospital named after Z. A. Bashlyaeva, Moscow City Health Department, Moscow, Russia; ^3^ Morozov Children’s City Clinical Hospital, Moscow Health Department, Moscow, Russia; ^4^ Sechenov First Moscow State Medical University, Ministry of Health of the Russian Federation, Moscow, Russia

**Keywords:** Sputnik M, Sputnik V, Gam-COVID-Vac, SARS-CoV-2, COVID-19

## Abstract

To protect young individuals against SARS-CoV-2 infection, we conducted an open-label, prospective, non-randomised dose-escalation Phase 1/2 clinical trial to evaluate the immunogenicity and safety of the prime-boost “Sputnik V” vaccine administered at 1/10 and 1/5 doses to adolescents aged 12–17 years. The study began with the vaccination of the older cohort (15-to-17-year-old participants) with the lower (1/10) dose of vaccine and then expanded to the whole group (12-to-17-year-old participants). Next, 1/5 dose was used according to the same scheme. Both doses were well tolerated by all age groups. No serious or severe adverse events were detected. Most of the solicited adverse reactions were mild. No significant differences in total frequencies of adverse events were registered between low and high doses in age-pooled groups (69.6% versus 66.7%). In contrast, the 1/5 dose induced significantly higher humoral and T cell-mediated immune responses than the 1/10 dose. The 1/5 vaccine dose elicited higher antigen-binding (both S and RBD-specific) as well as virus-neutralising antibody titres at the maximum of response (day 42), also resulting in a statistically significant difference at a distanced timepoint (day 180) compared to the 1/10 vaccine dose. Higher dose resulted in increased cross-neutralization of Delta and Omicron variants.;

**Clinical Trial Registration:**

ClinicalTrials.gov, NCT04954092, LP-007632.

## Introduction

Over two years have passed since the Coronavirus disease 2019 (COVID-19) pandemic was declared, and significant efforts to scale up vaccine manufacturing have enabled a step forward in reshaping vaccine distribution. The updated WHO global vaccination strategy to achieve the target of 70% of total population coverage requires vaccination coverage to be extended from a higher to a lower priority group, represented by young people under the age of 18 years (1).

Although children and adolescents are known to have a lower incidence of severe COVID-19, underlying health comorbidities (type 2 diabetes, severe asthma, immunocompromising conditions, and others) have been shown to increase mortality approximately by 10-fold compared to healthy individuals (2). While the direct effect of vaccination on SARS-CoV-2 transmission seems to be disputable (at least for parental vaccines) (3, 4), there are numerous additional socio-economic benefits of vaccinating children and adolescents in terms of continued educational processes, maintenance of physical activity and psycho-emotional status, to name a few.

The Gam-COVID-Vac (Sputnik V) adenoviral-based COVID-19 vaccine was developed at the Gamaleya Centre in 2020. Phase 1–3 clinical trials of Gam-COVID-Vac in adult volunteers (18+ years) have demonstrated a good safety profile, high immunogenicity, and over 91% efficacy in preventing symptomatic COVID-19 as early as 3 weeks after the first vaccination (5, 6). Once the vaccine was approved for clinical use in adults in Russia in August 2020, the new Phase 1–3 clinical trial was launched in January 2021 to assess safety, tolerability, and immunogenicity in adolescents. Here we report the results of a Phase 1/2 study involving 100 volunteers of 12–17 years old, followed by a gradual selection of 1/10 and 1/5 doses for adults with a 180-day follow-up.

## Methods

### Study design and participants

Considering the lower weight of adolescents and generally higher immune responses compared to adults, we used two different doses of 1/10 (1x10^10^ vp) and 1/5 (2x10^10^ vp) of the Sputnik V vaccine.

Before the start of the clinical trial, a pre-screening stage was added to ensure the rapid enrollment of the planned quantity of participants. Volunteers (not yet participants) signed informed voluntary consent to medical examination and underwent the same medical procedures (free of cost) at pre-screening stage as it was planned at screening (double-check examination). To minimize the likelihood of volunteers becoming seropositive, the individuals who passed the pre-screening step were invited immediately to the screening (in groups of 10–20) without waiting for the pre-screening stage to be completed. De facto, the average time between the pre-screening stage and vaccination with the first dose for each individual was 7 days. The study (including pre-screening and screening stages) was carried out in two centers in Russia: Morozov Children’s City Clinical Hospital of the Moscow City Health Department and Children’s City Clinical Hospital named after Z. A. Bashlyaeva of the Moscow City Health Department.

The study was launched on June 5, 2021 (screening of the first volunteer) and involved healthy volunteers of both sexes aged from 12 to 17 years. Screening procedure included physical examination assessing relevant vital functions (e.g., blood pressure, ECG, pulse oximetry and axillar temperature) as well as collection of demographic and anthropometric data. Volunteers underwent also laboratory testing: complete blood count and serum biochemistry, testing for infections (HIV, hepatitis, and syphilis), coagulation, COVID-19 diagnostics (PCR, IgM/IgG ELISA) and urine testing for drugs, alcohol, and pregnancy (in women). Healthy volunteers with no history of COVID-19 or prior contact with patients with COVID-19 within 14 days of participation in the study, did not receive any other vaccinations within 30 days, had not undergone therapy with steroids, immunoglobulins, or any other blood-derived products within 30 days, had not consumed any immunosuppressive drugs for more than 3 months and had no allergy to immunobiological preparations including any vaccine component were considered eligible. A complete list of inclusion and exclusion criteria is given in the **Supplementary File** (Clinical Study Protocol). Parents and the 12-to-17-year-old participants themselves provided written consent before enrollment. A total of first 100 enrolled healthy 12-to-17-year-old participants were split into two equal groups, having received either 1/10 or 1/5 dose of vaccine. The study began with the vaccination of 21 15-to-17-year-old participants with the lower (1/10) dose of vaccine. After receiving a preliminary safety report, the Data and Safety Monitoring Board (DSMB) authorized the entire group to be vaccinated at a dose of 1/10 (the rest 29 12-to-17-year-old participants). After receiving a preliminary safety report from the 1/10 dose group, DSMB authorized the second group to be vaccinated with a 1/5 dose according to the same schedule. The safety and immunogenicity parameters were assessed in age-pooled groups and stratified into older (15-to-17-year-old) and younger (12-to-14-year-old) cohorts.

The trial was registered at ClinicalTrials.gov (NCT04954092), approved by the ethics committee of Ministry of Health of the Russian Federation (#279 from Jun 29, 2021) and was conducted in compliance with the recommendations of the International Conference on Harmonization and National Good Clinical Practice guidelines and Declaration of Helsinki.

### Procedures

The Gam-Covid-Vac (Sputnik V) vaccine comprises two vector components at 10^11^vp, a recombinant adenovirus type 26 (rAd26) and a recombinant adenovirus type 5 (rAd5), with both carrying the gene for SARS-CoV-2 full-length glycoprotein S (rAd26-S and rAd5-S). The 1/10 (10^10^ vp of component I and 10^10^ vp of component II) and the 1/5 (2 ×10^10^ vp of component I and 2 × 10^10^ vp of component II) doses of Sputnik V were prepared specifically for this study in vials (0.5 ml/dose) and manufactured as a liquid formulation by the N. F. Gamaleya National Research Centre for Epidemiology and Microbiology (Moscow, Russia) according to Good Manufacturing Practice. The vaccine was administered intramuscularly in a prime-boost regimen: the interval between the first dose (rAd26) and the second dose (rAd5) was 21 days.

Along with the vaccination visits (hospitalization from one day before vaccination to one day after vaccination) on days 1 and 21, the additional observation visits were planned for day 28 ( ± 2 days), day 42 ( ± 3 days), day 90 ( ± 3 days) and day 180 ( ± 14 days). Several additional observation visits were also organized remotely as telemedicine consultations. If necessary, the volunteers were to be invited to the center for an unscheduled visit. The participants were instructed to complete a paper diary to record local and systemic adverse events (AEs). The participants were encouraged to contact center staff by telephone as required (for example, if AEs were registered or for consultation). Otherwise, the information on registered AEs was recorded from diaries at the next observation visit or *via* teleconsultation. The safety and immunogenicity analysis involved all participants having received two doses. Adverse events (both solicited and unsolicited) were recorded within 42 days after the first immunization. The serious adverse events were to be recorded throughout the study, and the follow-up was to continue until 12 months after the first immunization.

In addition to screening and vaccination visits, the participants underwent physical examination (palpation, auscultation, measurement of systolic and diastolic blood pressure, heart rate, body temperature, and others) on days 14, 28, 42, 90, and 180. Electrocardiogram and coagulogram were performed at screening and on day 28. Blood tests, including a complete blood count and biochemical analysis, as well as a urine test, were done at screening, on vaccination days, and on day 28.

The antibodies to the spike (S) protein SARS-CoV-2 and its receptor-binding domain (RBD) of SARS-CoV-2 were measured before the component I (day 1) and II (day 21) injections and on days 28, 42, 90, and 180 using ELISA commercial SARS-Cov-2-IgG-ELISA-BEST (Vector-Best, Russia) and SARS-CoV-2-RBD-ELISA-Gamaleya (N. F. Gamaleya National Research Centre for Epidemiology and Microbiology, Russia) test systems, correspondingly, according to manufacturers’ instructions. The level of neutralizing antibodies was analyzed using the microneutralization test with different variants of the SARS-CoV-2 virus: Wuhan B.1.1.1 hCoV-19/Russia/Moscow_PMVL-1/2020, Delta B.1.617.2 hCoV-19/Russia/SPE-RII-32758S-PMVL-CS-SPE32758/2021, Omicron BA.5 B.1.1.529 hCoV-19/Russia/SPE-RII-25357S/2022.

The cell-mediated immune response was studied before the component I vaccination (on day 1) and on day 28 by detecting antigen-specific proliferating CD4+ and CD8+ T cells by flow cytometry and quantifying interferon-γ release of peripheral blood mononuclear cells (PBMCs) upon antigen restimulation using ELISA method. Methods are described in detail in appendix pp 1-3.

### Outcomes

The primary outcome measure was the reactogenicity and safety profile of 1/10 and 1/5 doses of Sputnik V, including the number of participants with SAE’s and AE’s monitored throughout the study, the quantity and intensity of solicited local and systemic AE’s after vaccination. Secondary outcome measures were geometric mean titres (GMT) of antigen-specific IgGs measured by ELISA as well as the percentage of seroconverted participants on day 1 (before vaccination) and days 42 and 180.

### Statistical analysis

This Phase 1/2 clinical study was not designed to test any hypothesis. Thus, the sample size (n=50 for both groups) was calculated based on the probability of detecting frequent AEs (with a probability <10%) with a pre-determined sample size. The statistical analysis was performed using GraphPad Prism 9.3 and MS Excel 2019. When analyzing categorical data, we used Pearson’s Chi-square test. The Mann-Whitney test was used to compare the means of two unpaired samples. The Mann-Whitney U-test was used as a non-parametric alternative to Student’s t-test for small samples without a normal distribution. The non-parametric Wilcoxon test was used to compare the means of the two paired samples. The hypothesis testing was two-sided, with a p-value of less than 0.05 considered significant. We calculated two-sided 95% confidence intervals for the frequency of seroconversion using the Clopper-Pearson method. The correlation analysis was done with Spearman’s test, with the correlation coefficient *r* showing interactions between two datasets and taking values either from 0 to 1 (in case of positive correlation) or from –1 to 0 (in case of negative correlation).

### Role of the funding source

The funders of the study (Moscow Healthcare Department) had no role in study design, data collection, data analysis, data interpretation, or writing of the report. All authors had full access to data in the study and had final responsibility for the decision to submit for publication.

## Results

**Figure 1:**
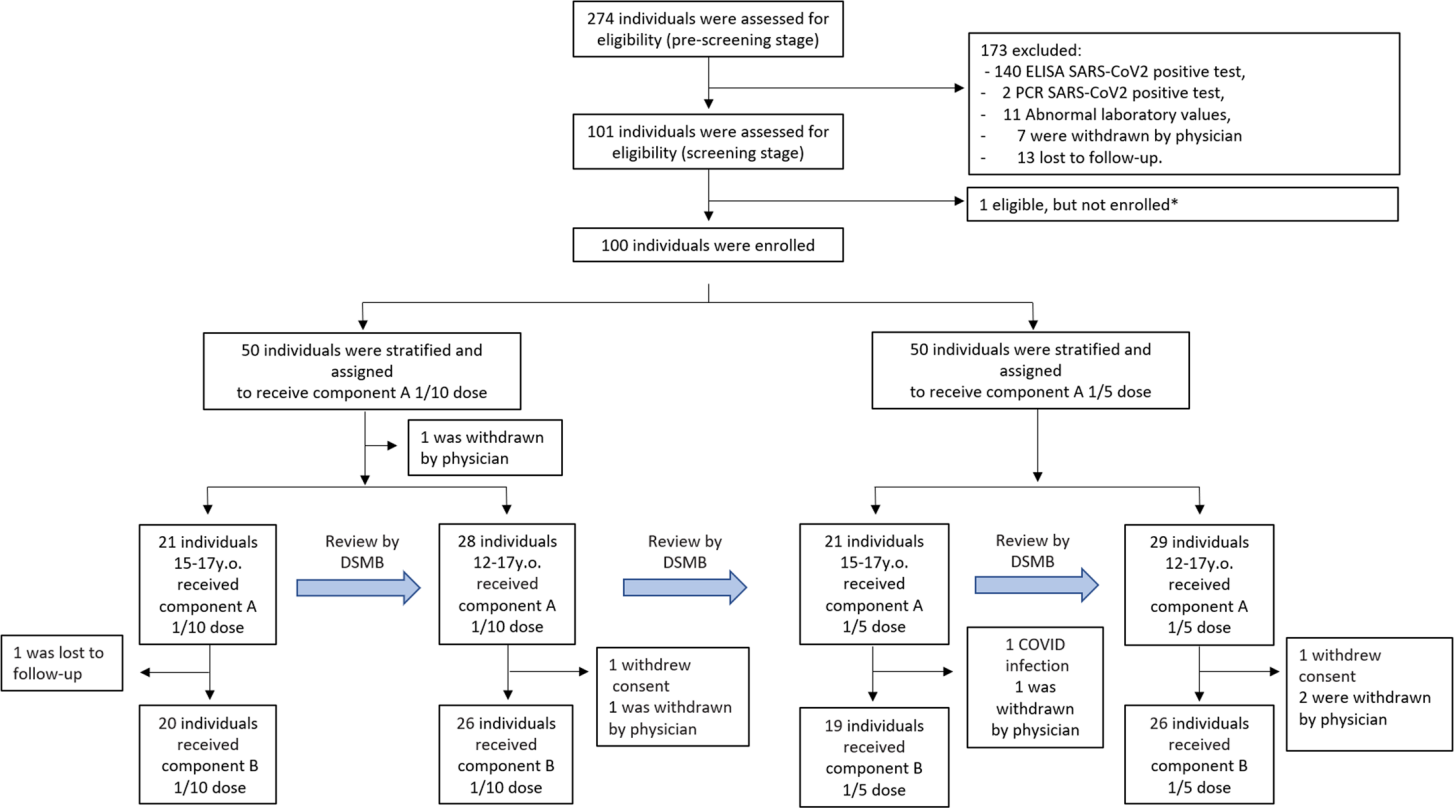
Trial profile. Due to the high percentage of volunteers with pre-existing COVID-19 immunity in the Russian population by mid-2021, an additional pre-screening stage was implemented no earlier than 3 weeks before vaccination. Screening period included 1 week before vaccination. *Due to competitive recruitment, all sites were screening participants individually; therefore, there was an excess of eligible participants who were not enrolled because the recruitment target was met. 9 participants (one before vaccination, and eight before vaccination with component B) were not included in the safety and immunogenicity analysis; reasons for their withdrawal are listed in **Supplementary Table 1**. DSMB - Data and Safety Monitoring Board.

Due to the high percentage of individuals immune to SARS-CoV-2 (approximately 16% received at least one dose of the vaccine, and 3.8% officially had COVID-19 in Russia at the moment of enrollment), the pre-screening stage was done to enrich the sample of volunteers with seronegative individuals eligible for enrollment (7). Of the 274 initially pre-screened individuals, 173 were excluded, with 140 having IgG antibodies against SARS-CoV-2, 2 being positive by PCR test, 11 having abnormal laboratory values, and 7 being withdrawn by the physician (**Figure 1**). Thirteen were lost to follow-up between the pre-screening (conducted from June 28 to July 29, 2021) and the screening stage (conducted from July 05 to July 31, 2021). Owing to competitive recruitment, 1 individual out of 101 who passed the pre-screening stage and were found to be eligible was not enrolled. On the vaccination day, 1 volunteer was withdrawn by the physician owing to the first diagnosed episode of arterial hypertension caused by excessive emotional arousal. Between the first and the second vaccination, 1 participant was lost to follow-up, 2 withdrew their consent, 4 were withdrawn by the physician due to unsolicited disorders, and 1 was diagnosed to have SARS-CoV-2 infection (**Table S1**). The final demographic characteristics of the 91 volunteers enrolled and having received two doses of vaccine are presented in **Table 1**, categorized by dose and age. No statistically significant differences were registered in demographic characteristics between participants vaccinated either with 1/5 or 1/10 vaccine dose in pooled or stratified by age groups (**Table S2**).

**Table 1:** Baseline demographic and immunological characteristics of the participants who received two-dose vaccination.

	1/10 dose			1/5 dose		
	12-14 y.o. (n=13)	15-17 y.o. (n=33)	Total (n=46)	12-14 y.o. (n=13)	15-17 y.o. (n=32)	Total (n=45)
Sex						
**Male**	7 (53.8)	18 (54.5)	**25 (54.3)**	5 (38.5)	22 (68.7)	**27 (60)**
**Female**	6 (46.2)	15 (45.5)	**21 (45.7)**	8 (61.5)	10 (31.3)	**18 (40)**
**Age,years**	13.5 (0.7)	16.2 (0.8)	**15.5 (1.5)**	12.9 (0.9)	16.0 (0.9)	**15.1 (1.6)**
Ethnicity*						
**White**	13 (100)	33 (100)	**46 (100)**	13 (100)	32 (100)	**45 (100)**
**Height,cm**	164.3 (7.6)	174.1 (9.4)	**171.3 (9.9)**	159.2 (8.5)	176.6 (9.3)	**171.5 (12.0)**
**Weight,kg**	54.3 (13.5)	66.0 (13.4)	**62.7 (14.3)**	54.1 (10.0)	68.9 (15.8)	**64.6 (15.8)**
**Body mass index, kg/m2^#^**	19.9 (3.4)	21.7 (3.3)	**21.2 (3.4)**	21.2 (2.9)	22.0 (4.0)	**21.8 (3.7)**
Immunity to SARS-CoV-2						
**Pre-existing anti-N** **SARS-CoV-2 IgG antibodies^$^,** **N (%)**	0 (0)	3 (9.1)	**3 (6.5)**	1 (7.7)	6 (18.8)	**7 (15.6)**
**Pre-existing anti-S SARS-CoV-2 IgG antibodies^$^, N (%)**	1 (7.7)	5 (15.2)	**6 (13.0)**	4 (30.7)	10 (32.3)	**14 (31.1)**
**SARS-CoV-2 infection, presence of anti-N SARS-CoV-2 IgG antibodies^$^, N (%)**	3 (23.1)	9 (27.3)	**12 (26.1)**	5 (38.5)	7 (21.9)	**12 (26.7)**

Withdrawn participants (n=9) are excluded. The data are n (%) or mean (SD), unless otherwise stated. *Ethnic group was reported by the participants. ^#^Calculation was based on the bodyweight and height measured at the time of screening. ^$^Detection of seropositive participants was done referring to the threshold for positivity set by the manufacturer (Vector-Best ELISA kits) Bold values represent summarized results for each dose

The safety analysis involved registering solicited local and systemic adverse reactions, including the changes in safety-related laboratory parameters within the first 28 days after the first dose. Adverse events are presented for each of the doses studied (1/10 and 1/5) as well as for each age stratum (12–14 years and 15–17 years old) (**Table 2**). Overall, vaccination with the adenovirus-based vaccine showed good tolerability in adolescents. The vast majority of adverse events were mild. No serious or severe (grade 3) adverse events were registered in all studied groups. In general, all local and systemic adverse events were identical to those registered in previous studies in adults after vaccination with Sputnik-V or Sputnik-Light vaccines (5, 8). Among all participants who received 1/10 and 1/5 vaccine doses, the most common local and systemic reactions were injection site pain (39.1% and 35.6%, not significant) and hyperthermia (26.1% and 26.7%, not significant). In age pooled groups, no differences were observed in frequencies of total (69.6% and 66.7%), local (45.7% and 40%), or systemic (39.1% and 40%) adverse events between the low and high-dose vaccine groups. Stratification by age showed that 12-to-14-year-old participants vaccinated at a low dose also exhibited a higher frequency of total adverse events than 15-to-17-year-olds (92.3% versus 60.6%, p<0.05). However, the high-dose vaccination resulted in less adverse events (53.8%) in 12-to-14-year-olds compared to the 15-to-17-year-olds (68.8%, not significant). When analyzing the dose-related defenses, we found that in the 15-to-17-year-old group, a high dose administration resulted in a slight increase of total (71.9% and 60.6%, not significant), local (43.8% and 33.3%, not significant), and systemic (46.9% and 39.4%, not significant) solicited adverse events comparing to a lower dose. In contrast, among younger adolescents (12–14 years old) high-dose vaccination surprisingly resulted in a lower frequency of total (53.8% and 92.3%, p<0.05), local (30.8% and 76.9%, p<0.05), and systemic (23.1% and 38.5%, not significant) solicited adverse events, compared to the high dose. Understanding that most adverse events are reported based on a subjective self-estimation of health status, with mostly adverse events being mild-level ones, allowed us to assume that inconsistency in data groups differed by age can be attributed to variations in higher health awareness of younger adolescents and their parents. No statistically significant differences were registered in frequencies of solicited adverse events between males and females stratified by vaccine dose or by age (**Table S3**).

**Table 2:** Systemic and local solicited adverse events within 28 days after vaccination with 1/10 and 1/5 dose of Sputnik V.

	1/10 dose								
	12-14 y.o. (n=13)			15-17 y.o. (n=33)			12-17 y.o. (n=46)		
	*Total*	*Grade 1*	*Grade 2*	*Total*	*Grade 1*	*Grade 2*	*Total*	*Grade 1*	*Grade 2*
**Any symptom**	**12 (92.3)**	**12 (92.3)**	**0**	**20 (60.6)**	**20 (60.6)**	**2 (6.1)**	**32 (69.6)**	**32 (69.6)**	**2 (4.3)**
With more than one AE	5 (38.5)	5 (38.5)	0	7 (21.2)	5 (15.2)	2 (6.1)	12 (26.1)	10 (21.7)	2 (4.3)
**Any injection-site symptoms**	**10 (76.9)**	**10 (76.9)**	**0**	**11 (33.3)**	**11 (33.3)**	**0**	**21 (45.7)**	**21 (45.7)**	**0**
Pain in injection site	9 (69.2)	9 (69.2)	0	9 (27.3)	9 (27.3)	0	18 (39.1)	18 (39.1)	0
Swelling	1 (7.7)	1 (7.7)	0	2 (6.1)	2 (6.1)	0	3 (6.5)	3 (6.5)	0
Redness	1 (7.7)	1 (7.7)	0	2 (6.1)	2 (6.1)	0	3 (6.5)	3 (6.5)	0
**Any systemic symptoms**	**5 (38.5)**	**5 (38.5)**	**0**	**13 (39.4)**	**12 (36.4)**	**2 (6.1)**	**18 (39.1)**	**17 (36.9)**	**2 (4.3)**
Flu-like syndrome	0	0	0	3 (9.1)	3 (9.1)	0	3 (6.5)	3 (6.5)	0
Headache	0	0	0	0	0	0	0	0	0
Muscle and joint pain	0	0	0	0	0	0	0	0	0
Hyperthermia	3 (23.1)	3 (23.1)	0	9 (27.3)	9 (27.3)	0	12 (26.1)	12 (26.1)	0
Chills	0	0	0	1 (3.0)	1 (3.0)	0	1 (2.2)	1 (2.2)	0
Decreased appetite	1 (7.7)	1 (7.7)	0	0	0	0	1 (2.2)	1 (2.2)	0
Abdominal pain	0	0	0	0	0	0	0	0	0
Abnoramal laboratory values	2 (15.4)	2 (15.4)	0	4 (12.1)	3 (9.1)	2 (6.1)	6 (13.0)	5 (10.9)	2 (4.3)
	1/5 dose								
	12-14 y.o. (n=13)			15-17 y.o. (n=32)			12-17 y.o. (n=45)		
	*Total*	*Grade 1*	*Grade 2*	*Total*	*Grade 1*	*Grade 2*	*Total*	*Grade 1*	*Grade 2*
**Any symptom**	**7 (53.8)**	**7 (53.8)**	**0**	**23 (71.9)**	**22 (68.8)**	**1 (3.1)**	**30 (66.7)**	**29 (64.4)**	**1 (2.2)**
With more than one AE	0	0	0	8 (25)	8 (25)	0	8 (17.8)	8 (17.8)	0
**Any injection-site symptoms**	**4 (30.8)**	**4 (30.8)**	**0**	**14 (43.8)**	**13 (40.6)**	**1 (3.1)**	**18 (40.0)**	**17 (37.8)**	**1 (2.2)**
Pain in injection site	4 (30.8)	4 (30.8)	0	13 (40.6)	12 (37.5)	1 (3.1)	17 (37.8)	16 (35.6)	1 (2.2)
Swelling	0	0	0	0	0	0	0	0	0
Redness	0	0	0	1 (3.1)	1 (3.1)	0	1 (2.2)	1 (2.2)	0
**Any systemic symptoms**	**3 (23.1)**	**3 (23.1)**	**0**	**15 (46.9)**	**15 (46.9)**	**0**	**18 (40.0)**	**18 (40.0)**	**0**
Flu-like syndrome	1 (7.7)	1 (7.7)	0	2 (6.3)	2 (6.3)	0	3 (6.7)	3 (6.7)	0
Headache	0	0	0	2 (6.3)	2 (6.3)	0	2 (4.4)	2 (4.4)	0
Muscle and joint pain	0	0	0	2 (6.3)	2 (6.3)	0	2 (4.4)	2 (4.4)	0
Hyperthermia	2 (15.4)	2 (15.4)	0	10 (31.3)	10 (31.3)	0	12 (26.7)	12 (26.7)	0
Chills	0	0	0	0	0	0	0	0	0
Decreased appetite	0	0	0	0	0	0	0	0	0
Abdominal pain	0	0	0	1 (3.1)	1 (3.1)	0	1 (2.2)	1 (2.2)	0
Abnoramal laboratory values	0	0	0	3 (9.4)	3 (9.4)	0	3 (6.7)	3 (6.7)	0

Participants who received each dose of vaccine were additionally divided into two age cohorts of 12–14 and 15–17y.o. This table shows the total number (%) of volunteers who developed solicited adverse events, based on the severity: mild [grade 1], moderate [grade 2], and no serious [grade 3] adverse events were reported. Some volunteers had several adverse events of different degrees of severity. Bold values represent summarized results of indicated groups of AEs.

**Figure 2:**
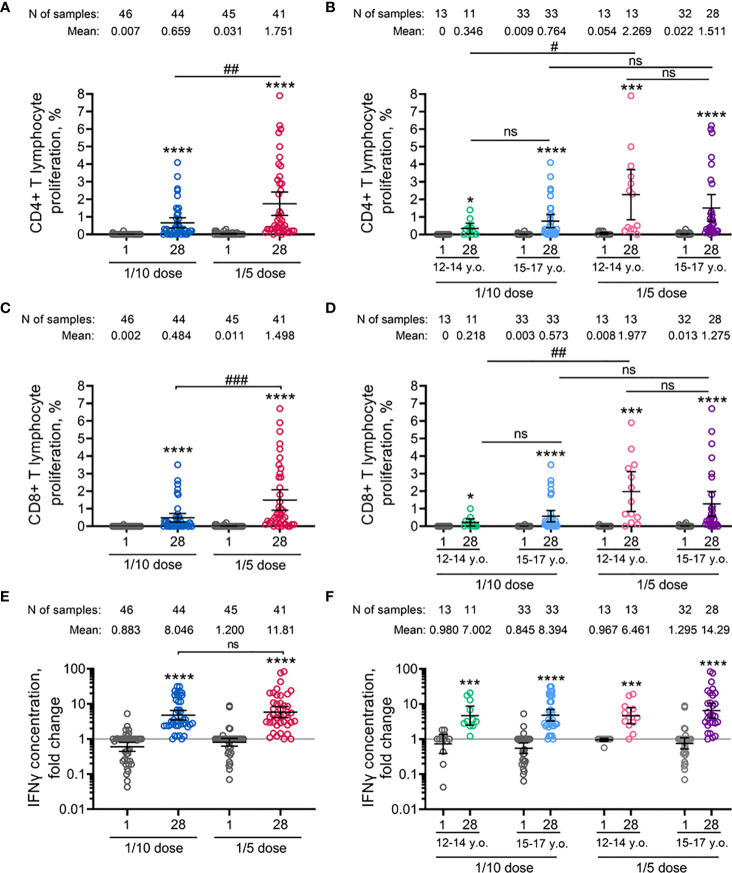
Parameters of cell-mediated immune response to SARS-CoV-2 glycoprotein in participants before immunisation (day 1) and on day 28 after vaccination with 1/10 or 1/5 dose of Sputnik V. Antigen-specific proliferation of CD4+ **(A, B)** and CD8+ **(C, D)** T cells and interferon-γ secretion in peripheral blood mononuclear cells measured by ELISA **(E, F)** in all vaccinated participants as well as separated by age strata. Dots represent individual data points. Horizontal lines represent the mean, and whiskers are 95% CIs. N represents the number of participants in each stratum. Significant differences between different timepoints within one group are indicated by asterisks: *for p<0.05, ***for p<0.005, ****for p<0.0001 (Wilcoxon test). Significant differences between different groups are indicated by hashes and lines: #for p<0.05, ##for p<0.01, ###for p<0.005 (Mann–Whitney U test). NS, not significant.

The vaccine-induced cellular immune response was evaluated by the proliferative response of CD4+ and CD8+ T cells to antigen-restimulation *in vitro*, indicated also by an increase in the concentration of interferon-γ secretion in peripheral blood mononuclear cells on day 28 after the first immunization. The age-pooled groups demonstrated statistically significant dose-dependent elevation of proliferating antigen-specific CD4+ T-helper cells (mean from 0.66% to 1.75%) and CD8+ T-killer cells (mean from 0.48% to 1.50%) (**Figures 2A, C**) in groups treated with 1/10 and 1/5 vaccine doses, respectively. Overall, in the age-pooled groups, the immunization with the 1/5 vaccine dose induced the T cell proliferative response in 100% of volunteers, compared to 90.1% for the 1/10 vaccine dose. The number of participants with cell proliferation responses to the antigen and descriptive statistics of CD4+ and CD8+ T cell proliferation for both groups vaccinated with 1/10 or 1/5 vaccine doses, stratified by age, are shown in the **Tables S4–S6**. Stratification by age revealed that in the 12-to-14-year-old cohort, the 1/5 vaccine dose resulted in statistically significant elevation of both CD4+ (mean 2.27% versus 0.35%) and CD8+ (mean 1.98% versus 0.22%) T cell proliferation compared to 1/10 vaccine dose (**Figures 2B, D**). Similar results were obtained in the 15-to-17-year-old cohort. However, the differences in proliferating CD4+ (1.51% versus 0.76%) and CD8+ (1.28% versus 0.57%) T cells between groups vaccinated with 1/5 and 1/10 doses in the 15-to-17-year-old cohort were statistically insignificant. When analyzing the age-related differences, we did not notice any statistically significant differences between 12-to-14-year-old and 15-to-17-year-old participants vaccinated with either 1/10 or 1/5 doses.

Consistent with the lymphoproliferative analysis, vaccination with the 1/5 vaccine dose resulted in a higher (but not statistically significant) increase in the concentration of interferon-γ secretion in peripheral blood mononuclear cells in age-pooled groups (11.81-fold increase) compared to 1/10 vaccine dose (8.05-fold increase) (**Figure 2E**). In detail, insignificant elevation was also observed between 12-to-14-year-old and 15-to-17-year-old cohorts after vaccination of 1/10 (7.00-fold and 8.29-fold increase) or 1/5 (6.46-fold and 14.29-fold increase) vaccine doses (**Figure 2F**). Overall, PBMCs of 97.6% (40/41) and 97.7% (43/44) of volunteers responded by interferon-γ secretion after the vaccination using 1/10 or 1/5 doses of Sputnik V, correspondingly (**Table S7**). Two IFNγ non-responders (one in 1/10 and one in 1/5 dose vaccinated groups) were positive in CD4+ and CD8+ T cell proliferation.

**Figure 3:**
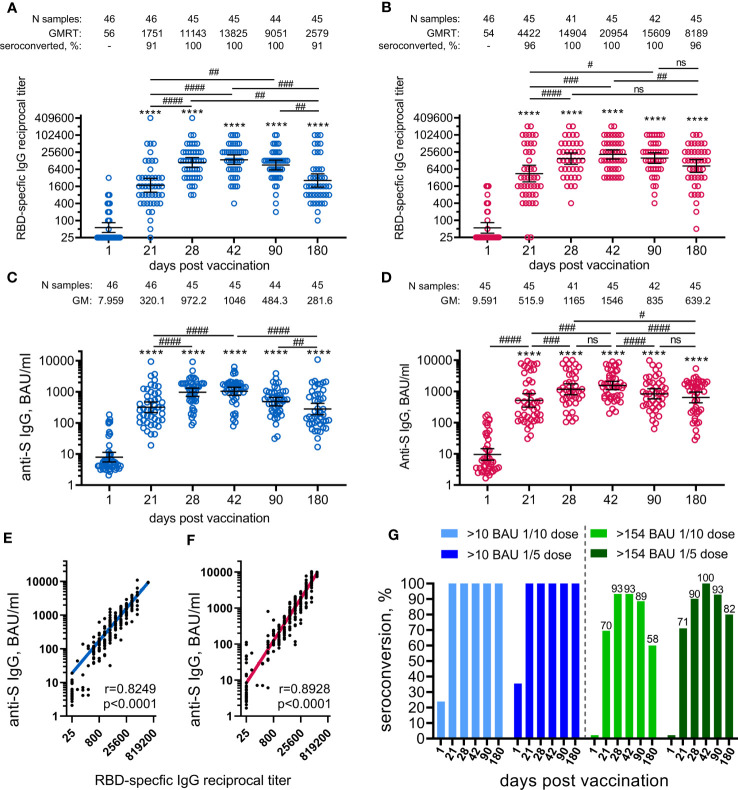
Parameters of humoral immune response in participants vaccinated with 1/10 or 1/5 dose of Sputnik V. Anti-RBD SARS-CoV-2 IgG reciprocal endpoint titres before vaccination (baseline, day 1) and on days 21, 28, 42, 90, and 180 in the participants vaccinated with 1/10 **(A)** or 1/5 dose **(B)** of Sputnik V. Anti-S IgG antibodies expressed in BAU/ml before vaccination (baseline, day 1) and on days 21, 28, 42, 90, and 180 in the participants vaccinated with 1/10 **(C)** or 1/5 dose **(D)** of “Sputnik V. Correlations with anti-S IgG measured in BAU/ml or anti-RBD IgG measured in reciprocal titre in all the participants vaccinated with 1/10 **(E)** or 1/5 dose **(F)** of “Sputnik V are shown with superimposed Log-log regression of the best-fit line. Pearson correlations were calculated, with r and p values are indicated. Dots show individual data points. Horizontal lines represent geometric mean titres, and whiskers are 95% CIs. Significant differences before (day 1) and after immunisation are indicated by asterisks (****for p<0.0001). Significant differences between different time points after immunisation are indicated by hashes and lines: #for p<0.05, ##for p<0.01, ###for p<0.001, ####for p<0.0001. NS, not significant. Dynamics of seroconversion to SARS-CoV-2 infection after the vaccination with 1/10 or 1/5 doses of Sputnik V detected by IgG ELISA, with different threshold levels in BAU/ml presented by stacked bar graphs **(G)**. Numbers above each group indicate the percentages of seroconversion.

The antigen-binding antibody response was evaluated by measuring SARS-CoV-2 RBD-specific and S-specific antibodies in volunteers’ sera collected before the immunisation (on day 1) and on days 21, 28, 42, 90, and 180. The analysis was performed immediately after receiving the samples from the day 180 timepoint. Both 1/10 and 1/5 vaccine doses induced statistically significant formation of SARS-CoV-2 RBD-specific (**Figures 3A, B**), as well as S-specific (**Figures 3C, D**) antibodies in age-pooled groups from day 21 (before boosting vaccination) and until the final day of observation (day 180). The kinetics of RBD- and S-specific IgGs had a similar shape in both 1/5 or 1/10 dose-treated groups, as confirmed by the strong correlation between the ELISA tests chosen (**Figures 3E, F**). However, the kinetics of antibody response (both RBD- and S-specific) and seroconversion rates between the two doses were different. In general, the 1/5 vaccine dose induced 1.5–3-fold higher geometric mean (GM) values over the entire observation period compared to the 1/10 dose. We also noted that vaccination with the 1/5 dose resulted in a faster elevation of RBD- and S-specific IgGs on day 21 and less steep slope after reaching a maximum on day 42 compared to the 1/10 dose. The most remarkable difference was observed on day 180, where the 1/5 vaccine dose resulted in statistically significant (p<0.01) elevation of the GM values of RBD-specific (8189 versus 2579) and S-specific (639.2 versus 281.6) IgGs compared to the group vaccinated with the 1/10 vaccine dose (**Figure S1**). A higher dose proved to be more immunogenic in both 12-to-14-year-old (11505 versus 3200, ns) and 15-to-17-year-old participants (7132 versus 2385, p<0.05) (**Figure S2**). Due to the small sample size in the 12-to-14-year-old group, these even larger differences in the antibody response (3.6-fold increase) cannot be considered statistically significant compared to the 15-to-17-year-old group (3.0-fold increase). The descriptive statistics for antigen-specific IgG titres for both groups vaccinated either with 1/10 or 1/5 vaccine doses, also stratified by age is presented in the appendix (**Tables S8–11**).

Negative results were obtained for all enrolled volunteers using the anti-S SARS-CoV-2 IgG ELISA test (Mindray Bio-Medical Electronics, China) performed at the local laboratory during the screening. However, the evaluation of the anti-N SARS-CoV-2 antibody response during the study (ELISA kit from Vector-Best, Russia) did reveal individuals with pre-existing anti-N SARS-CoV-2 antibodies before the first vaccination and those who were negative at the day 1 but were tested positive for anti-N IgG (considered as asymptomatically infected with SARS-CoV-2) during the survey. It is worth noting that the differences in RBD-specific antibody responses between different doses observed on day 180 remained statistically significant in a seronegative group (without anti-N IgG antibodies throughout the study) (**Figure S3**, **Tables S12, 13**).

A higher vaccine dose also resulted in higher seroconversion rates (96% versus 91% on day 180), defined as GMRT>4 in RBD-specific IgGs before and after the vaccination. To evaluate seroconversion rates based on S-specific IgGs expressed in BAU/ml, we applied two different cut-off values (**Figure 3G**). With the lowest cut-off of 10 BAU/ml (considered as a positive antibody response according to the ELISA kit manufacturer), both vaccine doses provided 100% seroconversion of volunteers from day 21 to 180. According to previously published data reporting the values above 154 BAU/ml to be the mean protective threshold for the WT virus, the differences in seroconversion rates between 1/5 and 1/10 vaccine doses became apparent (9). Only the 1/5 vaccine dose resulted in a 100% seroconversion rate on day 42 compared with 93% in the 1/10 dose group. The difference was also demonstrated by the slope of the curve after reaching maximum values on day 42. The most prominent and statistically significant difference between 1/5 and 1/10 vaccine dose groups was seen on day 180, with seroconversion rates of 82% (CI: 68–92%) and 58% (CI: 42–72%), respectively.

Stratification by age showed that 15-to-17-year-old participants responded with slightly lower titres (mostly statistically insignificant) of RBD-specific IgGs to vaccination with both 1/5 and 1/10 vaccine doses during the whole observation period (from 21 to 180 days) compared to 12-to-14-year-old participants (**Figure S4**). Only at day 42 at the 1/10 dose, when the RBD-specific IgG response was at its maximum, did the difference between the antibody titres between 15-to-17-year-old and 12-to-14-year-old participants reach the statistical significance (10595 versus 28735, p<0.05).

**Figure 4:**
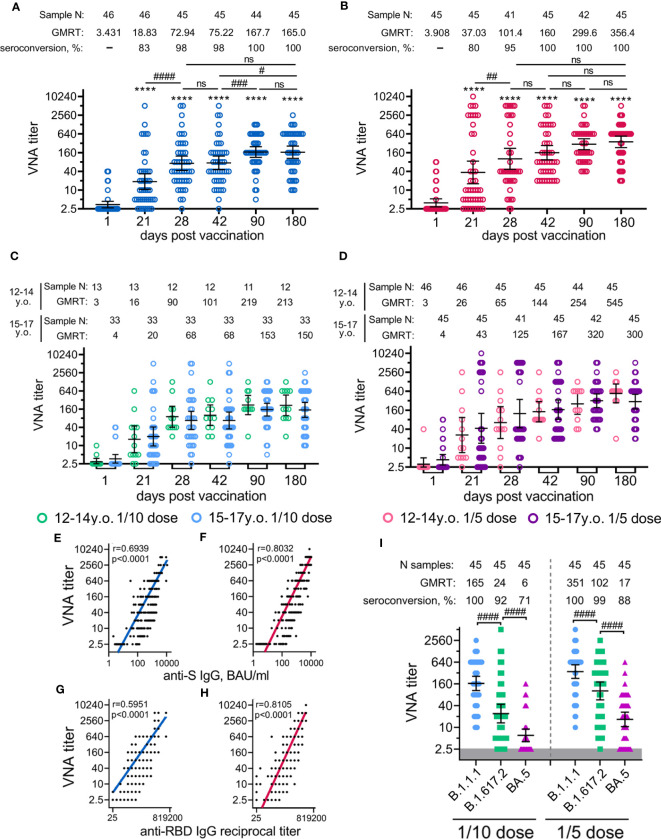
Neutralizing antibody response in participants vaccinated with 1/10 or 1/5 dose of Sputnik V. Neutralising antibodies before immunisation (day 1) and on days 21 (before vaccination with component B), 28, 42, 90, and 180, as measured by microneutralisation assay with 100 TCID50, in all participants vaccinated with 1/10 **(A)** or 1/5 **(B)** dose of Sputnik V, as well as separated by age strata **(C, D)**. Correlations with anti-S IgG measured in BAU/ml **(E, F)** or anti-RBD IgG measured in reciprocal titer **(G, F)** and titre of neutralising antibodies for both 1/10 **(E, G)** and 1/5 **(F,H)** doses were assessed with Log-log regression. Pearson correlations were calculated, and r and p values are indicated. The reciprocal neutralising antibody titres in serum against live B.1.1.1, B.1.617.2 and BA.5 SARS-CoV-2 variants on day 180 after vaccination with 1/10 or 1/5 dose of Sputnik V **(I)**. Dots represent individual data points. Horizontal lines represent geometric mean titres, and whiskers are 95% CIs. Significant differences before (day 1) and after immunisation (days 21, 28, 42, 90, 180) are indicated by asterisks (****for p<0.0001). Significant differences between different groups are indicated by hashes and lines: #for p<0.05, ##for p<0.01, ###for p<0.001, ####for p<0.0001. NS, not significant.

The analysis of neutralizing antibodies (NtAbs) to SARS-CoV-2 (B.1.1.1) revealed that immunization with both vaccine doses had statistically significant levels already on day 21 (before the boosting vaccination) up to day 180 (**Figures 4A, B**). As for antigen-specific antibodies, the kinetics of NtAbs varied in magnitude and shape depending on the vaccination dose. Overall, the 1/5 vaccine dose resulted in roughly 2-fold higher titres of NtAbs throughout the entire study than the 1/10 dose. After the vaccination with the 1/10 dose, NtAbs reached a plateau between day 90 (GMRT 167.7) and day 180 (GMRT 165.0). Notably that, NtAbs continued to increase from 299.6 on day 90 up to 356.4 on day 180 after the vaccination with the 1/5 vaccine dose. A statistically significant difference (p<0.05) between the doses was observed on the maximum of NtAbs on day 180, when NtAbs reached GMRT 356.4 and 165.0 in the 1/5 and 1/10 dose groups, respectively (**Figure S5**). The descriptive statistics of neutralizing antibody titres for both groups vaccinated either with 1/10 or 1/5 vaccine doses stratified by age are presented in the appendix (**Tables S14, 15**). Consistent with antigen-specific antibody titres, the 12-to-14-year-old participants demonstrated slightly more prominent (though not statistically significant) NtAbs titres after being administered either 1/10 or 1/5 vaccine dose when compared to the older 15-to-17-year-old group (a 1.4–1.8-fold increase) (**Figures 4C, D**). As previously shown, the analysis of the correlation between SARS-CoV-2 RBD- or S-specific antibody titres and neutralizing antibody titres showed a strong correlation between these variables (**Figures 4E, F**) (10). However, for the 1/5 dose vaccination, we noted a higher correlation coefficient (r) for both RBD- and S-specific antibodies, with neutralizing antibody titres (r=0.81, r=0.80) compared to 1/10 dose (r=0.59, r=0.69, correspondingly).

Given the significance of assessing the potential vaccine cross-protection breadth against different lineages of SARS-CoV-2, we examined the virus-neutralizing activity of sera from volunteers vaccinated with 1/10 or 1/5 vaccine dose against two variants of concern (VOC): Delta (B.1.617.2) and Omicron (BA.5) responses were compared with those from the original genetic lineage variant (B.1.1.1) (**Figure 4I**). Using serum samples obtained on day 180 from age-pooled volunteers, we detected a statistically significant decrease in the virus neutralization titres against Delta (6.9- and 3.4- fold decrease) and against Omicron (27.4- and 21.1- fold decrease) variants compared to B.1.1.1 in both groups vaccinated with the 1/10 or the 1/5 vaccine doses. A higher dose was found to cause a lower reduction in cross-reactive NtAbs than the lower dose. This observation is also confirmed by a higher percentage of seroconverted volunteers vaccinated with the 1/5 vaccine dose against Delta (98% versus 84% seropositive participants) and against Omicron (73% versus 42% seropositive participants) variants compared to the group vaccinated with the 1/10 dose (**Table S16**).

Finally, we assessed anti-Ad26 and anti-Ad5 NtAbs in volunteers before vaccination and on day 42 with different doses of the Sputnik V vaccine. Upon vaccination with both doses, GMRTs increased statistically significantly on day 42 compared to day 1, but GMRT and seroconversion rates were relatively low. When lower doses (1/10) were used, GMRTs against Ad26 and Ad5 vectors were 25 and 27, resulting in 11% and 22% of seroconversion rates, correspondingly. Upon vaccination with the 1/5 dose eliciting 100% seroconversion rate in NtAbs against SARS-CoV-2, GMRTs were 32 and 49 against Ad26 and Ad5 vectors with seroconversion rates of 13% and 47%, correspondingly (**Tables S17, 18**, **Figure S6**).

## Discussion

The ubiquitous spread of the SARS-CoV-2 virus requires not only specific prevention among adults but also the extension of vaccination coverage to young individuals under the age of 18 years.

Compared to other vaccination modalities (e.g. single shot or two dose homologous), the heterologous prime-boost vaccination schedule seemed to be the more effective option for providing robust and long-term protection from COVID-19 (11, 12). This study accessed the safety, tolerability, and immunogenicity of the adenovirus vector-based Sputnik V vaccine when used in a heterologous prime-boost regimen (with 21-day interval) in 100 adolescents (12–17 years) at low (1/10 of the adult dose) and high (1/5 of the adult dose) doses. After data collection, the participants were further subdivided into two groups: young (12-to-14-year-olds) and older (15-to-17-year-olds) cohorts to evaluate possible age-dependent differences.

The safety analysis of the vaccine revealed both the 1/10 and 1/5 doses to be safe and well tolerated for the 12-to-17-year-old participants. The adverse events reported after vaccination of adolescents with 1/10 or 1/5 doses of Sputnik V were generally similar to those reported in adult volunteers vaccinated with the full dose of Sputnik V or the single dose of Sputnik-Light vaccine (equal to the first component of the Sputnik V vaccine) (5, 8). However, the frequencies of AEs in this study differed from those in previous studies. Compared to adults, adolescents were reported to have lower frequencies of systemic AEs. For example, in volunteers aged 12–17 years, hyperthermia (37–37.9 C) was recorded in 26.7% and 26.1% after vaccination with the 1/5 and 1/10 doses of Sputnik V vaccine, respectively, while in adults vaccinated with the full dose, this symptom was detected in 95% of volunteers. This was also the case for the changes in the laboratory parameters detected in volunteers aged 12–17 years in 6.7% and 13% using 1/5 and 1/10 doses, respectively, while in adults, the laboratory parameters were altered in all volunteers. The analysis of local AEs revealed no differences in the frequency of AEs in adolescents and adults. The pain in the injection site was detected in 37.8% and 39.1% of adolescents (1/5 and 1/10 doses, respectively), and in adults, this AE was detected in 40% of volunteers. Compared to adults having a higher frequency of systemic AEs than in local AEs, adolescents tend to show an equal or even a higher frequency of local AEs (observed in the younger 12-to-14-year-old cohort using 1/5 or 1/10 vaccine doses) compared to systemic AEs. Similar tendencies were registered for BNT162b2 COVID-19 vaccine used in several studies in adolescents (13–15). It is important to note that in the context of other prime-boost COVID-19 vaccines, such as BNT162b2, ChAdOx1 nCoV-19 (AZD1222) and mRNA-1273, Sputnik V showed better reactogenicity causing no severe (grade 3) AEs in 12-to-17-year-old vaccinees (16–18). Perhaps this is due to the fact that the dosages of abovementioned vaccines for adolescents are the same as the dosages for adults, whereas Sputnik V dose for adolescents is 5-times lower than for adults.

The analysis of the humoral immune response dynamics demonstrated a dome-shaped antigen-specific IgG response, over 180 days of the study, regardless of the dose. After vaccination, a significant increase in IgG was detected, reaching its maximum by day 42 and then gradually decreasing. At the same time, the analysis of the dynamics of neutralizing antibodies over 180 days of the study found neutralizing antibodies to reach their peak values by day 90 of the study and remain at the same level on day 180. We assume that the different dynamics of the antibody response of antigen-specific IgG and neutralizing antibodies upon Sputnik V vaccination to be related to the affinity maturation of serum antibodies which was initially observed in COVID-19 convalescents (19). María M Gonzalez Lopez Ledesma et al. obtained similar data when studying the immune response duration after the vaccination of adults with Gam-COVID-Vac (Sputnik V) (20).

The emergence and spread of new SARS-CoV-2 virus variants led us to analyze further the level of neutralizing antibodies to the Delta variant (the most common variant of SARS-CoV-2 “serotype 1” and the Omicron variant sublineage BA.5 (the actual variant at the time of the manuscript writing) (21). We demonstrated that the 1/5 dose group had the highest level of neutralizing antibodies to the original virus variant (B.1.1.1) and to the Delta and Omicron BA.5 variants. Moreover, the analysis of seroconversion rates revealed that in the 1/5 dose group, 98% of volunteers had neutralizing antibodies to the Delta variant and 73% to the Omicron BA.5 variant. Obtained results are advantageous compared to those for other vector-based vaccines (ChAdOx1-S or Ad26.COV2.S) in terms of cross-reactivity against COVID-19 variants (22). Along with that the decrease of seroconversion rates to far standing variants brings the rational basis for Sputnik V vaccine adaptation against Omicron variants of virus.

This research has some limitations as it is the initial examination of the Gam-COVID-Vac (Sputnik V) vaccine in adolescents aged 12–17 years, with the primary aim of assessing safety and immunogenicity. Thus, the efficacy analysis was not the aim of the study. In addition, the sample size of the present study was insufficient to analyze rare and very rare adverse events which are to be investigated in the near future.

The advantage of this study was the duration of the follow-up of the volunteers. A six-month period allowed us to assess not only the safety of the vaccine in volunteers aged 12–17 years but also the dynamics of the humoral immune response. The study of the immune response duration demonstrated that the neutralizing antibodies reached their maximum by day 90th day of the study, with the level remaining stable until day 180, justifying that the revaccination period should not be earlier than half of the year.

In conclusion, the obtained data indicate the Gam-COVID-Vac vaccine at both doses (1/10 and 1/5 of the adult dose) in 12–17-year-old volunteers to be safe, well tolerated, and not to cause serious adverse events. Whereas immunogenicity differed in dose-dependent manner: administering a dose of 1/5 led to a more robust immune response in adolescents than a dose of 1/10. The results obtained prompted the selection of a higher (1/5 of the adult dose) vaccine dose of Sputnik V for immunisation of adolescents that has been given a separate name, Gam-Covid-Vac M (or Sputnik M vaccine). The chosen dose will be used in further multicenter, randomised, double-blind, placebo-controlled Phase 3 study in 3000 12-to-17-year-old adolescents (which is the stage II of the same ClinicalTrials.gov identifier: NCT04954092).

The results of Phase 1/2 clinical trials have served as the basis for the provisional vaccine approval for clinical use issued on November 24, 2021 (registration number LP-007632)

## Data availability statement

The original contributions presented in the study are included in the article/**Supplementary Material**. Further inquiries can be directed to the corresponding author.

## Ethics statement

The studies involving human participants were reviewed and approved by the Ethics committee of Ministry of Health of the Russian Federation (#279 from Jun 29, 2021). Written informed consent to participate in this study was provided by the participants’ legal guardian/next of kin.

## Author contributions

AT and ID wrote the original draft of the manuscript. AT, ID, NL, BN, DL and AG participated in the conceptualization of the work. AT, ID, AD, DG, AK, OZ, IZ, AI, AS, AE, OP, TO, DZ, FI, IE and VAG worked on investigation and methodology. AT, ID, DS, NL and DL analyzed and interpreted the data. AD, DS and VAG contributed to validation. ET, NN, NL, EK performed data curation. DS, BN, DL reviewed and edited the manuscript. NL, SB, AV, IO, VVG, AG and DL supervised and administered the project. AG and DL were responsible for the acquisition of funding and resources. All the authors critically reviewed the manuscript and approved the final version.

## References

[1] WHO. Strategy to Achieve Global Covid-19 Vaccination by mid-2022 (2022). Available at: https://www.who.int/news/item/22-07-2022-who-releases-global-covid-19-vaccination-strategy-update-to-reach-unprotected (Accessed 03.02.2023).

[2] Gonzalez-Garcia N Castilla-Peon MF Solorzano Santos F Jimenez-Juarez RN Martinez Bustamante ME Minero Hibert MA. Covid-19 incidence and mortality by age strata and comorbidities in Mexico City: A focus in the pediatric population. Front Public Health (2021) 9:738423. doi: 10.3389/fpubh.2021.738423 34568267 PMC8459904

[3] Franco-Paredes C. Transmissibility of SARS-CoV-2 among fully vaccinated individuals. Lancet Infect Dis (2022) 22(1):16. doi: 10.1016/S1473-3099(21)00768-4 PMC8694744 34953540

[4] Riemersma KK Haddock LA 3rd Wilson NA Minor N Eickhoff J Grogan BE. Shedding of infectious SARS-CoV-2 despite vaccination. PloS Pathog (2022) 18(9):e1010876. doi: 10.1371/journal.ppat.1010876 36178969 PMC9555632

[5] Logunov DY Dolzhikova IV Zubkova OV Tukhvatullin AI Shcheblyakov DV Dzharullaeva AS. Safety and immunogenicity of an rAd26 and rAd5 vector-based heterologous prime-boost COVID-19 vaccine in two formulations: two open, non-randomised phase 1/2 studies from Russia. Lancet (2020) 396(10255):887–97. doi: 10.1016/S0140-6736(20)31866-3 PMC7471804 32896291

[6] Logunov DY Dolzhikova IV Shcheblyakov DV Tukhvatulin AI Zubkova OV Dzharullaeva AS. Safety and efficacy of an rAd26 and rAd5 vector-based heterologous prime-boost COVID-19 vaccine: an interim analysis of a randomised controlled phase 3 trial in Russia. Lancet (2021) 397(10275):671–81. doi: 10.1016/S0140-6736(21)00234-8 PMC7852454 33545094

[7] Available at: https://www.statista.com/statistics/1239299/covid-19-vaccination-rate-in-russia.

[8] Tukhvatulin AI Dolzhikova IV Shcheblyakov DV Zubkova OV Dzharullaeva AS Kovyrshina AV. An open, non-randomised, phase 1/2 trial on the safety, tolerability, and immunogenicity of single-dose vaccine “Sputnik Light” for prevention of coronavirus infection in healthy adults. Lancet Reg Health Eur (2021) 11:100241. doi: 10.1016/j.lanepe.2021.100241 34746910 PMC8562788

[9] Goldblatt D Fiore-Gartland A Johnson M Hunt A Bengt C Zavadska D. Towards a population-based threshold of protection for COVID-19 vaccines. Vaccine (2022) 40(2):306–15. doi: 10.1016/j.vaccine.2021.12.006 PMC8673730 34933765

[10] Mendrone-Junior A Dinardo CL Ferreira SC Nishya A Salles NA de Almeida Neto C. Correlation between SARS-COV-2 antibody screening by immunoassay and neutralizing antibody testing. Transfusion (2021) 61(4):1181–90. doi: 10.1111/trf.16268 PMC8013621 33491194

[11] Garg I Sheikh AB Pal S Shekhar R. Mix-and-match COVID-19 vaccinations (Heterologous boost): A review. Infect Dis Rep (2022) 14(4):537–46. doi: 10.3390/idr14040057 PMC9326526 35893476

[12] WHO. Interim recommendations for heterologous COVID-19 vaccine schedules (2021). Available at: https://www.who.int/publications/i/item/WHO-2019-nCoV-vaccines-SAGE-recommendation-heterologous-schedules (Accessed 08.02.2023).

[13] Walter EB Talaat KR Sabharwal C Gurtman A Lockhart S Paulsen GC. Evaluation of the BNT162b2 Covid-19 vaccine in children 5 to 11 years of age. N Engl J Med (2022) 386(1):35–46. doi: 10.1056/NEJMoa2116298 34752019 PMC8609605

[14] Hause AM Shay DK Klein NP Abara WE Baggs J Cortese MM. Safety of COVID-19 vaccination in United States children ages 5 to 11 years. Pediatrics (2022) 150(2):1–26. doi: 10.1542/peds.2022-057313 PMC9706403 35581698

[15] Bloise S Marcellino A Frasacco B Gizzone P Proietti Ciolli C Martucci V. Cross-sectional survey on BNT162b2 mRNA COVID-19 vaccine serious adverse events in children 5 to 11 years of age: A monocentric experience. Vaccines (Basel) (2022) 10(8):1–12. doi: 10.3390/vaccines10081224 PMC9416594 36016112

[16] Li G Cappuccini F Marchevsky NG Aley PK Aley R Anslow R. Safety and immunogenicity of the ChAdOx1 nCoV-19 (AZD1222) vaccine in children aged 6-17 years: a preliminary report of COV006, a phase 2 single-blind, randomised, controlled trial. Lancet (2022) 399(10342):2212–25. doi: 10.1016/S0140-6736(22)00770-X PMC9183219 35691324

[17] Aldali J Meo SA Al-Khlaiwi T. Adverse effects of Pfizer (BioNTech), Oxford-AstraZeneca (ChAdOx1 CoV-19), and Moderna COVID-19 vaccines among the adult population in Saudi Arabia: A cross-sectional study. Vaccines (Basel) (2023) 11(2):1–10. doi: 10.3390/vaccines11020231 PMC9967558 36851109

[18] Ali K Berman G Zhou H Deng W Faughnan V Coronado-Voges M. Evaluation of mRNA-1273 SARS-CoV-2 vaccine in adolescents. N Engl J Med (2021) 385(24):2241–51. doi: 10.1056/NEJMoa2109522 PMC8385554 34379915

[19] Moriyama S Adachi Y Sato T Tonouchi K Sun L Fukushi S. Temporal maturation of neutralizing antibodies in COVID-19 convalescent individuals improves potency and breadth to circulating SARS-CoV-2 variants. Immunity (2021) 54(8):1841–52 e4. doi: 10.1016/j.immuni.2021.06.015 34246326 PMC8249673

[20] Gonzalez Lopez Ledesma MM Sanchez L Ojeda DS Oviedo Rouco S Rossi AH Varese A. Longitudinal Study after Sputnik V Vaccination Shows Durable SARS-CoV-2 Neutralizing Antibodies and Reduced Viral Variant Escape to Neutralization over Time. Mbio (2022) 13(1):e0344221. doi: 10.1128/mbio.03442-21 35073758 PMC8787469

[21] Simon-Loriere E Schwartz O. Towards SARS-Cov-2 serotypes? Nat Rev Microbiol (2022) 20(4):187–8. doi: 10.1038/s41579-022-00708-x PMC8855751 35181769

[22] van Gils MJ Lavell A van der Straten K Appelman B Bontjer I Poniman M. Antibody responses against SARS-CoV-2 variants induced by four different SARS-CoV-2 vaccines in health care workers in the Netherlands: A prospective cohort study. PloS Med (2022) 19(5):e1003991. doi:0.1371/journal.pmed.1003991 35580156 10.1371/journal.pmed.1003991 PMC9113667

