# Supplementary material for: Safety and immunogenicity of rAd26 and rAd5 vector-based heterologous prime-boost COVID-19 vaccine against SARS-CoV-2 in healthy adolescents: an open-label, non-randomized, multicenter, phase 1/2, dose-escalation study
Source: Front Immunol. 2023 Aug 1;14:1228461. doi: 10.3389/fimmu.2023.1228461 (PMC10432829; doi:10.3389/fimmu.2023.1228461)
Supplement: Supplementary file 1 [file DataSheet_2.docx]

Supplementary Material

Article Title

**Amir I. Tukhvatulin^1^*, Inna V. Dolzhikova^1^, Alina S. Dzharullaeva^1^, Daria M. Grousova^1^, Anna V. Kovyrshina^1^, Olga V. Zubkova^1^, Ilya D. Zorkov^1^, Anna A. Iliukhina^1^, Artem Y. Shelkov^1^, Alina S. Erokhova^1^, Olga Popova^1^, Tatiana A Ozharovskaia^1^, Denis I Zrelkin^1^, Fatima M Izhaeva^1^, Dmitry V. Shcheblyakov^1^, Ilias B. Esmagambetov^1^, Elisaveta A. Tokarskaya^1^, Natalia A Nikitenko^1^, Nadezhda L Lubenec^1^, Elizaveta A Khadorich^1^, Vladimir A Gushchin^1^, Svetlana N. Borzakova^3^, Anna V. Vlasova^4^, Ismail M. Osmanov^3^, Valerii V. Gorev^4^, Boris S. Naroditsky^1^, Denis Y. Logunov^1,2^, Alexander L. Gintsburg^1,2^ and the Vaccine Trial Group†**

*** Correspondence:** Amir I. Tukhvatulin, amir_tukhvatulin@gamaleya.org

# Supplementary Data

**1.1 Detection of antigen-specific IgG antibodies**

Titer of antigen-specific IgG antibodies was determined before vaccination (day 1) and on days 21,28, 42, 90 and 180 after vaccine administration. For quantitative detection of RBD-specific (measured in endpoint titers) or spike-specific (measured in BAU/ml) IgG antibodies we used anti-SARS-CoV-2 IgG ELISA test-system developed in Gamaleya NRCEM (registration for clinical use in Russian Federation РЗН 2020/10393 2020-05-18) or SARS-CoV-2-IgG ELISA kit from Vector-Best (registration for clinical use in Russia РЗН 2022/17065), correspondingly.

For evaluation of endpoint titers serum samples were previously 2-fold diluted (starting from 1:50) in dilution buffer and added in RBD-pre-coated (100 ng per well) and pre-blocked plates. Plates were incubated for 1 h at 37°C. After washing the plates the peroxidase-conjugated anti-human IgG detection antibodies diluted in dilution buffer were added and plates were incubated for another 1 h at 37°C. After extensive plate washing with washing buffer 100µl of TMB substrate were added in each well. 15 min later the reaction was stopped with addition of stop solution (100µl/well). The IgG titer was determined as the maximum dilution of serum, in which the OD450 value of the serum of the immunized participant exceeds the value of the control serum (serum of the participant before immunization) by more than 2 times. Seroconversion was defined by ≥4-fold increase in antigen-specific IgG titer between pre and post vaccination time points.

Assessment of spike-specific IgG antibodies was done according manufactuter’s instruction using same serum samples at 1:100 starting dilution. The concentration of IgG to S protein of SARS-CoV-2 in the samples was calculated using the calibration curve using standards in BAU. If OD of the sample was higher than OD of standards serum samples were additionally diluted by 10, 50 or 100 times prior redoing analysis. The resulting value for each sample was calculated by multiplying result in BAU of diluted sample and dilution factor. Samples with no signal at starting dilution points were attributed to twofold lower values. The OD signals were determined in both assays with a spectrophotometer Multiskan FC (Thermo Fisher Scientific Inc, USA).

- 1. **Determination of neutralizing antibodies (NtAb)**

Neutralizing antibody titers against the SARS-CoV-2 were measured before vaccination (day 1) and on days 21,28, 42, 90 and 180 after vaccine administration. NtAb titer was determined by microneutralization test using SARS-CoV-2 B.1.1.1 (hCoV-19/Russia/Moscow_PMVL-1/2020), B.1.617.2 (Delta) and Omicron BA.5 (S hCoV-19/Russia/SPE-RII-25357S/2022) in a 96-well plate. Serum samples were inactivated by incubation at 56°С for 30 min and serial two-fold dilutions in Dulbecco's modified eagle medium containing 2% heat-inactivated fetal bovine serum at a range 1:5 – 1: 5120 were made. In the analysis, blood serum samples with the characterized neutralizing activity are always taken as a control: positive control - with NtAb 80, negative control with NtAb <5 (taken as 2.5). Then 100TCID50 (median tissue culture infectious dose) of SARS-CoV-2 in volume of 50 µl was added to each sample. The samples were incubated at 37°C for 1 h, added to Vero E6 cells and incubated in a 5% CO2 incubator at 37°C for 96 h. The cytopathic effect (CPE) of the virus on the cell monolayer was assessed visually, if even a slight damage to the monolayer (1-2 «plaques») was observed in the well, this well was considered as a well with a manifestation of CPE. Neutralization titer was defined as the highest serum dilution without any CPE in two of three replicable wells. The volunteer was considered as “responder” to vaccination if the NtAb titer was ≥2-fold higher than before vaccination (neutralizing antibody seroconversion). Samples with no signal at starting dilution (1:5) were taken as 1:2.5.

- 1. **Determination of neutralizing antibodies (NtAb) against Ad5 and Ad26**

Neutralizing antibody titers against adenovirus serotype 5 and 26 were measured before vaccination (day 1) and on day 42. NtAb titer was determined by microneutralization test using recombinant Ad5-EGFP and Ad26-EGFP in a 96-well plate. Serum samples were inactivated by incubation at 56°С for 30 min and serial two-fold dilutions in Dulbecco's modified eagle medium containing 5% heat-inactivated fetal bovine serum at a range 1:25 – 1:3200 were made. If the sample neutralized Ad5-EGFP or Ad26-EGFP at 1:3200, then the analysis was repeated at a range 1:400 – 1:25600. Then 500TCID50 (median tissue culture infectious dose) of Ad5-EGFP or Ad26-EGFP in volume of 50 µl was added to each sample. The samples were incubated at 37°C for 1 h before the addition of 100 μl (4-5×10^4^ cells per well) of HEK293 cells. Then the 96-well plates were incubated in a 5% CO2 incubator at 37°C for 3 days. The number of EGFP fluorescent cell focuses was counted visually. Neutralization titers were defined as the maximum serum dilution where 50% reduction of EGFP fluorescent cell focuses compared with the positive control was determined. Samples with no signal at starting dilution (1:25) were taken as 1:12.5.

- 1. **Proliferative responses of CD4+ and CD8+ T-cells**

Whole-blood samples were collected before immunization and on day 28 after vaccine administration. Peripheral blood mononuclear cells (PBMCs) were isolated by Ficoll (1·077 g/mL; PanEco, Russia) density gradient centrifugation (800g for 30 min). Cells were washed with sterile PBS twice and stained with Carboxyfluorescein succinimidyl ester (CFSE) (Invitrogen, USA) according to the procedure described previously (1) The cells were seeded in 96-well plates (2×10^5^ cells/well), re-stimulated with the recombinant 5µg/ml SARS-CoV-2 S (S1+S2 mixture) protein (Sino biological, China). Unstimulated cells and phytohemagglutinin (PHA) stimulated cells were used as a negative and positive controls, respectively. After 96 hours cells were harvested and centrifuged at 500g for 10min. Cells were washed with sterile PBS and stained with 1 µg/mL DAPI (Sigma-Aldrich, USA) to exclude dead cells and anti-CD3 (clone SK7), anti-CD8 (clone SK1), and anti-CD4 (clone SK3) antibodies for 20 min at 4 °C in Staining Buffer (all, BD Biosciences, USA). Proliferating CD4 or CD8 T-lymphocytes were expressed as percent of cells in the final culture that divided at least once (referring to ‘Fraction diluted’ statistic) (2). Analysis was performed on FACSAriaIII flow cytometer using FACSDiva and FlowJo Software (all from BD Biosciences, USA). Proliferating CD4+ or CD8+ T lymphocytes were identified by forward and side light scatter, negative DAPI staining, expression of CD3, CD4, CD8, and low fluorescence from CFSE. Proliferation in unstimulated cells were subtracted from that of stimulated cells, and negative differences were set to zero. Results are reported as percent proliferating cells upon antigen restimulation (unspecific proliferation in unstimulated cells was subtracted for each sample). The volunteer was considered as “responder” if proliferation of antigen-restimulated T-cells after vaccination on day 28 exceeded the proliferation before vaccination, when calculating the percentage of responding volunteers.

**1.5** **Evaluation of IFNγ production**

Whole-blood samples were collected before immunization and on day 28 after vaccine administration. PBMCs were isolated as previously described and seeded in 96-well plates at 2×10^5^ cells/well density. 96 hours later cell-free media collected from unstimulated, stimulated with SARS-CoV-2 S (S1+S2 mixture) protein or PHA (1µg/ml) were used for evaluation of IFNγ concentration using gamma-Interferon-EIA-BEST ELISA kit (Vector-Best, Russia) according the manufacturer’s instructions. Values less than lower detection limit (2pg/ml) were set to 2pg/ml. Results are reported as fold increase in IFNγ concentration upon exposure to antigen (over unstimulated control) before vaccination (day 1) and on day 28. The volunteer was considered as “responder” if the fold increase in the concentration of IFNγ by antigen-restimulated PBMC on day 28 after vaccination exceeded the fold increase in the concentration of IFNγ before vaccination, when calculating the percentage of responding volunteers.

# Supplementary Figures and Tables

**Table S1. A detailed description of the condition of withdrawn volunteers**

| # | Randomization number | The reason for excluding | Vaccine dose | Age group |
| --- | --- | --- | --- | --- |
| 1 | 02-021 | First episode of arterial hypertension caused by excessive emotional arousal (withdrawn by physician) | Before prime vaccination | 15-17 y.o. |
| 2 | 01-009 | Lost to follow-up | 1/10 of therapeutic dose | 15-17 y.o. |
| 3 | 01-050 | Acute enterovirus infection (withdrawn by physician) | 1/5 of therapeutic dose | 12-14 y.o. |
| 4 | 01-046 | Acute enterovirus infection (withdrawn by physician) | 1/5 of therapeutic dose | 12-14 y.o. |
| 5 | 01-015 | Furunculosis (withdrawn by physician) | 1/10 of therapeutic dose | 15-17 y.o. |
| 6 | 02-036 | Rotavirus infection (withdrawn by physician) | 1/10 of therapeutic dose | 15-17 y.o. |
| 7 | 02-028 | Covid-19 infection | 1/5 of therapeutic dose | 15-17 y.o. |
| 8 | 02-050 | withdrew consent | 1/5 of therapeutic dose | 12-14 y.o. |
| 9 | 02-016 | withdrew consent | 1/10 of therapeutic dose | 15-17 y.o. |

**Table S2. Statistic data of demographic characteristics** **of participants (height, weight, body mass index) vaccinated with 1/10 or 1/5 dose of vaccine doses with or without stratification by age. P values were calculated using Student t-test.**

|  | **12-14 y.o.** | | | **15-17y.o.** | | | **12-17 y.o.** | | |
| --- | --- | --- | --- | --- | --- | --- | --- | --- | --- |
|  | **Height, cm** | **Weight, kg** | **Body mass index, kg/m^2^** | **Height, cm** | **Weight, kg** | **Body mass index, kg/m^2^** | **Height, cm** | **Weight, kg** | **Body mass index, kg/m^2^** |
| **1/10 dose** | 164.3 | 54.3 | 19.9 | 174.1 | 66.0 | 21.7 | 171.3 | 62.7 | 21.2 |
| **1/5 dose** | 159.2 | 54.1 | 21.2 | 176.6 | 68.9 | 22.0 | 171.5 | 64.6 | 21.8 |
| **p value**  **(t-test)** | 0.1158 | 0.9609 | 0.2849 | 0.2962 | 0.4219 | 0.7277 | 0.936 | 0.5389 | 0.4161 |

**Table S3. Number (N) and percentage (%) of male and female volunteers with any solicited adverse event (AE) in cohorts stratified ether by dose or by age. P values between males and females across different doses and age strata were calculated using Pearson’s Chi-square test.**

|  | **Male** | | | **Female** | | | | **p value** |
| --- | --- | --- | --- | --- | --- | --- | --- | --- |
|  | **N with any AE** | **N total** | **%** | **Any AE** | **Total** | | **%** |  |
| **12-17 y.o.** | | | | | | | | |
| 1/10 dose | 18 | 25 | 72.00 | 14 | 21 | | 66.67 | ns |
| 1/5 dose | 17 | 27 | 62.96 | 13 | 18 | | 72.22 | ns |
| **12-14 y.o.** | | | | | | | | |
| 1/10 dose | 6 | 7 | 85.71 | 6 | 6 | 100.00 | | ns |
| 1/5 dose | 3 | 5 | 60.00 | 4 | 8 | 50.00 | | ns |
| **15-17 y.o.** | | | | | | | | |
| 1/10 dose | 12 | 18 | 66.67 | 8 | 15 | 53.33 | | ns |
| 1/5 dose | 14 | 22 | 63.64 | 9 | 10 | 90.00 | | ns |
| **1/10 dose** | | | | | | | | |
| 12-14 y.o. | 6 | 7 | 85.71 | 6 | 6 | 100.00 | | ns |
| 15-17 y.o. | 12 | 18 | 66.67 | 8 | 15 | 53.33 | | ns |
| **1/5 dose** | | | | | | | | |
| 12-14 y.o. | 3 | 5 | 60.00 | 4 | 8 | 50.00 | | ns |
| 15-17 y.o. | 14 | 22 | 63.64 | 9 | 10 | 90.00 | | ns |

**Table S4. Statistic data (median, 25% and 75% percentile, 95%CI, Mean, SD, SEM) of antigen-specific CD4+ cell proliferation before (on day 1) and on day 28 after vaccination as measured by flow cytometry in all participants vaccinated with 1/10 or 1/5 dose of vaccine as well as stratified by age.**

|  | 1/10 dose | | | | | | 1/5 dose | | | | | |
| --- | --- | --- | --- | --- | --- | --- | --- | --- | --- | --- | --- | --- |
|  | 12-14 y.o. | | 15-17 y.o. | | 12-17 y.o. | | 12-14 y.o. | | 15-17 y.o. | | 12-17 y.o. | |
|  | 1 day | 28 day | 1 day | 28 day | 1 day | 28 day | 1 day | 28 day | 1 day | 28 day | 1 day | 28 day |
| Number of samples | 13 | 11 | 33 | 33 | 46 | 44 | 13 | 13 | 32 | 28 | 45 | 41 |
| 25% Percentile | 0.00 | 0.00 | 0.00 | 0.10 | 0.00 | 0.10 | 0.00 | 0.30 | 0.00 | 0.20 | 0.00 | 0.25 |
| Median | 0.00 | 0.30 | 0.00 | 0.20 | 0.00 | 0.25 | 0.00 | 2.30 | 0.00 | 0.55 | 0.00 | 0.60 |
| 75% Percentile | 0.00 | 0.40 | 0.00 | 1.15 | 0.00 | 0.90 | 0.15 | 3.60 | 0.00 | 2.50 | 0.00 | 3.00 |
| Lower 95% confidence limit | 0.00 | 0.00 | 0.00 | 0.10 | 0.00 | 0.10 | 0.00 | 0.30 | 0.00 | 0.30 | 0.00 | 0.30 |
| Upper 95% confidence limit | 0.00 | 0.90 | 0.00 | 0.70 | 0.00 | 0.40 | 0.20 | 3.90 | 0.00 | 1.20 | 0.00 | 2.30 |
| Mean | 0.00 | 0.35 | 0.01 | 0.76 | 0.01 | 0.66 | 0.05 | 2.27 | 0.02 | 1.51 | 0.03 | 1.75 |
| Std. Deviation | 0.00 | 0.44 | 0.04 | 1.06 | 0.03 | 0.96 | 0.09 | 2.37 | 0.06 | 1.98 | 0.07 | 2.11 |
| Std. Error of Mean | 0.00 | 0.13 | 0.01 | 0.18 | 0.00 | 0.14 | 0.02 | 0.66 | 0.01 | 0.37 | 0.01 | 0.33 |

**Table S5. Statistic data (median, 25% and 75% percentile, 95%CI, Mean, SD, SEM) of antigen-specific CD8+ cell proliferation before (on day 1) and on day 28 after vaccination as measured by flow cytometry in all participants vaccinated with 1/10 or 1/5 dose of vaccine as well as stratified by age.**

|  | 1/10 dose | | | | | | 1/5 dose | | | | | |
| --- | --- | --- | --- | --- | --- | --- | --- | --- | --- | --- | --- | --- |
|  | 12-14 y.o. | | 15-17 y.o. | | 12-17 y.o. | | 12-14 y.o. | | 15-17 y.o. | | 12-17 y.o. | |
|  | 1 day | 28 day | 1 day | 28 day | 1 day | 28 day | 1 day | 28 day | 1 day | 28 day | 1 day | 28 day |
| Number of samples | 13 | 11 | 33 | 33 | 46 | 44 | 13 | 13 | 32 | 28 | 45 | 41 |
| 25% Percentile | 0.00 | 0.00 | 0.00 | 0.10 | 0.00 | 0.00 | 0.00 | 0.35 | 0.00 | 0.10 | 0.00 | 0.10 |
| Median | 0.00 | 0.10 | 0.00 | 0.10 | 0.00 | 0.10 | 0.00 | 1.40 | 0.00 | 0.45 | 0.00 | 0.60 |
| 75% Percentile | 0.00 | 0.30 | 0.00 | 0.50 | 0.00 | 0.48 | 0.00 | 3.40 | 0.00 | 1.65 | 0.00 | 2.80 |
| Lower 95% confidence limit | 0.00 | 0.01 | 0.00 | 0.24 | 0.00 | 0.10 | 0.00 | 0.08 | 0.00 | 0.56 | 0.00 | 0.30 |
| Upper 95% confidence limit | 0.00 | 0.43 | 0.00 | 0.90 | 0.00 | 0.30 | 0.02 | 3.11 | 0.03 | 1.99 | 0.01 | 1.49 |
| Mean | 0.00 | 0.22 | 0.00 | 0.57 | 0.00 | 0.48 | 0.01 | 1.98 | 0.01 | 1.28 | 0.01 | 1.50 |
| Std. Deviation | 0.00 | 0.31 | 0.02 | 0.92 | 0.01 | 0.82 | 0.03 | 1.89 | 0.04 | 1.84 | 0.04 | 1.86 |
| Std. Error of Mean | 0.00 | 0.09 | 0.00 | 0.16 | 0.00 | 0.12 | 0.01 | 0.52 | 0.01 | 0.35 | 0.01 | 0.29 |

**Table S6. Number and (%) of participants with detected lymphoproliferative Т cell response before vaccination (day 1) and on day 28 after vaccination as measured by flow cytometry, in all participants vaccinated with 1/10 or 1/5 dose of vaccine as well as stratified by age.** N represents total number of subjects in each stratum.

|  | 1/10 dose | | | | | | 1/5 dose | | | | | |
| --- | --- | --- | --- | --- | --- | --- | --- | --- | --- | --- | --- | --- |
|  | 12-14 y.o. | | 15-17 y.o. | | 12-17 y.o. | | 12-14 y.o. | | 15-17 y.o. | | 12-17 y.o. | |
|  | 1 day (N=13) | 28 day  (N=11) | 1 day  (N=33) | 28 day  (N=33) | 1 day  (N=46) | 28 day  (N=44) | 1 day  (N=13) | 28 day  (N=13) | 1 day  (N=32) | 28 day  (N=28) | 1 day  (N=45) | 28 day  (N=41) |
| Proliferation of CD4+, N (%) | 0 | 7 (63.6) | 2 (6.1) | 27 (81.8) | 2 (4.3) | 34 (77.3) | 4 (30.8) | 12 (92.3) | 5 (15.6) | 27 (96.4) | 9 (20.0) | 39 (95.1) |
| Proliferation of CD8+, N (%) | 0 | 6 (54.5) | 1 (3.0) | 26 (78.8) | 1 (2.2) | 32 (72.7) | 1 (7.7) | 12 (92.3) | 3 (9.4) | 24 (85.7) | 4 (8.9) | 36 (87.8) |
| Proliferation of CD4+ and CD8+, N (%) | 0 | 4 (36.4) | 1 (3.0) | 22 (66.7) | 1 (2.2) | 26 (59.1) | 1 (7.7) | 11 (84.6) | 1 (3.1) | 23 (82.1) | 2 (4.4) | 34 (82.9) |
| Proliferation of CD4+ or CD8+, N (%) | 0 | 9 (81.8) | 2 (6.1) | 31 (93.9) | 2 (4.3) | 40 (90.1) | 4 (30.8) | 13 (100) | 7 (21.9) | 28 (100) | 11 (24.4) | 41 (100) |

**Table S7. Statistic data (median, 25% and 75% percentile, 95%CI, Mean, SD, SEM) of antigen-specific PBMC cell IFNɣ production (expressed in fold increase over unstimulated cells) before immunization (day 1) and on day 28, as measured by ELISA, in all participants vaccinated with 1/10 or 1/5 dose of vaccine as well as stratified by age.**

|  | 1/10 dose | | | | | | 1/5 dose | | | | | |
| --- | --- | --- | --- | --- | --- | --- | --- | --- | --- | --- | --- | --- |
|  | 12-14 y.o. | | 15-17 y.o. | | 12-17 y.o. | | 12-14 y.o. | | 15-17 y.o. | | 12-17 y.o. | |
|  | 1 day | 28 day | 1 day | 28 day | 1 day | 28 day | 1 day | 28 day | 1 day | 28 day | 1 day | 28 day |
| Number of samples | 13 | 11 | 33 | 33 | 46 | 44 | 13 | 13 | 32 | 28 | 45 | 41 |
| 25% Percentile | 0.65 | 2.41 | 0.24 | 2.42 | 0.25 | 2.42 | 1.00 | 3.10 | 0.41 | 2.89 | 0.60 | 2.97 |
| Median | 1.00 | 3.23 | 0.94 | 3.23 | 1.00 | 3.23 | 1.00 | 5.57 | 1.00 | 4.82 | 1.00 | 4.85 |
| 75% Percentile | 1.27 | 13.09 | 1 | 14.55 | 1.00 | 12.71 | 1.00 | 9.9 | 1.00 | 19.80 | 1.00 | 14.26 |
| Lower 95% confidence limit | 0.44 | 2.37 | 0.37 | 2.58 | 0.45 | 2.70 | 1.00 | 2.98 | 0.55 | 2.99 | 1.00 | 3.08 |
| Upper 95% confidence limit | 1.34 | 17.52 | 1.00 | 6.78 | 1.00 | 6.15 | 1.00 | 11.25 | 0.55 | 2.99 | 1.00 | 10.15 |
| Mean | 0.98 | 7.00 | 0.85 | 8.39 | 0.88 | 8.05 | 0.97 | 7.25 | 1.29 | 14.29 | 1.20 | 12.06 |
| Std. Deviation | 0.53 | 6.89 | 0.93 | 9.15 | 0.83 | 8.59 | 0.12 | 5.56 | 1.95 | 21.02 | 1.64 | 17.85 |
| Std. Error of Mean | 0.15 | 2.08 | 0.16 | 1.60 | 0.12 | 1.29 | 0.03 | 1.54 | 0.34 | 3.97 | 0.25 | 2.79 |
| Number and (%) of participants with detected IFNy response (fold increase on 1 day/fold increase on 28 day>1.1) | 11/11 (100) | | 32/33 (97.0) | | 43/44 (97.7) | | 13/13 (100) | | 27/28 (96.4) | | 40/41 (97.6) | |

**Supplementary Figure 1. Antigen-binding antibody response in all participants vaccinated with 1/10 or 1/5 dose of Sputnik V.**

Anti-RBD (A) and anti-S (B) SARS-CoV-2 IgG antibodies before immunization (day 1) and on days 21 (before vaccination with component B), 28, 42, 90, 180 in all participants vaccinated with 1/10 or 1/5 doses of “Sputnik V. Dots represent individual data points. Horizontal lines represent geometric mean titers, whiskers are 95% CIs. Significant differences between participants vaccinated with 1/10 and 1/5 doses are indicated by asterisks and lines (* for p<0.05, ** for p<0.01, Mann–Whitney U test). NS – not significant.


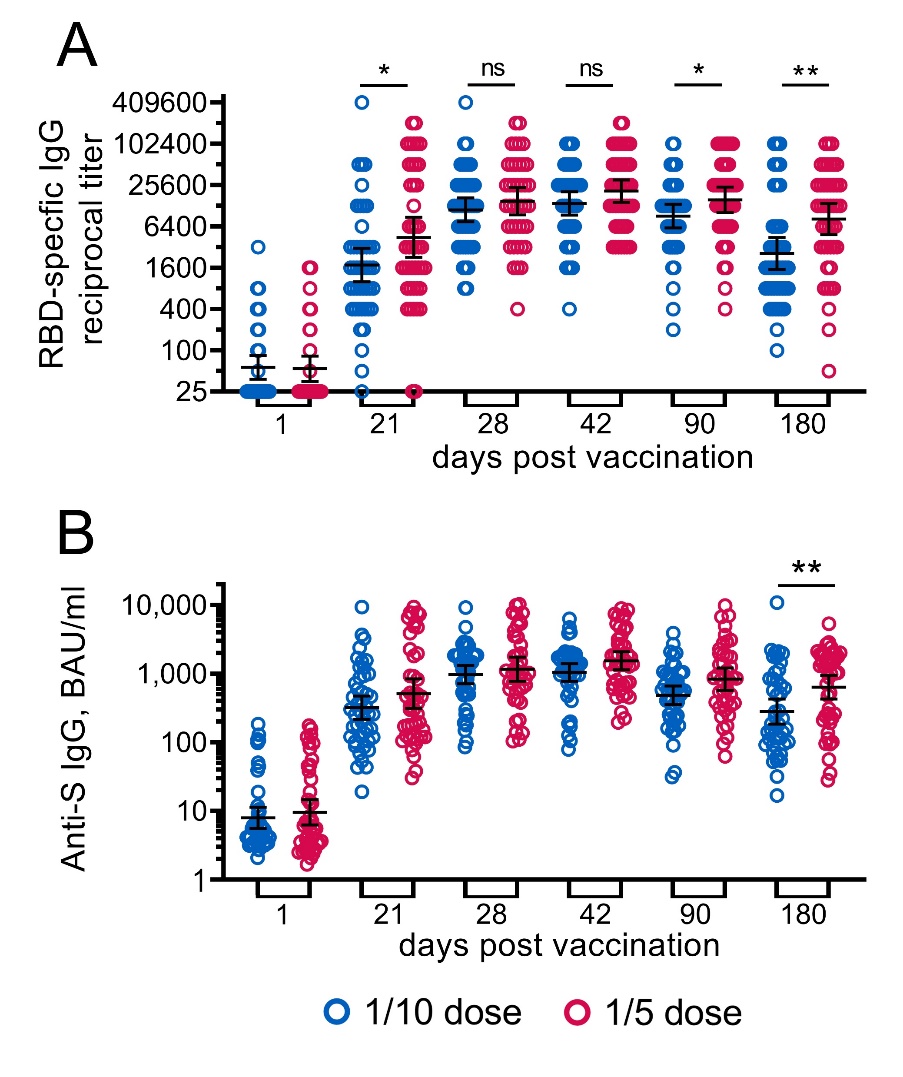


**Supplementary Figure 2. Antigen-binding antibody response in the 12-to-14-year-old and 15-to-17-year-old participants vaccinated with 1/10 or 1/5 dose of Sputnik V.**

Anti-RBD SARS-CoV-2 IgG antibodies before immunization (day 1) and on days 21 (before vaccination with component B), 28, 42, 90, and 180 in 12–14 y.o (A) and 15–17 y.o. (B) participants vaccinated with 1/10 and 1/5 dose of “Sputnik V. Dots represent individual data points. Horizontal lines represent geometric mean titers, and whiskers are 95% CIs. Significant differences between participants vaccinated with 1/10 and 1/5 doses are indicated by asterisks and lines (* for p<0.05, Mann–Whitney U test). NS – not significant.


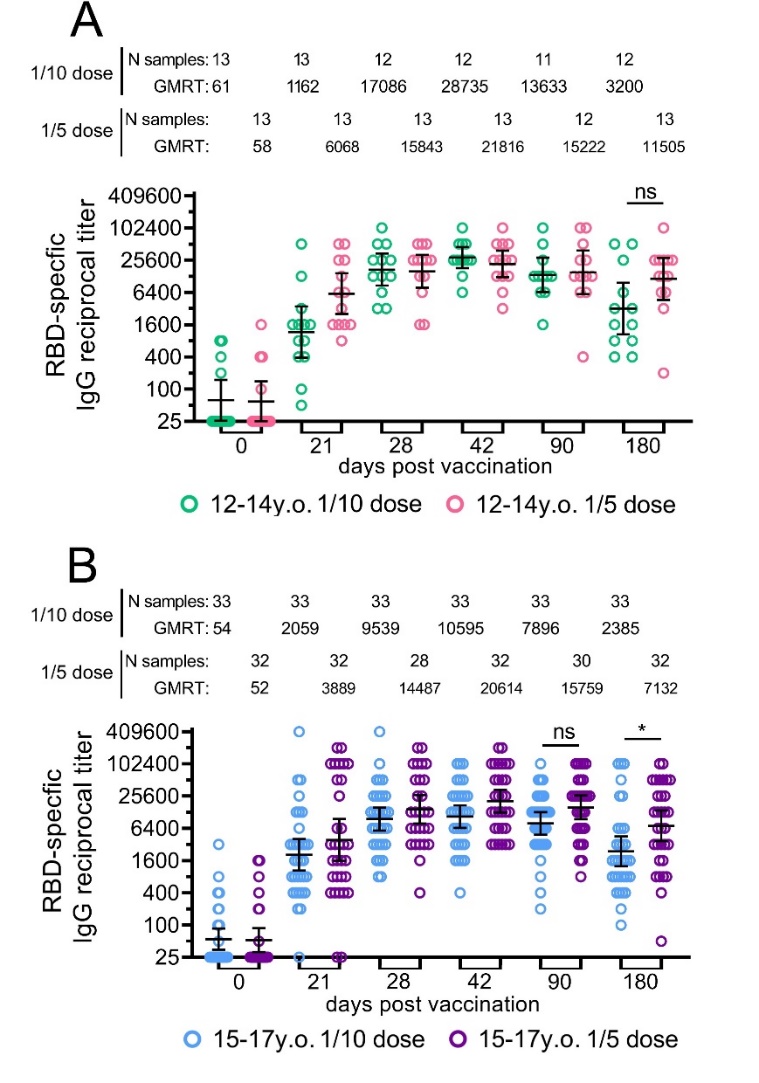


**Table S8. Seroconversion rate and statistic data (median, 25% and 75% percentile, geometric mean and 95% CI of geometric mean) of RBD-specific antibodies before (day 1) and on day 21, 28, 42, 90 and 180 after administration of 1/10 dose of vaccine as measured by ELISA in endpoint titers, in all participants as well as stratified by age.** Seroconversion was defined by ≥4-fold increase in antigen-specific IgG titer between pre and post vaccination time points. NA, non applicable.

|  | | N participants | Seroconversion rate, N (%) | 25% Percentile | Median | 75% Percentile | Geometric mean | Lower 95% CI of geo. mean | Upper 95% CI of geo. mean |
| --- | --- | --- | --- | --- | --- | --- | --- | --- | --- |
| 12-14 y.o. | 1 day | 13 | NA | 25 | 25 | 300 | 61.89 | 25.71 | 149 |
|  | 21 day | 13 | 11 (84.6) | 400 | 1600 | 2400 | 1162 | 385.6 | 3502 |
|  | 28 day | 12 | 12 (100) | 8000 | 19200 | 44800 | 17086 | 8579 | 34028 |
|  | 42 day | 12 | 12 (100) | 25600 | 25600 | 51200 | 28735 | 18257 | 45226 |
|  | 90 day | 11 | 11 (100) | 6400 | 12800 | 25600 | 13633 | 6537 | 9719 |
|  | 180 day | 12 | 11 (91.7) | 800 | 2400 | 20800 | 3200 | 1054 | 9719 |
| 15-17 y.o. | 1 day | 33 | NA | 25 | 25 | 100 | 2059 | 1045 | 4056 |
|  | 21 day | 33 | 31 (93.9) | 600 | 1600 | 4800 | 2059 | 1045 | 4056 |
|  | 28 day | 33 | 33 (100) | 3200 | 12800 | 25600 | 9539 | 5812 | 15656 |
|  | 42 day | 33 | 33 (100) | 3200 | 12800 | 25600 | 10595 | 6524 | 17206 |
|  | 90 day | 33 | 33 (100) | 3200 | 12800 | 19200 | 7896 | 4858 | 12833 |
|  | 180 day | 33 | 30 (90.1) | 800 | 1600 | 6400 | 2385 | 1255 | 45300 |
| 12-17 y.o. | 1 day | 46 | NA | 25 | 25 | 125 | 56.41 | 38.05 | 83.62 |
|  | 21 day | 46 | 42 (91.3) | 400 | 1600 | 3200 | 1751 | 1000 | 3067 |
|  | 28 day | 45 | 45 (100) | 3200 | 12800 | 25600 | 13825 | 9359 | 20422 |
|  | 42 day | 45 | 45 (100) | 6400 | 12800 | 25600 | 13825 | 9359 | 20422 |
|  | 90 day | 44 | 44 (100) | 4000 | 12800 | 22400 | 9051 | 6066 | 13504 |
|  | 180 day | 45 | 41 (91.1) | 800 | 1600 | 6400 | 2579 | 1512 | 4401 |

**Table S9. Seroconversion rate and statistic data (median, 25% and 75% percentile, geometric mean and 95% CI of geometric mean) of RBD-specific antibodies before (day 1) and on day 21, 28, 42, 90 and 180 after administration of 1/5 dose of vaccine as measured by ELISA in endpoint titers, in all participants as well as stratified by age.** Titer of 25 is a baseline characteristic (starting point of titration 1:50 gave zero signal). Seroconversion was defined by ≥4-fold increase in antigen-specific IgG titer between pre and post vaccination time points. NA, non applicable.

|  | | N participants | Seroconversion rate, N (%) | 25% Percentile | Median | 75% Percentile | Geometric mean | Lower 95% CI of geo. mean | Upper 95% CI of geo. mean |
| --- | --- | --- | --- | --- | --- | --- | --- | --- | --- |
| 12-14 y.o. | 1 day | 13 | NA | 25 | 25 | 250 | 58.67 | 24.47 | 140.7 |
|  | 21 day | 13 | 13 (100) | 1600 | 6400 | 25600 | 6068 | 2518 | 14623 |
|  | 28 day | 13 | 13 (100) | 9600 | 25600 | 38400 | 15843 | 7766 | 32321 |
|  | 42 day | 13 | 13 (100) | 12800 | 25600 | 51200 | 21816 | 12324 | 38619 |
|  | 90 day | 12 | 12 (100) | 8000 | 12800 | 44800 | 15222 | 5938 | 39018 |
|  | 180 day | 13 | 12 (92.3) | 6400 | 12800 | 25600 | 11505 | 4667 | 28365 |
| 15-17 y.o. | 1 day | 32 | NA | 25 | 25 | 43.75 | 52.21 | 31.18 | 87.43 |
|  | 21 day | 32 | 30 (93.75) | 800 | 3200 | 51200 | 3889 | 1584 | 9548 |
|  | 28 day | 28 | 28 (100) | 4000 | 12800 | 51200 | 14487 | 7854 | 26722 |
|  | 42 day | 32 | 32 (100) | 6400 | 19200 | 89600 | 20614 | 12637 | 33628 |
|  | 90 day | 30 | 30 (100) | 6400 | 25600 | 51200 | 15759 | 9469 | 26225 |
|  | 180 day | 32 | 31 (96.88) | 2000 | 9600 | 44800 | 7132 | 3686 | 13799 |
| 12-17 y.o. | 1 day | 45 | NA | 25 | 25 | 75 | 54 | 35.26 | 82.72 |
|  | 21 day | 45 | 45 (95.6) | 800 | 3200 | 38400 | 4422 | 2260 | 8653 |
|  | 28 day | 41 | 41 (100) | 6400 | 12800 | 51200 | 14904 | 9430 | 23553 |
|  | 42 day | 45 | 45 (100) | 6400 | 25600 | 51200 | 20954 | 14429 | 30431 |
|  | 90 day | 42 | 42 (100) | 6400 | 19200 | 51200 | 15603 | 10149 | 23989 |
|  | 180 day | 45 | 45 (95.6) | 3200 | 12800 | 25600 | 8189 | 4859 | 13799 |

**Table S10. Seroconversion rate and statistic data (median, 25% and 75% percentile, geometric mean and 95% CI of geometric mean) of S-specific antibodies before (day 1) and on day 21, 28, 42, 90 and 180 after administration of 1/10 dose of vaccine as measured by ELISA in BAU/ml, in all participants as well as stratified by age.**

A cut-off of 10 BAU/mL was used to determine the seroconversion (positive result by the method manufacturer). Levels of more than 154 BAU/ml correlate with a high neutralization capability against WT virus (protective threshold) (3)

|  | | N participants | Seroconversion rate,  >10 BAU/ml,  N (%) | Seroconversion rate,  >154 BAU/ml,  N (%) | 25% Percentile | Median | 75% Percentile | Geometric mean | Lower 95% CI of geo. mean | Upper 95% CI of geo. mean |
| --- | --- | --- | --- | --- | --- | --- | --- | --- | --- | --- |
| 12-14 y.o. | 1 day | 13 | 4 (30.77) | 0 | 3.263 | 4.466 | 27.62 | 8.175 | 3.739 | 17.87 |
|  | 21 day | 13 | 13 (100) | 10 (76.92) | 180.1 | 291.6 | 385 | 286.3 | 153.2 | 535 |
|  | 28 day | 12 | 12 (100) | 12 (100) | 738.6 | 1663 | 2372 | 1359 | 921 | 2006 |
|  | 42 day | 12 | 12 (100) | 12 (100) | 1021 | 1484 | 2018 | 1481 | 1049 | 2092 |
|  | 90 day | 11 | 11 (100) | 11 (100) | 408.1 | 582.3 | 645 | 582.1 | 366.6 | 924.1 |
|  | 180 day | 12 | 12 (100) | 6 (50) | 80.91 | 163.4 | 1482 | 242.3 | 96.45 | 608.9 |
| 15-17 y.o. | 1 day | 33 | 7 (21.2) | 1 (3.03) | 3.876 | 5.257 | 8.459 | 7.875 | 5.215 | 11.89 |
|  | 21 day | 33 | 33 (100) | 22 (66.67) | 112 | 253.6 | 965.3 | 334.5 | 921 | 2006 |
|  | 28 day | 33 | 33 (100) | 30 (90.91) | 394.9 | 1169 | 1846 | 860.7 | 581.3 | 1274 |
|  | 42 day | 33 | 33 (100) | 30 (90.91) | 560 | 1246 | 1796 | 922.3 | 630.8 | 1349 |
|  | 90 day | 33 | 33 (100) | 28 (84.85) | 219.7 | 549.1 | 1023 | 455.4 | 305.4 | 679.2 |
|  | 180 day | 33 | 33 (100) | 21 (63.64) | 98.78 | 223.4 | 850.3 | 297.4 | 181.1 | 488.4 |
| 12-17 y.o. | 1 day | 46 | 11 (23.91) | 1 (2.17) | 3.714 | 4.989 | 10.01 | 7.959 | 5.595 | 11.32 |
|  | 21 day | 46 | 46 (100) | 32(69.56) | 120.9 | 275.4 | 892.5 | 515.9 | 314.1 | 847.3 |
|  | 28 day | 45 | 45 (100) | 42 (93.33) | 538 | 1234 | 1860 | 972.2 | 717.1 | 1318 |
|  | 42 day | 45 | 45 (100) | 42 (93.33) | 790 | 1354 | 1886 | 1046 | 780.4 | 1403 |
|  | 90 day | 44 | 44 (100) | 39 (88.64) | 239 | 575.9 | 979.2 | 484.3 | 353.8 | 662.9 |
|  | 180 day | 45 | 45 (100) | 27 (60) | 98.78 | 185.7 | 850.3 | 281.6 | 184.9 | 428.7 |

**Table S11. Seroconversion rate and statistic data (median, 25% and 75% percentile, geometric mean and 95% CI of geometric mean) of S-specific antibodies before (day 1) and on day 21, 28, 42, 90 and 180 after administration of 1/5 dose of vaccine as measured by ELISA in BAU/ml, in all participants as well as stratified by age strata.** A cut-off of 10 BAU/mL was used to determine the seroconversion (positive result by the method manufacturer). Levels of more than 154 BAU/ml correlate with a high neutralization capability against WT virus (protective threshold) (3).

|  | | N participants | Seroconversion rate,  <10 BAU/ml,  N (%) | Seroconversion rate,  <154 BAU/ml,  N (%) | 25% Percentile | Median | 75% Percentile | Geometric mean | Lower 95% CI of geo. mean | Upper 95% CI of geo. mean |
| --- | --- | --- | --- | --- | --- | --- | --- | --- | --- | --- |
| 12-14 y.o. | 1 day | 13 | 4 (30.77) | 0 | 2.785 | 6.135 | 26.31 | 8.805 | 3.894 | 19.91 |
|  | 21 day | 13 | 13 (100) | 10 (76.92) | 189.8 | 470.3 | 1454 | 536.2 | 243.7 | 1180 |
|  | 28 day | 13 | 13 (100) | 11 (84.62) | 588 | 1173 | 2302 | 1034 | 509.1 | 2099 |
|  | 42 day | 13 | 13 (100) | 13 (100) | 1075 | 1889 | 2946 | 1758 | 1139 | 2713 |
|  | 90 day | 12 | 12 (100) | 11 (91.67) | 432.8 | 1085 | 1876 | 967.4 | 483.2 | 1937 |
|  | 180 day | 13 | 13 (100) | 11 (84.62) | 848.8 | 1716 | 1996 | 1034 | 487 | 2197 |
| 15-17 y.o. | 1 day | 32 | 12 (37.5) | 1 (3.125) | 3.384 | 5.540 | 41.51 | 9.930 | 5.9 | 16.71 |
|  | 21 day | 32 | 32 (100) | 22 (68.75) | 121.4 | 356.2 | 3498 | 507.8 | 266.1 | 969 |
|  | 28 day | 28 | 28 (100) | 26 (92.86) | 554.2 | 1003 | 4938 | 1232 | 733.1 | 2071 |
|  | 42 day | 32 | 32 (100) | 32 (100) | 623.7 | 1382 | 4481 | 1468 | 982.3 | 2193 |
|  | 90 day | 30 | 30 (100) | 28 (93.33) | 316.4 | 866.6 | 2057 | 757.2 | 493.4 | 1256 |
|  | 180 day | 32 | 32 (100) | 25 (78.125) | 197.3 | 707.5 | 1463 | 525.7 | 326.5 | 846.3 |
| 12-17 y.o. | 1 day | 45 | 16 (35.56) | 1 (2.22) | 3.263 | 5.569 | 33.67 | 9.591 | 6.292 | 14.62 |
|  | 21 day | 45 | 45 (100) | 32 (71.11) | 123.1 | 375.1 | 2126 | 515.9 | 314.1 | 847.3 |
|  | 28 day | 41 | 41 (100) | 37 (90.24) | 567.4 | 1055 | 3489 | 1165 | 778.9 | 1744 |
|  | 42 day | 45 | 45 (100) | 45 (100) | 790 | 1354 | 1886 | 1046 | 780.4 | 1403 |
|  | 90 day | 42 | 42 (100) | 39 (92.85) | 367.5 | 866.6 | 1911 | 835 | 574.7 | 1213 |
|  | 180 day | 45 | 45 (100) | 36 (80) | 224.7 | 1041 | 1869 | 639.2 | 429.6 | 951 |

**Supplementary Figure 3. Comparative analysis of RBD-specific IgG response between all the participants and in the participants having no pre-existing immunity to SARS-CoV-2 during the study.**

Anti-RBD SARS-CoV-2 IgG antibodies before immunization (day 1) and on days 21 (before vaccination with component B), 28, 42, 90, 180 in all (A) as well as in seronegative participants (without N-specific IgGs) (B) vaccinated with 1/10 or 1/5 dose of Sputnik V. Dots represent individual data points. Horizontal lines represent geometric mean titers, and whiskers are 95% CIs. Significant differences between participants vaccinated with 1/10 and 1/5 doses are indicated by asterisks and lines (* for p<0.05, ** for p<0.01, Mann–Whitney U test). NS – not significant.


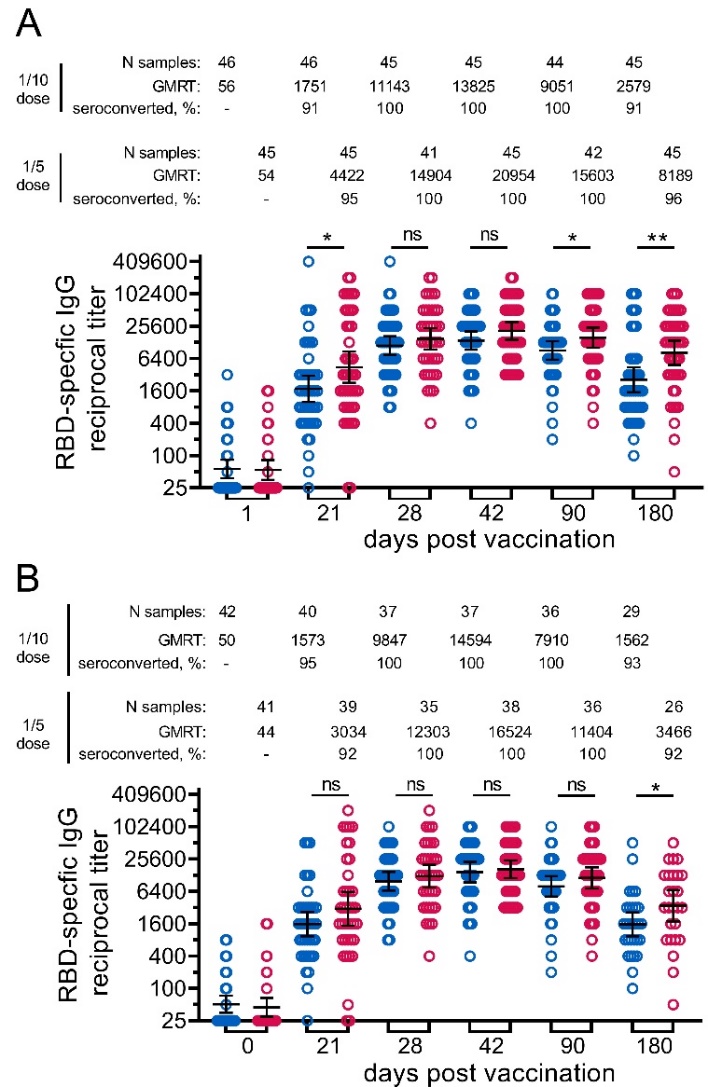


**Table S12. Seroconversion rate and statistic data (median, 25% and 75% percentile, geometric mean and 95% CI of geometric mean) of RBD-specific antibodies before (day 1) and on day 21, 28, 42, 90 and 180 after administration of 1/10 dose of vaccine as measured by ELISA in endpoint titers, in seronegative participants (without N-specific IgGs) as well as stratified by age.** Seroconversion was defined by ≥4-fold increase in antigen-specific IgG titer between pre and post vaccination time points. NA, non applicable.

|  | | N participants | Seroconversion rate, N (%) | 25% Percentile | Median | 75% Percentile | Geometric mean | Lower 95% CI of geo. mean | Upper 95% CI of geo. mean |
| --- | --- | --- | --- | --- | --- | --- | --- | --- | --- |
| 12-14 y.o. | 1 day | 13 | NA | 25 | 25 | 300 | 61.89 | 25.71 | 149 |
|  | 21 day | 13 | 12 (92.3) | 600 | 1600 | 8000 | 1688 | 609.6 | 4672 |
|  | 28 day | 13 | 13 (100) | 4800 | 12800 | 25600 | 13501 | 7046 | 25869 |
|  | 42 day | 12 | 12 (100) | 25600 | 25600 | 44800 | 25600 | 15576 | 42074 |
|  | 90 day | 11 | 11 (100) | 6400 | 12800 | 25600 | 13633 | 6537 | 28429 |
|  | 180 day | 10 | 9 (90) | 800 | 2400 | 11200 | 2986 | 986,4 | 9037 |
| 15-17 y.o. | 1 day | 29 | NA | 25 | 25 | 100 | 46.54 | 31.17 | 69.48 |
|  | 21 day | 27 | 26 (96.3) | 400 | 1600 | 3200 | 1520 | 797.5 | 2897 |
|  | 28 day | 24 | 24 (100) | 3200 | 9600 | 22400 | 8300 | 4882 | 14111 |
|  | 42 day | 25 | 25 (100) | 3200 | 12800 | 38400 | 11143 | 6142 | 20215 |
|  | 90 day | 25 | 25 (100) | 3200 | 6400 | 12800 | 6225 | 3611 | 10732 |
|  | 180 day | 19 | 18 (94.7) | 400 | 1600 | 3200 | 1111 | 648.5 | 1903 |
| 12-17 y.o. | 1 day | 42 | NA | 25 | 25 | 100 | 50.83 | 35.18 | 73.44 |
|  | 21 day | 40 | 38 (95) | 500 | 1600 | 3200 | 1573 | 934.5 | 2646 |
|  | 28 day | 37 | 37 (100) | 3200 | 12800 | 25600 | 9847 | 6590 | 14713 |
|  | 42 day | 37 | 37 (100) | 6400 | 25600 | 38400 | 14594 | 9412 | 22628 |
|  | 90 day | 36 | 36 (100) | 4000 | 12800 | 12800 | 7910 | 5108 | 12247 |
|  | 180 day | 29 | 27 (93.1) | 800 | 1600 | 3200 | 1562 | 937.6 | 2603 |

**Table S13. Seroconversion rate and statistic data (median, 25% and 75% percentile, geometric mean and 95% CI of geometric mean) of RBD-specific antibodies before (day 1) and on day 21, 28, 42, 90 and 180 after administration of 1/5 dose of vaccine as measured by ELISA in endpoint titers, in seronegative participants (without N-specific IgGs) as well as stratified by age**. Seroconversion was defined by ≥4-fold increase in antigen-specific IgG titer between pre and post vaccination time points. NA, non applicable.

|  | | N participants | Seroconversion rate, N (%) | 25% Percentile | Median | 75% Percentile | Geometric mean | Lower 95% CI of geo. mean | Upper 95% CI of geo. mean |
| --- | --- | --- | --- | --- | --- | --- | --- | --- | --- |
| 12-14 y.o. | 1 day | 11 | NA | 25 | 25 | 100 | 53.25 | 20.30 | 139.7 |
|  | 21 day | 11 | 11 (100) | 1600 | 3200 | 25600 | 4974 | 1904 | 12997 |
|  | 28 day | 11 | 11 (100) | 6400 | 25600 | 25600 | 13633 | 6012 | 30912 |
|  | 42 day | 9 | 9 (100) | 9600 | 12800 | 38400 | 16127 | 7970 | 32633 |
|  | 90 day | 7 | 7 (100) | 6400 | 12800 | 12800 | 7066 | 2022 | 24693 |
|  | 180 day | 6 | 5 (83.3) | 2450 | 6400 | 16000 | 4525 | 773.2 | 26486 |
| 15-17 y.o. | 1 day | 30 | NA | 25 | 25 | 25 | 41.56 | 26.35 | 65.55 |
|  | 21 day | 28 | 25 (89.3) | 500 | 2400 | 6400 | 2498 | 962 | 6488 |
|  | 28 day | 24 | 24 (100) | 3200 | 12800 | 44800 | 11738 | 6177 | 22303 |
|  | 42 day | 29 | 29 (100) | 6400 | 12800 | 51200 | 16649 | 10410 | 26628 |
|  | 90 day | 29 | 29 (100) | 4800 | 12800 | 25600 | 12800 | 7663 | 21380 |
|  | 180 day | 20 | 19 (95) | 800 | 3200 | 12800 | 3200 | 1446 | 7084 |
| 12-17 y.o. | 1 day | 41 | NA | 25 | 25 | 25 | 44.42 | 29.77 | 66.28 |
|  | 21 day | 39 | 36 (92.3) | 800 | 3200 | 12800 | 3034 | 1476 | 6236 |
|  | 28 day | 35 | 35 (100) | 3200 | 12800 | 25600 | 12303 | 7564 | 20010 |
|  | 42 day | 38 | 38 (100) | 6400 | 12800 | 51200 | 16524 | 11302 | 24159 |
|  | 90 day | 36 | 36 (100) | 6400 | 12800 | 25600 | 11404 | 7219 | 18013 |
|  | 180 day | 26 | 24 (92.3) | 800 | 3200 | 12800 | 3466 | 1768 | 6798 |

**Supplementary Figure 4. Antigen-binding antibody response in participants vaccinated with 1/10 or 1/5 dose of “Sputnik V” stratified by age.** Anti-RBD SARS-CoV-2 IgG antibodies before immunization (day 1) and on days 21 (before vaccination with component B), 28, 42, 90, 180 in 12-14 y.o and 15-17 y.o. participants vaccinated with 1/10 (A) and 1/5 (B) dose of “Sputnik V. Dots represent individual data points. Horizontal lines represent geometric mean titers, whiskers are 95% CIs. Significant differences between participants vaccinated with 1/10 and 1/5 dose are indicated by asterisks and lines (* for p<0.05, Mann–Whitney U test). NS, not significant.

**
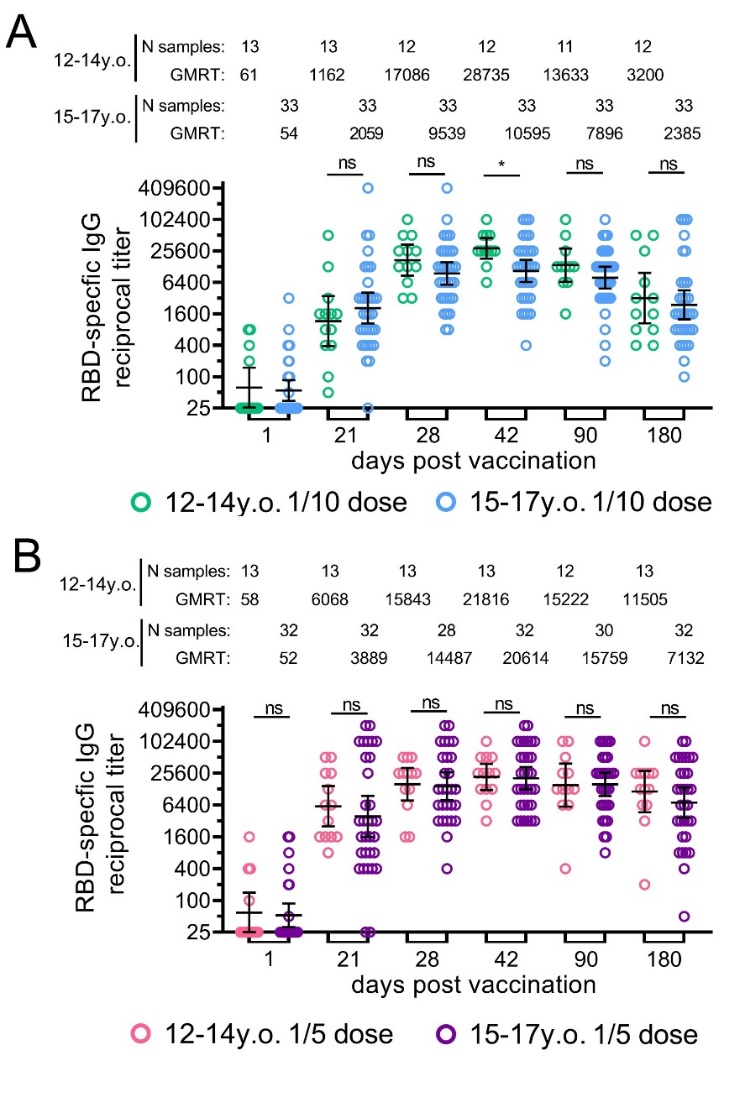
**

**Supplementary Figure 5. Comparative analysis of neutralizing antibody response in participants vaccinated with 1/10 or 1/5 dose of Sputnik V.** Neutralizing antibodies before immunization (day 1) and on days 21 (before vaccination with component B), 28, 42, 90, 180, as measured by microneutralization assay with 100 TCID50, in all participants vaccinated with 1/10 or 1/5 dose of “Sputnik V. Dots represent individual data points. Horizontal lines represent geometric mean titers, and whiskers are 95% CIs. Significant difference between participants vaccinated with 1/10 and 1/5 dose is indicated by an asterisk (* for p<0.05) and a line.


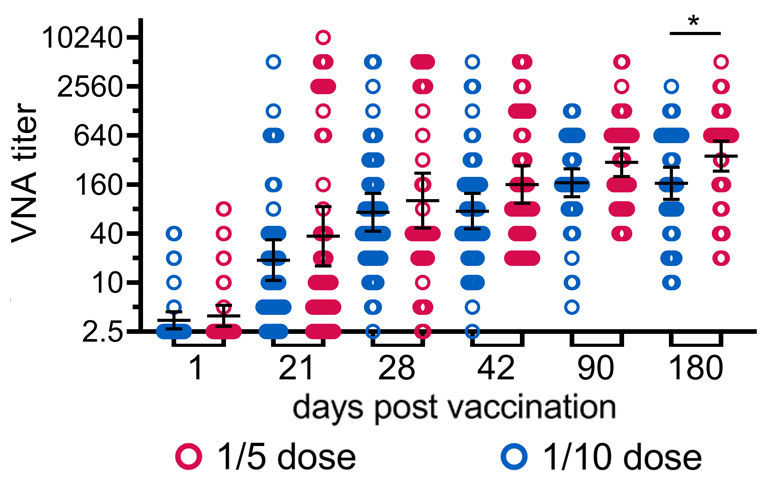


**Table S14. Seroconversion rate and statistic data (median, 25% and 75% percentile, geometric mean and 95% CI of geometric mean) of SARS-Cov2** **neutralizing antibodies before (day 1) and on day 21, 28, 42, 90 and 180 after administration of 1/10 dose of vaccine as measured by neutralisation assay with 100 TCID50, in all participants as well as stratified by age strata.** Titer of 2.5 is a baseline characteristic (starting point of titration 1:5 gave no protection). The volunteer was considered as “responder” to vaccination if the NtAb titer was ≥2-fold higher than before vaccination (neutralizing antibody seroconversion). NA, non applicable.

|  | | N participants | Seroconversion rate,  N (%) | 25% Percentile | Median | 75% Percentile | Geometric mean | Lower 95% CI of geo. mean | Upper 95% CI of geo. mean |
| --- | --- | --- | --- | --- | --- | --- | --- | --- | --- |
| 12-14 y.o. | 1 day | 13 | NA | 2.5 | 2.5 | 2.5 | 2.934 | 2.283 | 3.771 |
|  | 21 day | 13 | 11 (84,62) | 5 | 10 | 40 | 16.16 | 5.843 | 44.69 |
|  | 28 day | 12 | 12 (100) | 40 | 60 | 160 | 89.80 | 40.63 | 198.5 |
|  | 42 day | 12 | 12 (100) | 40 | 80 | 160 | 67.63 | 35.6 | 128.5 |
|  | 90 day | 11 | 11 (100) | 160 | 160 | 640 | 219.3 | 105.4 | 456 |
|  | 180 day | 12 | 12 (100) | 80 | 160 | 640 | 213.6 | 97.45 | 468.1 |
| 15-17 y.o. | 1 day | 33 | NA | 2.5 | 2.5 | 2.5 | 3.649 | 2.635 | 5.053 |
|  | 21 day | 33 | 27 (81,82) | 5 | 10 | 40 | 20.0 | 9.692 | 41.27 |
|  | 28 day | 33 | 32 (96,97) | 20 | 40 | 160 | 89.8 | 40.63 | 198.5 |
|  | 42 day | 33 | 32 (96,97) | 20 | 40 | 160 | 67.63 | 35.6 | 128.5 |
|  | 90 day | 33 | 33 (100) | 80 | 160 | 640 | 153.4 | 94.59 | 248.8 |
|  | 180 day | 33 | 33 (100) | 40 | 160 | 640 | 150.2 | 84.87 | 265.9 |
| 12-17 y.o. | 1 day | 46 | NA | 2.5 | 2.5 | 2.5 | 3.431 | 2.697 | 4.363 |
|  | 21 day | 46 | 38 (82.61) | 5 | 10 | 40 | 18.83 | 10.61 | 33.42 |
|  | 28 day | 45 | 44 (97.78) | 20 | 40 | 240 | 72.94 | 42.77 | 124.4 |
|  | 42 day | 45 | 44 (97.78) | 20 | 80 | 160 | 75.22 | 45.53 | 124.3 |
|  | 90 day | 44 | 44 (100) | 80 | 160 | 640 | 167.7 | 113 | 248.9 |
|  | 180 day | 45 | 45 (100) | 60 | 160 | 640 | 165.0 | 104.7 | 260 |

**Table S15. Seroconversion rate and statistic data (median, 25% and 75% percentile, geometric mean and 95% CI of geometric mean) of SARS-Cov2 neutralizing antibodies before (day 1) and on day 21, 28, 42, 90 and 180 after administration of 1/5 dose of vaccine as measured by neutralisation assay with 100 TCID50, in all participants as well as stratified by age strata.** Titer of 2.5 is a baseline characteristic (starting point of titration 1:5 gave no protection). The volunteer was considered as “responder” to vaccination if the NtAb titer was ≥2-fold higher than before vaccination (neutralizing antibody seroconversion). NA, non applicable.

|  | | N participants | Seroconversion rate,  N (%) | 25% Percentile | Median | 75% Percentile | Geometric mean | Lower 95% CI of geo. mean | Upper 95% CI of geo. mean |
| --- | --- | --- | --- | --- | --- | --- | --- | --- | --- |
| 12-14 y.o. | 1 day | 13 | NA | 2.5 | 2.5 | 2.5 | 3.094 | 19.44 | 4.925 |
|  | 21 day | 13 | 12 (92.31) | 5 | 10 | 120 | 4.297 | 2.924 | 6.313 |
|  | 28 day | 13 | 12 (92.31) | 25 | 40 | 240 | 64.63 | 20.17 | 207.1 |
|  | 42 day | 13 | 13 (100) | 80 | 80 | 320 | 143.8 | 68.46 | 302.1 |
|  | 90 day | 12 | 12 (100) | 100 | 400 | 640 | 254 | 118.9 | 542.5 |
|  | 180 day | 13 | 13 (100) | 480 | 640 | 960 | 545.4 | 274.3 | 1084 |
| 15-17 y.o. | 1 day | 32 | NA | 2.5 | 2.5 | 4.375 | 4.297 | 2.924 | 6.313 |
|  | 21 day | 32 | 24 (75) | 3.125 | 10 | 2240 | 42.69 | 14.34 | 127.1 |
|  | 28 day | 28 | 27 (96.43) | 20 | 40 | 2560 | 124.9 | 44.37 | 351.7 |
|  | 42 day | 32 | 32 (100) | 25 | 120 | 1280 | 167.1 | 82.42 | 338.7 |
|  | 90 day | 30 | 30 (100) | 80 | 320 | 640 | 320 | 194.2 | 527.3 |
|  | 180 day | 32 | 32 (100) | 80 | 640 | 640 | 299.9 | 175.4 | 512.6 |
| 12-17 y.o. | 1 day | 45 | NA | 2.5 | 2.5 | 2.5 | 3.908 | 2.9 | 5.266 |
|  | 21 day | 45 | 36 (80) | 5 | 10 | 640 | 37.03 | 16.06 | 85.41 |
|  | 28 day | 41 | 39 (95.12) | 20 | 40 | 1920 | 101.4 | 46.72 | 219.9 |
|  | 42 day | 45 | 45 (100) | 40 | 80 | 960 | 160 | 94.09 | 272.1 |
|  | 90 day | 42 | 42 (100) | 80 | 320 | 640 | 299.6 | 200.5 | 447.5 |
|  | 180 day | 45 | 45 (100) | 160 | 640 | 640 | 356.4 | 233.4 | 544.3 |

**Table S16*.* Serum neutralization titers against variants of SARS-CoV-2. Serum samples were obtained on day 180 after from participants received 1/10 (N=45) or 1/5 dose (N=45) of vaccine.** Virus cross-neutralizing antibodies against initial (B.1.1.1) genetic lineage variant, Delta (B.1.617.2), and Omicron (BA.5) SARS-Cov2 variants of concern were detected using microneutralization method. Titer of 2.5 is a baseline characteristic (starting point of titration 1:5 gave no protection. NA, non applicable.

|  | 1/10 dose (N=45) | | | 1/5 dose (N=45) | | |
| --- | --- | --- | --- | --- | --- | --- |
|  | B.1.1.1 | Delta  B.1.617.2 | Omicron BA.5 | B.1.1.1 | Delta  B.1.617.2 | Omicron BA.5 |
| 25% Percentile | 60 | 5 | 2.5 | 160 | 20 | 2.5 |
| Median | 160 | 20 | 2.5 | 640 | 160 | 20 |
| 75% Percentile | 640 | 80 | 10 | 640 | 480 | 60 |
| Geometric mean | 165 | 24.06 | 6.015 | 351 | 102.4 | 16.62 |
| Lower 95% CI of geo. mean | 104.7 | 13.28 | 4.040 | 227.4 | 57.75 | 10.49 |
| Upper 95% CI of geo. mean | 260 | 43.61 | 8.956 | 541.8 | 181.4 | 26.34 |
| Decrease in GMT comparing to initial strain, folds | NA | 6.86 | 27.43 | NA | 3.43 | 21.12 |
| Seroconversion, % (N) | 100 (45) | 84.4(38) | 42.2 (19) | 100 (45) | 97.8 (44) | 73.3 (33) |

**Table S17. Statistic data (median, 25% and 75% percentile, geometric mean and 95% CI of geometric mean) of NtAb against Ad26 before and on day 42 after administration of 1/10 and 1/5 dose of vaccine in all participants as well as stratified by age.** Titer of 12.5 is a baseline characteristic (starting point of titration 1:25 gave no protection). The volunteer was considered to be seroconverted if the NtAb titer was ≥4-fold higher than before vaccination.

|  | 1/10 dose | | | | | | 1/5 dose | | | | | |
| --- | --- | --- | --- | --- | --- | --- | --- | --- | --- | --- | --- | --- |
|  | 12-14 y.o. | | 15-17 y.o. | | 12-17 y.o. | | 12-14 y.o. | | 15-17 y.o. | | 12-17 y.o. | |
|  | 1 day | 42 day | 1 day | 42 day | 1 day | 42 day | 1 day | 42 day | 1 day | 42 day | 1 day | 42 day |
| Number of samples | 13 | 12 | 33 | 33 | 46 | 45 | 13 | 13 | 32 | 32 | 45 | 45 |
| 25% Percentile | 12.5 | 12.5 | 12.5 | 12.5 | 12.5 | 12.5 | 12.5 | 12.5 | 12.5 | 12.5 | 12.5 | 12.5 |
| Median | 12.5 | 12.5 | 12.5 | 12.5 | 12.5 | 12.5 | 12.5 | 12.5 | 12.5 | 31.25 | 12.5 | 12.5 |
| 75% Percentile | 12.5 | 100 | 12.5 | 25 | 12.5 | 12.5 | 12.5 | 50 | 12.5 | 50 | 12.5 | 50 |
| Geometric mean | 12.5 | 42.04 | 14.48 | 20.69 | 18.22 | 27.42 | 12.5 | 23.7 | 13.34 | 36.92 | 13.09 | 32.48 |
| Lower 95% confidence limit of geo.mean | 12.5 | 14.75 | 11.73 | 14.01 | 13.14 | 15.59 | 12.5 | 14.01 | 11.68 | 22.97 | 11.93 | 22.57 |
| Upper 95% confidence limit of geo.mean | 12.5 | 119.9 | 17.88 | 30.56 | 25.26 | 48.22 | 12.5 | 40.11 | 15.23 | 59.35 | 14.37 | 46.76 |
| Seroconversion rate, N (%) | 5/12 (41.67) | | 5/33 (15.15) | | 10/45 (22.22) | | 5/13 (38.46) | | 16/32 (50) | | 21/45 (46.67) | |

**Table S18. Statistic data (median, 25% and 75% percentile, geometric mean and 95% CI of geometric mean) of NtAb against Ad5 before and on day 42 after administration of 1/10 and 1/5 dose of vaccine in all participants as well as stratified by age.** Titer of 12.5 is a baseline characteristic (starting point of titration 1:25 gave no protection). The volunteer was considered to be seroconverted if the NtAb titer was ≥4-fold higher than before vaccination.

|  | 1/10 dose | | | | | | 1/5 dose | | | | | |
| --- | --- | --- | --- | --- | --- | --- | --- | --- | --- | --- | --- | --- |
|  | 12-14 y.o. | | 15-17 y.o. | | 12-17 y.o. | | 12-14 y.o. | | 15-17 y.o. | | 12-17 y.o. | |
|  | 1 day | 42 day | 1 day | 42 day | 1 day | 42 day | 1 day | 42 day | 1 day | 42 day | 1 day | 42 day |
| Number of samples | 13 | 12 | 33 | 33 | 46 | 45 | 13 | 13 | 32 | 32 | 45 | 45 |
| 25% Percentile | 12.5 | 12.5 | 12.5 | 12.5 | 12.5 | 12.5 | 12.5 | 12.5 | 12.5 | 12.5 | 12.5 | 12.5 |
| Median | 12.5 | 12.5 | 12.5 | 12.5 | 12.5 | 12.5 | 12.5 | 12.5 | 12.5 | 12.5 | 12.5 | 12.5 |
| 75% Percentile | 12.5 | 12.5 | 12.5 | 12.5 | 12.5 | 25 | 12.5 | 12.5 | 200 | 800 | 12.5 | 50 |
| Geometric mean | 12.5 | 12.5 | 21.13 | 36.49 | 13.89 | 25 | 22.47 | 30.94 | 37.73 | 59.46 | 13.09 | 32.48 |
| Lower 95% confidence limit of geo.mean | 12.5 | 12.5 | 17.14 | 11.73 | 11.95 | 16.98 | 9.42 | 7.931 | 19.11 | 23.24 | 11.93 | 22.57 |
| Upper 95% confidence limit of geo.mean | 12.5 | 12.5 | 77.65 | 17.88 | 16.14 | 36.82 | 53.6 | 120.7 | 74.49 | 152.1 | 14.37 | 46.76 |
| Seroconversion rate,  N (%) | 0 | | 5/33 (15.15) | | 5/45 (11.11) | | 1/13 (7.69) | | 5/32 (15.625) | | 6/45 (13.33) | |

**Supplementary Figure 6. Neutralizing antibody response to rAd26 and rAd5 vectors in participants vaccinated with 1/10 or 1/5 dose of Sputnik V.**

Neutralizing antibodies to rAd26 and rAd5 vectors before immunization (day 1) and on day 42 as measured by microneutralization test using recombinant Ad26-EGFP and Ad5-EGFP, in all participants vaccinated with 1/10 or 1/5 dose of Sputnik V. Dots represent individual data points. Horizontal lines represent geometric mean titers, whiskers are 95% CIs. Significant differences between different timepoints are indicated by asterisks and lines: * for p<0.05, ** for p<0.01. *** for p<0.001, or **** for p<0.0001.


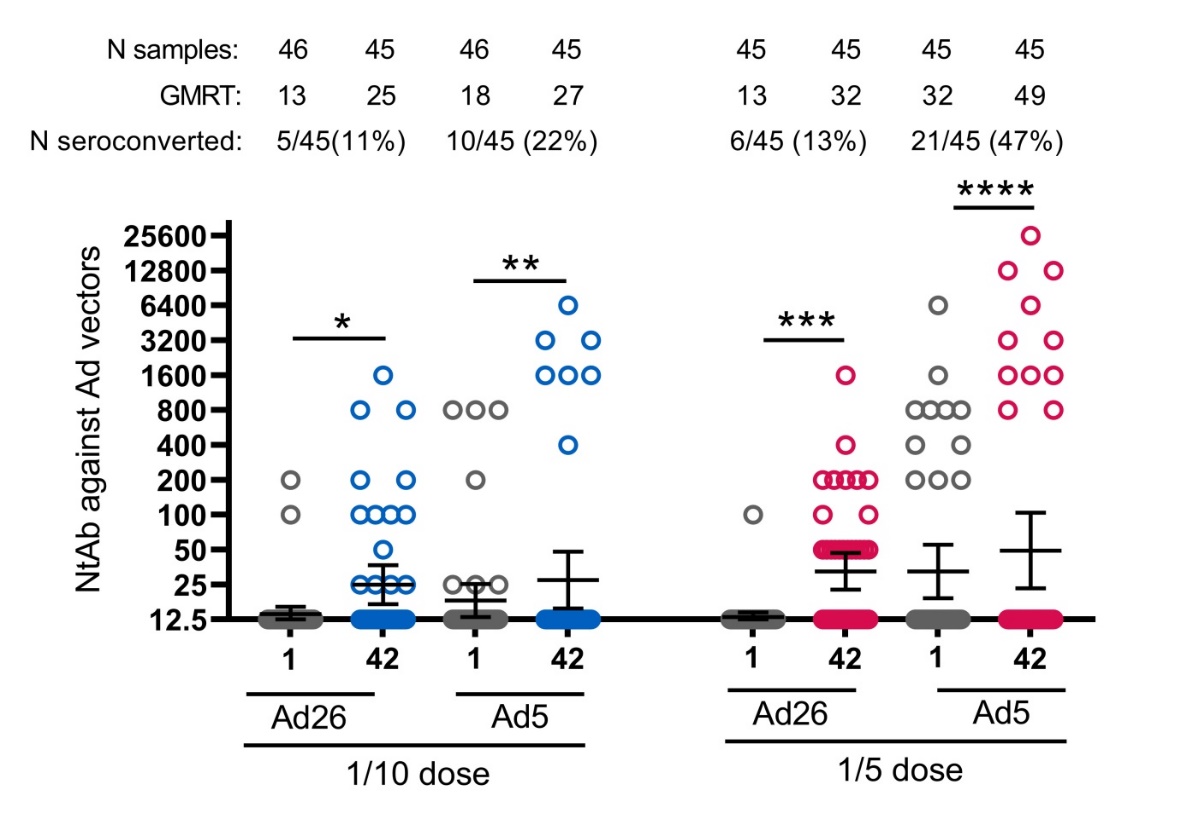


**† Supplementary Authors List – Vaccine Trial Group**

| Majkova D. Irina | The State Budgetary Institution of Healthcare of the City of Moscow "Children's City Clinical Hospital named after Z.A. Bashlyaeva of the Department of Health of the City of Moscow |
| --- | --- |
| Belous Y. Yuliya | The State Budgetary Institution of Healthcare of the City of Moscow "Children's City Clinical Hospital named after Z.A. Bashlyaeva of the Department of Health of the City of Moscow |
| Dmitriev I. Igor' | The State Budgetary Institution of Healthcare of the City of Moscow "Children's City Clinical Hospital named after Z.A. Bashlyaeva of the Department of Health of the City of Moscow |
| Smirnova V. Elena | The State Budgetary Institution of Healthcare of the City of Moscow "Morozov Children's City Clinical Hospital of the Department of Health of the City of Moscow" |
| Romanova V. Yulia | The State Budgetary Institution of Healthcare of the City of Moscow "Morozov Children's City Clinical Hospital of the Department of Health of the City of Moscow" |
| Rakhalina A. Antonina | The State Budgetary Institution of Healthcare of the City of Moscow "Morozov Children's City Clinical Hospital of the Department of Health of the City of Moscow" |
| Sharshakova A. Anastasia | The State Budgetary Institution of Healthcare of the City of Moscow "Morozov Children's City Clinical Hospital of the Department of Health of the City of Moscow" |
| Tikhonova N. Olga | The State Budgetary Institution of Healthcare of the City of Moscow "Morozov Children's City Clinical Hospital of the Department of Health of the City of Moscow" |
| Yablokova S. Yulia | The State Budgetary Institution of Healthcare of the City of Moscow "Morozov Children's City Clinical Hospital of the Department of Health of the City of Moscow" |
| Asalkhanova B. Saryuna | The State Budgetary Institution of Healthcare of the City of Moscow "Morozov Children's City Clinical Hospital of the Department of Health of the City of Moscow" |

**References**

1. Quah BJ, Warren HS, Parish CR. Monitoring lymphocyte proliferation in vitro and in vivo with the intracellular fluorescent dye carboxyfluorescein diacetate succinimidyl ester. Nat Protoc. 2007;2(9):2049-56.

2. Roederer M. Interpretation of cellular proliferation data: avoid the panglossian. Cytometry A. 2011;79(2):95-101.

3. Goldblatt D, Fiore-Gartland A, Johnson M, Hunt A, Bengt C, Zavadska D, et al. Towards a population-based threshold of protection for COVID-19 vaccines. Vaccine. 2022;40(2):306-15.
