# Supplementary material for: Safety and immunogenicity of rAd26 and rAd5 vector-based heterologous prime-boost COVID-19 vaccine against SARS-CoV-2 in healthy adolescents: an open-label, non-randomized, multicenter, phase 1/2, dose-escalation study
Source: Front Immunol. 2023 Aug 1;14:1228461. doi: 10.3389/fimmu.2023.1228461 (PMC10432829; doi:10.3389/fimmu.2023.1228461)
Supplement: Supplementary file 2 [file DataSheet_1.pdf]

CLINICAL STUDY PROTOCOL

**Study title:**	Double-blind, placebo-controlled study with an open dose selection period to assess the safety, tolerability, and immunogenicity of the drug "Gam-COVID-Vak M combined vector vaccine for the prevention of coronavirus infection caused by SARS-CoV-2 virus" in adolescents
**Protocol identification number:**	07-Gam-COVID-Vak-2021
**Protocol version:**	3.0
**Version date:**	23.07.2021
**Experimental drug:**	Gam-COVID-Vak M combined vector vaccine for the prevention of coronavirus infection caused by SARS-CoV-2 virus
**Study phase:**	I-II-III
**Study sponsor**	Federal Government Budgetary Institution "N.F. Gamaleya National Research Center for Epidemiology and Microbiology" of the Ministry of Health of the Russian Federation
**Drug developer**	Federal Government Budgetary Institution "National Research Center for Epidemiology and Microbiology n.a. Honorary Academician N.F. Gamaleya" of the Ministry of Health of the Russian Federation<br><br>**Legal address:**<br><br>**123098, Russia, Moscow, 18 Gamaleya St., Tel.: 8 (499) 193-30-01, Fax: 8 (499) 193-61-83**<br><br>**E-mail: info@gamaleya.org**

Privacy Notice

This document is the property of the Federal Government Budgetary Institution "N.F. Gamaleya National Research Center for Epidemiology and Microbiology" of the Ministry of Health of the Russian Federation. All information contained in this document is confidential and intended for researchers, members of ethical committees, and experts. Presentation of this document to third parties can be done only in compliance with the requirements of the legislation of the Russian Federation on personal data, commercial, state and other legally protected secrets.

The exception is cases of obtaining the informed consent of a volunteer to participate in the clinical study. This document can also be used to obtain approval by the current review committee of the institution involved in the conduct of the study.

Protocol approval by the study sponsor page

Protocol name: Double-blind, placebo-controlled study with an open dose selection period to assess the safety, tolerability, and immunogenicity of the drug “Gam-COVID-Vak M combined vector vaccine for the prevention of coronavirus infection caused by SARS-CoV-2 virus” in adolescents
Identification number: 07-Gam-COVID-Vak-2021
Protocol version: 3.0
Date: 23.07.2021
Sponsor representative:

A. L. Gintsburg
(Employee name)
Director of the Federal Government Budgetary Institution “N.F. Gamaleya National Research Center for Epidemiology and Microbiology” of the Ministry of Health of the Russian Federation
(Job position)

Name of the institution: Federal Government Budgetary Institution “N.F. Gamaleya National Research Center for Epidemiology and Microbiology” of the Ministry of Health of the Russian Federation
Address: 123098, Russia, Moscow, 18 Gamaleya St.
Telephone: 8 (499) 193-30-01
E-mail: info@gamaleya.org.

______________________________ __________________2021
Signature Date

Investigator’s informed consent with the study protocol page

I certify that I have read and understood this protocol, the investigator’s brochure, including potential risks and side effects of the drug, and other information about the drug and the study provided by the Sponsor.

I agree to conduct this study in accordance with the requirements of this protocol, and to protect the rights, safety, privacy, and health of volunteers in accordance with the ethical requirements set forth in the World Medical Association (WMA) Declaration of Helsinki, Federal Law of April 12, 2010. No. 61-FZ “On circulation of medicinal products”; the Rules of Good Clinical Practice (GCP) of the Eurasian Economic Union (EAEU), the requirements of the order of the Ministry of Health of the Russian Federation dated 01.04.2016 No. 200n “On Approval of the Rules of Good Clinical Practice”; the principles of the National Standard of the Russian Federation SUST 52379-2005 “Good Clinical Practice” and other regulatory requirements of the Russian Federation.

I agree to make changes to the protocol only upon notice to the Sponsor, except to the extent necessary to protect the safety, rights, or well-being of volunteers. I fully understand that any changes made by the Investigator(s) without prior discussion with the Sponsor’s representative will constitute a breach of protocol (other than those procedures necessary to preserve the health of the volunteers).

I agree to personally conduct or supervise the described study.

I agree to personally conduct or supervise the described study.

I agree to inform volunteers that drugs are used for research purposes; I will ensure compliance with informed consent requirements subject to the approval of the Ethics Council and the local Independent Ethics Committee (IEC) and in accordance with GCP principles.

In accordance with the GCP principles, I agree to report adverse events developing during the course of the study to the Sponsor.

I agree to ensure that all employees, colleagues, and individuals involved in the conduct of the study are informed of their obligations to comply with the agreements described above. I agree to maintain adequate and accurate records and to make these records available for review in accordance with the GCP principles.

I will ensure that the local IEC, which operates in accordance with GCP requirements, is responsible for conducting ethical review, as well as for approving the study. I also agree to promptly report any changes in research activity and any unexpected issues, including risk to volunteers and other issues, to the local IEC. In addition, I will not make any changes to the study without the approval of the Ethics Council/local IEC, except for the necessary cases of elimination of a clear unexpected threat to the life and health of volunteers.

I am ready to provide direct access to primary documents and agree to be audited by auditors from representatives of the Sponsor and regulatory authorities. I warrant that the research product(s) supplied by the Sponsor will only be used as described in this protocol.

I agree to comply with all other requirements regarding the responsibilities of clinical investigators, as well as all other essential requirements of the GCP.

Investigator:
Signature: ____
Date: _________________202_.
Full name:
Job position:
Institution:
Address:

List of changes made to the document since the release of the previous version

Version	Date	Changes
1.0	17.06.2021	Not applicable (initial version of the study protocol)
2.0	12.07.2021	Errors and inconsistencies in the text are corrected<br>Information on the independent data monitoring committee is added<br>The inclusion criterion for BMI has been adjusted: it was brought into line with age standards, Appendix No. 4 containing BMI tables for boys and girls aged 5–19 (WHO)<br>was added
3.0	23.07.2021	The number of participants was increased to 3,000 volunteers<br>Redesign. The second phase of the study will be conducted as a double-blind, placebo-controlled study<br>✓ 1:4 randomization<br>✓ Stratification into groups aged 12–14 and 15–17<br>✓ Blinding of all volunteers based on the results after 28 days<br>✓ Vaccination of the placebo group<br>✓ Continuation of observation according to the study plan for 1 year after vaccination:<br>• AE 42 days<br>• SAE throughout whole follow-up period<br>✓ Information about the placebo planned for use was added<br>✓ Laboratory studies of the immunogenicity were supplemented by a test for detection of antibodies against adenoviruses on day 180 (at the 1st stage) and before drug administration on days 42 and 180 (at the 2nd stage) to assess the impact of pre-existing immunity to the types of adenoviruses used in the vaccine<br>✓ Changes were made to the structure of payments to volunteers<br>Technical errors are corrected

Table of content

Table of Contents

Privacy Notice *1*

Protocol approval by the study sponsor page 2

Sponsor representative: *2*

Table of content 5

Abbreviations 9

Synopsis 11

19

Inclusion criteria *19*

1 General information 27

1.1 Protocol name, protocol identification number and date: 27

1.2 Administrative structure of the study 27

2 Rationale for the study 28

2.1 Experimental drug 28

Registration number: n/a *28*

Composition per 1 dose (0.5 ml): *28*

Dose: 28

Dose: 28

Description: *28*

There was no statistically significant difference in volunteers of different sex and age. Immunization with Gam-COVID-Vac forms intense antigen-specific cellular anti-infective immunity in almost all examined volunteers (formation of antigen-specific cells of both populations of T lymphocytes: T helper (CD4+) and T killer (CD8+) and a significant increase in IFNγ secretion) *29*

The protective antibody titer is currently unknown. The duration of protection is unknown. *29*

Clinical studies to study the epidemiological efficacy are currently ongoing. According to the interim analysis, the efficiency is more than 91%. *29*

Indications for use: *29*

Contraindications: *29*

The decision to vaccinate should be based on an assessment of the benefit/risk ratio in each specific situation *30*

Use during pregnancy and during breastfeeding *30*

Therapeutic measures in this case will include symptomatic therapy in accordance with the indications (antipyretic / NSAIDs and desensitizing agents), corticosteroids - parenterally for severe toxic-allergic syndrome). The regimen of prescribing drugs should be selected according to the recommendations for the use and dosages of this drug. *32*

Interaction with other drugs *32*

Not studied *32*

2.2 Placebo 32

Component II contains: *32*

2.3 Summary of non-clinical findings 32

Protection studies in sensitive animal species *34*

Conclusions based on the results of preclinical studies *35*

2.4 Summary of clinica study results 36

Safety, immunogenicity, and efficacy of the Gam-Kovid-Vak vaccine has been evaluated in studies: 36

2.4.1 Phase I/II studies 36

No severe AE were reported during the study. 38

AE outcomes 38

In general, it can be said that the AE identified during the first and second stages of the study are typical for most vaccine drugs. There were no cases of severe AE development. 39

2.4.2 Phase II, III, and IV study 42

Randomized, double-blind, placebo-controlled, multicenter clinical trial of the efficacy, immunogenicity, and safety of the Gam-COVID-Vac combined vector vaccine in parallel groups in the prevention of coronavirus infection caused by the SARS-CoV-2 virus" 42

Study period: 42

First Participant Screening: 07-Sep-2020 42

First Injection: 09-Sep-2020 42

Last randomized volunteer 29-Dec-2020 42

Study is currently ongoing 42

Study Goals and Endpoints 42

For qualitative and categorical indicators (gender, frequency of abnormalities according to physical examination data, incidence of adverse events, etc.), absolute values are presented in the n/N format, as well as the proportion (%). 46

Application of parametric and non-parametric statistical methods 46

Analysis of security settings parameters 47

Study is currently ongoing 47

Table 2–1. Seroconversion rate (geometric mean and 95% CI geometric mean) of RBD-specific antibodies at day 42, measured by ELISA, in volunteers immunized with vaccine (n = 342) or placebo (n = 114). Data are presented separately for groups stratified by age and gender of volunteers, as well as in general for vaccine and placebo groups. 49

Table 2–2. Seroconversion rate and statistical data (geometric mean and 95% CI geometric mean) of VNA at day 42 in participants immunized with vaccine (n = 72) or placebo (n = 28) as measured by microneutralization test. Data are presented in groups stratified by age and sex of volunteers and in general for vaccine and placebo groups. 50

Study is currently ongoing 51

2.5 Potential risks and benefits of investigational medicinal products for the study participant 51

2.6 Description and rationale for the route of administration, dosage, dosage regimen and course of treatment 59

2.7 Description of the study population 60

2.8 Rationale for the clinical study 60

2.9 Regulatory basis for conducting a clinical trial 65

2.9.1 Responsibility of the investigator 66

2.9.2 Medical care provided to volunteers in a clinical trial 67

3 Aims and objectives of the clinical trial 67

The study aim: 67

Research objectives and endpoints: 67

4 Description of the overall plan (design) of the clinical trial 69

4.1 Main and additional parameters of interest 69

4.2 Study design 71

4.2.1 Study type 71

4.2.2 Content of visits 71

Visit 3 (day 14): follow-up 76

Visit 4 (days 20–22 ± 1): vaccination 76

Visit 1 (day -7 – -1/0): screening *79*
Visit 2 (day 1): vaccination *82*
Visit 3 (day 21): vaccination *83*
Visit 5 (day 42): follow-up *84*
4.2.3 Graphic presentation of the study 85
4.2.4 Schedule of examination procedures in tabular form 87
2 stage .... *Ошибка! Закладка не определена.*
4.2.5 Study procedures 94
4.2.1 Handling of biological samples 98
4.2.2 Withdrawal of informed consent for submitted biological samples and data 99
4.2.3 Monitoring compliance with procedures 99
4.3 Duration of participation of volunteers in the study 100
4.4 Rules for Stopping Parts of a Clinical Study and/or the Clinical Study as a Whole 100
5 Study population 101
5.1 Inclusion criteria 101
5.2 Exclusion Criteria 102
5.3 Exclusion Criteria 103
5.4 Procedure and conditions for the inclusion of volunteers 104
5.5 Compliance control 105
5.6 Authorized concomitant therapy and contraception 105
5.7 Concomitant and prior therapy, lifestyle 105
6 Information about the medicinal products used in the clinical trial 106
6.1 Experimental drug 106
Experimental drug: *106*
6.2 Measures to minimize subjectivity 106
6.3 Experimental drug regimen 106
6.4 Dosage form, packaging and labeling of the investigational product 106
6.5 Investigational medicinal product accounting procedures 107
6.6 Storage of randomization codes and procedures for their disclosure 107
6.6.1 Distribution by study group 107
6.6.2 Revealing randomization codes 107
7 Assessment of immunogenicity parameters 108
8 Evaluation of safety parameters 108
8.1 List of safety parameters 108
8.2 Methods and timing for assessing, recording and analyzing safety parameters 110
8.3 Requirements for reporting, registration and reporting of adverse events and intercurrent diseases 110
8.3.1 Definition of adverse events and serious adverse events 110
Life threat *111*
8.3.2 Adverse events of particular interest 111
8.3.3 AE intensity 111
8.3.4 Causal relationship to investigational product 111
Guidance on interpreting the question of causality *112*
8.3.5 Documentation of adverse events 113
8.3.6 Teports of serious adverse events 114
8.4 Pregnancy 115
8.5 Method and duration of follow-up of clinical study participants after the occurrence of adverse events 116

9 Statistical aspects of the clinical study 116

9.1 Description of statistical analysis methods 116

9.1.1 Demographic and other background data (group comparability for analysis) 117

9.1.2 Analysis of the primary security parameter 117

9.1.3 Analysis of immunogenicity parameters 117

9.1.4 Analysis of other safety, tolerability and reactogenicity parameters 119

9.2 Timing of the interim review 119

9.3 Planned number of clinical trial participants with justification for sample size 120

Probability of seeing at least 1 AE based on the expected frequency of AE........ 120

9.4 Applicable level of significance of the clinical investigation 121

9.5 Criteria for termination of a clinical trial 121

9.6 Procedures for handling missing, non-analyzable and doubtful data 121

9.7 Procedures for reporting any deviations from the original statistical plan 121

9.8 Procedures for selecting clinical trial participants for analysis 122

10 Description of quality control and quality assurance activities 122

10.1 Study monitoring and quality control 122

10.2 Documentation 123

10.3 Amendments to the protocol 125

10.4 Protocol deviations 125

Procedure for documenting deviations from the Protocol 126

10.5 List of data recorded in an individual registration card (without prior written or electronic recording) and considered as primary data 126

11 Description of the ethical aspects of the clinical trial 127

11.1 General 127

11.2 Procedure for obtaining an informed consent 127

11.3 Confidentiality and identification of research subjects 128

11.4 Recruitment of subjects from vulnerable and special groups 128

12 Description of data handling and record keeping 129

12.1 Direct access to primary data/documentation 129

13 Financing and insurance 130

13.1 Funding 130

13.2 Insurance 130

14 Independent data monitoring committee 131

15 Publications 135

16 References 134

Appendix 1. Visual analog scale (VAS) for pain assessment 135

Appendix 2 DIARY OF A VOLUNTEER. Template 136

Appendix 3 Vaccination Adverse Events Rating Scale[9] 139

Annex 4 Body mass index 144

Abbreviations

Abbreviation	Definition
BP	Blood pressure
ALT	Alanine aminotransferase
AST	Aspartate aminotransferase
AT 2	Alveolar cells type II
ATC	Anatomical therapeutic chemical classification
APTT	Activated partial thrombin time
EVD	Ebola virus disease
MERS	Middle East Respiratory Syndrome
HIV	Human immunodeficiency virus
WMA	World Medical Association
WHO	World Health Organization
DTH	Delayed type hypersensitivity
CI	Confidence interval
EAEU	Eurasian Economic Union
HID	Human immunizing dose
ED	Experimental drug
BMI	Body mass index
INF	Interferon
ICA	Immunochromatographic Analysis
CT	Computed tomography
LEK	Local ethical committee
MIBP	Medical immunobiological preparation
ICD-10	International Classification of Diseases 10th revision
INR	International Normalized Ratio
NSAID	Non-steroidal anti-inflammatory drug
IDMC	Independent Data Monitoring Committee
IEC	Independent Ethics Committee
AE	Adverse event
ARF	Acute respiratory failure
ARVI	Acute respiratory viral infection
ARDS	Acute respiratory distress syndrome
PBSC	Peripheral blood stem cells
PCR	Polymerase chain reaction
RNA	Ribonucleic acid
PPE	Personal protective equipment
SAE	Serious adverse event
SOP	Standard Operating Procedure
AIDS	Acquired immunodeficiency syndrome
SARS	Severe Acute Respiratory Syndrome
RR	Respiratory rate
HR	Heart rate
EDTA	Ethylenediaminetetraacetic acid
EIDr	Equi-immunizing dose for rats
eIRC	Electronic individual registration card
ECG	Electrocardiography

ACE2	Angiotensin-converting enzyme type II (angiotensin-converting enzyme 2)
ADE	Antibody-dependent enhancement
COVID-19	Coronavirus disease 2019
CTCAE	Common Terminology Criteria for Adverse Events
FAS	Full analysis set
GCP	Good Clinical Practice
HBsAg	Hepatitis B surface antigen
HCV	Hepatitis C virus
ICH	International council for harmonisation of technical requirements for pharmaceuticals for human use
Ig	Immunoglobulin
MedDRA	Medical Dictionary for Regulatory Activities
MERS-CoV	Middle East Respiratory Syndrome-Related Coronavirus
PT	Preferred term
RBD	Receptor-binding domain
SAF	Safety Set
SARS-CoV	Coronavirus severe acute respiratory syndrome
SARS-CoV-2	Severe Acute Respiratory Syndrome Coronavirus 2
SD	Standard deviation
SOC	System organ class

Synopsis

**Study title:**	Double-blind, placebo-controlled study with an open dose selection period to assess the safety, tolerability, and immunogenicity of the drug "Gam-COVID-Vak M combined vector vaccine for the prevention of coronavirus infection caused by SARS-CoV-2 virus in adolescents"
**Study protocol title abbreviation**[1]	An open label study with an open dose selection period to assess the safety, tolerability and immunogenicity of the drug "Gam-COVID-Vac, a combined vector vaccine for the prevention of coronavirus infection caused by the SARS-CoV-2 virus", with the participation of Adolescent Volunteers<br>**OLSTAD**
**Name of the drug developer**	Federal Government Budgetary Institution "National Research Center for Epidemiology and Microbiology n.a. Honorary Academician N.F. Gamaleya" of the Ministry of Health of the Russian Federation
**Information about the experimental drug (ED)**	**Trade name:** Gam-COVID-Vak M combined vector vaccine for the prevention of coronavirus infection caused by SARS-CoV-2 virus<br>**Registration number:** n/a<br>**International non-proprietary or grouping name:** Vaccine for prevention of coronavirus infection (Coronavirus disease 2019, COVID-19)<br>**Dosage form:** solution for intramuscular injection<br>**Composition per 1 dose (0.5 ml):**<br>**Component I contains:**<br>*Active substance:* recombinant adenoviral particles of serotype 26 containing the S protein gene of Severe Acute Respiratory Syndrome Coronavirus 2 (SARS-CoV-2).<br>*Excipients:* Tris(hydroxymethyl)aminomethane – 1.21 mg, sodium chloride – 2.19 mg, sucrose – 25.0 mg, magnesium chloride hexahydrate – 102.0 µg, ethylenediaminetetraacetic acid (EDTA) disodium salt dihydrate – 19.0 µg, polysorbate 80–250 µg, ethanol 95% – 2.5 µl, water for injection – ≤0.5 ml.<br>**Dose:**<br>1.0×10^{10} of component I dose and 1.0×10^{10} of component II dose (hereinafter referred to as 1/10 of the immunizing dose)<br>2.0×10^{10} of component I dose and 2.0×10^{10} of component II dose (hereinafter referred to as 1/5 of the immunizing dose)

[1] Abbreviated name retained from the first version of the protocol

	**Component II contains:**<br>*Active substance:* recombinant adenoviral particles of serotype 5 containing the S protein gene of SARS-CoV-2.<br>*Excipients:* Tris(hydroxymethyl)aminomethane – 1.21 mg, sodium chloride – 2.19 mg, sucrose – 25.0 mg, magnesium chloride hexahydrate – 102.0 µg, ethylenediaminetetraacetic acid (EDTA) disodium salt dihydrate – 19.0 µg, polysorbate 80–250 µg, ethanol 95% – 2.5 µl, water for injection – ≤0.5 ml.<br><br>**Dose:**<br>1.0 $\times 10^{10}$ of component I dose and 1.0 $\times 10^{10}$ of component II dose (hereinafter referred to as 1/10 of the immunizing dose)<br>2.0 $\times 10^{10}$ of component I dose and 2,0 $\times 10^{10}$ of component II dose (hereinafter referred to as 1/5 of the immunizing dose)<br><br>*Reference drug*: no<br><br>**Description:**<br>Component I. It is a frozen solution, a dense hardened whitish mass. After thawing, it becomes a homogeneous colorless or slightly opalescent yellowish solution.<br>Component II. It is a frozen solution, a dense hardened whitish mass. After thawing, it becomes a homogeneous colorless or slightly opalescent yellowish solution.<br>**Characteristics:** The vaccine was obtained using the biotechnology method that does not use the SARS-CoV-2 virus pathogenic for humans. The drug consists of two components: component I and component II. Component I includes a recombinant adenoviral vector based on human adenovirus serotype 26 carrying the SARS-CoV-2 virus S protein gene, component II includes a vector based on human adenovirus serotype 5 carrying the SARS-CoV-2 virus S protein gene.<br><br>**Placebo**<br>**Composition per 1 dose (0.5 ml):**<br>**Component I contains**:<br>Tris(hydroxymethyl)aminomethane – 1.21 mg, sodium chloride – 2.19 mg, sucrose – 25.0 mg, magnesium chloride hexahydrate – 102.0 µg, ethylenediaminetetraacetic acid (EDTA) disodium salt dihydrate – 19.0 µg, polysorbate 80–250 µg, ethanol 95% – 2.5 µl, water for injection – ≤0.5 ml.**Component II contains**:<br>Tris(hydroxymethyl)aminomethane – 1.21 mg, sodium chloride – 2.19 mg, sucrose – 25.0 mg, magnesium chloride hexahydrate – 102.0 µg, ethylenediaminetetraacetic acid (EDTA) disodium salt dihydrate – 19.0 µg, polysorbate 80–250 µg, ethanol 95% – 2.5 µl, water for injection – ≤0.5 ml.

**Clinical study phase**	I-II-III
**Study period, number of reserach centers and volunteers**	The duration of participation of volunteers in the study will be at least 53 weeks, including 1 week of screening, 3 weeks of therapy and 49 weeks of follow-up. Pre-screening is possible.<br><br>Approximately 3,100 volunteers will be randomized to the study.<br><br>Enough participants will be screened to select at least 100 participants for Phase 1 and approximately 3,000 participants for Phase 2. A pre-screening procedure is included in order to form a database of volunteers for subsequent inclusion in the study.<br><br>The study will include volunteers of both genders aged 12–17 inclusive.<br><br>No more than 50 people will be included in each dosing group.<br><br>In general, the scheme of drug administration will be as follows:<br><br>group 1: of 50 volunteers –1/10 of the adult dose<br><br>Interim safety assessment on day 7 with transition to the second dosing group – 1/5 of the adult dose.<br><br>group 2: 50 volunteers – 1/5 of the adult dose<br><br>Based on the results of the assessment of safety and immunogenicity patameters on day 28 of the study, a report will be prepared, and the dose will be selected.<br><br>The results of the follow-up during the 28-day follow-up period, with the processing of data from all surveys, will be presented in the report, which will be subsequently submitted to the regulatory authorities. The report will be drawn up in accordance with the requirements of order 200n, section 1. P 9 to the extent of the data available at the date of its writing.<br>The results of the 365-day follow-up will be presented in the Final Report.
**Purpose and objectives of the study**	**Study purpose**:<br>To assess the immune response and safety, tolerability, and immunogenicity profile of the COVID-19 vaccine in adolescents aged 12–17.<br><br>**Study objectives**<br>1. *Study the safety of the drug in healthy volunteers aged 12–17 inclusive by sequential escalation of 2 doses: 1/10 and 1/5 of the immunizing dose.*<br>During the entire follow-up period, the effect of the drug on vital (systolic and diastolic blood pressure, heart rate, and body temperature) and laboratory parameters in healthy volunteers will be studied, as well as on the presence of general and local post-vaccination reactions compared with background values before drug administration.

	A planned transition to the next dose is allowed based on the interim report on safety and tolerability on day 28 after immunization.<br><br>In case of severe general and local post-vaccination reactions or AE in the group, the study in the group is immediately stopped. The safe adjusted dose will be the dose used in the previous study group.<br>2. *Determine a safe dose for healthy volunteers aged 12–17 and proceed to the second stage of the study.*<br>3. *Describe the intensity of the cellular immune response when using “Gam-COVID-Vac M Combined vector vaccine for the prevention of coronavirus infection caused by the SARS-CoV-2 virus (on the day of drug administration before receiving the first dose of the vaccine and 28 days later, based on the concentration of interferon gamma in T-lymphocyte culture after re-stimulation with SARS-CoV-2 glycoprotein).*<br>4. *Describe the state of the antigen-specific cellular immune response when using the “Gam-COVID-Vac M combined vector vaccine for the prevention of coronavirus infection caused by the SARS-CoV-2 virus”.*<br>5. *Study the immunogenicity of the selected dose of the drug in healthy volunteers:*<br>– *study of immunogenicity based on the level of anti-RBD antibodies will be carried out on days 21, 28, 42, 90, and 180 after immunization with the first component by determining the titer of specific antibodies by ELISA compared to background values (day 1),*<br>– *study of virus neutralizing activity will be carried out on days 21, 28, 42, 90, and 180 after immunization with the first component by determining the titer of specific antibodies by ELISA compared to background values (day 1).*<br>6. *Assess the immunogenicity of “Gam-COVID-Vac M Combined vector vaccine for the prevention of coronavirus infection caused by the SARS-CoV-2 virus”, in comparison with placebo, based on the geometric mean of the titer of SARS-CoV-2 glycoprotein-specific antibodies (on dosing day before the first dose of ED/placebo and 28 days after).*<br>7. *Evaluate the safety of using “Gam-COVID-Vak M combined vector vaccine for the prevention of coronavirus infection caused by the SARS-CoV-2 virus” compared to placebo use after receiving the first dose of ED/placebo.*<br>8. *Study of the safety profile of the drug in healthy volunteers aged 12–17 years up to 365 days after vaccination.*<br>9. *Compare immunogenicity scores in adolescents with safety and immunogenicity scores in young people aged 18–21 years old who are participants of the clinical trial No. 04-Gam-COVID-Wak-2020 “Randomized, double-blind, placebo-controlled, multicenter clinical study of the efficacy, immunogenicity, and safety of combined Gam-COVID-Vac vector vaccine in parallel groups in preventing coronavirus infection caused by the SARS-CoV-2 virus” as the closest to the study group according to the selection criteria.*

	10. Assess the vaccine’s protective effect between days 28 and 365 of the study (describe cases of COVID-19, severe COVID-19, and death).
**Study design**	Double-blind, placebo-controlled, randomized clinical trial to evaluate the immunogenicity and safety of the “Gam-COVID-Vac M combined intramuscular vector vaccine for the prevention of coronavirus infection caused by the SARS-CoV-2 virus” in study subjects aged 12–17 years inclusive in preventing coronavirus infection caused by the SARS-CoV-2 virus.<br>The study will include healthy volunteers aged 12–17 inclusive. Age stratification will be carried out into two age strata: 12–14 and 15–17 years old.<br>Volunteers will be included in at least two clinical centers. Groups will be divided into subgroups to ensure the safety of volunteers for the period of hospitalization during the ongoing pandemic.<br>**Stage 1 (Phase I-II)**: 100 volunteers will be included in two dosing groups (50 in each group), a sufficient number of volunteers should be screened (no more than 7 days before inclusion in the study). To ensure the required number of volunteers who have completed the screening procedures (pre-screening is possible).<br>The conditions for the study are selection of doses (administration of the drug and follow-up for 48 hours) at the hospital, the rest of the visits are on an outpatient basis.<br>The study is planned to be started at a dose of 1/10 of the full dose for adults. Volunteers will be hospitalized the day before vaccination. It is allowed to conduct screening procedures the day before vaccination at the hospital, subject to prior approval of the volunteer based on the results of prescreening. The first dose will be administered on day 1. After that, the volunteer will be observed at the hospital for 48 hours. On days 3 and 7, a telephone consultation will be held.<br><br>In case of a favorable safety profile after 7 days post full dose, the Principal Investigators present a consolidated decision to escalate the dose to 1/5 of the adult dose for IDMC.<br><br>**The criteria for switching to a higher dose are:**<br>– absence of SAE, the relationship of which with vaccination is defined as “probable” or “certain”<br>– he absence of potentially life-threatening AE (class 4, according to the classification presented in Appendix 3), the relationship of which with vaccination is defined as “probable” or “certain, with the exception for allergic reactions

	– the absence of severe AE (class 3, according to the classification presented in Appendix 3) in more than half of the volunteers after the first administration of the vaccine, the relationship of which with vaccination is defined as "probable" or "certain".<br><br>If 1/10 of the dose is deemed unsafe, the study will be stopped and no second dose will be administered.<br><br>If 1/5 of the dose is deemed unsafe, the second dose will not be administered.<br><br>If both doses show an acceptable safety profile, the choice of dose for continuing the study will be made based on the results of immunogenicity analysis.<br><br><u>An interim report on the 1st stage of the study on day 28 will be submitted to the Russian Ministry of Health.</u><br><br>The transition to the next stage is planned based on the report on the safety and tolerability of the ED on the 28th day after administration of the first component. The adjusted dose will be determined based on day 28 immunogenicity, which should be consistent with that in adults after receiving a full dose of ED. Intramuscular administration of the vaccine will be carried out at vaccination visits No. 2 and No. 4 (Day 1 and Day 21±2) in a hospital setting. Hospitalization is planned on the eve of the drug administration, after vaccination; adolescents will be under medical supervision for 2 days, and discharged on the third day.<br>Subsequent follow-up visits No. 3, 5, 6, 7, and 8 will be held on days 14±1, 28±2, 42±4, 90±14, and 180±14, respectively. Telephone contacts with the researcher are also included in the study.<br><br>**Stage 1 (Phase III)**: 3,000 volunteers who will be randomized into two groups: active drug and placebo at a ratio of 4:1. Volunteers will receive the drug at the dose selected at the first stage. The study is carried out on an outpatient basis.<br>Subjects will be randomized into two age strata: 12–14 and 15–17 years old.<br>IM vaccine/placebo will be administered at vaccination visits #2 and #3 (Day 1 and Day 21±2) on an outpatient basis. After vaccination, TMK will be carried out within 2 days.<br>On day 28, deblinding will be performed.<br>Volunteers who received the vaccine will be followed up according to the plan of follow-up visits 5 (42±4), 6 (90±7), and 7 (180±14); and remote visits (telephone contact) on days 270±14 and 365±14.<br>Volunteers who received a placebo will receive the vaccine and start over with the study plan.

	Volunteers who donate blood for immunogenicity assessment and volunteers who receiving placebo will receive monetary compensation.<br><br>Historical control data (data from phase III clinical trials of efficacy, safety, and immunogenicity obtained by their evaluation in a sample of adult volunteers aged 18–21 years) will also be used as a comparison group;<br><br>The duration of participation in the study for one subject will be 365±14 days after the first dose of vaccine, during which each subject will undergo a screening visit, 2 hospitalizations for drug administration, 5 face-to-face visits to the investigator and telephone contacts with the investigator.<br><br>The duration of participation in the study for one subject will be 365±14 days after the first dose of the vaccine.<br><br>Research subjects' data will be collected using electronic forms of individual registration cards, as well as using electronic questionnaires (diaries) filled out by research subjects.
**Safety assessment**	The duration of volunteer participation in the study will be 53 weeks, including 1 week of screening, 3 weeks of therapy and 49 weeks of follow-up. For placebo volunteers, the study will be extended by 28 days to unblind and the period of unblind procedures by a total of no more than 2 months.<br><br>Sfety assessment will be based on registration of AE for 42 days and SAE during the entire follow-up period.<br><br>In order to prevent the development of post-vaccination reactions and complications, volunteers are screened to determine the initial indicators of the state of the body. In addition, volunteers will undergo a medical examination. All baseline indicators (examinations before drug administration) are added to the electronic individual registration card (eIRC) of volunteers.<br><br>Throughout the vaccination period and during the follow-up period, safety information will be collected using the following parameters:<br>• development, severity, and relationship between administration of the two components of the drug Gam-COVID-Vac M combined vector vaccine for the prevention of coronavirus infection caused by the SARS-CoV-2 virus and all SAE during the entire study;<br>• development, severity, and relationship between administration of the two components of the drug Gam-COVID-Vak M combined vector vaccine for the prevention of coronavirus infection caused by the SARS-CoV-2 virus and all AE during 42 days of the study;<br>• the frequency and severity of local signs and symptoms, the relationship with the drug use with general signs and symptoms after drug administration;

	• physical, laboratory, and instrumental study/analysis data;<br>• reactogenic properties are assessed in terms of local (injection site area) and general (systemic reactions of the body to drug administration) reactions.<br>Local reactions are evaluated based on the following parameters:<br>• hyperemia at the injection site;<br>• edema (infiltration);<br>• pain<br>• increase in regional lymph nodes;<br>• rash;<br>• itching.<br>Common post-vaccination reactions include: fever, physical discomfort, headache, dizziness, decreased appetite, insomnia, nausea, vomiting, dyspepsia, weakness, sweating, muscle and joint pain, abdominal pain, convulsions, etc. In addition, allergic reactions are possible.<br><br>Accounting for local and general reactions is carried out by the researcher based on body temperature measurement, examination, and questioning of the volunteer, conducted in accordance with the schedule of visits and research procedures.<br><br>Changes in laboratory parameters (clinical and biochemical blood tests), changes in vital signs (blood pressure [BP], heart rate [HR], respiratory rate [RR], body temperature) will also be assessed.<br><br>When assessing, the investigator should keep in mind that there are no pathognomonic symptoms that would allow one to unambiguously consider each specific case of AE associated with vaccination. Such clinical symptoms as high fever, intoxication, neurological symptoms, and various types of allergic reactions, including the immediate type, may not be due to immunization but to a disease coincided with vaccination. Therefore, each case of the disease that developed in the post-vaccination period requires careful differential diagnosis of both infectious and non-infectious diseases using instrumental and laboratory research methods based on the disease clinical symptoms.<br><br>One of the criteria is the time of onset. As a rule, the symptoms of post-vaccination reactions develop no later than 48 hours, while the duration of fever for more than 72 hours or its occurrence after this time indicates a possible presence (onset) of infection.
**Assessment of immunogenicity**	Volunteers will be assessed for the following indicators of immunogenicity:<br>a) Indicators of the intensity of the humoral immune response:<br>• Geometric mean titer of IgG antibodies against SARS-CoV-2 spike protein;

	• Percentage of volunteers with specific IgG detected;<br>• Geometric mean titer of neutralizing antibodies against SARS-CoV-2;<br>• Percentage of volunteers with detected neutralizing antibodies.<br>b) Indicators of the intensity of the cellular immune response:<br>• An increase in the concentration of interferon gamma in the culture of T-lymphocytes after re-stimulation with a specific SARS-CoV-2 antigen (glycoprotein S or RBD);<br>• Level of proliferative activity of CD4+ and CD8+ cells after re-stimulation with a SARS-CoV-2 specific antigen (glycoprotein S or RBD).<br>Immunogenicity parameters can be compared with those of healthy volunteers participating in the Gam-COVID-Vak clinical trials at the same time.
**Inclusion criteria**	The study will include volunteers who meet all of the following criteria:<br>1. Written informed consent of the study subject and his parents/adoptive parents to participate in the study;<br>2. Boys and girls aged 12–17 inclusive;<br>3. Negative test result for HIV, hepatitis, and syphilis;<br>4. A negative result of the study for the presence of IgM and IgG antibodies against SARS CoV2 by enzyme immunoassay, and, if available, the result of a survey performed at medical organizations of the Moscow Health Department 7 days before inclusion in the study can be taken into account;<br>5. A negative test result for COVID-19, determined by PCR at the screening visit, and, if available, the result of a survey performed at medical organizations of the Moscow Health Department 7 days before inclusion in the study can be taken into account;<br>6. No history of COVID-19;<br>7. Absence of contact of the study subject with COVID-19 patients for at least 14 days prior to inclusion in the study (according to the study participant and parents/adoptive parents of the subject);<br>8. Consent to the use of effective methods of contraception during the entire period of participation in the study;<br>9. Negative pregnancy test based on the results of urine test at the screening visit (for all female participants in the study);<br>10. Negative test for the presence of narcotic and psychostimulant drugs in the urine at the screening visit;<br>11. Negative alcohol test at the screening visit;

	12. No history of severe post-vaccination reactions or post-vaccination complications after the use of immunobiological preparations;<br>13. Absence of acute infectious and/or respiratory diseases for at least 14 days before enrollment in the study.
**Non-inclusion criteria**	Volunteers cannot be included in the study if at least one of the following exclusion criteria is met:<br>1. Any vaccination/immunization received within 30 days prior to enrollment in the study.<br>2. Therapy with steroids (with the exception of hormonal contraceptives) and/or immunoglobulins or other blood products that did not end 30 days before enrollment in the study;<br>3. Immunosuppressive therapy and systemic corticosteroid therapy completed in less than 3 months prior to inclusion in the study.<br>4. History of acute coronary syndrome or stroke for the period less than one year prior to inclusion in the study<br>5. Any immunodeficiency (e.g., hereditary immunodeficiency, acquired immunodeficiency syndrome [AIDS], etc.).<br>6. Infectious diseases:<br>• a history of HIV (antibodies against HIV types 1 and 2), hepatitis (positive test for HBsAg or antibodies against HCV), active syphilis;<br>• Tuberculosis;<br>• Active infection (excluding onychomycosis) or any major episode of infection requiring intravenous antibiotic treatment within 4 weeks prior to screening or oral antibiotic treatment within 2 weeks prior to screening;<br>• A history of any other serious recurrent or chronic infection not listed above.<br>7. Major surgery within 4 weeks prior to screening.<br>8. Chronic autoimmune diseases or systemic collagenosis in history requiring immunosuppressive therapy.<br>9. Volunteers who have undergone organ transplantation, including bone marrow transplantation or peripheral blood stem cell (PBSC) transplantation, and who receives immunosuppressive therapy.<br>10. A history of splenectomy.

	11. Volunteers with a previous or concomitant history of neoplasms (ICD codes C00-D09).<br><br>12. Aggravated allergic history (history of anaphylactic shock, Quincke's edema and other life-threatening conditions), hypersensitivity or allergic reactions to the administration of immunobiological preparations, known allergic reactions to any of the components of the vaccine or a vaccine containing similar components, exacerbation of allergic diseases on the day of enrollment in the study.<br><br>13. Neutropenia (decrease in the absolute number of neutrophils less than 1000 cells / mm3), agranulocytosis, significant blood loss, severe anemia (hemoglobin concentration less than 80 g / l), thrombocytopenia (decrease in the absolute number of platelets less than 50,000 cells / mm3).<br><br>14. Anorexia, protein deficiency of any origin.<br><br>15. Volunteers with a BMI value in the range from -2 SD to +2 SD, according to age (Appendix 4).<br><br>16. Extensive tattoos at the sites of drug injection (deltoid muscle area), which do not allow assessing the local reaction to ED administration.<br><br>17. Chronic diseases of the cardiovascular, bronchopulmonary, neuroendocrine systems, as well as diseases of the gastrointestinal tract, liver, kidneys, muscle and connective tissue in the stage of exacerbation or decompensation.<br><br>18. The presence or suspicion of drug, alcohol or drug addiction and other mental disorders.<br><br>19. Medical conditions that, from the investigator's point of view, put the participant's health at risk if they participate in the study or potentially make it difficult to interpret the results of the study.<br><br>20. Family members of the staff of research centers directly involved in the conduct of the study and.<br><br>21. Participation in other clinical trials and use of other investigational drugs within 28 days prior to screening.<br><br>22. Planned vaccination against COVID-19 with any vaccine, both as part of other studies and as part of a civil circulation.<br><br>23. Female subjects during pregnancy or lactation<br><br>24. Inability to read Russian; inability or unwillingness to understand the essence of the study. Any other condition that limits the eligibility of obtaining informed consent or may affect a volunteer's ability to participate in the study will affect the volunteer's ability to participate in the study.

**Exclusion criteria**	Volunteers stop undergoing research procedures and remain under observation until the completion of the study in the following cases:<br><br>1. Refusal of the volunteer from further participation in the study.<br><br>2. Non-observance by the volunteer of the rules for participation in the study.<br><br>3. Occurrence of causes / occurrence during the study of situations that threaten the safety of the volunteer (for example, hypersensitivity reactions, etc.).<br><br>4. Volunteers selected to participate in the study in violation of the inclusion / non-inclusion criteria.<br><br>5. Vaccination with any vaccine for the prevention of coronavirus infection (COVID-19) both in clinical trials and in the framework of civil circulation<br><br>6. Positive pregnancy test in female subjects.<br><br>7. Occurrence in the course of the study of other reasons that prevent the conduct of the study according to the protocol.<br><br>In the event of an SAE or pregnancy, volunteers are excluded from the continuation of the vaccination program, but they continue to be observed in accordance with the rules described in Section 8 of the current protocol. In case of infection with SARS-CoV-2 (laboratory and/or instrumentally confirmed diagnosis), the volunteer is excluded from the vaccination program, but not from the study. Once disease is identified, there are no further standard study visits, but at least one ad hoc visit for SARS-CoV-2 infections (Section 4.2.5.8).
**Prohibited concomitant therapy**	No immunotropic drugs (immunosuppressants, immunomodulators), steroids (except for emergency corticosteroids, contraceptives), and/or immunoglobulin or other blood product therapy not completed 30 days prior to study entry are prohibited in this study. It is not allowed to use any vaccination 30 days before enrollment in the study and within 30 days after the second vaccination, then vaccination is allowed in accordance with the regional vaccination schedule.<br><br>Transfusion of blood and its components, plasmapheresis and donation are not allowed during the entire study. The use of immunoglobulins, preparations of monoclonal antibodies, interferons, colony-stimulating factors, growth factors is not allowed. If the need arises for therapy with the above drugs and procedures, the volunteer should be withdrawn from the study.<br><br>In the event that it becomes necessary to prescribe such drugs to a volunteer for health reasons, the volunteer will be withdrawn from the study with subsequent follow-up.

	It is not allowed to take phytopreparations and biologically active food supplements during the entire study.<br><br>Vaccination with any other COVID-19 vaccines is not permitted, including in other clinical trials.<br><br>All applicants from the placebo group can continue to participate in the study, after blinding they will receive the vaccine and will be under observation for 1 year after vaccine administration.
**Statistical methods**	Analysis of the research results will be carried out in accordance with the Statistical Analysis Plan. Descriptive statistics will be used to analyze safety, efficacy and immunogenicity data.<br><br>*Populations for analysis*<br><br>When conducting statistical analysis, it is planned to study three populations of subjects:<br>• All subjects included in the study (FAS, full analysis set). This population will be used to describe baseline characteristics, including demographics, anthropometrics, comorbidities, and all immunogenicity endpoints;<br>• An additional immunogenicity endpoint analysis will be performed on a population of subjects who completed study participation without major deviations from the study protocol and who have valid immunogenicity data at least at one time point after vaccine administration (PP population, Per Protocol);<br>• All subjects who received at least one component of the vaccine will be included in the analysis of safety parameters.<br>*Analysis of basic characteristics*<br>Subject distribution, demographics, and other baseline characteristics will be presented using descriptive statistics for all subjects included in the study (FAS population). Comparison of baseline scores between dose groups will be done using a t-test. For categorical data, a $\chi 2$-test or Fisher's exact test will be applied if the expected frequency in any of the cells is less than 5. Descriptive statistics will also be used to present data on comorbidities and concomitant therapy. Concomitant medications will be listed by their INN. Comorbidity data will be coded according to MedDRA.<br>*Analysis of safety parameters*<br><br>Adverse events (AE) will be coded according to the MedDRA Regulatory Glossary. For each AE/SAE, the absolute number will be given, as well as the proportion of subjects with the development of one or another AE. AE data will be tabulated by organ system and class (SOC) and presented with preferred terms (PT) according to MedDRA in relation to study therapy and severity.

To analyze the frequency of local reactions and abnormalities according to the physical examination, descriptive statistics methods will be used, indicating the absolute number of subjects in n/N format and the proportion of subjects in each category. For intergroup analysis, the χ2-test or Fisher's exact test will be used if the expected frequency in any of the cells is less than 5.

Quantitative safety data variables (clinical and biochemical blood tests, dynamics of vital signs) will be presented using descriptive statistics methods:

- Number of non-missing values (N);
- Minimum (Min);
- Maximum (Max);
- Arithmetic mean (M);
- Standard deviation (SD);
- 95% confidence interval for the mean (CI);
- Average (Median);
- Interquarter range (IQR).

Analysis of immunogenicity parameters

Data on the geometric mean titer of antibodies against SARS-CoV-2 IgG spike (S) protein will be analyzed using a paired t-test after their logarithmic transformation. The ratio of geometric mean titer before and after vaccination will be estimated separately for each dose group, as well as the 90% CI for this ratio. Similarly, the analysis of data on the geometric mean titer of neutralizing antibodies will be carried out. In addition, a mixed model can be used using antibody titer as dependent variable and dose group, evaluation visit, sex and age as fixed factors, and subject number as a random factor.

Descriptive statistics methods will be used to analyze data on the concentration of interferon gamma in the T-lymphocyte culture and the level of proliferative activity of CD4+ and CD8+ cells after restimulation with a specific SARS-CoV-2 antigen (glycoprotein S or RBD). The data will be presented by dose groups and assessment points. Intergroup comparison will be carried out using a t-test or Mann-Whitney test (depending on the type of data distribution). Within-group comparisons at each evaluation visit to baseline will be performed using a paired t-test or Wilcoxon test (depending on the type of data distribution). To assess the normality of the distribution, the Shapiro-Wilk test will be used.

A χ2-square test will be used to compare the proportion of subjects with seroconversion (titer increase ≥ 4 times from baseline) for anti-SARS-CoV-2 IgG spike (S) protein antibodies and for neutralizing antibodies. Additionally, to account for various covariates, the construction of a logistic regression model with an estimate of the odds ratio (OR), as well as 95% CI for OR for each of the factors of the model, can be used.
Safety and immunogenicity analyzes will be conducted separately for the 12–14 and 15-17 age cohorts.

Immunogenicity indicators, if necessary, can be compared with the indicators of healthy volunteers in the Gam-COVID-Vak clinical trials at the same time.

Rationale for sample size

The main purpose of the study is to evaluate immunogenicity and safety based on the development of adverse events. At the first stage of the study, it is planned to select a dose that will have a good safety profile. However, injection site reactions and systemic reactions of mild to moderate severity are expected in this study, AE that prevent further study of the vaccine or severe AE are not expected. The calculation showed that vaccination of 50 subjects in each dose group will detect with a 90% probability at least one AE (reaction at the injection site or systemic reaction) with an expected frequency of at least 5%. At the same time, in total, in two dose groups (100 people), the probability of detecting at least 1 AE will be 90% with an expected frequency of AE of at least 2.5%:

Probability of seeing at least 1 AE based on the expected frequency of AE

**Expected frequency of AE**	**Probability of observing at least 1 AE in N volunteers**						
	**N=50**	**N=100**	**N=200**	**N=250**	**N=350**	**N=2400**	**N=3000**
**0,05%**	2%	5%	10%	12%	16%	70%	78%
**0,1%**	5%	10%	18%	22%	30%	91%	95%
**0,5%**	22%	39%	63%	71%	83%	>99%	>99%
**1%**	39%	63%	87%	92%	97%	>99%	>99%
**1,5%**	53%	78%	95%	98%	99%	>99%	>99%
**2,5%**	72%	92%	99%	>99%	>99%	>99%	>99%
**5%**	92%	99%	>99%	>99%	>99%	>99%	>99%
**10%**	99%	>99%	>99%	>99%	>99%	>99%	>99%

At the second stage of the study, one of the main tasks is to evaluate the immunogenicity parameters for the selected vaccine dose. Therefore, in the second step, it is planned to include a sufficient number of subjects who will donate blood to estimate the proportion of subjects with seroconversion with an accuracy (90% confidence interval width) of at least 0.05, with an expected seroconversion rate of at least 0.95.

At least 500 people (400 vaccine and 100 placebo) will be included in the immunogenicity study for a statistically significant assessment of immunogenicity:

	*N = 3.8416 * X * (1-X) / 0.0025*<br>% | 95 | 90 | 85 | 80 | 75 | 70 | 65 | 60 | 55 | 50<br>number | 73 | 139 | 196 | 246 | 289 | 323 | 350 | 369 | 381 | 385
**Blinding, Randomization**	Blinding and randomization into treatment groups in the first stage of the design of this study is not provided.<br><br>Groups will be formed as volunteers are included<br><br>The second stage was a double-blind, placebo-controlled study stratified by age. Blinding was conducted after 28 days.

1 General information

1.1 Protocol name, protocol identification number and date:

Name: Double-blind, placebo-controlled study with an open dose selection period to assess the safety, tolerability, and immunogenicity of the drug "Gam-COVID-Vak M combined vector vaccine for the prevention of coronavirus infection caused by SARS-CoV-2 virus" in adolescents

Protocol identification number: 07-Gam-COVID-Vak-2021
Version: 3.0
Date: 23.07.2021

1.2 Administrative structure of the study

**Authorized person for pharmacovigilance**	Chernukha Marina Yurievna<br>*MD, responsible for pharmacological safety*<br>Bakhareva Alla Vladimirovna<br><br>*responsible for pharmacological safety*	Legal address/actual address:Russia, 123098, Moscow,18 Gamaleya St.<br>Tel: 8 (499) 193-30-01<br>Fax: 8 (499) 193-61-83<br>E-mail: info@gamaleya.org
**Drug Development Team (Scientific Experts)**	Logunov Denis Yurievich<br>*Deputy Director for Science, Doctor of Biological Sciences, Corresponding Member of the Russian Academy of Sciences*<br><br>Dolzhikova Inna Vadimovna<br>*Head of the Laboratory of the State Collection of Viruses, PhD of Biological Sciences*	Legal address/actual address:Russia, 123098, Moscow,18 Gamaleya St.<br>Tel: 8 (499) 193-30-01<br>Fax: 8 (499) 193-61-83<br>E-mail: info@gamaleya.org
**Expert from the organization organizing the clinical trial**	*Solving medical issues, safety analysis*<br><br>Ordzhonikidze Maria Konstantinovna<br><br>*Organizational and methodological issues*<br>Lubenets Nadezhda Leonidovna	Legal address/actual address: Russia, 123098,Moscow,18 Gamaleya St.<br>Tel: 8 (499) 193-30-01<br>Fax: 8 (499) 193-61-83<br><br>E-mail: info@gamaleya.org
		State budgetary health care institution of the city of Moscow Morozov Children's City Clinical Hospital of the Department of Health of the City of Moscow", 119049, Moscow, 4th Dobryninsky lane, 1/9

**Person responsible for making medical decisions in the Research Center**	Vlasova Anna Viktorovna	-

	Borzakova Svetlana Nikolaevna	State budgetary health care institution of the city of Moscow "Children's City Clinical Hospital named after Z.A Bashlyaeva of the Department of Healtl of the City of Moscow", 125373, Moscow, Panfilovtsev st., 28
**Coordinating Investigator,**<br><br>**Head of Expert Group Coordinating Investigator**	Zyryanov Sergey Kensarinovich<br>*MD, Professor, Deputy Chief Physician for the Therapy of GKB No24, Head of the Department of General and Clinical Pharmacology of the Peoples' Friendship University of Russia.*<br>Osmanov Ismail Magomedtagirovich<br>*Chief Freelance Children's Specialist, Chief Physician of GBUZ “Children's City Clinical Hospital. BEHIND. Bashlyaeva DZM, Doctor of Medical Sciences”, Professor*	Legal address/actual address: Moscow, Pistsovaya st., 10,<br>125373, Moscow, st. Geroev Panfilovtsev, 28
**Names and addresses of clinical laboratories participating in the clinical trial**	**Efficency assessment**	
	Laboratory of Cell Microbiology at the Federal Government Budgetary Institution “N.F. Gamaleya National Research Center for Epidemiology and Microbiology” of the Ministry of Health of the Russian Federation	Legal address/actual address:Russia, 123098, Moscow,18 Gamaleya St.<br>Tel: 8 (499) 193-30-01<br>Fax: 8 (499) 193-61-83<br>E-mail: info@gamaleya.org
	**Safety assessment**	
	Safety Laboratory Research: Laboratory of the Research Center	Fax: 8 (499) 193-61-83
**Independent Data Monitoring Committee**	Naigovzina Nelli Borisovna Chairman of the IDMC Head of the Department of Public Health and Healthcare of the Moscow State Medical University Evdokimova of the Ministry of Health of Russia, President of Evogen LLC, Doctor of Medical Sciences, Prof., Honored Doctor of the Russian Federation<br>The list of members of the IDMC is given in section 14	

2 Rationale for the study

2.1 Experimental drug

Registration number: n/a
Trade name: Gam-COVID-Vak M combined vector vaccine for the prevention of coronavirus infection caused by SARS-CoV-2 virus
International non-proprietary or grouping or chemical name: vaccine for the prevention of novel coronavirus infection (COVID-19).
Drug form: solution for intramuscular injection.
Composition per 1 dose (0.5 ml):
Component I contains:
Active ingredient: recombinant adenoviral particles of serotype 26 containing the S protein gene of the SARS-CoV-2 virus, in the amount of:
Dose:
1.0 $\times 10^{10}$ of component I dose and 1.0 $\times 10^{10}$ of component II dose (hereinafter referred to as 1/10 of the immunizing dose)
2.0 $\times 10^{10}$ of component I dose and 2.0 ×1010 of component II dose (hereinafter referred to as 1/5 of the immunizing dose)
Excipients: Tris(hydroxymethyl)aminomethane - 1.21 mg, sodium chloride - 2.19 mg, sucrose - 25.0 mg, magnesium chloride hexahydrate - 102.0 μg, ethylenediaminetetraacetic acid (EDTA) disodium salt dihydrate - 19.0 μg, polysorbate 80–250 μg, ethanol 95% – 2.5 μl, water for injection – ≤0.5 ml.
Component I contains:
Active ingredient: recombinant adenoviral particles of serotype 5 containing the S protein gene of the SARS-CoV-2 virus, in the amount of:
Dose:
1.0 $\times 10^{10}$ of component I dose and 1.0 $\times 10^{10}$ of component II dose (hereinafter referred to as 1/10 of the immunizing dose)
2.0 $\times 10^{10}$ of component I dose and 2.0 ×1010 of component II dose (hereinafter referred to as 1/5 of the immunizing dose)
Excipients: Tris(hydroxymethyl)aminomethane - 1.21 mg, sodium chloride - 2.19 mg, sucrose - 25.0 mg, magnesium chloride hexahydrate - 102.0 μg, ethylenediaminetetraacetic acid (EDTA) disodium salt dihydrate - 19.0 μg, polysorbate 80–250 μg, ethanol 95% – 2.5 μl, water for injection – ≤0.5 ml.
Description:
Frozen preparation
Component I. Frozen solution. It is a dense hardened whitish mass. After thawing: homogeneous colorless or slightly opalescent solution with a yellowish tinge.
Component II. The solution is frozen. It is a dense hardened whitish mass. After thawing: homogeneous colorless or slightly opalescent solution with a yellowish tinge.
liquid drug
Component I. Homogeneous colorless or slightly opalescent solution with a yellowish tint.
Component II. Homogeneous colorless or slightly opalescent solution with a yellowish tinge.
Characteristic
The vaccine was obtained in a biotechnological way, which does not use the SARS-CoV-2 virus pathogenic for humans. The drug consists of two components: component I and component II.

Component I includes a recombinant adenoviral vector based on human adenovirus serotype 26 carrying the SARS-CoV-2 virus S-protein gene, component II includes a vector based on human adenovirus serotype 5 carrying the SARS-CoV-2 virus S protein gene .

Pharmacotherapeutic group: medical immunobiological preparation (MIBP)-vaccine.

ATX code: J07B

Pharmacological properties

The vaccine induces the formation of humoral and cellular immunity against coronavirus infection caused by the SARS-CoV-2 virus.

Immunological efficacy

The immunological properties and safety of the vaccine have been studied in various clinical studies in adult volunteers of both sexes over the age of 18 years.

Interim analysis of immunogenicity showed that the vaccine generates an immune response in volunteers. In the study of the humoral immune response, the sera of volunteers were analyzed for the presence of antibodies specific to the receptor-binding domain of the glycoprotein S of the SARS-CoV-2 virus on day 42 from the start of vaccination: in the vaccinated group, the geometric mean value of the antibody titer was 8996, the seroconversion level was 98, 25. When comparing the level of RBD-specific antibodies between age strata, a statistically significant difference was shown for the group of 18–30 years relative to other age groups: the geometric mean value of the antibody titer was 18102–22067, the seroconversion rate was 100%.

Antibody levels did not differ significantly between men and women. On day 42 from the start of vaccination, the geometric mean titer of neutralizing antibodies in immunized volunteers was 44.47, the seroconversion rate was 95.83%.

There was no statistically significant difference in volunteers of different sex and age. Immunization with Gam-COVID-Vac forms intense antigen-specific cellular anti-infective immunity in almost all examined volunteers (formation of antigen-specific cells of both populations of T lymphocytes: T helper (CD4+) and T killer (CD8+) and a significant increase in IFNγ secretion) .

The protective antibody titer is currently unknown. The duration of protection is unknown.

Clinical studies to study the epidemiological efficacy are currently ongoing. According to the interim analysis, the efficiency is more than 91%.

Indications for use:

Prevention of novel coronavirus infection (COVID-19) in adults over 18 years of age.

Contraindications:

- Hypersensitivity to any component of the vaccine or a vaccine containing similar components;
- Severe allergic reactions in history;
- Acute infectious and non-infectious diseases, exacerbation of chronic diseases - vaccination is carried out 2–4 weeks after recovery or remission. For non-severe acute respiratory viral infections, acute infectious diseases of the gastrointestinal tract, vaccination is carried out after the temperature normalizes;
- Pregnancy and breastfeeding period;
- Age up to 18 years (due to the lack of data on efficacy and safety)

Contraindications for administration of component II: severe post-vaccination complications (anaphylactic shock, severe generalized allergic reactions, convulsions, temperature above 40 ° C, etc.) to administration of component I of the vaccine.

With caution: use the vaccine with caution in chronic diseases of the liver and kidneys, endocrine diseases (severe disorders of the thyroid gland and diabetes mellitus in the stage of decompensation), severe diseases of the hematopoietic system, epilepsy and other diseases of the central nervous system, acute coronary syndrome and acute cerebrovascular accident, myocarditis, endocarditis, pericarditis.

Due to lack of information, vaccination may pose a risk for the following patient groups:

- with autoimmune diseases (stimulation of the immune system can lead to an exacerbation of the disease, especially care should be taken in patients with autoimmune pathology, which tends to develop severe and life-threatening conditions)
- with malignant neoplasms

The decision to vaccinate should be based on an assessment of the benefit/risk ratio in each specific situation.

Use during pregnancy and during breastfeeding

There is no clinical experience with Gam-COVID-Vac during pregnancy. In the course of the study of reproductive toxicity in animals, no negative effects on the course of pregnancy, embryofetal development (on females) and prenatal development of offspring have been established. In this regard, Gam-COVID-Vak should be used during pregnancy only in cases where the expected benefit to the mother outweighs the potential risk to the fetus. There are no clinical data on the use of Gam-COVID-Vac in breastfeeding women. A risk to breastfed newborns/infants cannot be excluded. In this regard, the use of the drug Gam-COVID-Vac during breastfeeding is contraindicated.

Dosage and administration

The vaccine is for intramuscular administration only. Intravenous administration of the drug is strictly prohibited. The vaccine is injected into the deltoid muscle (upper third of the outer surface of the shoulder). If it is impossible to inject into the deltoid muscle, the drug is injected into the vastus lateral muscle of the thigh. Vaccination is carried out in two stages: first with component I, then after 3 weeks with component II.

The drug is administered intramuscularly: first component I at a dose of 0.5 ml, then after 3 weeks component II at a dose of 0.5 ml.

Frozen preparation

Before vaccination, the vial or ampoule with component I or II is removed from the freezer and kept at room temperature until completely thawed. Residual ice inclusions are not allowed! Wipe the outside of the vial or ampoule with an alcohol wipe to remove moisture. Gently mix the contents by shaking. Do not shake the vial or ampoule!

Remove the protective plastic cover from the vial and treat the rubber stopper with an alcohol wipe. The ampoule is opened along the ring or break point.

Using a disposable syringe with a needle, a dose of 0.5 ml is taken for administration to the patient. If subsequent injections are postponed for any reason, it is allowed to store the opened vial or ampoule - no more than 2 hours at room temperature.

Repeated freezing of the vial or ampoule with the solution is not allowed!

Attention! The drug (liquid and / or frozen) is unsuitable for use if the closure system is defective and / or the labeling of the vial or ampoule is broken, if the physical properties change (turbidity, staining) and/or the expiration date has passed.

▼ Information for medical workers performing vaccination with a medicinal product: this medicinal product is registered according to a special registration procedure, in connection with which it is necessary to notify the Federal Service for Surveillance in Healthcare of each fact of using a medicinal product by entering information into the appropriate section of the USHIS information system

Side effects

Adverse events specific to the use of the vaccine, identified in clinical trials, as well as studies of other vaccines based on a similar technological platform, are predominantly mild or moderate in severity, may develop on the first or second day after vaccination and resolve within 3 subsequent days. More often than others, short-term general (a short flu-like syndrome characterized by chills, fever, arthralgia, myalgia, asthenia, general malaise, headache) and local (soreness at the injection site, hyperemia, swelling) reactions may develop. The appointment of non-steroidal anti-inflammatory drugs (NSAIDs) is recommended when the temperature rises after vaccination.

Less common are nausea, dyspepsia, loss of appetite, and sometimes an increase in regional lymph nodes. Some patients may develop allergic reactions, a short-term increase in the level of hepatic transaminases, creatinine and creatine phosphokinase in the blood serum.

As part of the clinical studies of the safety, tolerability and immunogenicity of the drug Gam-COVID-Vak, the following AE were registered after vaccination:

“General disorders and reactions at the injection site”: hyperthermia, pain, swelling, itching at the vaccination site, asthenia, pain, malaise, pyrexia, increased skin temperature at the vaccination site, decreased appetite. The frequency of development is very often and often.

“Respiratory, thoracic and mediastinal disorders”: pain in the oropharynx, nasal congestion, sore throat, rhinorrhea. The frequency of development is often.

“Nervous system disorders”: headache - often, dizziness, fainting – rarely.

“Gastrointestinal disorders”: nausea, vomiting, dyspepsia - often.

“Laboratory and instrumental data”: multidirectional deviations of immunological status indicators: an increase in the number of T-lymphocytes, an increase in the percentage of lymphocytes, a decrease in the number of natural killer cells, an increase in the number of CD4 lymphocytes, a decrease in the number of CD4-lymphocytes, an increase in the number of B-lymphocytes, a decrease number of B-lymphocytes, increase in the number of natural killer cells, increase in the number of CD8 lymphocytes, increase in the level of IgE in the blood, increase in the ratio of CD4 / CD8, decrease in the ratio of CD4 / CD8, increase in IgA in the blood, decrease in the percentage of CD8 lymphocytes. Deviations in the general blood test: an increase in the percentage of lymphocytes, a decrease in hematocrit, an increase in the number of lymphocytes, an increase in the erythrocyte sedimentation rate, an increase in the number of leukocytes, an increase in the number of monocytes, an increase in the number of platelets, a decrease in the number of neutrophils, a decrease in the number of platelets. Abnormal urinalysis: erythrocytes in the urine.

Most AE ended in recovery without sequelae. Laboratory abnormalities had no clinical significance (did not require additional diagnostic procedures and therapy).

Overdose

No cases of overdose have been reported.

Given that the distribution of the medicinal product is allowed only for medical institutions, and the vaccination itself is carried out only by qualified medical personnel, the risk of overdose is extremely low.

However, it can be assumed that in case of accidental overdose, the development of the above toxic and toxic-allergic reactions to a more severe degree is possible. There are no specific antidotes for the drug.

Therapeutic measures in this case will include symptomatic therapy in accordance with the indications (antipyretic / NSAIDs and desensitizing agents), corticosteroids - parenterally for severe toxic-allergic syndrome). The regimen of prescribing drugs should be selected according to the recommendations for the use and dosages of this drug.

Interaction with other drugs

Not studied.

Special instructions

Patients receiving immunosuppressive therapy and patients with immunodeficiency may not develop a sufficient immune response. Therefore, it is recommended to postpone the use of drugs that depress the function of the immune system for at least 1 month before and after vaccination due to the risk of reduced immunogenicity.

Influence on the ability to drive vehicles, mechanisms

Studies on the effect of the vaccine on the ability to drive vehicles and potentially dangerous mechanisms have not been conducted.

2.2 Placebo

Composition per 1 dose (0.5 ml):

Component I contains:

Tris(hydroxymethyl)aminomethane - 1.21 mg, sodium chloride - 2.19 mg, sucrose - 25.0 mg, magnesium chloride hexahydrate - 102.0 μg, ethylenediaminetetraacetic acid (EDTA) disodium salt dihydrate - 19.0 μg, polysorbate 80–250 μg, ethanol 95% – 2.5 μl, water for injection – ≤0.5 ml.

Component II contains:

Tris(hydroxymethyl)aminomethane - 1.21 mg, sodium chloride - 2.19 mg, sucrose - 25.0 mg, magnesium chloride hexahydrate - 102.0 μg, ethylenediaminetetraacetic acid (EDTA) disodium salt dihydrate - 19.0 μg, polysorbate 80–250 μg, ethanol 95% – 2.5 μl, water for injection – ≤0.5 ml.

The placebo will not differ in appearance from the study drug (vaccine).

2.3 Summary of non-clinical findings

Previously developed drugs for Ebola virus disease (EVD), influenza and Middle East respiratory syndrome (MERS) have been studied in preclinical studies and have shown a favorable safety profile. The effect of drugs on the organism of animals was studied in a wide range of doses, including doses exceeding therapeutic doses by 1000 times. The death of animals was not registered, there was no development of pronounced toxic phenomena, deviations of laboratory and physiological parameters were not clinically significant, the absence of significant pathological processes in the body of animals and local irritant effects was shown, allergenic and immunotoxic effects were not shown, reproductive toxicity was not detected (toxic effects on reproductive

function of males and females, embryo- and fetotoxic effects recorded in the prenatal period, anomalies and delays in the development of embryos, embryotoxic and teratogenic effects in the antenatal period were not detected). The safety profile of various vaccines based on recombinant adenoviruses developed at the N.N. N.F. Gamalei” of the Ministry of Health of Russia is comparable and does not differ significantly in the developed vaccines among themselves and according to the literature for similar drugs.

Previously developed drugs for EVD, influenza, and MERS have been studied in preclinical studies and have shown a favorable safety profile. The effect of drugs on the organism of animals was studied in a wide range of doses, including those exceeding therapeutic doses by 1,000 times. The death of animals was not registered, there was no development of pronounced toxic phenomena, deviations of laboratory and physiological parameters were not clinically significant, the absence of significant pathological processes in the body of animals and local irritant effects was shown, allergenic and immunotoxic effects were not shown, reproductive toxicity was not detected (toxic effects on reproductive function of males and females, embryo- and fetotoxic effects recorded in the prenatal period, anomalies and delays in the development of embryos, embryotoxic and teratogenic effects in the antenatal period were not detected). The safety profile of various vaccines based on recombinant adenoviruses developed at the N.N. N.F. Gamalei” of the Ministry of Health of Russia is comparable and does not differ significantly in the developed vaccines among themselves and according to the literature for similar drugs.

Preclinical safety studies (toxicity)

Studies of the toxicity of the vaccine for the prevention of SARS-CoV-2 (component 1, the active ingredient of which are recombinant adenoviral vectors based on human adenovirus serotype 26, carrying the S-protein gene of the SARS-CoV-2 virus, and component 2, the active ingredient which are recombinant adenoviral vectors based on human adenovirus serotype 5 carrying the gene for the S protein of the SARS-CoV-2 virus) have been completed.

Detailed results of preclinical studies are presented in the investigator's brochure.

Studies in mice, guinea pigs, and rabbits

The program for the study of toxicity in rodents (mice, rabbits, guinea pigs) was carried out on the basis of the Center for Preclinical Studies, First Moscow State Medical University. I. M. Sechenov of the Ministry of Health of Russia (Sechenov University).

A toxicity study was carried out with a single administration of each component separately on mature outbred mice and rabbits of both sexes. A total of 400 mice (200 males and 200 females) and 24 rabbits (12 males and 12 females) were used in a single dose toxicity study. The vaccine was administered to mice intramuscularly and intravenously at increasing doses: 108, 109, 1010, 1011 virus particles/dose. During the experiment, the death of animals was not recorded, symptoms of intoxication were not detected. Necropsy performed 14 days after administration of the combined vector vaccine showed no abnormalities in the structure of the internal organs. The presence of a local irritant effect in the study drug when administered intravenously and intramuscularly was not established.

A toxicity study was conducted with a single injection of component 1, then component 2 (in the mode provided for clinical use) on mature rabbits of both sexes. A total of 24 rabbits were used in the single dose toxicity study: 12 males and 12 females. The vaccine was administered intramuscularly in increasing doses: 1010 and 1011 viral particles/dose. During the experiment, the death of animals was not recorded, symptoms of intoxication were not detected. Necropsy performed 14 days after administration of the combined vector vaccine showed no abnormalities in the structure of the internal organs. According to the data obtained, the toxic effect of the vaccine on the state of the internal organs and systems of the body using biochemical, hematological, coagulometric, physiological and pathomorphological methods of research was not revealed. The presence of a local irritant effect in the studied drug, in the framework of the experiment, was not established.

The allergenic properties of components 1 and 2 of the SARS-CoV-2 vector vaccine in albino guinea pigs at doses of 109 and 1010 viral particles/dose were not detected in tests of general anaphylaxis and conjunctival test.

During the study of immunotoxic properties, no effect of components 1 and 2 of the vector vaccine on the immune system of mice in a delayed-type hypersensitivity reaction (DTH) was found at doses of 108 and 109 viral particles/dose. There was no effect on the activity of peritoneal macrophages and the formation of antibodies in the hemagglutination reaction. The effect of the vaccine on the mass and cellularity of immunocompetent organs was not revealed. These studies indicate the absence of immunotoxic properties of components 1 and 2 of the vector vaccine for the prevention of SARS-CoV-2.

Protection studies in sensitive animal species

Lethal model in Syrian hamsters

The administration of the vaccine allows to form a protective immune response that protects 100% of animals (Syrian hamsters with induced immunodeficiency) from a lethal infection caused by the SARS-CoV-2 virus. Analysis of viral load in the lungs of infected hamsters showed a significant decrease in viral load in the group of vaccinated animals at 6, 8 and 10 days after infection. Lung damage in the group of vaccinated animals is significantly less than in non-vaccinated animals, both according to pathomorphological and histological data. An antibody-dependent enhancement (ADE) effect was not detected.

Non-lethal model in Syrian hamsters

The administration of the vaccine allows to form a protective immune response that protects animals (intact Syrian hamsters) from severe infection caused by the SARS-CoV-2 virus. Analysis of viral load in the lungs of infected hamsters showed a significant decrease in viral load in the group of vaccinated animals at 6, 8 and 10 days after infection. Lung damage in the group of vaccinated animals is significantly less than in non-vaccinated animals, both according to pathomorphological and histological data. ADE effect was not detected

Studies in primates

The study of acute toxicity and the ADE effect in primates after intramuscular administration with the provision of an interim report on the results of the study on the 28th day of follow-up was carried out on the basis of the Federal State Budgetary Institution “Central Research Institute No. 48” of the Ministry of Defense of Russia.

The total number of animals used in the work is 17 individuals, 11 in the experimental group and 6 in the control group. No toxic effects were registered. 5 animals were left to observe the duration of the preservation of the antibody response.

The phenomenon of antibody-dependent increase in infection in a previously vaccinated person can be associated with: inadequate immunization, with the peculiarities of the interaction of the pathogen of an infectious disease with the human immune system, with the peculiarities of the epidemic focus in which vaccination is carried out.

A study of the ADE effect was carried out in vaccinated and unvaccinated animals after they were infected with SARS-CoV-2. To evaluate the ADE effect, monkeys were sacrificed with an overdose of anesthetics. During the autopsy of the animals, a macroscopic assessment of the state of the lungs and sampling of biomaterial for histological examination and assessment of the virus titer were performed in 6 control animals and 6 immunized animals after infection: on days 2, 4, and 6, 4 animals each (two animals from each cohort). During a macroscopic visual examination of the slaughtered monkeys in dynamics, it was noted that the state of the lungs of vaccinated animals and monkeys receiving placebo differ significantly - in the lungs of control animals, changes are noted that are characteristic of damage by the SARS-CoV-2 virus, which were not detected in vaccinated animals, changes in the direction of burdening the infection did not occur. ADE effect is missing.

The results of daily monitoring of body weight and temperature of rhesus monkeys, monitoring of the physical condition (behavior, appearance, physiological functions), the presence of general symptoms of monkey intoxication, local reactions, changes in laboratory parameters vaccinated with a vector vaccine against COVID-19 based on recombinant adenoviruses expressing SARS-CoV-2 virus S-protein gene, indicate the safety of the study vaccine when administered intramuscularly in primates at a human therapeutic prime-boost dose.

Acute toxicity study M.P. Chumakov RAS” was carried out on monkeys of the common marmoset species (Callithrix jacchus) with a confirmed health status, born and raised under conditions of isolated keeping in a vivarium certified for work with pathogens of III–IV hazard groups. The total number of animals used in the work was 18 individuals (16 males and two females) aged 2–5 years: 12 in the experimental group (10 males and 2 females) and 6 in the control group (6 males). No toxic effects were registered. Six animals were left to observe the duration of the preservation of the antibody response. The study of the ADE effect was carried out on the basis of the Federal State Budgetary Institution “48 Central Research Institute” of the Russian Ministry of Defense in 6 vaccinated and 6 non-vaccinated animals after they were infected with SARS-CoV2. To evaluate the ADE effect, monkeys were sacrificed with an overdose of anesthetics. During the autopsy of the animals, a macroscopic assessment of the state of the lungs and sampling of biomaterial for histological examination and assessment of the virus titer were performed in 6 control animals and 6 immunized animals after infection: on days 2, 4, and 11, 4 animals each (two animals from each cohort). During a macroscopic visual examination of the slaughtered monkeys in dynamics, it was noted that the state of the lungs of vaccinated animals and monkeys receiving placebo differ significantly - in the lungs of control animals, changes are noted that are characteristic of damage by the SARS-CoV-2 virus, which were not detected in vaccinated animals, changes in the direction of burdening the infection did not occur. ADE-effect was absent.

Conclusions based on the results of preclinical studies

The he obtained data of the study indicate the absence of toxicity of the combined vaccine in a wide range of doses on different types of laboratory animals. The safety shown in preclinical studies has led to clinical studies involving volunteers.

2.4 Summary of clinica study results

Safety, immunogenicity, and efficacy of the Gam-Kovid-Vak vaccine has been evaluated in studies:

1) “An open study of the safety, tolerability and immunogenicity of the drug Gam-COVID-Vac solution for intramuscular injection with the participation of healthy volunteers" according to the protocol 02-Gam-COVID-Vac-2020
2) “An open study of the safety, tolerability and immunogenicity of the drug "Gam-COVID-Vac Lyo” lyophilizate for the preparation of a solution for intramuscular injection with the participation of healthy volunteers" according to the protocol 03-Gam-COVID-Vac Lyo-2020
3) No. 04-Gam-COVID-Vac-2020 “Randomized, double-blind, placebo-controlled, multicenter clinical trial of the efficacy, immunogenicity and safety of the combined vector vaccine Gam-COVID-Vac in parallel groups in the prevention of coronavirus infection caused by the SARS-CoV-2 virus”
4) No. 05-Gam-COVID-Vac -2020 “Open study of the safety, tolerability and immunogenicity of the drug Gam-COVID-Vac, solution for intramuscular injection, with the participation of volunteers in the age group over 60 years old”

Detailed information on the results of clinical studies conducted with other vaccines based on the adenoviral vector platform is presented in the investigator's brochure. Below are key research data.

2.4.1 Phase I/II studies

Study title: An open study of the safety, tolerability and immunogenicity of the drug Gam-COVID-Vac solution for intramuscular injection in healthy volunteers.
Protocol number: 02-Gam-COVID-Vac-2020.
Research Center and Principal Investigator: Federal State Budgetary Institution Main Military Clinical Hospital named after Academician N. N. Burdenko” of the Ministry of Defense of the Russian Federation, branch No. 7.
Legal address: Russia, 105229, Moscow, Hospital Square, 3.
Actual address: Russia, 141306, Moscow region, г. Sergiev Posad-6, st. Novaya, .4.
Principal Investigator: Gagarina Irina Viktorovna, Infectious Diseases Specialist, PhD of Medical Sciences.
First participant screening: 17-Jun-2020
Start of stage I: 17-Jun-2020
Start of stage II: 19-Jun-2020
End of day 28 of follow-up: 20-Jul-2020
End of day 42 of follow-up: 03-Aug-2020
SUMMARY – CONCLUSIONS

A total of 43 volunteers were screened, as a result of which 18 volunteers (+2 understudies) were included in the first stage and 20 volunteers (+3 understudies) were included in the second stage. Doubles were not required. Thus, 38 healthy volunteers took part in the study and received the study drug: 18 volunteers in stage 1 (9 volunteers received Component I and 9 volunteers - Component II) and 20 volunteers in stage 2. All 38 volunteers completed the study according to the protocol.

The first stage involved only men (n=18), all Caucasians, the average age of the volunteers (mean (standard deviation, SD)) was 26.6 (5.60) years. The body mass index (mean (SD)) was 25.7 (2.75) kg/m^2.

The second stage involved 14 men and 6 women, all Caucasian, the average age of the volunteers (mean (standard deviation, SD)) was. 26.4 (4.36) years old. Body mass index (mean (SD)) was 24.4 (3.11) years.

There were no significant medical conditions that could preclude participation in the study. 1 stage 1 volunteer had a history of appendectomy and 2 stage 2 volunteers had an appendectomy (n=1) and meniscus removal (n=1). The results of the physical examination at screening were without pathology. Test results for HIV, syphilis, hepatitis B and C were negative in all volunteers. There were no indications for vaccination in the 30-day period prior to screening. Antibodies IgM and IgG to the SARS-CoV-2 virus (according to the results of a qualitative analysis) were not detected. Other screening surveys met the inclusion/non-inclusion criteria.

All 38 volunteers received the study drug according to the protocol, without deviations from the planned regimen.

IMMUNOLOGICAL EFFICIENCY ANALYSIS RESULTS:

The efficacy analysis included all volunteers who completed the study in accordance with the Protocol - 38 volunteers (18 in the first stage and 20 in the second stage).

The data obtained allow us to confirm the hypothesis of the study that administration of the developed vaccine induces the formation of a tense immune response to the SARS-CoV-2 virus, involving both humoral and cellular immunity.

SAFETY ANALYSIS RESULTS:

The safety analysis included 38 volunteers (all volunteers who received a dose of study drug) (18 volunteers in the first phase and 20 volunteers in the second phase of the study).

The frequency of development of any AE

A total of 295 AE were reported during this study. 120 AE were registered in volunteers of the first stage of the study, of which 73 AE were in volunteers who received the first component of the drug (60.1%), and 47 AE in volunteers who received the second component of the drug (39.9%). 175 AE were reported in volunteers who participated in the second phase of the study. AE were registered in all volunteers who took part in the study without exception (in both stages).

SAE frequency

No SAE were reported in the study.

Causal relationship with the experimental drug

At the first phase of the study, 119 AE were reported with at least a possible connection with the study drug (i.e. certain, possible or probable). These AE were observed in all 9 volunteers in the group who received the first component of the drug (72 events) and in all volunteers who received the second component of the drug (47 events).

According to their connection with the intake of the study drug, the AE were distributed as follows. A total of 12 (Component I) and 7 (Component II) AE were reported that were clearly related to the study drug, 18 (Component I) and 5 (Component II) AE that were considered possibly related to the study drug, 42 (Component I) and 35 (Component II) AE assessed as likely to be related to study drug, 1 (Component I) AE with questionable drug association.

In the second phase of the study, 164 AE were reported with at least a possible connection with the study drug (i.e. certain, possible or probable). 4 AE were considered to be in doubt with the drug, 2 AE were considered to be conditionally related to the drug, and in 4 AE the relationship was considered “unknown” by the investigator.

At the second phase of the study, 164 AE were reported with at least a possible association with the study drug (i.e. certain, possible or probable). 4 AE were considered to be in doubt with the drug, 2 AE were considered to be conditionally related to the drug, and in 4 AE the relationship was considered “unknown” by the investigator.

<u>AE severity</u>

At the first stage, AE of mild severity prevailed in terms of severity (73 AE in the group that received the first component of the drug and 45 AE in the group that received the second component of the drug); 2 AE of moderate severity were registered in the group that received the second component of the drug (increased AST levels and hyperthermia in different volunteers).

At the second stage, AE of mild severity prevailed in terms of severity (169 AE), 6 AE of moderate severity were registered, which were associated with asthenia – 2 cases, pain (this MedDRA term means body aches accompanied by arthralgia) – 1 case, hyperthermia (increase in body temperature up to 38.7°C – 1 case), headache – 2 cases.

No severe AE were reported during the study.

<u>AE outcomes</u>

None of the AE at either Stage 1 or Stage 2 resulted in withdrawal of the volunteer from the study or discontinuation of the study drug.

Most AE ended in recovery without sequelae.

At the first stage of the study, recovery was completed without consequences: 44 AE in the group that received the first component of the drug and 21 AE in the group that received the second component of the drug. For AE 29 and 26, respectively, outcome was unknown at study day 42, and volunteers continue to be followed up to day 180.

At the second stage of the study, 144 AE ended in recovery without consequences. On study day 42, outcome was unknown for 27 AE, and recovery was ongoing for 4 AE. Volunteers, according to the Protocol, continue to be monitored up to 180 days.

<u>Description of AE</u>

At the first stage, among the adverse reactions, the most frequent were deviations of laboratory and instrumental parameters (SOC “Laboratory and instrumental data”) and “General disorders and reactions at the injection site”.

The AE of the category “Laboratory and instrumental data” included:

- Abnormalities in immunological status: increase in the number of T-lymphocytes, decrease in the number of natural killer cells, increase in the number of B-lymphocytes, increase in the number of CD4-lymphocytes, increase in the number of CD8-lymphocytes, increase in the level of immunoglobulin E (IgE) in the blood, increase in the number of natural killer cells, decrease in the CD4/CD8 ratio, increase in the level of immunoglobulin M (IgM) in the blood, decrease in the number of CD4-lymphocytes, decrease in the number of CD8-lymphocytes, decrease in the level of immunoglobulin M (IgM) in the blood, decrease in the number of T-lymphocytes, increase in the ratio CD4/CD8.

- Abnormalities in KLA: increase in the number of monocytes, decrease in the percentage of lymphocytes, increase in the erythrocyte sedimentation rate, increase in the number of leukocytes, increase in the number of platelets, increase in the percentage of lymphocytes.
- Deviations in the BAC: increased levels of aspartate aminotransferase, increased levels of lactate dehydrogenase in the blood, increased levels of cholesterol in the blood.
- Increase or decrease in blood pressure.

AEs in the category “*General disorders and reactions at the injection site*” were represented by reactions such as pain at the vaccination site, hyperthermia, asthenia, pain (this MedDRA term means body aches accompanied by arthralgia), decreased appetite, itching at the vaccination site.
Less common AE were: headache, palpitations (palpitations), diarrhea, pain in the oropharynx, and urticaria (this allergic reaction occurred in one volunteer who received Component I).
At the second stage, AE of the MedDRA system-organ class category “Laboratory and instrumental data” were most frequently noted. The next in frequency were AE of the category “*General disorders and reactions at the injection site*”. Other AE were less common.
The AE category "*General disorders and reactions at the injection site*" included: hyperthermia, pain at the vaccination site, asthenia, pain, malaise, pyrexia, increased skin temperature at the vaccination site, swelling at the vaccination site.
The AE of the category "*Laboratory and instrumental data*" included:

- Abnormal immunological status: increased T-lymphocytes, increased percentage of lymphocytes, decreased natural killer cells, increased CD4-lymphocytes, decreased CD4-lymphocytes, increased B-lymphocytes, decreased B-lymphocytes, increased natural killer cells, increased CD8 lymphocytes, increased blood immunoglobulin E (IgE), increased CD4/CD8 ratio, decreased CD4/CD8 ratio, increased blood immunoglobulin A (IgA), decreased percentage of CD8 lymphocytes.
- Deviations in the KLA: an increase in the percentage of lymphocytes, a decrease in hematocrit, an increase in the number of lymphocytes, an increase in the erythrocyte sedimentation rate, an increase in the number of leukocytes, an increase in the number of monocytes, an increase in the number of platelets, a decrease in the number of neutrophils, a decrease in the number of platelets.
- Deviations d OAM: Erythrocytes in the urine.

Other more rarely encountered AE were headache, diarrhea, pain in the oropharynx, nasal congestion, sore throat, rhinorrhea.

In general, it can be said that the AE identified during the first and second stages of the study are typical for most vaccine drugs. There were no cases of severe AE development.

Key vital signs

The average values of the main vital signs of volunteers (body temperature, systolic and diastolic blood pressure, heart rate, respiratory rate) obtained at visits according to the schedule of protocol procedures did not change significantly. Individual cases of increased body temperature or fluctuations in blood pressure were recorded as corresponding AE.

Laboratory indicators

Isolated cases of clinically significant abnormalities from the laboratory parameters were registered, which were registered as adverse events of the system organ class category of the MedDRA dictionary "Laboratory and instrumental data".

ECG in 12 leads

There were no clinically significant deviations from the norm in terms of ECG indicators in 12 leads during the first and second stages.

<u>CONCLUSIONS</u>:

- Both a single immunization with component I or II of the Gam-COVID-Vac drug, and a two-time prime-boost immunization with the Gam-COVID-Vac drug can lead to the formation of antigen-specific IgG antibodies in 100% volunteers 21 days after immunization. With double immunization in the "prime-boost" regimen with the drug "Gam-COVID-Vak", the maximum antibody titers were shown by the 42nd day after the start of immunization.
- Double prime-boost immunization with Gam-COVID-Vac leads to significantly higher titers of antigen-specific IgG antibodies compared to single immunization with component I or II of Gam-COVID-Vac, which shows the advantages of the booster vaccination scheme and allows us to recommend it for further clinical practice.
- When comparing the titers of antigen-specific IgG antibodies in vaccinated volunteers on the 28th day from the start of vaccination with the titers of antigen-specific IgG antibodies in convalescents after COVID-19, it was shown that with a single injection, the titers are comparable, and with prime-boost vaccination, titers in vaccinated volunteers exceed the titers of convalescents.
- In volunteers, after administration of component 1 of the vaccine on day 14, there is a significant increase in the level of VNA to the SARS-CoV-2 virus ($p = 0.0002$). In volunteers who received both components of the vaccine (component 1 and component 2 with an interval of 21 days), on the 28th day from the start of vaccination, VNA to the SARS-CoV-2 virus were detected in the blood serum with an average titer of 16.3 (95% confidence interval 10 .4-25.4), the seroconversion rate was 95%. On the 42nd day from the start of vaccination, VNA to the SARS-CoV-2 virus was detected in the blood serum of volunteers with an average titer of 49.3 (95% confidence interval 33.2-73.1), the seroconversion rate was 100%.
- The administration of the vaccine GamKovidVak in the full scheme (component 1 and component 2 with an interval of 3 weeks) allows to induce VNA in 95% of volunteers already on the 7th day after boosting immunization (day 28 from the start of immunization). On day 21 after booster immunization (day 42 from the start of immunization), 100% of volunteers had VNA detected in the blood serum at a level that was detected in convalescents after suffering COVID-19 coronavirus infection, which indicates the high efficacy of the vaccine.

- Both single immunization with component I or II of the drug "Gam-COVID-Vac", and two-time immunization in the "prime-boost" mode with the drug "Gam-COVID-Vac" can cause the formation of a tense antigen-specific cellular link of anti-infective immunity, which is confirmed by a high degree of statistical significance in the measured parameters before and after immunization.
- Both a single immunization with component I or II of the Gam-COVID-Vac drug, and a two-time prime-boost immunization with the Gam-COVID-Vac drug are able to form antigen-specific cells of both populations of T lymphocytes: T helper (CD4+) and T-killer (CD8+).
- Double immunization in prime-boost mode with Gam-COVID-Vac (both in terms of lymphoproliferative response and increase in IFNɣ concentration) forms cellular antigen-specific immunity of greater intensity compared to single immunization with component I or II drug "Gam-COVID-Vak".
- A single immunization with component I or II of the Gam-COVID-Vac drug leads to a significant increase in IFNɣ secretion on day 14 compared with study day 0 (before immunization of volunteers), while the values of the lymphoproliferative response on study day 14 do not statistically differ from 0 day indicators. A significant statistical difference in the rates of lymphoproliferative response is observed at a later date of the study (day 28).
- According to the results of the study, there was no significant correlation between the titer of neutralizing antibodies against recombinant viral vectors on day 0 and the titer of RBD-specific IgG in the blood serum of volunteers on days 14, 21, 28 and 42 from the start of vaccination, therefore, the presence of a pre-existing immune response to components of vaccine vectors does not affect the effectiveness of vaccination.
- The safety analysis of the study results (up to day 42 of follow-up) showed that any AE were observed in all volunteers who received the study vaccine in both the first and second phases of the study.
- Most of the identified AE, both in the first and second phases of the study, were of mild severity and ended in recovery. There were no cases of severe AE development.
- The AE identified in both the first and second phases of the study are typical for most vaccine medicinal products and in most cases were laboratory abnormalities or AE of the category "General disorders and reactions at the injection site".
- During the first and second stages of the study, there were no AE that would require the volunteer to be excluded from the study, as well as serious AE (SAE).

2.4.2 Phase II, III, and IV study

Randomized, double-blind, placebo-controlled, multicenter clinical trial of the efficacy, immunogenicity, and safety of the Gam-COVID-Vac combined vector vaccine in parallel groups in the prevention of coronavirus infection caused by the SARS-CoV-2 virus"

Study period:

First Participant Screening: 07-Sep-2020

First Injection: 09-Sep-2020

Last randomized volunteer 29-Dec-2020

Study is currently ongoing

Study Goals and Endpoints

	**Primary goal**	**Primary endpoint**
Efficiency	Demonstrate superiority of Gam-COVID-Vac Combination Vector Vaccine for the Prevention of Coronavirus Infection Caused by SARS-CoV-2 Virus versus placebo based on the proportion of study subjects with coronavirus disease (COVID-19) developed before 6 months after receiving a second dose of ED/placebo and confirmed by polymerase chain reaction (PCR)	Proportion of study subjects with COVID-19 disease developing up to 6 months after receiving a second dose of ED/placebo and confirmed by PCR after vaccination was scheduled. The prophylactic efficacy of the investigational vaccine should be ≥50% compared to placebo, with the lower limit of the confidence interval of the point estimate on the primary efficacy variable should be >33%.
	**Secodnary goals**	**Secondary endpoints**
Efficiency	Demonstrate superiority of Gam-COVID-Vac Combination Vector Vaccine for the Prevention of Coronavirus Infection with SARS-CoV-2 Virus compared to placebo based on the proportion of study subjects with anti-SARS-CoV-2 N-protein antibodies developed after vaccinations on days 42 and 180 compared to day 1.	Proportion of study subjects with anti-SARS-CoV-2 N-protein antibodies post-vaccination on days 42 and 180 compared to day 1. Preventive efficacy of study vaccine should be ≥50% compared to placebo, with lower confidence interval the point estimate for the primary efficacy variable should be >30%.
Efficiency	To evaluate the effectiveness of "Gam-COVID-Vac Combined vector vaccine for the prevention of coronavirus infection caused by the SARS-CoV-2 virus" in comparison with placebo, based on the severity of the clinical course of COVID-19	• Proportion of study subjects with mild, moderate, severe, and very severe COVID-19 developing up to 6 months after receiving the first dose of ED/placebo;<br>• Duration of COVID-19

Efficiency	Ealuate the effectiveness of "Gam-COVID-Vac Combined vector vaccine for the prevention of coronavirus infection caused by the SARS-CoV-2 virus" in comparison with placebo, based on COVID-19 outcomes	Proportion of study subjects who died due to COVID-19 among those who got infected
Immunogenicity	Assess the immunogenicity of Gam-COVID-Vac Combined Vector Vaccine for the Prevention of Coronavirus Infection Caused by the SARS-CoV-2 Virus, in comparison with placebo, based on the geometric mean of the SARS-CoV-2 glycoprotein-specific antibody titer	Geometric mean titer of SARS-CoV-2 glycoprotein-specific antibodies measured in up to 9,520 study subjects on the day of dosing prior to the first dose of ED/placebo and 42 ± 2 and 180 ± 14 days after the first dose
Immunogenicity	Assess the immunogenicity of Gam-COVID-Vac Combination Vector Vaccine for the Prevention of Coronavirus Infection Caused by SARS-CoV-2 Virus compared to placebo based on seroconversion rate	Proportion of study subjects with a 4-fold or higher increase in antibody titer to SARS-CoV-2 glycoprotein, detected in no more than 9,520 study subjects on the day of administration of the drug before receiving the first dose of ED/placebo and 42±2 and 180±14 days after receiving first dose
Immunogenicity	Describe the state of tension of the cellular immune response when using "Gam-COVID-Vac Combined vector vaccine for the prevention of coronavirus infection caused by the SARS-CoV-2 virus" in comparison with placebo	The level of interferon gamma concentration in the culture of T-lymphocytes after restimulation with SARS-CoV-2 glycoprotein, determined in 107 study subjects on the day of drug administration before receiving the first dose of ED/placebo and 28 ± 2 days after receiving the first dose
Immunogenicity	Describe the state of the antigen-specific cellular immune response when using "Gam-COVID-Vac Combined vector vaccine for the prevention of coronavirus infection caused by the SARS-CoV-2 virus" in comparison with placebo	The number of proliferating CD4 cells and CD8 cells in response to mitogen stimulation and their ratio, determined in 50 study subjects on the day of administration of the drug before receiving the first dose of ED/placebo and 28 ± 2 days after receiving the first dose
Immunogenicity	Describe the immunogenicity of Gam-COVID-Vac Combined Vector Vaccine for Prevention of Coronavirus Infection Caused by SARS-CoV-2 virus in Comparison with Placebo	Geometric mean VNA titer measured in 300 study subjects on the day of drug administration before receiving the first dose of ED/placebo and 42 ± 2 days after receiving the first dose

Version number: 3.0	Version data: 23.07.2021	Page 44 of 145
Double-blind, placebo-controlled study with an open dose selection period to assess the safety, tolerability, and immunogenicity of the drug "Gam-COVID-Vak M combined vector vaccine for the prevention of coronavirus infection caused by SARS-CoV-2 virus" in adolescents		
Protocol No. 07-Gam-COVID-Vac-2021		CONFIDENTIAL

Safety	To evaluate the safety of using "Gam-COVID-Vac Combined vector vaccine for the prevention of coronavirus infection caused by the SARS-CoV-2 virus" in comparison with placebo	Frequency and severity of adverse events (including adverse events of special interest) occurring in study subjects within 6 months after receiving the first dose of ED/placebo

Assessment criteria:

The choice of these parameters was due to the results of our own preclinical studies, as well as data from literature sources.

The following indicators of immunological efficacy were evaluated:

- Indicators of epidemiological effectiveness
- Indicators of the intensity of the humoral immune response:
- Seroconversion rate
- Geometric mean titers of antibodies specific to SARS-CoV-2 glycoprotein S
- Titers of neutralizing antibodies
- Indicators of the intensity of the cellular immune response:
- Percentage of proliferating T-lymphocytes after restimulation with SARS-CoV-2 glycoprotein S or RBD
- Increase in the concentration of interferon gamma in the culture of T-lymphocytes after restimulation with glycoprotein S or RBD SARS-CoV-2

The safety criteria assessed included:

- data on the development of AE,
- results of assessment of indicators of vital functions,
- results of instrumental studies,
- physical examination results

Study methodology

Randomized, double-blind (blinded for the subject of the study and the researcher), placebo-controlled, multicenter, parallel-group clinical study to evaluate the efficacy, immunogenicity and safety of the Gam-COVID-Vac Combined vector vaccine for the prevention of coronavirus infection caused by the SARS-CoV virus -2" in adult subjects of the study in the prevention of coronavirus infection caused by the SARS-CoV-2 virus.

To achieve the objectives of the study, it was planned that 34,213 study subjects would have to complete their participation in the study as planned. Given the potential for early dropout and the lack of data to assess the primary endpoint, it was planned to screen 40,000 volunteers over the age of 18 years. Approximately 39,000 study subjects were expected to complete all screenings.

Statistical methods:

Descriptive statistics

The report provides descriptive statistics characterizing the volunteers included in the study. For each quantitative indicator (age, weight, laboratory test data, vital signs), the following were calculated:

- Number of non-missing values (N);
- Minimum value (Min);
- Maximum value (Max);
- Arithmetic mean (Mean);
- Standard deviation (SD);
- 95% confidence interval (CI) for the mean;
- Median (Me);
- Interquartile range (IQR).

For qualitative and categorical indicators (gender, frequency of abnormalities according to physical examination data, incidence of adverse events, etc.), absolute values are presented in the n/N format, as well as the proportion (%).

Application of parametric and non-parametric statistical methods

In data analysis, quantitative variables were pre-tested for normal distribution using the Shapiro-Wilk test, as well as a test for skewness and kurtosis, indicating the p value when testing the null hypothesis of the normal distribution of the variable. In the case of a normal distribution, parametric tests were used for statistical analysis: t-test, paired t-test. With a significant deviation from normality ($p<0.05$), nonparametric tests were used: the Mann-Whitney test, the Wilcoxon test. A paired t-test was used to assess the dynamics of indicators between visits. The $\chi 2$-test and, if necessary, Fisher's exact test (if the expected frequency in any of the cells was less than 5) were used to compare the frequency indicators between groups.

Analysis of efficacy and immunogenicity parameters

Data analysis for the primary endpoint was performed using the $\chi 2$-squared test. The odds ratio (OR) of the disease in the two groups was assessed, and a 95% confidence interval for the OR was calculated. Additionally, to analyze various covariates (gender, age, history data, etc.), we used the construction of a logistic regression model.

To compare secondary endpoints that are numerical data (geometric mean of SARS-CoV-2 glycoprotein-specific antibodies, interferon gamma concentration in T-lymphocyte culture after restimulation with SARS-CoV-2 glycoprotein S or RBD, number of proliferating CD4 cells and cells CD8 in response to mitogen stimulation and their ratio, geometric mean VNA titer) for intergroup comparison, the t-test or Mann-Whitney test was used (depending on the type of data distribution). Intragroup comparison was a paired t-test.

For analysis of frequency variables (proportion of study subjects with anti-SARS-CoV-2 N-protein antibodies, proportion of study subjects with mild, moderate, severe, and very severe COVID-19, proportion of study subjects who died due to COVID-19, proportion of subjects studies with a four-fold or higher increase in the titer of antibodies against the SARS-CoV-2 glycoprotein), the $\chi 2$ test was used and, if necessary, the Fisher's exact test (if the expected frequency in any of the cells was less than 5).

Analysis of security settings parameters

Medical Dictionary during the database formation phase until the database was closed interim. The incidence of AE/SAE during treatment was calculated for each organ system (SOC) and preferred term (PT) by the absolute and relative number of volunteers who developed adverse events. Summary data on the severity of AE, their relationship with the drug, outcomes are presented for each organ system. Separately, data are presented on serious adverse events (SAE), AE leading to the need for early termination of the study by a volunteer, as well as AE with CTCAE severity of 3 or more.

To analyze categorical safety parameters (frequency of AE/SAE, incidence of abnormalities according to physical examination and vital signs, frequency of allergic reactions), descriptive statistics methods were used indicating the absolute number of volunteers in the n/N format, as well as the proportion of volunteers in each categories.

For the presentation of quantitative data (vital signs), descriptive statistics methods were used, indicating the following parameters:

- Number of non-missing values (N);
- Arithmetic mean (Mean);
- Standard deviation (SD);
- 95% confidence interval (CI) for the mean;
- Interquartile range (IQR).

A paired t-test was used to assess changes in quantitative indicators from baseline.

RESULTS

At the moment, the Federal State Budgetary Institution “National Research Center for Epidemiology and Microbiology named after N.F. Gamaleya" of the Ministry of Health of Russia is held "Randomized, double-blind, placebo-controlled, multicenter clinical trial of the efficacy, immunogenicity and safety of the combined vector vaccine Gam-COVID-Vac in parallel groups in the prevention of coronavirus infection caused by the SARS-CoV-2 virus" according to protocol: No. 04-Gam-COVID-Vac -2020.

Study is currently ongoing**.**

RESULTS OF SAFETY AND EFFICIENCY ANALYSIS:

Interim Safety Report 3 describes the results of a follow-up of 33,771 subjects vaccinated with the two components of the Gam-COVID-Vac/placebo vaccine with data processing.

The analysis included data from 33,771 subjects vaccinated with the two components of the Gam-COVID-Vac/placebo vaccine, for which the data were entered as fully as possible into the e-RCF (demographic and anthropometric indicators, physical examination results before and after vaccination, etc.).

The study included volunteers aged 18 to 92 years. The mean age was 43.9 ± 12.1 years in group I (IP) and 43.9 ± 12.0 years in group II (Placebo). 22419 male volunteers (16771 in the IP group and 5648 in the placebo group) and 11347 female volunteers (8545 in the IP group and 2799 in the placebo group) were included.

Analysis of data on comorbidities showed that 5501 volunteers (22.7%) in the PI group and 1889 volunteers (23.2%) in the placebo group had comorbidities.

For most of the volunteers in both groups, the risk of infection was assessed as general. However, for 87 volunteers (0.4%) in the PI group and 28 volunteers (0.4%) in the placebo group, this risk was assessed as high.

The vast majority of volunteers were Caucasian (about 97%), 1023 volunteers were of Asian race and 130 were of other race.

Physical examination results at the screening visit showed 1204 abnormalities, 54 of which were clinically significant. Most of the deviations accounted for deviations of the cardiovascular system (749 out of 1204 deviations or 62.2%). Intergroup comparison of these data did not reveal statistically significant differences ($p > 0.05$).

There were no statistically significant differences between groups in the distribution of volunteers in terms of demographic and anthropometric indicators, comorbidities, and the risk of COVID-19 infection.

The frequency and severity of adverse events that occurred in study subjects within 6 months after receiving the first dose of ED/placebo.

The safety analysis included **33,771** volunteers (all volunteers who received a dose of study drug).

The frequency of development of any AE

To date, 26405 cases of AE have been registered in the study, which developed in 12080 volunteers (35.8%).

SAE frequency

During the study period, 114 episodes of SAE were recorded: 76 in the IP group and 38 in the placebo group. In volunteers over 60 years of age, 30 episodes of SAE were reported during the study: 22 in the PI group and 8 in the placebo group. According to the investigator, all SAE were not associated with the use of ED/placebo.

Causal relationship with the experimental drug

6 SAE in the safety population were associated with ED/placebo use, according to the investigator. In the population of persons over 60 years of age, there was no association with the use of ED/placebo.

Conclusion

1) Investigational vaccine has a favorable safety profile

2) The safety profile in the 60+ age group does not differ from the safety profile in the general population of volunteers

3) No unexpected serious adverse events identified

4) Adverse events are specific to vaccine preparations in general

Reported serious adverse events are comparable between vaccine and placebo groups

Immunogenicity study

The study of the intensity of humoral immunity was carried out at the Federal State Budgetary Institution "National Research Center for Epidemiology and Microbiology named after Honorary Academician N.F. Gamaleya» of the Ministry of Health of the Russian Federation (FGBU «NITsEM named after N.F. Gamaleya» of the Ministry of Health of Russia). The titer of IgG antibodies specific to the RBD domain S of the SARS-Cov2 virus protein was determined by enzyme immunoassay (ELISA). The principle of this method is the specific interaction of the antigen with antibodies and subsequent detection using secondary antibodies against the human IgG Fc fragment labeled with horseradish peroxidase. In a humoral immune response study, sera were analyzed for antibodies specific to the receptor-binding domain of SARS-CoV-2 glycoprotein S 42 days after the start of vaccination compared to pre-vaccination baseline levels.

Analysis of data from 456 volunteers (342 from the vaccinated group and 114 from the placebo group) when comparing the level of RBD-specific antibodies between age groups, a statistically significant difference was found between the 18-30 year old group (combined data from men and women) and the rest of the age groups (p = 0.007).

In the 18-30 year old group, GMT was higher than in other groups. When comparing antibody levels between all age groups except 18-30, no statistically significant difference was found between the groups (p = 0.343). Antibody levels did not differ significantly between the male and female groups (p = 0.258). Descriptive statistics by age strata and gender are presented below.

Table 2–1. Seroconversion rate (geometric mean and 95% CI geometric mean) of RBD-specific antibodies at day 42, measured by ELISA, in volunteers immunized with vaccine (n = 342) or placebo (n = 114). Data are presented separately for groups stratified by age and gender of volunteers, as well as in general for vaccine and placebo groups.

	Age strata (years)										Total	
	18–30		31–40		41–50		51–60		60+			
	females	males	females	males	females	males	females	males	females	males	females	males
number	14	14	35	44	45	47	39	45	26	29	114	342

Seroconversion rate, %	100	100	100	100	97.78	97.87	97.44	97.78	96.15	96.55	14.91	98.25
GMT (95% CI)	18,102 (7,689-42,616)	22,067 (11,971-40,676)	10,925 (6,807-17,532)	10,106 (7,047-14,494)	7,940 (4,874-12,935)	6,123 (3,658-10,250)	8,063 (4,715-13,789)	7,129 (4,466-11,379)	10,908 (5,462-21,785)	8,128 (4,071-16,228)	3,055 (2,018 - 4,626)	8,996 (7,610-10,635)

An assessment of the titer of antigen-specific IgG was carried out in 980 volunteers (733 from the vaccinated group and 247 from the placebo group). Interim immunogenicity analysis showed that the vaccine elicited an immune response in volunteers.

On the 42nd day of the study, in the group receiving the vaccine, the geometric mean antibody titer was 9818, the seroconversion rate was 98.64%. In the placebo group, the seroconversion rate was significantly lower at 12.55% ($p<0.001$).

Parameter	Placebo	ED
Geometric mean	2.848	9,818
Lower 95% CI of geo. mean	2.001	8,713
Upper 95% CI of geo. mean	4.055	11,063

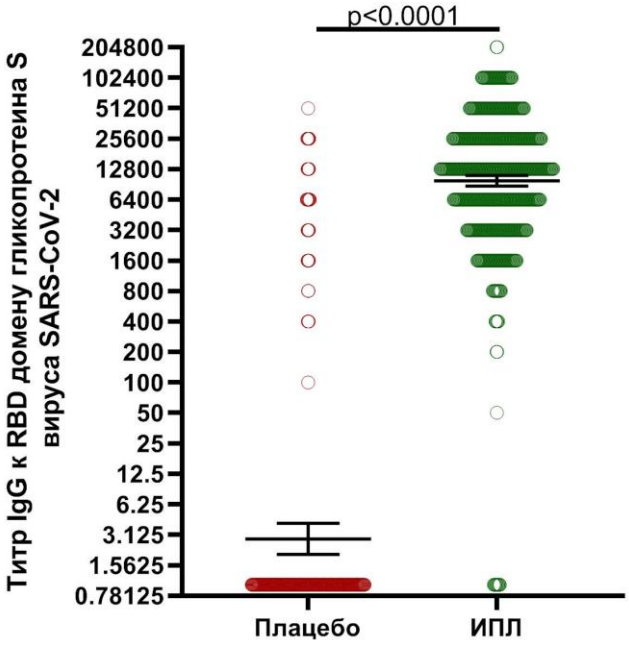

Figure 2–1. The results of the analysis of humoral immunity (antigen-specific IgG titer was carried out in 980 volunteers)

Determination of the intensity of post-vaccination humoral immunity in volunteers on day 42 after receiving the first dose, according to the virus-neutralizing activity of peripheral blood serum *in vitro*

When studying the induction of a humoral immune response, the sera of 100 volunteers were analyzed for the presence of neutralizing antibodies on the 42nd day from the start of vaccination (Table 13-10). On the 42nd day of the study in the vaccinated group,

the geometric mean titer of neutralizing antibodies was 44.47, the seroconversion rate was 95.83%. In the placebo group, the seroconversion rate was significantly lower at 7.14% ($p<0.001$). When comparing the level of neutralizing antibodies between age strata, there was no statistically significant difference between groups ($p = 0.222$), when comparing the level of neutralizing antibodies between the groups of men and women, there was also no statistically significant difference. ($p = 0.639$).

Table 2–2. Seroconversion rate and statistical data (geometric mean and 95% CI geometric mean) of VNA at day 42 in participants immunized with vaccine (n = 72) or placebo (n = 28) as measured by microneutralization test. Data are presented in groups stratified by age and sex of volunteers and in general for vaccine and placebo groups.

	Age strata					Total		Gender	
	18–30	31–40	41–50	51–60	60+	Placebo	Vaccine	females	males
Number of cases	7	15	22	21	7	28	72	26	46
Seroconversion rate, %	85.71	100.0	90.91	100.0	100.0	7.14	95.83	96.15	95.65
Geometric mean	53.84	72.94	48.32	28.75	36.23	1.562	44.47	48.21	42.49
Lower 95% CI of geo. mean	8.198	44.25	22.21	16.71	13.21	1.117	31.79	25.51	28.42
Upper 95% CI of geo. mean	353.5	120.2	105.1	49.49	99.35	2.185	62.2	91.08	63.51

According to the study results, it was shown that volunteers on the 42nd day after vaccination showed a significant increase in the titer of VNA in the blood serum, which indicates the formation of a specific immune response.

Statistical processing of intermediate research results was performed using GraphPad Prism 8.0 software. The D'Agostino-Pearson test was used to determine the normality of the data distribution. When comparing data from unrelated samples, the Mann-Whitney test was used. When comparing data from related samples, the Wilcoxon test [Petri Aviva, Sabine Caroline. Visual medical statistics. Tutorial. // GEOTAR-Media, 2015].

The D'Agostino-Pearson test was used to determine the normality of the data distribution. To identify the relationship between the results of studies of VNA to the components of vectors and RBD-specific antibodies in the blood sera of volunteers, the Spearman correlation analysis method was used.

Figure 2–2. Humoral immunity analysis results

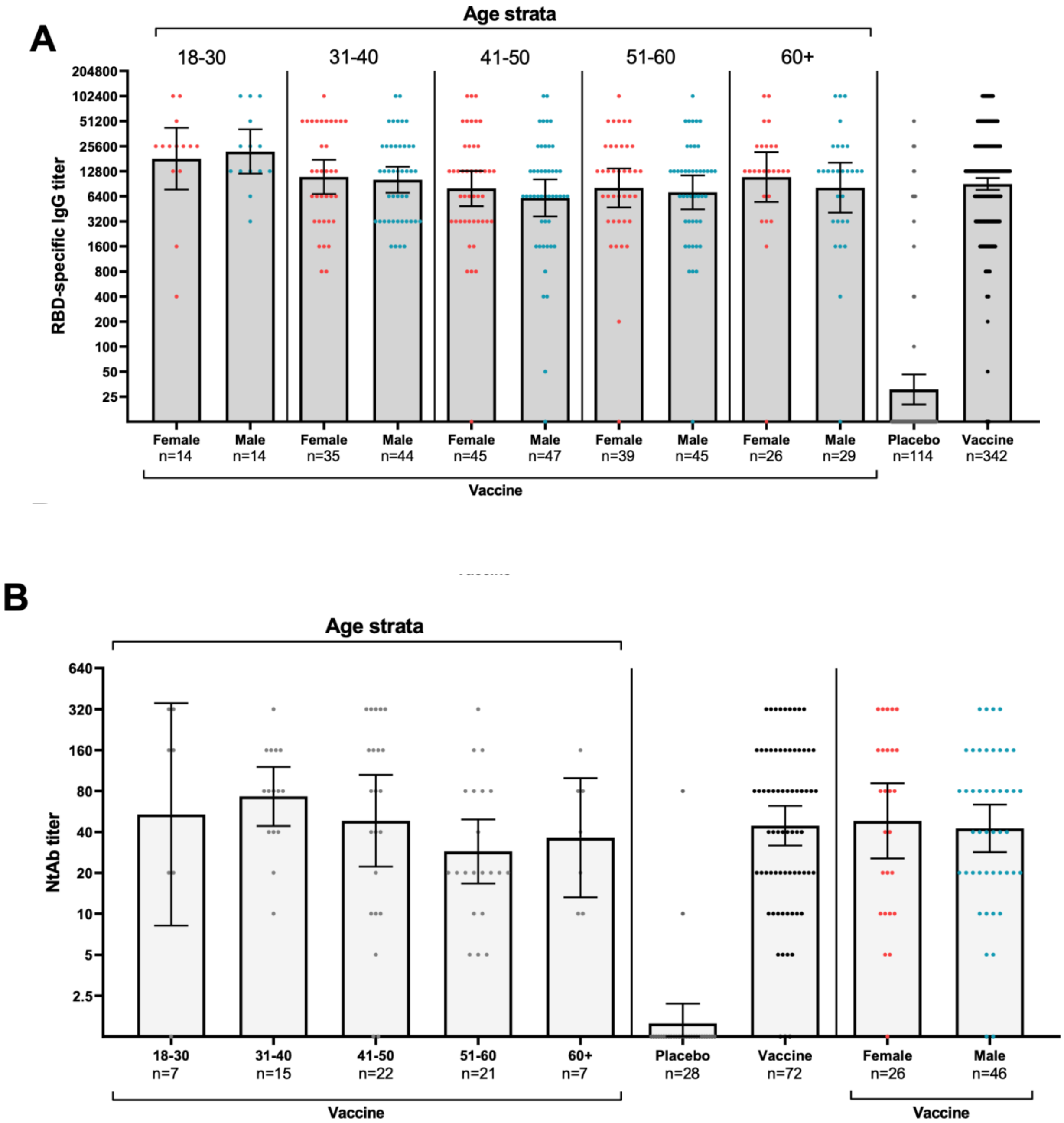

<u>Study is currently ongoing.</u>

2.5 Potential risks and benefits of investigational medicinal products for the study participant

The available data on the safety and immunogenicity of the study drug "Gam-COVID-Vac, a combined vector vaccine for the prevention of coronavirus infection caused by the SARS-CoV-2 virus" allow us to consider the developed vaccine as a drug, the potential benefit of which significantly outweighs the risk.

In the context of an unfavorable epidemiological situation, it is assumed that participants in the study will receive a clear benefit from participation in the study.

The risk of disease development, the clinical picture and features of the course of the disease

As of 14 June 2021, there were 175,686,814 registered with WHO confirmed cases of COVID-19, including 3,803,592 deaths.

The data available today indicate that children make up to 10% of those infected with SARS-CoV-2 and up to 2% of patients diagnosed with clinical cases of COVID-19.

In the Russian Federation, children account for 7.6% of reported cases of COVID-19, and the number and age structure of overt illnesses in children are not currently known.[2] The disease in newborns is extremely rare, while intrauterine transmission of the infection has not been proven.

Children make up a growing proportion of new COVID-19 cases in the US, accounting for nearly 21% at the time of writing. About 88,500 new cases of COVID-19 in children were reported between April 8 and April 15, according to the latest weekly report from the AAP and the Association of Children's Hospitals. Since the beginning of the pandemic, more than 3.63 million children have tested positive, about 13.6% of all cases. At least 297 children have died from COVID-19, accounting for about 0.06% of all deaths. About 0.01% of children diagnosed with COVID-19 have died. At least 14,849 children were hospitalized, which is about 2% of all hospitalizations. About 0.8% of children with COVID-19 are hospitalized.[3]

Children have significantly different risk factors, tk. only 9% of cases of infection with the SARS-CoV-2 virus are associated with travel to other countries, and 91% of the cases had local contacts, mainly in family foci.

More than 7,100 children and adolescents have died from COVID-19, representing 0.3 percent of the 2.4 million COVID-19 deaths in 78 countries.

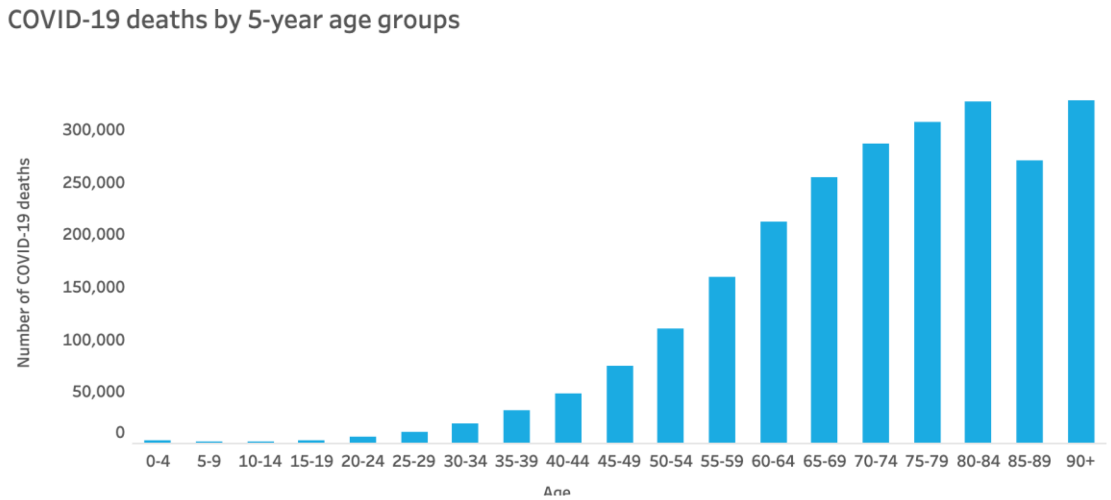

[2] "Interim guidelines "Prevention, diagnosis and treatment of a new coronavirus infection (COVID-19). Version 11 (05/07/2021)" (approved by the Russian Ministry of Health), 6. Features of managing children with COVID-19
[3] https://www.aappublications.org/news/2021/04/19/pediatric-covid-cases-041921 American Academy of Pediatrics, 2021.

To date, according to UNICEF4, the disease has claimed the lives of 1,774 children aged 0 to 4 years, 1,000 children aged 5 to 9 years, 1,591 children aged 10 to 14, and 2,826 children aged 15 to 19 worldwide.

Children have a milder course of the disease compared to adults, the development of viral pneumonia is not typical, the symptoms are less pronounced, and deaths are extremely rare. In children, as in adults, fever and respiratory syndrome dominate, but lymphopenia and inflammatory markers are less pronounced.

The incubation period in children ranges from 2 to 10 days, more often 2 days.

The clinical symptoms of COVID-19 in children correspond to the clinical picture of SARS caused by other viruses: fever, cough, sore throat, sneezing, weakness, myalgia. The severity of the febrile reaction may be different: fever up to 38 ° C is observed in half of the sick children, in a third of the children an increase in body temperature from 38.1 to 39.0 ° C is recorded.

The accumulated experience of monitoring manifest cases of COVID-19 in children shows that the characteristic combination of fever, cough and shortness of breath occurs only in 73% (in adults - 93%). Comparison of the frequency of individual symptoms also shows their predominance in adults. According to US researchers, only 56% of pediatric patients reported fever, 54% cough, and 13% dyspnea, compared with 71%, 80%, and 43%, respectively, among patients aged 18 to 64 years. In children, not only are clinical manifestations of intoxication (headache 28% versus 58% in adults; myalgia - 23% versus 61% of adults) and respiratory failure less common, but also gastrointestinal manifestations (nausea / vomiting - in 11% versus 16% ; abdominal pain - 5.8% versus 12%; diarrhea - 13% versus 31%). Tachycardia is noted in half of the hospitalized children, tachypnea - in a third. Children rarely experience SpO2 < 92%.

According to various sources, from 5.7% to 20% of children with COVID-19 were hospitalized, the rest were treated on an outpatient basis, including patients with mild, subclinical and asymptomatic forms, in whom the SARS-CoV-2 virus was isolated during a contact examination. In the United States, only 1.6% to 2.5% of children with COVID-19 required hospitalization, and there was no need for resuscitation.

The severity of the clinical manifestations of coronavirus infection varies from the absence of symptoms (asymptomatic course) or mild respiratory symptoms to severe SARS, occurring with:

- high fever;
- a pronounced disturbance of well-being up to a violation of consciousness;
- chills, sweating;
- headaches and muscle pains;
- dry cough, shortness of breath, rapid and difficult breathing;
- palpitations.

The most common manifestation of SARS is bilateral viral pneumonia complicated by ARDS or pulmonary edema. Respiratory arrest is possible, which requires mechanical ventilation and assistance in the ICU.

---

[4] https://data.unicef.org/covid-19-and-children/
Source: The MPIDR COVerAGE database

Unfavorable outcomes develop with progressive respiratory failure, the addition of a secondary infection that occurs in the form of sepsis.

Possible complications:

- ARDS;
- acute heart failure;
- acute renal failure;
- septic shock;
- multiple organ failure (impaired functions of many organs and systems).

Not all children with suspected COVID-19 who had severe forms of the disease had laboratory-isolated SARS-CoV-2 virus, which does not allow us to exclude co-infections or the presence of other respiratory diseases in children with suspected cases of diseases based on clinical and epidemiological data.

Mild severity is characterized by an increase in body t not higher than 38.5 ° C, the absence of dyspnea at rest, but it may appear during physical exertion, SpO2 > 95%.

Moderate severity is characterized by an increase in body temperature above 38.5 ° C, the absence of dyspnea at rest, but its appearance during physical exertion (crying / crying), SpO2 < 95%.

Severe COVID-19 is characterized by dyspnea (feeling short of breath, chest tightness, shortness of breath or tachypnea), cyanosis/acrocyanosis, SpO2 <93%.

An extremely severe degree is recorded with the development of respiratory failure with the need for respiratory support, ARDS, shock, signs of multiple organ failure (encephalopathy, cardiovascular, renal, liver failure, DIC).

The frequency of severe and extremely severe cases of the disease does not exceed 1%. Cases of extremely severe COVID-19 in children are usually recorded in the presence of risk factors: severe premorbid diseases (children with lung diseases, congenital heart defects, bronchopulmonary dysplasia, Kawasaki disease, hydronephrosis, leukemia, etc.), immunodeficiency states of various genesis (more often children over 5 years of age; pneumonia is recorded 1.5 times more often), it is also impossible to exclude the effect on the severity of the state of coinfection with other respiratory viruses (respiratory syncytial virus, rhinovirus, bocavirus, adenovirus), which are characterized by damage to the lower respiratory tract (pneumonia , bronchiolitis).

Adverse events and allergic reactions to the vaccine

According to previous clinical studies of the studied vaccine and vaccines based on a similar platform of adenoviral vectors (vaccines against Ebola virus disease, EVD, registered in the Russian Federation, consisting of recombinant particles based on the vesicular stomatitis virus expressing the Ebola virus GP gene, and recombinant pseudoadenoviral particles expressing the Ebola virus GP gene), and those at the stage of clinical trials (against the Middle East respiratory syndrome coronavirus [Middle East Respiratory Syndrome, MERS-CoV], and against pandemic strains of influenza - recombinant pseudoadenoviral particles expressing the gene for the nucleoprotein protein and Influenza virus M2 protein A) These vaccines have a good safety profile. Allergic reactions of varying severity remain one of the most significant AE to these vaccines.

To mitigate this risk, patients with a aggravated allergic history will not be included in the study. The research center where vaccination will be carried out must have anti-shock therapy (in accordance with the Order of the Ministry of Health of the Russian Federation dated December 20, 2012 No. 1079n "On approval of the standard for emergency medical care for anaphylactic shock"), which is a prerequisite for using any parenteral biological preparations. After administration of the study drug, patients will be under medical supervision for 48 hours during the first phase of the study and at least 30 minutes during the second, which will minimize the risks to their health.

Pregnancy

In animal experiments of a similar vaccine (developed on the same platform of adenoviral vectors), it was proved that the vaccine does not adversely affect the embryonic development of rats in the antenatal and postnatal period of development when administered intramuscularly. The risks of this vaccine to the human fetus are currently unknown.

Preclinical studies data

1) **Study of generative toxicity**

Female rats: at the moment, three injections of the vaccine have been carried out in full at various doses (intramuscularly), once a week for 15 days (10^9 v.h., 10^{10} v.h., diluent). There were no differences in appearance or behavior between the diluent group and the vaccine groups. The females were then mated with intact males.

All females became pregnant, and pregnancy was recorded using vaginal swabs.

Half of the pregnant females were euthanized on the 20th day of pregnancy. Dead fetuses were not detected, the weight was determined in the fetuses and the craniocaudal size was measured; in the ovaries, the number of corpus luteum was counted; in the uterus, the number of sites of implantation and resorption. There were no abnormalities in the appearance of the fruit. No differences were observed between the diluent group and the vaccine groups.

The second half of the pregnant females were left until delivery. At the moment, almost all females have given birth. All newborn rat pups are alive, there are no anomalies in appearance and behavior.

Female rats: at the moment, seven injections of the vaccine have been carried out in full at various doses (intramuscularly), once a week for 48 days (10^9 vp, 10^{10} vp, diluent). There were no differences in appearance or behavior between the diluent group and the vaccine groups. At the moment (06/11/2021), males are mated with intact females.

2) **Study of embryotoxicity (antenatal and postnatal period)**

Intact females were mated with intact males, after which pregnancy was recorded using vaginal swabs.

Next, the females were carried out in full three injections of the vaccine at various doses (intramuscularly), once a week for 19 days (109 v.h., 1010 v.h., diluent). There were no differences in appearance or behavior between the diluent group and the vaccine groups.

Half of the pregnant females were euthanized on the 20th day of pregnancy. Dead fetuses were not detected, the weight was determined in the fetuses and the craniocaudal size was measured; in the ovaries, the number of corpus luteum was counted; in the uterus, the number of sites of implantation and resorption. There were no abnormalities in the appearance of the fruit. No differences were observed between the diluent group and the vaccine groups.

The second half of the pregnant females were left until delivery. All females have given birth so far. All newborn rat pups are alive, there are no anomalies in appearance and behavior.
Research on the reproductive toxicity of the COVID-19 vaccine is ongoing.
Impact on reproductive function
Potential risks for the reproductive health of the population during a pandemic include the impact on the reproductive system and its functioning, as well as the development of gametes and embryos of the following factors:

- Impact of the SARS-CoV-2 virus and COVID-19 disease;
- Influence of concomitant diseases;
- Medicines for the prevention and treatment of COVID-19;
- Chemical disinfectants;
- Psychological factors associated with panic during the COVID-19 outbreak;
- Reduced quality of life associated with economic factors;
- Decreased physical activity and quality of sexual life due to isolation.

It is not expected that there is a biologically plausible way that the vaccine could cause infertility in any woman or man, developmental pathology, or affect offspring, because:

- The vaccine does not use adjuvants;
- The potentially toxic (in rats) excipient (polysorbate 80) used in the vaccine is used at a dose that would not affect human fertility and reproductive function;
- The vaccine virus does not multiply, after administration, the virus delivers the S-protein gene into the cell and ceases to exist in the human body;
- The gene encoding the S-protein in the body leads to the production of the viral S-protein and the development of an immune response to it;
- The anti-S protein antibodies produced in response to immunization are similar to those produced in response to the disease caused by SARS-CoV-2, therefore the risk of immunization is no greater than that of infection;
- Adenovirus antibodies produced in response to immunization are similar to adenovirus antibodies produced in response to disease caused by adenovirus, a common pathogen, and therefore the risk of immunization is no greater than that of infection.

In preclinical studies of reproductive toxicity, a similar vaccine was studied, developed on the platform of adenoviral vectors 26 and 5 of identical composition. At the moment, studies of the Gam-Kovid-Vak vaccine are underway, according to preliminary data, toxicity has not been identified.

No increase in risk is expected with the use of the vaccine in study participants under the conditions of compliance with the restrictions indicated in the instructions for medical use.

Lactation

There are no data on the excretion of the drug into the milk of lactating women and the risks associated with possible excretion for infants are unknown. Therefore, lactating subjects will not be included in the study to minimize all possible risks.

Venipuncture

During this procedure, there may be mild pain that is transient and / or a hematoma may form in the area where the needle is inserted. Much less often, a blood clot can form in a vein or an infectious complication develops. Dizziness and/or weakness may occur during or shortly after blood sampling. These risks will be minimized, taking into account the involvement of qualified medical personnel in the implementation of this procedure.

In total, blood sampling for research will be performed: from participants - 10 times during the entire participation in the 1st phase of the study and about 120.4 ml of blood will be taken and 59.1 ml in the 2nd phase (Table 3).

Table 3 Approximate volume of blood samples required for testing

1 stage

**Visit, day**	**Visit 1 (d. -7… 0)**	**Visit 2 (d. 1)**	**Visit 2 (d. 2)**	**Visit 4 (d. 20)**	**Visit 4 (d. 21)**	**Visit4 (d. 22)**	**Visit5 (d. 28)**	**Visit6 (d. 42)**	**Visit7 (d. 90)**	**Visit 8 (d. 180)**	**Total**
Blood volume	18.3	17.3	7.3	9.8	7.3	4.9	20.9	14.8	9.9	9.9	120.4

2 stage for volunteers randomized to the vaccine group and donating blood for immunogenicity testing

**Visit, day**	**Visit 1 (day -7… 0)**	**Visit 2 (day 1)**	**Visit 4 (day 28 ± 1)**	**Visit 5 (day 42 ± 3)**	**Visit 6 (day 90 ± 7)**	**Visit 7 (day 180 ± 14)**	**Total**
Blood volume	18.3	7.5	13.4	7.5	4.9	7.5	59.1

2 stage for volunteers randomized to the vaccine group

**Visit, day**	**Visit 1 (day -7… 0)**	**Visit 2 (day 1)**	**Visit 4 (day 28 ± 1)**
Blood volume	18.3	7.5	13.4

2 stage for volunteers randomized to the placebo group

<table>
<tr><td>Visit, day</td><td>Visit 1
(day -7… 0)</td><td>Visit 2
(day 1)</td><td>Visit 4
(day 28 ± 1)</td><td colspan="4" rowspan="2">Blinding, vaccination and start of observation from the moment of vaccination</td></tr>
<tr><td>Blood volume</td><td>18.3</td><td>7.5</td><td>13.4</td></tr>
<tr><td>Visit, day</td><td></td><td>Visit 2
(day 1)</td><td>Visit 4
(day 28 ± 1)</td><td></td><td></td><td></td><td></td></tr>
<tr><td>Blood volume</td><td></td><td>7.5</td><td>13.4</td><td></td><td></td><td></td><td></td></tr>
</table>

Blood sampling in planned quantities is safe for study participants[5,6].

The maximum volume of blood for a single collection depending on the patient's weight	
**Body weight, kg**	**Max. amount of blood, ml**
20.9–22.7	20
23.2–25.0	20
25.5–27.3	20
27.7–29.5	25
30.0–31.8	30
32.3–34.1	30
34.5–36.4	30
36.8–38.6	30
39.1–40.9	30
41.4–43.2	30
43.6–45.5	30

**Age, years**	**Girls**		**Boys**	
	**Body weight, kg**	**Height, cm**	**Body weight, kg**	**Height, cm**
**12**	44.05 ± 7.48	152.81 ± 7.01	41.25 ± 7.40	150.05 ± 6.40
**13**	48.70 ± 9.16	156.85 ± 6.20	45.85 ± 8.26	156.65 ± 8.00
**14**	51.32 ± 7.30	160.86 ± 6.36	51.18 ± 7.34	162.62 ± 7.34
**15**	56.65 ± 9.85	161.80 ± 7.40	56.50 ± 13.50	168.10 ± 9.50
**16**	58.00 ± 9.60	162.70 ± 7.50	62.40 ± 14.10	172.60 ± 9.40
**17**	58.60 ± 9.40	163.10 ± 7.30	67.35 ± 12.75	176.30 ± 9.70

Since the study will involve volunteers over 12 years old, whose body weight according to the average body weight tables exceeds 30 kg, simultaneous blood sampling in the quantities indicated above does not pose a risk to the health of volunteers.

Unknown risks

In addition to the risks and discomfort listed above, previously unreported or atypical effects may occur. AE that may occur during the first use of an investigational drug in humans cannot be foreseen in advance. Therefore, the possibility of developing an unforeseen SAE cannot be ruled out. At the first stage, vaccination will be carried out at the hospital, the volunteer will be under observation for 48 hours after the administration of the drug. At the second stage, after the administration of the drug, volunteers will be under the supervision of a doctor for 30-60 minutes after the administration of the drug, which will minimize the risks and provide qualified medical care as quickly as possible in the event of an AE.

Medical conditions that develop during the conduct of a clinical study may lead to termination of a volunteer's participation in a clinical study if medically necessary or if the volunteer desires so.

---

[5] Howie SR. Blood sample volumes in child health research: review of safe limits. *Bull World Health Organ*. 2011;89(1):46-53. doi:10.2471/BLT.10.080010

[6] Blood sampling for laboratory research in pediatric practice. Laboratory diagnosis. Special issue LABORATORY No. 3, 2013

Volunteers withdrawn from a clinical trial due to an AE should receive appropriate treatment in accordance with current clinical standards and be followed until they are cured or stabilized, or until an acceptable explanation for the event is identified.

There are no known environmental risks. All biohazard waste will be disposed of in accordance with government regulations.

Taking into account the above information, we can conclude that the benefits of participating in this study outweigh the potential risk to its participants.

2.6 Description and rationale for the route of administration, dosage, dosage regimen and course of treatment

An important feature of the combined vector vaccine "Gam-COVID-Vac" is that it is based on the approach of heterologous prime-boost vaccination, when different recombinant viral vectors are used for priming and boosting the immune response: component 1 of the vaccine includes adenovirus-based recombinant particles human serotype 26, and component 2 of the vaccine includes recombinant particles based on human adenovirus serotype 5. This approach allows, firstly, to form a long-term protective immune response, and secondly, to avoid the influence of the immune response on the vector that develops after the first vaccination on the effectiveness of the second vaccination.

Studies in adults have shown the safety and efficacy of the vaccine at a dose of 1011 viral particles. The literature presents data on the study in animal models of the effect of various doses of adenovirus vaccines dose correlation with weight and immune response. Adenovirus vectors are extremely effective in inducing immunity. It should be noted that the change in antibody titer is non-linearly dependent on the administered dose. So, changing the dose by an order of magnitude does not lead to a corresponding change in the level of antibodies and cellular response, there are limits to the correlation between dose and response.

This has been shown by the developer in studies of vaccines on the adenoviral platform, as well as in publications of other developers and scientific groups.

Below are our own data on the dependence of the immune response on the dose.

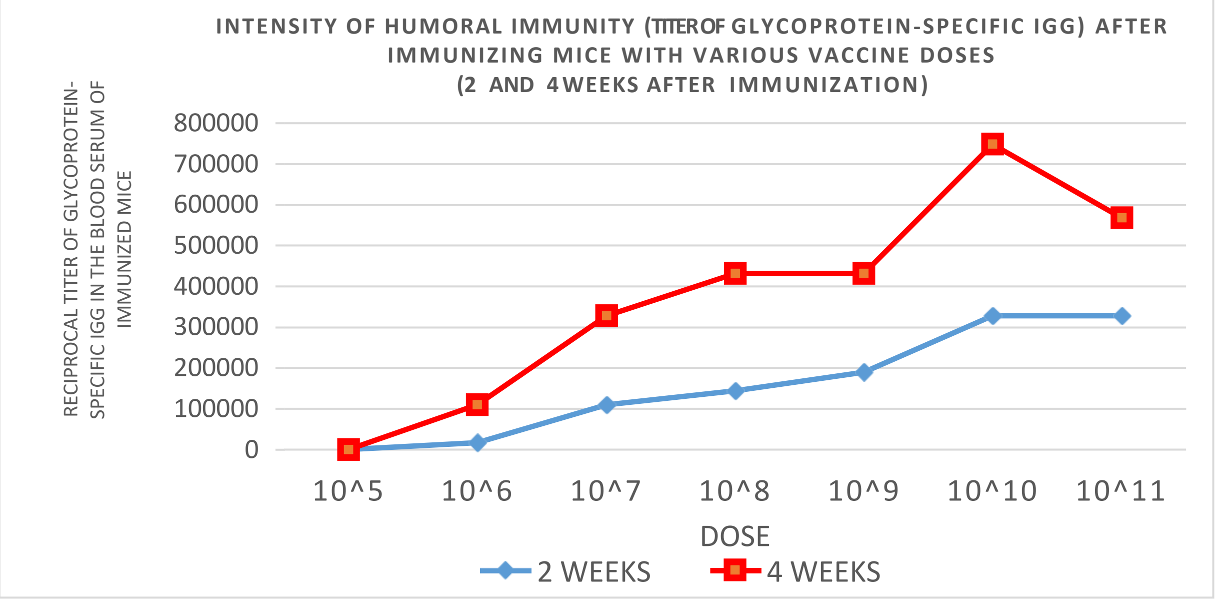

Reducing the dose by an order of magnitude should not lead to a pronounced decrease in the immune response, but it is expected to reduce the number of adverse reactions. If the dose is found to be safe, a switch to 1/5 of the adult immunization dose will be made, as the higher dose will allow faster rise in antibody titers and provide protection in a shorter time frame.
In this regard, in this study it is planned to study 1/10 and 1/5 of the full immunizing dose.

2.7 Description of the study population

About 3,100 volunteers will be randomized in the study, 2,500 volunteers will receive the study drug, 600 volunteers will receive a placebo. The study will include volunteers of both sexes aged 12–17 years inclusive, who meet the inclusion criteria (Section 5.1) and who do not meet the exclusion criteria (Section 5.2).
At the first stage of dose selection, each dosing group (1/10 and 1/5 immunizing dose for adults) will include no more than 50 people who will receive the drug in a hospital setting.
Group 1 - dose 1/10 after an interim safety assessment on day 7, a transition to the second dosing group 1/5 is planned.
Based on the results of the assessment of safety and immunogenicity indicators on the 28th day of the study, a report will be prepared and a dose will be selected.
The second stage is a randomized, double-blind, placebo-controlled trial with stratification by age into two groups of 12-14 and 15-17 years old - volunteers will receive the vaccine at the selected dose (about 2400 people) or placebo (about 600 people). The second stage will be carried out on an outpatient basis.

2.8 Rationale for the clinical study

Coronaviruses (*Coronaviridae*) are a large family of RNA viruses that can infect humans and some animals. In humans, coronaviruses can cause a range of illnesses, from mild acute respiratory infections to severe acute respiratory syndrome (SARS). Currently, four coronaviruses (HCoV-229E, -OC43, -NL63 and -HKU1) are known to circulate among the population, which are present all year round in the structure of SARS, and, as a rule, cause mild to moderate damage to the upper respiratory tract.
According to the results of serological and phylogenetic analysis, coronaviruses are divided into four genera: *Alphacoronavirus*, *Betacoronavirus*, *Gammacoronavirus*, and *Deltacoronavirus*. The natural hosts of most of the currently known coronaviruses are mammals.
Until 2002, coronaviruses were considered as pathogens that cause mild upper respiratory infections (with extremely rare fatal outcomes). In late 2002, the coronavirus (SARS-CoV), the causative agent of SARS, emerged that caused SARS in humans. This virus belongs to the genus *Betacoronavirus*. The natural reservoir of Coronavirus severe acute respiratory syndrome (SARS-CoV) coronavirus is bats, and intermediate hosts are camels and Himalayan civets. In total, more than 8,000 cases were registered in 37 countries around the world during the epidemic period, of which 774 were fatal. Since 2004, no new cases of SARS caused by SARS-CoV have been reported.

In 2012, the world faced the coronavirus MERS-CoV, the causative agent of the Middle East respiratory syndrome, also belonging to the genus *Betacoronavirus*. The main natural reservoir of MERS-CoV coronaviruses are one-humped camels (dromedaries). From 2012 to January 31, 2020, 2519 cases of coronavirus infection caused by the MERS-CoV virus were registered, of which 866 were fatal. All cases are geographically associated with the Arabian Peninsula (82% of cases have been reported in Saudi Arabia). Currently, MERS-CoV continues to circulate and cause new cases of the disease.

On December 31, 2019, the World Health Organization (WHO) received notification of a cluster of cases of pneumonia of unknown etiology in Wuhan, Hubei Province, China. On January 7, 2020, the Chinese authorities determined that the causative agent of this disease is a coronavirus related to SARS-CoV, and on February 11, 2020, WHO assigned the name to the new disease "coronavirus infection COVID-19". (“Coronavirus disease 2019”). On February 11, 2020, the International Committee on the Taxonomy of Viruses assigned the official name to the infectious agent - SARS-CoV-2 [7].

The new SARS-CoV-2 coronavirus is a single-stranded RNA-containing virus, belongs to the *Coronaviridae* family, belongs to the Beta-CoV B line. The virus is assigned to pathogenicity group II, like some other representatives of this family (SARS-CoV virus, MERS- CoV).

The entrance gate of the pathogen is the epithelium of the upper respiratory tract and epithelial cells of the stomach and intestines. The initial stage of infection is the penetration of SARS-CoV-2 into target cells that have angiotensin-converting enzyme type II receptors (angiotensin-converting enzyme 2, ACE2). ACE2 receptors are present on the cells of the respiratory tract, kidneys, esophagus, bladder, ileum, heart, and central nervous system. However, the main and quickly achievable target is type II alveolar cells (AT2) of the lungs, which determines the development of pneumonia. The role of CD147 in SARS-CoV-2 cell invasion is also discussed.

It has been established that the dissemination of SARS-CoV-2 from the systemic circulation or through the plate of the cribriform bone (*Lamina cribrosa*) can lead to brain damage.

The most common symptoms of COVID-19 include fever, fatigue and dry cough. Some patients may experience various pains, nasal congestion, runny nose, pharyngitis or diarrhea. As a rule, these symptoms develop gradually and are mild. Some infected individuals do not develop any symptoms or feel unwell. In most people (about 80%), the disease ends in recovery, while specific therapeutic measures are not required. In about one in six cases of COVID-19, severe symptoms develop with respiratory failure. Older people, as well as people with existing physical diseases, such as arterial hypertension, heart disease or diabetes, are more likely to have a severe course of the disease. According to most estimates, the incubation period for COVID-19 ranges from 1 to 14 days, and is most often around five days.

You can get infected from other people if they are infected with the virus. The disease can be spread from person to person through small droplets released from the nose or mouth of a person with COVID-19 when coughing or sneezing.

Since the end of January 2020, cases of COVID-19 have been reported in many countries around the world, mainly associated with travel to China. At the end of February 2020, the COVID-19 epidemiological situation in South Korea, Iran and Italy worsened sharply, which subsequently led to a significant increase in the number of cases in other countries of the world associated with travel to these countries. On March 11, 2020, the WHO declared the spread of COVID-19 a pandemic.

While children and adolescents generally have a better prognosis for COVID-19 than adults, severe illness can also develop in this age group, especially in those with underlying medical conditions. Adolescents may play an important role in the transmission of SARS-CoV-2. Thus, their vaccination can prevent the disease and promote the formation of herd immunity. The pandemic interrupted the education and social development of students, activity decreased, which entails significant problems with education, health and adaptation in society. Therefore, safe and effective

vaccines for the young population are of paramount importance.

At the end of July 2020, the Pfizer-BioNTech Phase 3 clinical trial began, with participants aged 12 and over. About three thousand participants were teenagers: 2,259 participants were between the ages of 12 and 15, and 754 participants were between the ages of 16 and 17. In March 2021, Pfizer and BioNTech dosed the first healthy children as part of a Phase 1/2/3 global continuous trial to see if the vaccine could elicit an immune response against COVID-19 and, if safe, in children aged 6 and over. months to 11 years. BNT162b2 vaccination against COVID-19 is now widely used in the 16+ age group - BioNTech and Pfizer received December 11, 2020 BNT162b2 received emergency use approval from the US Food and Drug Administration for the prevention of Covid-19 in persons 16 years of age and older. On May 10, 2021, the emergency use authorization was expanded to include persons 12 years of age. Clinical studies are ongoing in children over 6 months of age.

In the Russian Federation, registered vaccines are allowed for persons aged 18 and over. There are no drugs to prevent COVID-19 in children and adolescents. The need for immunization of younger age groups is a sufficient rationale for this study.

Adolescents and children are susceptible to coronavirus infection, and although the severity of the disease in children is usually lower than in adults, children and adolescents of all ages are one of the key players in the spread of coronavirus, and therefore they need to be given due attention. Since even asymptomatic children are a source of infection for surrounding adults, and their regular stay in organized crowded groups (universities, colleges, schools, etc.) increases the risk of transmission of infection to surrounding adults. What is a significant factor in the spread of infection. In addition, there is an increase in the incidence among children, an increase in the number of severe cases, especially among adolescents.

That is why the issue of the most prompt start of vaccination of children (aged 18 and younger) against coronavirus infection is of significant economic and social importance.

Use of the experimental drug in humans

In total, based on the data of the NICEM them. N.F. Gamalei of the Ministry of Health of Russia developed 9 vaccines, including three vaccines for the prevention of coronavirus infection (Gam-COVID-Vak, Gam-COVID-Vac-Lio, Sputnik-Light), three vaccines for the prevention of Ebola fever, registered in Russia in 2015 –2019 and 2 experimental vaccines against Middle East respiratory syndrome (MERS) and pandemic influenza.

The vaccine "Gam-COVID-Vak", created by the Federal State Budgetary Institution "National Research Center for Epidemiology and Microbiology" N.F. Gamalei” of the Ministry of Health of Russia, based on recombinant adenoviral vectors, forms both humoral and cellular immunity to the COVID-19 pathogen.

Details of the results of previous clinical trials are available in the Investigator's Brochure.

The list of vaccines developed by the Federal State Budgetary Institution "National Research Center for Epidemiology and Microbiology" N.F. Gamaleya" of the Ministry of Health of Russia on the platform of adenoviral vectors is presented in Table 4 below.

Table 4 Vaccines developed by the FGBU “National Research Center for Epidemiology N.F. Gamaleya" of the Ministry of Health of Russia on the platform of adenoviral vectors

Drug	Pharmaceutical form	Number of subjects	Doses studied in the CS	Current status
GamEvac vector vaccine against Ebola virus	solution for intramuscular injections 0.5 ml/dose 0.5 ml × 2	92 healthy volunteers	½ of the estimated immunizing dose and full vaccine dose	Registered LP-003389 28.12.2015
GamEvac-Combi combined vector vaccine against Ebola virus	solution for intramuscular injections, dose/0.5 ml	10 healthy volunteers (Russia) 2,000 healthy volunteers (Guinea)	½ of the estimated immunizing dose and full vaccine dose	Registered LP-003390 28.12.2015 NCT03072030
GamEvac-Lio combined vector vaccine against Ebola virus	lyophilizate for preparing solution for intramuscular injections [component A (vial)(1 dose) + component B (vial) (1 dose)]	260 healthy volunteers	full vaccine dose	Registered LP-006175 17.04.2020
GamEvac (vector vaccine against Ebola virus)	solution for intramuscular injections 0.5 ml/dose	60 healthy volunteers	½ of the	RCT No. 435 (24.08.2018) CS, IV

			estimated immunizing dose and full vaccine dose	
GamFluVac (Vector vaccine for prevention of influenza A (recombinant))	Nasal drops dose/0.5 ml	60 healthy volunteers	½ of the estimated immunizing dose and full vaccine dose	RCT No. 393 (22.07.2019) CS, II
GamFluVac (Vector vaccine for prevention of influenza A (recombinant)	Nasal drops dose/0.5 ml	300 healthy volunteers	full vaccine dose	RCT No. 393 (22.07.2019) CS, II

BVRS-GamVak<br><br>(vector vaccine for prevention of Middle East respiratory syndrome)	lyophilizate for preparing solution for intramuscular injections	194 healthy<br><br>volunteers	½ of estimated immunizing dose and full vaccine dose	RCT No. 506 (09.09.2019)<br><br>CS, I–II
BVRS-GamVak-Combi<br><br>(combined vector vaccine for prevention of Middle East respiratory syndrome)	lyophilizate for preparing solution for intramuscular injections	291 healthy<br><br>volunteers	½ of estimated immunizing dose and full vaccine dose	RCT No. 507 (09.09.2019)<br><br>CS, I–II
Gam-COVID-Vac combined vector vaccine for the prevention of coronavirus infection caused by SARS-CoV-2 virus	solution for<br><br>intramuscular injections	43 healthy<br><br>volunteers	full vaccine dose	RCT No. 241 (16.06.2020)<br><br>The drug is registered<br><br>11.08.2020 (LP-006395)
Gam-COVID-Vac-Lio combined vector vaccine for the prevention of coronavirus infection caused by SARS-CoV-2 virus	lyophilizate for preparing solution for intramuscular injections	43 healthy<br><br>volunteers	full vaccine dose	RCT No. 242 (16.06.2020)<br><br>The drug is registered<br><br>25.08.2020 (LP_006423)
Gam-COVID-Vac combined vector vaccine for the prevention of coronavirus infection caused by SARS-CoV-2 virus	solution for<br><br>intramuscular<br><br>injections	40,000<br><br>volunteers (Russia)<br><br>100 volunteers (Belarus)	full vaccine dose	Russia: RCT No. 450<br><br>(25.08.2020)<br><br>is currently ongoing<br>Belarus: Order of the Ministry of Health of the

				Republic of Belarus dated 21.09.2020 No. 954
Gam-COVID-Vac combined vector vaccine for the prevention of coronavirus infection caused by SARS-CoV-2 virus	solution for intramuscular injections	150 volunteers older 60	full vaccine dose	RCT No. 566 (13.10.2020)
Sputnik light vector vaccine for prevention of coronavirus infection caused by SARS-CoV-2 virus	solution for intramuscular injections	150 healthy volunteers	full vaccine dose	RCT No. 1 (08.01.2021)
Sputnik light vector vaccine for prevention of coronavirus infection caused by SARS-CoV-2 virus	solution for intramuscular injections	4,000 volunteers older 18	full vaccine dose	RCT No. 90 (17.02.2021)

The study is being conducted with the aim of developing a safe and effective drug for the prevention of SARS CoV and expanding indications for vaccination.

2.9 Regulatory basis for conducting a clinical trial

This document is a clinical trial protocol, which is planned to be conducted in accordance with the principles of the WMA Declaration of Helsinki (adopted at the 18th Assembly of the WMA in Helsinki in June 1964, the latest version was approved at the 64th Assembly in Fortaleza in October 2013), by a tripartite agreement according to GCP adopted by the International Council for Harmonization of Technical Requirements for Pharmaceuticals for Human Use (ICH) E6(R2) dated 09.11.2016 and is regulated by the current legislation of the EAEU and RF:

- Rules for the registration and examination of medicinal products for medical use (approved by the Decision of the Council of the Eurasian Economic Commission dated November 3, 2016 No. 78);
- Decree of the Government of the Russian Federation of April 3, 2020 No. 441 "On the peculiarities of the circulation of medicinal products for medical use, which are intended for use in conditions of the threat of the emergence, occurrence and liquidation of an emergency and for organizing the provision of medical care to persons affected by emergency situations, prevention of emergency situations, prevention and treatment of diseases that pose a danger to others, diseases and injuries resulting from exposure to adverse chemical, biological, radiation factors"
- Rules of good clinical practice of the EAEU (approved by the Decision of the Council of the Eurasian Economic Commission dated November 3, 2016 No. 79);
- Federal Law No. 61-FZ of April 12, 2010 “On the Circulation of Medicines” (as amended);
- Federal Law No. 152-FZ of July 27, 2006 “On Personal Data” (as amended);
- Order of the Ministry of Health of the Russian Federation dated April 1, 2016 No. 200n “On Approval of the Rules of Good Clinical Practice”;
- National Standard of the Russian Federation GOST R52379-2005 "Good Clinical Practice";
- Decree of the Government of the Russian Federation No. 714 dated September 13, 2010 (as amended) “On Approval of Standard Rules for Compulsory Life and Health Insurance of a Patient Participating in Clinical Trials of a Medicinal Product”;
- Order of the Ministry of Health of the Russian Federation dated November 29, 2012 No. 986n “On Approval of the Regulations on the Ethics Council”;
- Order of the Ministry of Health of the Russian Federation dated October 30, 2017 No. 878 “On the Composition of the Ethics Council”.
- Order of the Ministry of Health of the Russian Federation of May 7, 2018 No. 202n “On approval of the Procedure for posting information about the composition of the Ethics Council, its work plans and current activities on the official website of the Ministry of Health of the Russian Federation in the Internet information and telecommunication network”.

2.9.1 Responsibility of the investigator

A prerequisite for assuming responsibility for the proper conduct of a clinical investigation in accordance with applicable regulatory requirements is that the investigator has relevant education, training and experience. The researcher's qualifications must be proven (autobiography to date).

The investigator is obliged to:

- be thoroughly familiar with the use of the investigational product as described in the Protocol, Investigator's Brochure;
- know and comply with the Good Clinical Practice Guidelines and applicable regulatory requirements;
- allow Sponsor to conduct monitoring and auditing, and allow inspections by relevant regulatory authorities;
- maintain a list of qualified personnel to whom he has delegated significant responsibilities within the clinical investigation;
- be able to demonstrate the ability to recruit the required number of volunteers meeting inclusion/non-inclusion criteria within an agreed recruitment period;
- have sufficient time to properly conduct and complete the study within the agreed study period;
- have available qualified personnel in sufficient numbers and adequate equipment for the intended duration of the study for the proper conduct of the study and safety;
- ensure that all research assistants are adequately informed about the Protocol, the investigational product and their responsibilities and roles related to the conduct of research;
- conduct research in accordance with the Protocol, he confirms his consent to this by signing the Protocol;
- document and explain any deviation from the approved Protocol.

During the study and after the completion of the subject's participation in the study, the investigator (health care facility) must ensure that the study subject receives the necessary medical care in the event of an AE, including clinically significant changes in laboratory parameters. The researcher (medical institution) is obliged to inform the research subject about intercurrent diseases that have become known to the researcher and require medical attention.

The Investigator shall not resort to any deviation or modification of the Protocol without the consent of the Sponsor and prior to the review and documented approval/favorable opinion of the IEC/ESA of an amendment to the Protocol, unless there is an urgent need to immediately reduce the risk to participants in a clinical trial or when the changes concern only logistical or administrative aspects of the study (for example, changing the monitor, phone number).

2.9.2 Medical care provided to volunteers in a clinical trial

The qualified investigator should be responsible for making all medical decisions within the study.

The person responsible for making medical decisions in each clinical center is the Principal Investigator.

During and after a volunteer's participation in a study, it is the investigator's responsibility to ensure that adequate medical care is provided to the volunteer in the event of an AE associated with the investigational drug. The investigator must inform the volunteers of the medical care required to treat the comorbid disease(s) of which the investigator has become aware.

A volunteer receives medical assistance within the framework of compulsory health insurance in accordance with the general rules.

The investigator is advised to inform the volunteer that he/she would like to inform his/her attending physician about participation in the clinical trial, if the volunteer has one.

If the volunteer has a voluntary health insurance policy, the volunteer should be advised to read the terms and conditions carefully before being included in the study. Participation in a clinical trial may disqualify a volunteer from receiving medical care under VHI.

Although a volunteer is not required to provide reasons for withdrawing from a study prematurely, the researcher should make reasonable efforts to identify the reasons, in full respect of their rights.

3 Aims and objectives of the clinical trial

The study aim:

To assess the immune response and the safety, tolerability, and immunogenicity profile of the COVID-19 vaccine in adolescents 12–17 years of age.

Research objectives and endpoints:

1. *To study the safety of the drug in healthy volunteers 12–17 years old inclusive by sequential escalation of 2 doses: 1/10 and 1/5 of the immunizing dose.*

During the entire observation period, the effect of the drug on vital (systolic and diastolic blood pressure, heart rate, body temperature) and laboratory parameters in healthy volunteers will be studied, as well as on the presence of general and local post-vaccination reactions compared with background values before drug administration.

A planned transition to the next dose is allowed based on the interim report on safety and tolerability on the 7th day after immunization.

In the event of severe general and local post-vaccination reactions or AE in the group, the study in the group is immediately stopped. The decision to stop a study is made by the Principal Investigators and the Sponsor. The safe adjusted dose will be the dose used in the previous study group.

2. *Determine a safe dose for healthy volunteers 12–17 years old and proceed to the second stage of the study.*
3. *Describe the state of tension of the cellular immune response when using "Gam-COVID-Vac M Combined vector vaccine for the prevention of coronavirus infection caused by the SARS-CoV-2 virus (on the day of the drug administration before receiving the first dose of the vaccine and 28 days later, according to the concentration of interferon gamma in T-lymphocyte culture after restimulation with SARS-CoV-2 glycoprotein)*
4. *Describe the state of the antigen-specific cellular immune response when using the "Gam-COVID-Vac M Combined vector vaccine for the prevention of coronavirus infection caused by the SARS-CoV-2 virus",*
5. *To study the immunogenicity of the selected dose of the drug in healthy volunteers:*
   - *The study of immunogenicity by the level of antibodies against RBD will be carried out on days 21, 28, 42, 90 and 180 after immunization with the first component by determining the titer of specific antibodies by ELISA compared with background values (day 1)*
   - *The study of virus neutralizing activity will be carried out on days 21, 28, 42, 90 and 180 after immunization with the first component by determining the titer of specific antibodies by ELISA compared with background values (day 1)*

6. *Assess the immunogenicity of "Gam-COVID-Vac M Combined vector vaccine for the prevention of coronavirus infection caused by the SARS-CoV-2 virus" in comparison with placebo, based on the geometric mean of the titer of SARS-CoV-2 glycoprotein-specific antibodies (in day of dosing prior to first dose of ED/placebo and 28 days later)*
7. *To evaluate the safety of using "Gam-COVID-Vak M Combined vector vaccine for the prevention of coronavirus infection caused by the SARS-CoV-2 virus" in comparison with placebo during the period after receiving the first dose of ED/placebo*
8. *To study the safety performance of the drug in healthy volunteers aged 12–17 years up to 365 days after vaccination*
9. *Compare immunogenicity and safety scores in adolescents with safety and immunogenicity scores in young adults aged 18-21 years in clinical trial #04-Gam-COVID-Wak-2020 A randomized, double-blind, placebo-controlled, multicenter clinical trial of the efficacy, immunogenicity, and safety of the combined Gam-COVID-Vac vector vaccine in parallel groups in the prevention of coronavirus infection caused by the SARS-CoV-2 virus" as the group closest to the study group in terms of selection criteria*
10. *Assess the vaccine’s protective effect between days 28 and 365 of the study (describe cases of COVID-19, cases of severe COVID-19 and death)*

4 Description of the overall plan (design) of the clinical trial

Double-blind, placebo-controlled, open-label, prospective clinical trial in healthy volunteers aged 12–17 years, stratified by age into two groups of 12–14 and 15–17 years.

The study includes pre-screening outside the clinical study (examination and selection of volunteers for participation in the study, formation of a waiting list), screening (inclusion in the study of volunteers that meet the selection criteria) and the study itself - 2-fold administration of the drug with an interval of 21 days and follow-up up to a year from first administration of the vaccine.

4.1 Main and additional parameters of interest

Immunogenicity assessment:

The following indicators of immunogenicity will be assessed in volunteers:

a) Indicators of the intensity of the humoral immune response:
- Geometric mean titer of IgG antibodies against SARS-CoV-2 spike protein;
- Percentage of volunteers with detected specific IgGs;
- Geometric mean titer of neutralizing antibodies against the SARS-CoV-2 virus;
- Percentage of volunteers with detected neutralizing antibodies.

b) Indicators of the intensity of the cellular immune response:
- An increase in the concentration of interferon gamma in a culture of T-lymphocytes after restimulation with a specific SARS-CoV-2 antigen (glycoprotein S or RBD);
- Level of proliferative activity of CD4+ and CD8+ cells after restimulation with SARS-CoV-2 specific antigen (glycoprotein S or RBD).

Immunogenicity parameters can be compared with those of healthy volunteers in the Gam-COVID-Vak clinical trials at the same time.

Basic security settings:

The duration of participation of volunteers in the study will be 53 weeks, including 1 week of screening, 3 weeks of therapy and 49 weeks of follow-up. For placebo volunteers, the study will be extended by 28 days to unblind and the period of unblind procedures by a total of no more than 2 months.

The safety assessment will be based on the registration of AE within 42 days and SAE during the entire follow-up period.

In order to prevent the development of post-vaccination reactions and complications, volunteers are screened to determine the initial indicators of the state of the body. In addition, volunteers will undergo a medical examination. All baseline indicators (examinations before drug administration) are entered into the electronic individual registration card (eIRC) of volunteers.

Throughout the vaccination period and during the follow-up period, safety information will be collected on the following parameters:

- development, severity and relationship with administration of the two components of the drug Gam-COVID-Vak M Combined vector vaccine for the prevention of coronavirus infection caused by the SARS-CoV-2 virus of all SAE during the entire study;
- development, severity and relationship with administration of the two components of the drug Gam-COVID-Vac M Combined vector vaccine for the prevention of coronavirus infection caused by the SARS-CoV-2 virus of all AE during 42 days of the study
- the frequency and severity of local signs and symptoms, the relationship with the use of the drug of general signs and symptoms after drug administration;
- data of physical, laboratory and instrumental studies;
- reactogenic properties are assessed in terms of local (area of the injection site) and general (systemic reactions of the body to the administration of the drug) reactions.

Local reactions are evaluated on the following grounds:

- hyperemia at the injection site;
- edema (infiltration);
- pain
- increase in regional lymph nodes;
- rash;
- itching.

Common post-vaccination reactions include: fever, feeling unwell, headache, dizziness, decreased appetite, insomnia, nausea, vomiting, dyspepsia, weakness, sweating, muscle and joint pain, abdominal pain, convulsions, etc. In addition, allergic reactions are possible. reactions.

Accounting for local and general reactions is carried out by the researcher on the basis of body temperature measurement, examination and questioning of the volunteer, conducted in accordance with the schedule of visits and research procedures.

Changes in laboratory parameters (clinical and biochemical blood tests), changes in vital signs (blood pressure [BP], heart rate [HR], respiratory rate [RR], body temperature) will also be assessed.

When assessing, it should be borne in mind that there are no pathognomonic symptoms that would unambiguously consider each specific case of AE associated with vaccination. Such clinical symptoms as high fever, intoxication, neurological symptoms, various types of allergic reactions, including the immediate type, may not be due to immunization, but to a disease that coincided with the vaccination. Therefore, each case of the disease that developed in the post-vaccination period requires careful differential diagnosis with both infectious and non-infectious diseases using instrumental and laboratory research methods, based on the clinical symptoms of the disease.

One of the criteria is the time of occurrence. As a rule, the symptoms of post-vaccination reactions develop no later than 48 hours, while the duration of fever for more than 72 hours or its occurrence after this time indicates the possible presence (attachment) of infection.

4.2 Study design

4.2.1 Study type

This clinical trial is a double-blind, placebo-controlled, prospective, open-label, titration clinical trial in healthy volunteers aged 12–17 years.

This study will include 3000 healthy volunteers aged 12–17 years: The clinical study will consist of two stages:

1. dose selection
2. study of the selected dose

4.2.2 Content of visits

4.2.2.1 1 stage (phase I–II)

Two dose groups will include 100 volunteers (50 per group), screened (no more than 7 days prior to enrollment in the study) should have a sufficient number of volunteers to ensure the required number of volunteers who have completed screening procedures7.

The conditions for the study are the selection of doses (administration of the drug and follow-up for 48 hours) at the hospital, the rest of the visits on an outpatient basis.

It is planned to start the study with a dose of 1/10 of the full dose for adults.

Volunteers will be hospitalized the day before vaccination. It is allowed to conduct screening procedures the day before vaccination at the hospital, subject to prior approval of the volunteer based on the results of prescreening.

The first dose will be administered on day 1. After that, the volunteer will be observed for 48 hours. The subject will be asked to rate the pain of the injection using the VAS for pain (see Appendix 1) immediately after injection, 2, 12 and 48 hours after injection.

On days 3 and 7, a telephone consultation will be held. With a favorable safety profile, after 7 days after the full dose, the Principal Investigators make a consolidated decision to escalate the dose to 1/5 of the adult dose, informing the Sponsor by letter.

Diet standardization, restrictions.

The administration of the study drug is carried out on an empty stomach - the volunteer does not eat 10 hours before the administration of the study drug.

The volunteer should refrain from intense physical activity and contact sports 24 hours before and for a week after the administration of the drug.

The volunteer must not take any medications (including herbal preparations and nutritional supplements) without the consent of the research physician.

Volunteers are required to use adequate contraception throughout their participation in the study.

Visit 1 (day -7 – -1/0): screening

[7] Prior to the start of the study, in order to form a base of volunteers planning to participate in the study, it is allowed to organize a pre-screening procedure. Prescreening involves a routine examination 2-3 weeks before the administration of the drug.

The screening period in the study begins from the moment the informed consent is signed and lasts no more than 7 days (from -7 to -1/0 days) until the volunteer is included in the study. Conducting any procedures related to the study to the subject of the study is not allowed until the signing of the IS form. At the same time, each subject should have sufficient time to familiarize himself with the text of consent in order to give informed and voluntary consent to participate in the study. The date and time of signing the IS must be included in the primary medical documentation and CRF.

During Visit 1, volunteers will be assessed for meeting the eligibility criteria and not eligibility criteria. Volunteers potentially eligible to participate in a clinical trial will be invited to participate.

During the screening visit, the following procedures will be performed:

- Signing by the subject and his parent/adoptive parent of the IP form for participation in the study;
- Collection of anamnesis, demographic and anthropometric data: collection of data on concomitant and previously transferred diseases, concomitant therapy, collection of information on the presence of allergies, on the contacts of the subject of the study on contact with patients with COVID-19, on previous vaccinations / immunizations, on blood donation. Collection of demographic data: gender, race, age. Body weight and height of the volunteer are measured without shoes. Body mass index (BMI) according to the formula: BMI \u003d weight / height2 (kg / m2), which uses the value of body weight in kg, and height - in meters;
- Evaluation of the main vital signs: heart rate, respiratory rate, systolic and diastolic blood pressure, body temperature. Heart rate and blood pressure should be measured after the volunteer has rested for 5 minutes in a sitting position;
- Physical examination, including an assessment of the following parameters of the body: the condition of the skin, musculoskeletal system, gastrointestinal tract, respiratory organs, cardiovascular system, urogenital system. Palpation of the lymph nodes (submandibular, cervical, elbow, inguinal) should include an assessment of their size, consistency, pain, mobility, adhesion to each other and to the surrounding tissues and skin. Auscultation data should include a description of the nature of breathing (vesicular, wheezing);
- Electrocardiography (ECG)
- Pulse oximetry
- Collection of data on comorbidities and medical conditions. Concomitant diseases include abnormalities identified during an objective or instrumental examination, which will be described in the primary documentation and in the e-CRI of the subject of the study, including information about the anamnestic data of the subjects included in the study. When describing a concomitant disease or medical condition, the date the condition began and the therapy (if any) for this reason should be indicated;
- Collection of data on previous therapy It is necessary to find out whether the study subject took any medications, including vitamins and dietary supplements, including if the drug was discontinued at the screening visit or within 30 days prior to study entry. At the same time, the primary documentation will need to reflect the international non-proprietary name of the drug (INN), indications for use, daily dose, frequency and timing of taking the drug.

- Blood sampling will be carried out for
    - Biochemical blood test (ALT, AST, protein, bilirubin, cholesterol, LDH, alkaline phosphatase, glucose, urea, creatinine)
    - General clinical blood test (with calculation of the absolute and relative number of leukocytes and ESR).
    - Coagulograms (APTT and prothrombin / prothrombin time / Quick prothrombin / INR)
    - Complete blood count, biochemical blood test, if the study participant does not receive results of previous tests within 14 days before the date of screening
    - Human Immunodeficiency Virus (HIV), Hepatitis B surface Antigen (HBsAg), Hepatitis C virus (HCV) antibodies, *Treponema pallidum* antibodies, if the participant has no results received within 30 days prior to the screening date of previous test results;
- Urinalysis (specific gravity, pH, transparency, protein, glucose, ketones, microscopy: erythrocytes, leukocytes, epithelium, bacteria, mucus, salts);
- PCR testing for COVID-19. Smear sampling will be carried out in the manner accepted in the research center. Do not eat, drink, brush your teeth, rinse your mouth/throat, do not chew gum, do not smoke 3–4 hours before taking smears from the oropharynx (pharynx). 3–4 hours before taking swabs from the nose, do not instill drops / sprays and do not rinse the nose; the analysis is performed at the screening, if the participant of the study does not have the results of previously conducted tests performed in the institutions of the Moscow Department of Health within 7 days before the date of the screening;
- All subjects of the study at the screening stage will take a sample of venous blood to determine the antibodies of the IgM and IgG classes to the SARS-CoV-2 virus, the analysis is performed at the screening, if the study participant does not have the results of previously conducted tests within 7 days before the screening date performed in the institutions of the system of the Department of Health of the city of Moscow;

The sampling of venous blood and the performance of the study will be carried out in the manner accepted in the research center. It is necessary to donate blood on an empty stomach (drinking - as usual, on the eve of avoiding food overload);

- Testing for pregnancy;
- Testing for the presence of narcotic and psychostimulant drugs; Will be tested for alcohol content;

Further, in the course of the study, the subject of the study throughout the study, on all issues, applies to the medical organization in which (or with a representative of which during the exit screening) the subject and his parent/adoptive parent signed the Informed Consent.

From the moment of inclusion in the study, registration of SAE is carried out.

Additional testing, retests and rescreening

In case of obtaining ambiguous or doubtful results of laboratory tests at screening, a second analysis (re-test) may be carried out at the discretion of the investigator or in agreement with the sponsor or his representative.

Volunteers who do not meet inclusion/non-inclusion criteria due to correctable conditions may be re-screened in consultation with the sponsor or its representative. In this case, all screening procedures will be carried out again, including the procedure for obtaining Informed Consent and assigning a new identification number.

If the researcher considers it necessary, additional diagnostic procedures can be performed on the volunteer using instrumental and laboratory research methods not provided for in this Research Protocol to decide on compliance with the inclusion/non-inclusion criteria.

Volunteers meeting all eligibility criteria will be included in the study.

Visit 2 (hospitalization for 3 days: days 0-2): vaccination

Day 0 (hospitalization)

The investigational drug will be administered at the hospital setting in order to monitor the condition of volunteers - they will be hospitalized at the hospital on the eve of the day of drug administration.

PCR testing for COVID-19 will be carried out (in case of a positive test result, the volunteer will be excluded from the study, the vaccine will not be administered).

On day 0, all or part of the screening procedures may be performed. A urine sample will be obtained for:

- performing a drug test
- carrying out a pregnancy test (for female subjects). An alcohol test will be done.

A positive urine drug or alcohol test result will result in the subject being disqualified from further participation in the study.

Subjects eligible to participate in the study will then undergo a physical examination, and their vital signs (HR, BP, RR, and temperature) will be determined.

Day 1 (vaccination with the first component of the ED)

Subjects will be asked about any changes in health status or any medications taken since the day of screening.

Blood samples will be taken for:

- Study of cellular immunity
- Titer of SARS-CoV-2 glycoprotein-specific antibodies
- Virus neutralizing activity
- Antibodies against adenoviruses

A physical examination, assessment of vital signs, registration of concomitant therapy will be carried out. Pulse oximetry will be performed immediately prior to vaccination.

An **intramuscular injection of the first component** of the drug Gam-COVID-Vac M Combined vector vaccine for the prevention of coronavirus infection caused by the SARS-CoV-2 virus will be performed. The administration of the drug is carried out in a sitting or lying position.

Registration of AE is carried out after ED administration.

Vital sign assessment, local and systemic reactions will be assessed immediately before, 0.5 and 2, 6, 12 hours after ED injection.

The injection site will be evaluated for local reactions immediately after injection, 0.5 and 2, 6, 12 hours after drug administration (hyperemia at the injection site, swelling, edema, assessment of regional lymph nodes).

If necessary and in case of adverse events, the frequency of observations and questioning of the volunteer can be increased at the discretion of the researcher, additional information should be entered in the volunteer's CRF.

The subject will be asked to rate the pain of the injection using the VAS for pain (see Appendix 1) immediately after injection, 2 and 12 hours after injection.

Day 2 (follow-up)

Twice during the day, 24 and 36 hours after injection, subjects will undergo a physical examination, the indicators of the functions of the main vital organs will be determined, local and systemic reactions will be assessed.

The injection site will be evaluated for local reactions during physical examinations (hyperemia at the injection site, swelling, edema, evaluation of regional lymph nodes). If necessary and the development of adverse events - the frequency of observations and questioning of the volunteer can be increased at the discretion of the researcher, additional information should be entered in the volunteer's CRF. The subject will be asked to rate the pain of the injection using the VAS for pain (see Appendix 1) 24 and 36 hours after drug administration.

Adverse events (AE/SAE) are recorded. It is noted whether there have been any changes in concomitant therapy and comorbid conditions of the volunteer.

The discharge of the volunteer is carried out on the morning of the next day, after the examination 48 hours after the administration of the drug.

Subjects will undergo a physical examination, key vital organ function tests will be determined, and local and systemic responses will be assessed.

The injection site will be evaluated for local reactions during physical examinations (injection site hyperemia, swelling, edema, evaluation of regional lymph nodes), VAS for pain.

If necessary and the development of adverse events - the frequency of observations and questioning of the volunteer can be increased at the discretion of the researcher, additional information should be entered in the volunteer's CRF.

Blood samples will be taken for:

- Biochemical blood test (ALT, AST, protein, bilirubin, cholesterol, LDH, alkaline phosphatase, prothrombin, glucose, urea, creatinine)
- Complete blood count (with calculation of the absolute and relative number of leukocytes and ESR).

A urine sample will be obtained for general urinalysis with sediment microscopy (specific gravity, pH, transparency, protein, glucose, ketones, microscopy: erythrocytes, leukocytes, epithelium, bacteria, mucus, salts).

At the end of the visit, volunteers will be trained in keeping a volunteer diary, in which they will independently record possible AE and the use of concomitant therapy. Volunteers will also be able to contact the Researcher by telephone.

After the completion of the procedures, the volunteers will be discharged.

Phone contacts: day 3/4, day 5, day 7

On study days 3/4, 5, and 7, the investigator will contact volunteers by telephone to assess complaints, AE/SAE, and administer concomitant therapy. If necessary, volunteers will be invited to the center for an unscheduled visit.

Visit 3 (day 14): follow-up

Subjects will undergo a physical examination, key vital organ function tests will be determined, and local and systemic responses will be assessed.

The injection site will be evaluated for local reactions during physical examinations (hyperemia at the injection site, swelling, edema, evaluation of regional lymph nodes). If necessary and the development of adverse events - the frequency of observations and questioning of the volunteer can be increased at the discretion of the researcher, additional information should be entered in the volunteer's CRF. The volunteer's diary will be checked. Complaints, AE/SAE and concomitant therapy were assessed.

A visit, at the discretion of the investigator's physician, may be substituted for a telephone consultation.

Visit 4 (days 20–22 ± 1): vaccination

Day 20 (hospitalization)

The investigational drug will be administered at the hospital setting in order to monitor the condition of volunteers - they will be hospitalized at the hospital on the eve of the day of drug administration.

PCR testing for COVID-19 will be carried out (in case of a positive test result, the volunteer will be excluded from the study, the vaccine will not be administered).

A urine sample will be obtained for:

- general urine analysis with sediment microscopy (specific gravity, pH, transparency, protein, glucose, ketones, microscopy: erythrocytes, leukocytes, epithelium, bacteria, mucus, salts).
- performing a drug test
- carrying out a pregnancy test (for female subjects). An alcohol test will be done.

An alcohol test will be done.

A positive urine drug or alcohol test result will result in the subject being disqualified from further participation in the study.

Subjects eligible to participate in the study will then undergo a physical examination, and their vital signs (HR, BP, RR, and temperature) will be determined.

Blood samples will be taken for:

- Biochemical blood test (ALT, AST, protein, bilirubin, cholesterol, LDH, alkaline phosphatase, prothrombin, glucose, urea, creatinine)
- Complete blood count (with calculation of the absolute and relative number of leukocytes and ESR).
- Titer of SARS-CoV-2 glycoprotein-specific antibodies

It is allowed to carry out procedures in a sequence convenient for the clinical center.

The transfer of part of the procedures to day 21 is allowed.

Day 21 (Vaccination with the second ED component)

Prior to vaccination, a physical examination and assessment of vital signs will be performed. Pulse oximetry will be done.

An **intramuscular injection of the second component** of the Gam-COVID-Vac M preparation will be performed. Combined vector vaccine for the prevention of coronavirus infection caused by the SARS-CoV-2 virus. Drug administration is carried out in a sitting or lying position.

Vital sign assessment, local and systemic reactions will be assessed immediately before, 0.5 and 2, 6, 12 hours after ED injection.

The injection site will be evaluated for local reactions immediately after injection, 0.5 and 2, 6, 12 hours after drug administration (hyperemia at the injection site, swelling, edema, assessment of regional lymph nodes).

If necessary and the development of adverse events - the frequency of observations and questioning of the volunteer can be increased at the discretion of the researcher, additional information should be entered in the volunteer's CRF.

The subject will be asked to rate the pain of the injection using the VAS for pain (see Appendix 1) immediately after injection, 2 and 12 hours after injection.

Day 22 (follow-up)

Twice during the day, 24 and 36 hours after injection, subjects will undergo a physical examination, the indicators of the functions of the main vital organs will be determined, local and systemic reactions will be assessed.

The injection site will be evaluated for local reactions during physical examinations (hyperemia at the injection site, swelling, edema, evaluation of regional lymph nodes). If necessary and the development of adverse events - the frequency of observations and questioning of the volunteer can be increased at the discretion of the researcher, additional information should be entered in the volunteer's CRF. The subject will be asked to rate the pain of the injection using the VAS for pain (see Appendix 1) 24 and 36 hours after drug administration.

Undesirable phenomena (AEs) are registered. It is noted whether there have been any changes in concomitant therapy and comorbid conditions of the volunteer.

The discharge of the volunteer is carried out on the morning of the next day, after the examination 48 hours after the administration of the drug.

Subjects will undergo a physical examination, key vital organ function tests will be determined, and local and systemic responses will be assessed.

The injection site will be evaluated for local reactions during physical examinations (hyperemia at the injection site, swelling, edema, evaluation of regional lymph nodes).

If necessary and the development of adverse events - the frequency of observations and questioning of the volunteer can be increased at the discretion of the researcher, additional information should be entered in the volunteer's CRF.

Blood samples will be taken for:

- Biochemical blood test (ALT, AST, protein, bilirubin, cholesterol, LDH, alkaline phosphatase, prothrombin, glucose, urea, creatinine)
- Complete blood count (with calculation of the absolute and relative number of leukocytes and ESR).

A urine sample will be obtained for general urinalysis with sediment microscopy (specific gravity, pH, transparency, protein, glucose, ketones, microscopy: erythrocytes, leukocytes, epithelium, bacteria, mucus, salts).

Upon completion of the visit procedures, the volunteers will be discharged.

Phone contacts: day 23/24, day 25, day 27

On study days 23/24, 25, and 27, the investigator will contact volunteers by telephone to assess complaints, AE/SAE, and administer concomitant therapy. If necessary, volunteers will be invited to the center for an unscheduled visit.

Visit 5 day 28 (follow-up)

Subjects will undergo a physical examination, key vital organ function tests will be determined, and local and systemic responses will be assessed. Undesirable phenomena (AEs) are registered. It is noted whether there have been any changes in concomitant therapy and comorbid conditions of the volunteer. An electrocardiogram (ECG) will be performed.

A urine sample will be obtained for general urinalysis with sediment microscopy (specific gravity, pH, transparency, protein, glucose, ketones, microscopy: erythrocytes, leukocytes, epithelium, bacteria, mucus, salts).

Blood samples will be taken for:

– Study of cellular immunity
– Titer of SARS-CoV-2 glycoprotein-specific antibodies
– Virus neutralizing activity
– Biochemical blood test (ALT, AST, protein, bilirubin, cholesterol, LDH, alkaline phosphatase, glucose, urea, creatinine)
– General clinical blood test (with calculation of the absolute and relative number of leukocytes and ESR).
– Coagulograms (APTT and prothrombin / prothrombin time / Quick prothrombin / INR)

Visit 6 day 42 (follow-up)

Subjects will undergo a physical examination, key vital organ function tests will be determined, and local and systemic responses will be assessed. Adverse events (AE/SAE) are recorded. It is noted whether there have been any changes in concomitant therapy and comorbid conditions of the volunteer. An electrocardiogram (ECG) will be performed if indicated.

Blood samples will be taken for:

– Titer of SARS-CoV-2 glycoprotein-specific antibodies
– Virus neutralizing activity
– Antibodies against adenoviruses

Volunteer diaries will be checked.

Visit 7 day 90 (follow-up)

Subjects will undergo a physical examination, key vital organ function tests will be determined, and local and systemic responses will be assessed. Adverse events (AE/SAE) are recorded. It is noted whether there have been any changes in concomitant therapy and comorbid conditions of the volunteer.

Blood samples will be taken for:

– Titer of SARS-CoV-2 glycoprotein-specific antibodies
– Virus neutralizing activity Volunteers' diaries will be checked.

Visit 8 day 180 (follow-up)

Subjects will undergo a physical examination, key vital organ function tests will be determined, and local and systemic responses will be assessed. Adverse events (AE/SAE) are recorded. It is noted whether there have been any changes in concomitant therapy and comorbid conditions of the volunteer.

Blood samples will be taken for:

- Titer of SARS-CoV-2 glycoprotein-specific antibodies
- Virus neutralizing activity
- Antibodies against adenoviruses

Volunteer diaries will be checked

Phone contacts: day 270, day 365

On study days 270, 365, the investigator will contact volunteers by phone to assess complaints, AE/SAE, and administer concomitant therapy. If necessary, volunteers will be invited to the center for an unscheduled visit.

4.2.2.2 2 stage (phase III)

The second phase of the study will include 3,000 volunteers who will be randomized into two groups - the active drug (vaccines) and placebo in a 4: 1 ratio, screened (no more than 7 days before enrollment in the study) there must be a sufficient number of volunteers to be included in the study. research.8. Subjects will be randomized into two age strata: 12–14 years old and 15–17 years old.

500 volunteers will donate blood for the study of immunogenicity during the study (400 in the vaccine group and 100 in the placebo group, after blinding, only volunteers who received the vaccine will continue to donate blood for immunogenicity: 400 people).

The conditions for the study were vaccination with the dose selected at the first stage on an outpatient basis.
The first dose will be given on day 1. After that, the volunteer will be observed for 30-60 minutes. On days 2, 3 and 7, a telephone consultation will be held.

Diet standardization, restrictions.
The administration of the study drug is carried out on an empty stomach, if possible.
The volunteer should refrain from intense physical activity and contact sports 24 hours before and for a week after the administration of the drug.
The volunteer must not take any medications (including herbal preparations and nutritional supplements) without the consent of the research physician.
Volunteers are required to use adequate contraception throughout their participation in the study.

Visit 1 (day -7 – -1/0): screening

The screening period in the study begins from the moment the informed consent is signed and lasts no more than 7 days (from -7 to -1/0 days) until the volunteer is included in the study. Conducting any procedures related to the study to the subject of the study is not allowed until the signing of the IS form. At the same time, each subject should have sufficient time to familiarize himself with the text of consent in order to give informed and voluntary consent to participate in the study. The date and time of signing the IS must be included in the primary medical documentation and CRF.

[8] Prior to the start of the study, in order to form a base of volunteers planning to participate in the study, it is allowed to organize a pre-screening procedure. Prescreening involves a routine medical examination

During Visit 1, volunteers will be assessed for meeting the eligibility criteria and not eligibility criteria. Volunteers potentially eligible to participate in a clinical trial will be invited to participate.

During the screening visit, the following procedures will be performed:

- Signing by the subject and his parent/adoptive parent of the study of the IP form for participation in the study;
- Collection of anamnesis, demographic and anthropometric data: collection of data on concomitant and previously transferred diseases, concomitant therapy, collection of information on the presence of allergies, on the contacts of the subject of the study on contact with patients with COVID-19, on previous vaccinations / immunizations, on blood donation. Collection of demographic data: gender, race, age. Body weight and height of the volunteer are measured without shoes. Body mass index (BMI) according to the formula: BMI \u003d weight / height2 (kg / m2), which uses the value of body weight in kg, and height - in meters;
- Evaluation of the main vital signs: heart rate, respiratory rate, systolic and diastolic blood pressure, body temperature. Heart rate and blood pressure should be measured after the volunteer has rested for 5 minutes in a sitting position;
- Physical examination, including an assessment of the following parameters of the body: the condition of the skin, musculoskeletal system, gastrointestinal tract, respiratory organs, cardiovascular system, urogenital system. Palpation of the lymph nodes (submandibular, cervical, elbow, inguinal) should include an assessment of their size, consistency, pain, mobility, adhesion to each other and to the surrounding tissues and skin. Auscultation data should include a description of the nature of breathing (vesicular, wheezing);
- Electrocardiography (ECG)
- Pulse oximetry
- Collection of data on comorbidities and medical conditions. Concomitant diseases include abnormalities identified during an objective or instrumental examination, which will be described in the primary documentation and in the e-CRI of the subject of the study, including information about the anamnestic data of the subjects included in the study. When describing a concomitant disease or medical condition, the date the condition began and the therapy (if any) for this reason should be indicated;
- Collection of data on previous therapy It is necessary to find out whether the study subject took any medications, including vitamins and dietary supplements, including if the drug was discontinued at the screening visit or within 30 days prior to study entry. At the same time, the primary documentation will need to reflect the international non-proprietary name of the drug (INN), indications for use, daily dose, frequency and timing of taking the drug.
- Blood sampling will be carried out for
    - Biochemical blood test (ALT, AST, protein, bilirubin, cholesterol, alkaline phosphatase, glucose, urea, creatinine)
    - General clinical blood test (with calculation of the absolute and relative number of leukocytes and ESR).
    - Coagulograms (APTT and prothrombin / prothrombin time / Quick prothrombin / INR)

  - Complete blood count, biochemical blood test, if the study participant does not receive results of previous tests within 14 days before the date of screening
  - Human Immunodeficiency Virus (HIV) test, Hepatitis B surface Antigen (HBsAg), Hepatitis C virus (HCV) antibody test, Treponema pallidum antibody test, if participant does not have studies obtained within 30 days prior to the screening date of previous test results;
- Urinalysis (specific gravity, pH, transparency, protein, glucose, ketones, microscopy: erythrocytes, leukocytes, epithelium, bacteria, mucus, salts);
- PCR testing for COVID-19. Smear sampling will be carried out in the manner accepted in the research center. Do not eat, drink, brush your teeth, rinse your mouth/throat, do not chew gum, do not smoke 3-4 hours before taking smears from the oropharynx (pharynx). 3-4 hours before taking swabs from the nose, do not instill drops / sprays and do not rinse the nose; the analysis is performed at the screening, if the participant of the study does not have the results of previously conducted tests performed in the institutions of the Moscow Department of Health within 7 days before the date of the screening;
- All subjects of the study at the screening stage will take a sample of venous blood to determine the antibodies of the IgM and IgG classes to the SARS-CoV-2 virus, the analysis is performed at the screening, if the study participant does not have the results of previously conducted tests within 7 days before the screening date performed in the institutions of the system of the Department of Health of the city of Moscow;

The sampling of venous blood and the performance of the study will be carried out in the manner accepted in the research center. It is necessary to donate blood on an empty stomach (drinking - as usual, on the eve of avoiding food overload);

Urinalysis for testing:

- for pregnancy;
- for the presence of narcotic and psychostimulant drugs;

An alcohol test will be conducted.

Further, in the course of the study, the subject of the study throughout the study, on all issues, applies to the medical organization in which (or with a representative of which during the exit screening) the subject and his parent/adoptive parent signed the Informed Consent.

From the moment of inclusion in the study, registration of SAE is carried out.

Additional testing, retests and rescreening

In case of obtaining ambiguous or doubtful results of laboratory tests at screening, a second analysis (re-test) may be carried out at the discretion of the investigator or in agreement with the sponsor or his representative.

Volunteers who do not meet inclusion/non-inclusion criteria due to correctable conditions may be re-screened in consultation with the sponsor or its representative. In this case, all screening procedures will be carried out again, including the procedure for obtaining Informed Consent and assigning a new identification number.

If the researcher considers it necessary, additional diagnostic procedures can be performed on the volunteer using instrumental and laboratory research methods not provided for in this Research Protocol to decide on compliance with the inclusion/non-inclusion criteria.

The rules for using the volunteer diary will be explained.

Volunteers meeting all eligibility criteria will be included in the study.

Visit 2 (day 1): vaccination

Volunteers will be randomized into two groups - the active drug (vaccines) and placebo in a ratio of 4: 1, randomization of study subjects will be carried out in two age strata: 12–14 years and 15–17 years.

Vaccination is carried out on an outpatient basis, in the same medical organizations in which screening was carried out.

Before vaccination, volunteers will undergo a physical examination, assessment of the main vital signs: heart rate, respiratory rate, systolic and diastolic blood pressure, body temperature. Heart rate and blood pressure should be measured after the volunteer has rested for 5 minutes in a sitting position. Concomitant therapy will be registered. Pulse oximetry will be performed immediately prior to vaccination.

All volunteers will be tested by PCR for COVID-19 prior to vaccination. Smear sampling will be carried out in the manner accepted in the research center. Do not eat, drink, brush your teeth, rinse your mouth/throat, do not chew gum, do not smoke 3–4 hours before taking smears from the oropharynx (pharynx). 3–4 hours before taking swabs from the nose, do not instill drops / sprays and do not rinse the nose.

All participants in the additional immunogenicity study (500 volunteers) will receive blood samples for:

- Titer of SARS-CoV-2 glycoprotein-specific antibodies
- Virus neutralizing activity

100 volunteers - will be tested for antibodies against adenoviruses (among those vaccinated with the active drug, which will be determined after blinding)

An **intramuscular injection of the first component** of the drug Gam-COVID-Vac M Combined vector vaccine for the prevention of coronavirus infection caused by the SARS-CoV-2 virus will be performed. Administration of the drug is carried out in a sitting or lying position.

A urine sample will be obtained for:

- Testing for pregnancy;
- Testing for the presence of narcotic and psychostimulant drugs.

An alcohol test will be conducted.

Registration of AE is carried out after ED administration.

Vital sign assessment, local and systemic reactions will be assessed immediately before, 30-60 minutes after ED injection.

The injection site will be assessed for local reactions immediately after administration, 30-40 minutes after administration of the drug (hyperemia at the injection site, swelling, edema, assessment of regional lymph nodes).

Changes in vital signs after vaccination compared with baseline, regarded as adverse events, should be recorded in the primary documentation and in the e-CRF, as appropriate;

Phone contacts: day 2, day 3, day 7

On study days 2, 3, and 7, the study physician will contact volunteers by telephone to assess complaints, AE/SAE, and administer concomitant therapy. If necessary, volunteers will be invited to the center for an unscheduled visit.

Visit 3 (day 21): vaccination

Vaccination is carried out on an outpatient basis, in the same medical organizations in which screening was carried out.

Before vaccination, volunteers will undergo a physical examination, assessment of the main vital signs: heart rate, respiratory rate, systolic and diastolic blood pressure, body temperature. Heart rate and blood pressure should be measured after the volunteer has rested for 5 minutes in a sitting position. Concomitant therapy will be registered. Pulse oximetry will be performed immediately prior to vaccination.

All volunteers will be tested by PCR for COVID-19 prior to vaccination. Smear sampling will be carried out in the manner accepted in the research center. Do not eat, drink, brush your teeth, rinse your mouth/throat, do not chew gum, do not smoke 3-4 hours before taking smears from the oropharynx (pharynx). 3-4 hours before taking swabs from the nose, do not instill drops / sprays and do not rinse the nose.

An **intramuscular injection of the second component** of the drug Gam-COVID-Vac M Combined vector vaccine for the prevention of coronavirus infection caused by the SARS-CoV-2 virus will be performed. Administration of the drug is carried out in a sitting or lying position.

A urine sample will be obtained for:

- Testing for pregnancy;
- Testing for the presence of narcotic and psychostimulant drugs.

An alcohol test will be conducted.

Registration of AE is carried out after ED administration.

Vital sign assessment, local and systemic reactions will be assessed immediately before, 30-60 minutes after ED injection.

The injection site will be assessed for local reactions immediately after administration, 30-40 minutes after administration of the drug (hyperemia at the injection site, swelling, edema, assessment of regional lymph nodes).

Changes in vital signs after vaccination compared with baseline, regarded as adverse events, should be recorded in the primary documentation and in the e-CRF, as appropriate;

Phone contacts: day 22, day 23

On study days 22, 23, the investigator will contact volunteers by telephone to assess complaints, AE/SAE, and administer concomitant therapy.

If necessary, volunteers will be invited to the center for an unscheduled visit. The volunteer will undergo a physical examination, including an assessment of the following parameters of the body: the condition of the skin, the musculoskeletal system, the gastrointestinal tract, the respiratory system, the cardiovascular system, and the genitourinary system. Palpation of the lymph nodes (submandibular, cervical, elbow, inguinal) should include an assessment of their size, consistency, pain, mobility, adhesion to each other and to the surrounding tissues and skin. Auscultation data should include a description of the nature of breathing (vesicular, wheezing);

Visit 4 (day 28): follow-up

The volunteer will undergo a physical examination, including an assessment of the following parameters of the body: the condition of the skin, the musculoskeletal system, the gastrointestinal tract, the respiratory system, the cardiovascular system, and the genitourinary system. Palpation of the lymph nodes (submandibular, cervical, elbow, inguinal) should include an assessment of their size, consistency, pain, mobility, adhesion to each other and to the surrounding tissues and skin. Auscultation data should include a description of the nature of breathing (vesicular, wheezing); An electrocardiogram (ECG) will be performed.

All volunteers will have urine samples taken for general analysis and blood samples for:

- Biochemical blood test (ALT, AST, protein, bilirubin, cholesterol, alkaline phosphatase, glucose, urea, creatinine)
- General clinical blood test (with calculation of the absolute and relative number of leukocytes and ESR).
- Coagulograms (APTT and prothrombin / prothrombin time / Quick prothrombin / INR)
- Approximately 500 volunteers
- Virus neutralizing activity
- Titer of SARS-CoV-2 glycoprotein-specific antibodies

The sampling of venous blood and the performance of the study will be carried out in the manner accepted in the research center. It is necessary to donate blood on an empty stomach (drinking - as usual, on the eve of avoiding food overload);

After the 28th day, the unblinding procedure will be carried out. Volunteers who received the vaccine will continue to follow the study plan. Volunteers who receive a placebo will be asked to administer the vaccine and continue follow-up as planned.

Visit 5 (day 42): follow-up

The volunteer will undergo a physical examination, including an assessment of the following parameters of the body: the condition of the skin, the musculoskeletal system, the gastrointestinal tract, the respiratory system, the cardiovascular system, and the genitourinary system. Palpation of the lymph nodes (submandibular, cervical, elbow, inguinal) should include an assessment of their size, consistency, pain, mobility, adhesion to each other and to the surrounding tissues and skin. Auscultation data should include a description of the nature of breathing (vesicular, wheezing); An electrocardiogram (ECG) will be done if indicated.

Approximately 400 volunteers will receive blood samples for:

- Titer of SARS-CoV-2 glycoprotein-specific antibodies
- Virus neutralizing activity

About 100 volunteers: antibodies against adenoviruses

Diaries will be checked

Visit 6, day 90 (follow-up)

The Investigating Physician will contact volunteers by phone to assess complaints, AE/SAE, and administer concomitant therapy.

If necessary, volunteers will be invited to the center for a visit. Subjects will undergo a physical examination, key vital organ function tests will be determined, and local and systemic responses will be assessed. Adverse events (AE/SAE) are recorded. It is noted whether there have been any changes in concomitant therapy and comorbid conditions of the volunteer.

Approximately 400 volunteers will have blood samples taken to analyze the titer of SARS-CoV-2 glycoprotein-specific antibodies.

Volunteer diaries will be checked.

Visit 7, day 180 (follow-up)

Subjects will undergo a physical examination, key vital organ function tests will be determined, and local and systemic responses will be assessed. Adverse events (AE/SAE) are recorded. It is noted whether there have been any changes in concomitant therapy and comorbid conditions of the volunteer.

Approximately 400 volunteers will have blood samples taken for:

- Titer of SARS-CoV-2 glycoprotein-specific antibodies
- Virus neutralizing activity

About 100 volunteers: antibodies against adenoviruses

Volunteer diaries will be checked.

Phone contacts: day 270, day 365

On study days 270 and 360, the study physician will contact volunteers by telephone to assess complaints, AE/SAE, and administer concomitant therapy. If necessary, volunteers will be invited to the center for an unscheduled visit.

4.2.3 Graphic presentation of the study

General scheme of the study

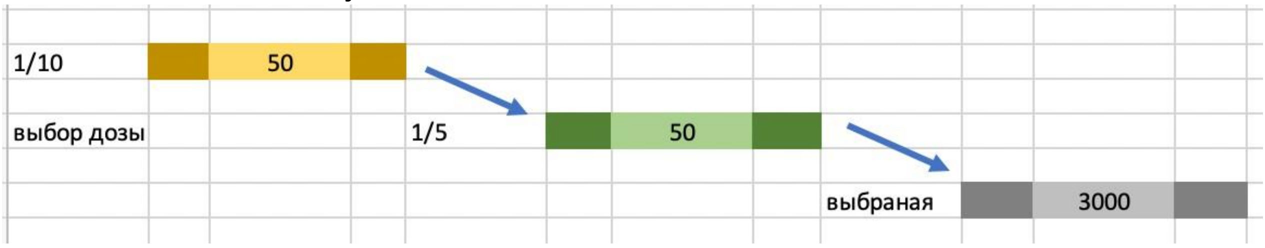

Scheme of stage 1 of the study

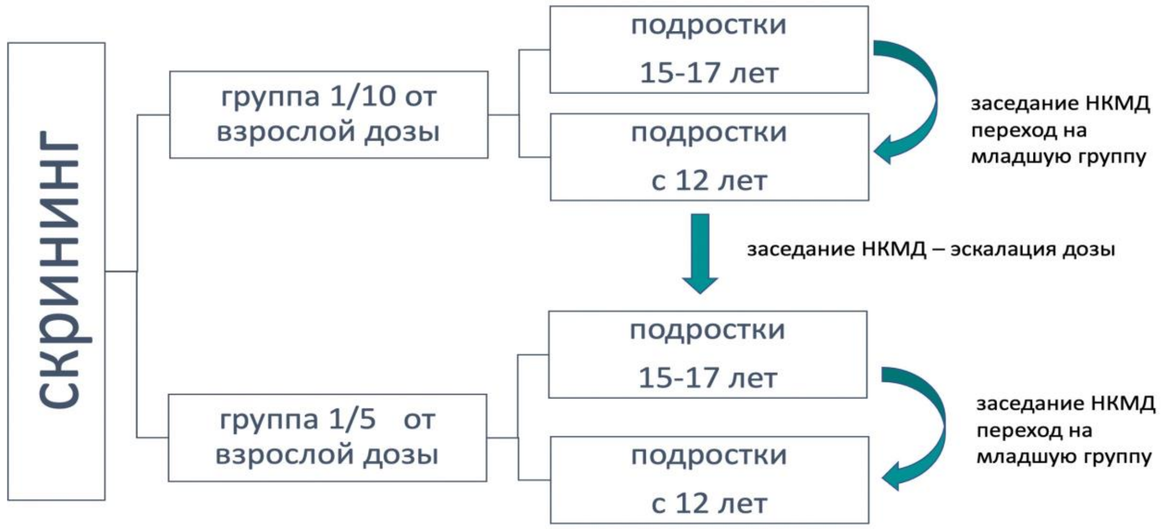

Dose selection will be based on safety and immunogenicity results.

Scheme of stage 2 of the study

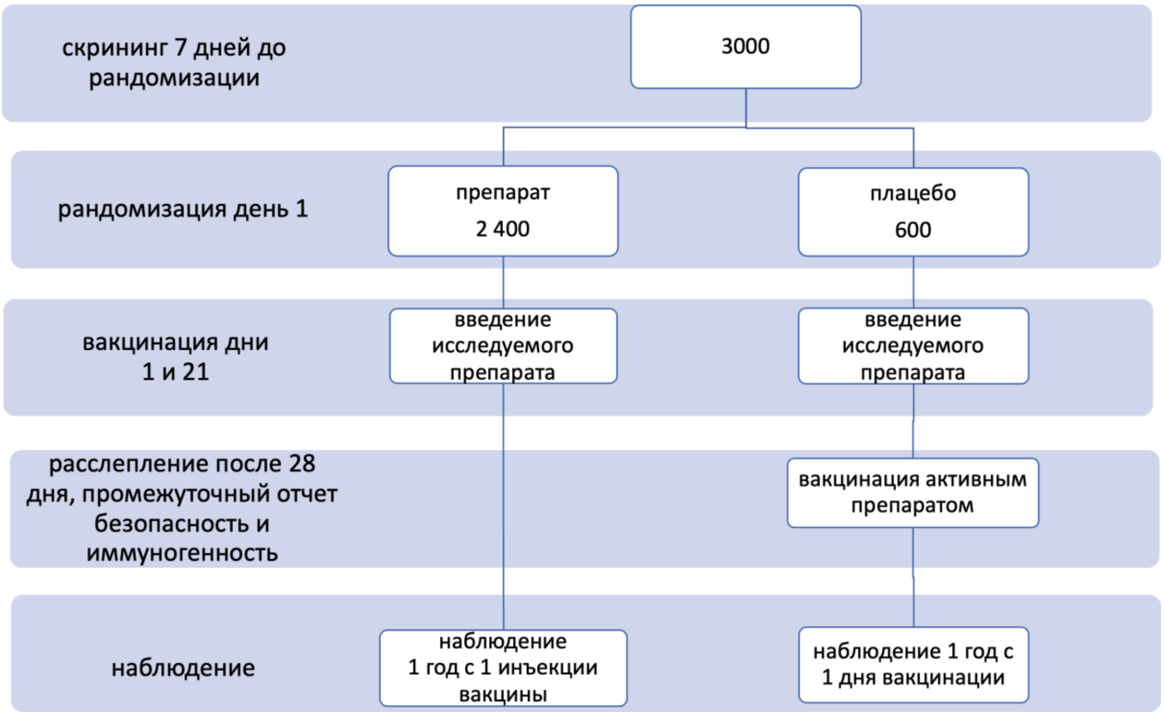

4.2.4 Schedule of examination procedures in tabular form

1 stage

parameter	screening / visit 1	hospotalization / visit 2			PMC	PMC	PMC	visit 3	hospotalization / visit 4			PMC	PMC	PMC	visit 5	visit 6	visit 7	visit 8	PMC	PMC
	-7-0	0	1	2	3	5	7	14	20	21	22	23	25	27	28	42	90	180	270	365
Signing the IS Form	x																			
collection of anamnesis, demographic and anthropometric data	x																			
Evaluation of inclusion/exclusion criteria	x																			
Physical examination. Estimation of the main vital important indicators	x	x	x	x				x	x	x	x				x	x	x	x		
ECG in 12 leads	x														x	x**				
clinical blood test	x			x					x		x				x					
urinalysis	x			x					x		x				x					
Coagulogram	x														x					
biochemical blood assay	x			x					x		x				x					
Testing for IgM and IgG antibodies against SARS CoV2	x																			
Testing for HIV, syphilis, hepatitis B and C	x																			
PCR testing for COVID-19	x	x							x											
Pulse oximetry			x							x										
Pregnancy testing	x	x							x											

Testing for the presence of narcotic and psychostimulant drugs	x	x							x											

Alcohol testing	x	x							x											
Vaccination			x							x										
Telephone contact [6]					x	x	x	/x				x	x	x					x	x
Filling in diaries by subjects					x	x	x	x				x	x	x	x	x	x	x	x	x
VNA test			x							x					x	x	x	x		
Determination of cellular immunity parameters			x												x					
Determination of the titer of SARS-CoV-2 glycoprotein-specific antibodies			x							x					x	x	x	x		
Antibodies against adenovirus			x													x				x
Collection of data on the occurrence of AE/SAE		x	x	x	x	x	x	x	x	x	x	x	x	x	x	x	x	x	x	x

2 stage
group receiving vaccine

parameters	screening / visit 1	vaccination / visit 2	remote monitoring			vaccination /visit 3	remote monitoring		visit 4	visit 5	visit 6	visit 7	remote monitoring	
	-7-0	1	2	3	7	21	22	23	28	42	90	180	270	365
Signing the IS Form	x													
collection of anamnesis, demographic and anthropometric data	x													
Evaluation of inclusion/exclusion criteria	x													
Physical examination. Estimation of the main vital important indicators	x	x				x			x	x		x		
ECG in 12 leads	x								x	x**				
clinical blood test	x								x					
urinalysis	x								x					
Coagulogram	x								x					
biochemical blood assay	x								x					
Testing for IgM and IgG antibodies against SARS CoV2	x													
Testing for HIV, syphilis, hepatitis B and C	x													
PCR testing for COVID-19	x	x				x								
Pulse oximetry	x	x				x								
Pregnancy testing	x	x				x								

Testing for the presence of narcotic and psychostimulant drugs	x	x				x								
Alcohol testing	x	x				x								
Randomization		x												
Vaccination		x				x								
Blinding									x					
Phone contact			x	x	x		x	x			x		x	x
Filling in diaries by subjects		x	x	x	x	x	x	x	x	x	x	x	x	x
VNA test		x*							x*	x*		x*		
Determination of antibodies against adenoviruses		x***								x***		x***		
Determination of the titer of SARS-CoV-2 glycoprotein-specific antibodies		x*							x*	x*	x*	x*		
Collection of data on the occurrence of AE		x	x	x	x	x	x	x	x	x	x	x	x	X

* – only for the blood donation group (about 400 volunteers)
** – based on indications
x*** – for 100 volunteers

Stage 2
placebo group

parameters	screening / visit 1	vaccination / visit 2	remote monitoring			vaccination / visit 3	remote monitoring		visit 4	vaccination / visit 2	remote monitoring			vaccination / visit 3	remote monitoring		visit 4	visit 5	visit 6	visit 7	remote monitoring	
	-7-0	1	2	3	7	21	22	23	28	1	2	3	7	21	22	23	28	42	90	180	270	365
Signing the IS Form	x																					
collection of anamnesis, demographic and anthropometric data	x																					
Evaluation of inclusion/exclusion criteria	x																					
Physical examination. Estimation of the main vital important indicators	x	x				x			x	x				x			x	x		x		
ECG in 12 leads	x								x								x	x**				
clinical blood test	x								x								x					
Coagulogram	x								x								x					
biochemical blood assay	x								x								x					
Testing for IgM and IgG antibodies against SARS CoV2	x																					
Testing for HIV, syphilis, hepatitis B and C	x																					
PCR testing for COVID-19	x	x				x				x				x								
Pulse oximetry	x	x				x				x				x								
Pregnancy testing	x	x				x				x				x								
Testing for the presence of narcotic and psychostimulant drugs	x	x				x				x				x								
Alcohol testing	x	x				x				x				x								

Randomization		x								x												
Vaccination		x				x				x				x								
Blinding									x								x					
Phone contact			x	x	x		x	x			x	x	x		x	x			x		x	x
Filling in diaries by subjects		x	x	x	x	x	x	x	x	x	x	x	x	x	x	x	x	x	x	x	x	x
Urinalysis	x								x								x					
VNA test		x*							x*													
Determination of the titer of SARS-CoV-2 glycoprotein-specific antibodies		x*							x*													
Collection of data on the occurrence of AE		x	x	x	x	x	x	x	x	x	x	x	x	x	x	x	x	x	x	x	x	X

* – only for the group of blood donators (about 100 volunteers before blinding - after blinding they do not donate blood)

** – based on indications

4.2.5 Study procedures

4.2.5.1 Obtaining informed consent

Prior to any study procedures, the volunteer and their parent/adoptive parent must read the information for the volunteer and sign the informed consent form. The researcher must ensure that the volunteer and his/her parent/adoptive parent correctly understand the information about research procedures and associated limitations.

The volunteer and his/her parent/adoptive parent should have sufficient time to reflect on their participation in the study. The volunteer and his parent/adoptive parent should be given the opportunity to ask additional questions and get answers to them.

The ethical and regulatory aspects of obtaining this procedure, as described in Section 11.2, must be taken into account when obtaining informed consent.

In a conversation between the Researcher and the volunteer, special attention should be paid to discussing the following aspects:

- Features (pharmacological group, mechanism of action, indications and contraindications for use, possible adverse reactions, route of administration and dose) of the study drug;
- Conditions for blood sampling;
- Time of arrival of the volunteer at the Research Center;
- Day regimen, eating regimen, drinking regimen, restrictions on taking medications and other restrictions;
- The possibility of AE and their manifestations;
- Ability to provide medical assistance during the study;
- Conditions of health insurance and remuneration;
- Opportunity to voluntarily withdraw from the study;
- Confidentiality of personal data and information about the volunteer.

4.2.5.2 Collection of anamnesis, demographic data, anthropometry

When collecting an anamnesis, attention is paid to previous diseases, concomitant chronic diseases, heredity, habitual intoxications (smoking status, alcohol, drug use), allergic anamnesis, surgeries and injuries, permanent or periodic drug therapy. The method of contraception used by the study participant is specified, when signing the informed consent, the participant and his parent / adoptive parent agree to use one of the methods of contraception recommended in accordance with the study protocol from the start of the study to 3 months. after the second dose of the vaccine.

In the primary documentation, the gender and age of the volunteer are noted. With the help of a stadiometer, the height of a volunteer is measured in centimeters, with the help of medical scales - body weight. Height will be measured using a certified stadiometer, body weight - using a certified medical scale.

The calculation of body mass index (BMI) will be carried out in accordance with the formula:

BMI = Body weight (kg)/(height in m)^2.

4.2.5.3 Physical examination

Physical examination will be carried out according to the general rules of propaedeutics of internal diseases: a general examination, examination of the mucous membranes and skin, including palpation of the lymph nodes, assessment of the musculoskeletal system, palpation, percussion and auscultation of the main organ systems (cardiovascular, respiratory, digestive , urinary system).

4.2.5.4 Laboratory studies

Blood sampling for analysis will be done x. The tests required for this study will require

approximately 120.4 ml of blood in the first step and 59.1 ml in the second. Sampling for laboratory examination is carried out in accordance with the study plan (Section 4.2.4).

Clinical and biochemical blood tests, coagulogram and serological analysis for HIV type 1 and 2, HBsAg, HCV, antibodies against *Treponema pallidum* antigens, urinalysis and SARS-CoV-2 RNA analysis by PCR can be performed in the local laboratory of the research center. The research center can also perform rapid tests for SARS-CoV-2 using immunochromatographic assay (ICA). Blood samples for evaluation of immunogenicity (SARS-CoV-2 IgG spike (S) protein antibody titers, virus neutralizing antibody titer, cell-mediated immunity tests) will be performed at the central laboratory.

4.2.5.4.1 Clinical blood analysis

Clinical blood test parameters:

- Hemoglobin concentration;
- Hematocrit;
- Number of erythrocytes;
- Number of platelets;
- The number of leukocytes;
- Leukocyte formula;
- Erythrocyte sedimentation rate.

The leukocyte formula will be calculated indicating the absolute and relative content of: stab neutrophils, segmented neutrophils, eosinophils, basophils, monocytes, lymphocytes.

4.2.5.4.2 Biochemical blood test

Blood samples for biochemical analysis will be taken on an empty stomach from a vein under aseptic/antiseptic conditions. Indicators of a biochemical blood test:

- Glucose concentration;
- The concentration of total cholesterol;
- Concentration of total protein;
- The concentration of total bilirubin;
- Creatinine concentration;
- Alkaline phosphatase (AP) activity;
- Activity of alanine aminotransferase (ALT);
- Aspartate aminotransferase (AST) activity.

4.2.5.4.3 Coagulogram

Coagulogram parameters:

- Prothrombin time / Prothrombin (according to Quick) / International normalized ratio (INR);
- Activated partial thromboplastin time (APTT).

4.2.5.4.4 Serological blood test

Serological blood test parameters:

- Antibodies against HbsAg;
- Antibodies against HCV antigens;
- Antibodies against HIV 1 and 2 types;
- Antibodies against *Treponema pallidum* antigens.

4.2.5.4.5 Urinalysis

Urinalysis parameters:

- Relative density;
- Color;
- Transparency;
- pH;
- Protein concentration;
- Glucose concentration;
- Content of erythrocytes;
- The content of leukocytes;
- Content of epitheliocytes;
- Presence of cylinders;
- The presence of mucus;
- Presence of bacteria.

4.2.5.4.6 Pregnancy test

A pregnancy test for women will be carried out in the conditions of the Research Center by the method of rapid determination of the content of the β-subunit of human chorionic gonadotropin (β-hCG) in the urine using a test strip.

4.2.5.4.7 Laboratory testing of biomaterial (nasopharyngeal and oropharyngeal swab) for SARS-Cov-2 (COVID-19) RNA

Smears are taken with dry sterile probes. The probe is inserted with a slight movement along the outer wall of the nasal cavity to a depth of 2–3 cm to the inferior turbinate. Then the probe is slightly lowered down, inserted into the lower nasal passage under the lower nasal concha and removed along the outer wall of the nasal cavity, making rotational movements. The end of the probe is broken off so that it allows the lid of the tube to be tightly closed. From the oropharynx, a smear is taken with a second probe with rotational movements from the surface of the tonsils, palatine arches and the posterior wall of the oropharynx. After taking the material, the working part of the probe with a swab is placed in a test tube with a transport medium for storing and transporting respiratory swabs and a probe with a swab from the nasopharynx. The end of the probe with a swab is broken off into a test tube, holding the tube lid so that it allows you to tightly close the test tube. The tube is labelled.

The determination of SARS-CoV-2 RNA in the obtained biomaterial will be carried out by PCR.

4.2.5.4.8 Immunological studies

As part of the study, the following indicators will be determined:

- Analysis for IgM and IgG to SARS-CoV-2;
- Titer of IgG antibodies against SARS-CoV-2 spike (S) protein;
- Virus neutralizing activity (total titer of neutralizing antibodies);
- IFN-γ (interferon level IFN-γ in T-lymphocyte culture after restimulation with SARS-CoV-2 specific antigen (glycoprotein S or RBD)) at the first stage;
- The number and ratio of CD4+ and CD8+ cells (in response to mitogen stimulation) at the first stage.

4.2.5.5 Assessment of vital signs

The determination of the main vital signs (measurement of heart rate, respiratory rate, blood pressure, body temperature) is performed before the physical examination at rest (after 15 minutes of rest, in a sitting or lying position). Heart rate is measured during auscultation of the heart in parallel with determining the pulse rate on the radial artery (or on the carotid artery with a weak pulsation on the radial artery) for a minute in a sitting position, in case of a pulse deficit, both parameters are recorded: heart rate and pulse rate. Respiratory rate is measured for a minute at rest in the supine position, noting the respiratory movements of the chest or abdominal wall, without attracting the attention of the volunteer.

Blood pressure measurement will be performed on the brachial artery with the volunteer sitting in the Korotkoff position using a certified tonometer using a cuff of length and width matched to the length and circumference of the volunteer's upper arm.

4.2.5.6 Electrocardiography

The ECG will be conducted in accordance with the Examination Schedule (Section 4.2.4). The procedure includes recording an electrocardiogram in 3 standard and
3 enhanced leads from the extremities, as well as 6 chest leads at rest in the supine position. The interpretation of the ECG will be carried out by a cardiologist, therapist, or functional diagnostics doctor. If there are ECG changes, the Investigator should assess their clinical significance.

4.2.5.7 Pulse oximetry

Pulse oximetry will be performed to determine blood oxygen saturation (SpO2). The procedure will be performed using a pulse oximeter according to the standard procedure in accordance with the Study Procedures Schedule (Section 4.2.4).

4.2.5.8 Unscheduled visits in case of SARS-CoV-2 infection

In the case of laboratory and/or instrumental confirmed (or suspected) COVID-19 disease, additional unscheduled COVID-19 disease monitoring visits may be conducted through telephone contact. Infection with COVID-19 is a reason to exclude a participant from the vaccination program (Section 5.3), however, since this study also assesses the effectiveness of vaccination, the volunteer is not completely excluded from the study, but additional information is collected on the course of COVID-19 in this volunteer and his outcomes. Treatment of COVID-19 will be carried out ***in full accordance with*** standard practice (the latest version of the current interim guidelines for the prevention, diagnosis and treatment of COVID-19) in the community.

The investigating physician should collect all the necessary information to evaluate the efficacy parameters defined in this study. If it is not possible to contact the volunteer, the investigator should try again to contact the patient or a trusted contact, if available.

If a coronavirus infection is detected, this event will be assessed as an AE. In the event that a volunteer is hospitalized for coronavirus infection, this event will be considered an SAE, the investigator should collect detailed information about this SAE.

In case of COVID-19 disease monitoring visits, defined as unscheduled, the following procedures are recommended (examination and taking of biological samples for laboratory research is carried out only in case of a face-to-face visit):

- Collection of anamnesis, including the collection of data on complaints and on all studies (laboratory and instrumental) conducted by the volunteer outside the scope of this clinical study, and the treatment received;
- •Obtaining and analyzing data from a paper diary of a volunteer;
- Registration of concomitant therapy;
- Assessment of AE/SAE.

4.2.5.9 Study termination visit

During the visit, the following procedures/examinations should be carried out:

- Collection of complaints;
- Physical examination;
- Measurement of vital signs;
- Determination of SARS-CoV-2 RNA by PCR (nasal and/or oropharyngeal swab);
- IgG to SARS-CoV-2 spike (S) protein;
- Clinical blood test;
- Obtaining and analyzing data from a volunteer's diary;
- Checking the diary;
- Registration of concomitant therapy;
- Assessment of AE/SAE.

4.2.1 Handling of biological samples

A complete chain of custody must be guaranteed for all samples throughout their life cycle.
The investigator, or his designee at each center, ensures that biological samples obtained from participants held at the center are fully traceable to shipment or disposal (when applicable), and records relevant information about the handling of samples while they are at the center. Information about storage violations must be documented and reported to an authorized representative of the sponsor. The sample taker maintains full traceability of the samples during storage and use until they are used, disposed of or sent onward, and maintains a record of receipt and subsequent shipment or disposal.
Procedures for handling biological specimens will be described in the Laboratory Manual for each type of biological specimen.

4.2.2 Withdrawal of informed consent for submitted biological samples and data

All biological samples and data that were collected prior to the withdrawal of informed consent should be analyzed and included in the overall data assessment.

4.2.3 Monitoring compliance with procedures

For the purposes of this study, it is the responsibility of the subject and the investigator to adhere to the schedule prescribed by the protocol, as well as to comply with the rules for the preparation for the study, including adherence to food intake prescriptions (it is important to indicate to the volunteer the need to limit the intake of fatty foods 3 days before each visit to ensure high-quality biological collection material for laboratory testing), abstinence from alcohol and restrictions on concomitant therapy. Since all of these procedures must be described in the primary documentation and CRF, control over the observance of research procedures by volunteers is carried out by the researcher.

The following allowable deviations are established for the timing of the procedures (which do not require documentation as a deviation from the protocol):

visit / examination, biomaterial sampling	permissible variation
day 1<br>examination<br>30 min<br>2 hours<br>6 hours<br>12 hours<br>after drug administration	 <br>10 min<br>30 min<br>30 min<br>1 hour
day 2<br>examination<br>24 hours<br>36 hours<br>after drug administration	 <br>2 hours<br>2 hours
examination after 48 hours	Examination in the morning upon discharge from the hospital at any time at the discretion of the investigator's physician
days 7–28	2 days
days 42–90	3 days
180 days	14 days
270–365 days	14 days

In case of more significant deviations, a protocol deviation form should be completed.

4.3 Duration of participation of volunteers in the study

The duration of participation of volunteers in the study will be 53 weeks, including 1 week of screening, 3 weeks of therapy and 49 weeks of follow-up.

4.4 Rules for Stopping Parts of a Clinical Study and/or the Clinical Study as a Whole

Adolescents will be included in dose groups (1/10 or 1/5) as informed consents are signed. Hospitalization is carried out in stages of 10–20 people. At the beginning, older teenagers 15-17 years old will be included, then younger ones 12–14 years old. Day 3 safety analysis of the AE identified at the time of analysis and the possibility of moving to the next age group is carried out by the Principal Investigator(s) and submitted to the IDMC for consideration. The meeting can be held online.

With a favorable safety profile after 7 days after the full dose, the Principal Investigators make a consolidated decision to escalate the dose to 1/5 of the adult dose. Safety analysis on day 7 for the AE identified at the time of analysis and the possibility of moving to the next dose group is carried out by the Principal Investigator(s) and submitted to the IDMC for consideration. The meeting can be held online.

The criteria for transition to vaccination of subsequent volunteers will be:

- absence of SAE, the relationship of which with vaccination is defined as “probable” or “certain”
- absence of potentially life-threatening AE (class 4 according to the classification given in Appendix 3), the relationship of which with vaccination is defined as
- "probable" or "certain".
- absence of severe AE (class 3 according to the classification given in Appendix 3), in more than half of the volunteers after the first administration of the vaccine, the relationship of which with vaccination is defined as "probable" or"certain".

If 1/10 of the dose is considered unsafe, the study will be stopped, the second dose will not be given.

If 1/5 of the dose is deemed unsafe, the second dose will not be given.

If both doses show an acceptable safety profile, the choice of dose for continuation of the study will be made based on the results of the study of immunogenicity.

<u>An interim report on the 1st stage of the study on day 28 will be submitted to the Russian Ministry of Health.</u>

The transition to the next stage is planned based on the report on the safety and tolerability of ED on day 28 after administration of the first component. The adjusted dose will be determined based on day 28 immunogenicity, which should be consistent with immunogenicity in adults after receiving a full dose of ED.

The study may be stopped for the following reasons:

1. At the initiative of the sponsor:
   a. obtaining new toxicological or pharmacological data, or data on SAE that force a review of a previously conducted benefit/risk assessment of participation in the study;
   b. the frequency of AE and/or their severity does not allow continuing the study;
   c. other reasons, including administrative ones.
2. Investigator-initiated: The frequency and/or severity of AE unacceptably increases the risk of participating volunteers in a study.
3. By decision of the regulatory authorities.
4. By decision of the IDMC

If the study is terminated prematurely, the sponsor must notify the study site staff and regulatory authorities, indicating the reason for the premature termination of the study.

Study termination rules for each study participant are listed in Section 5.3.

5 Study population

5.1 Inclusion criteria

The study will include volunteers who meet all of the following criteria:

1. Written informed consent of the subject of the study and the parents/adoptive parents of the subject to participate in the study;
2. Boys and girls aged 12–17 inclusive;
3. Negative test result for HIV, hepatitis, syphilis;
4. A negative result of the study for the presence of IgM and IgG antibodies against SARS CoV2 by enzyme immunoassay, and, if available, the result of a survey performed in medical organizations of the Moscow Health Department 7 days before inclusion in the study can be taken into account;
5. A negative test result for COVID-19, determined by PCR at the screening visit, and, if available, the result of a survey performed in medical organizations of the Moscow Health Department 7 days before inclusion in the study can be taken into account;
6. No history of COVID-19;
7. Absence of contact of the subject of the study with patients with COVID-19 for at least 14 days prior to inclusion in the study (according to the study participant, parents/adoptive parents of the subject);
8. Consent to the use of effective methods of contraception during the entire period of participation in the study;
9. Negative pregnancy test based on the results of a urine test at the screening visit (for all female participants in the study);
10. Negative test for the presence of narcotic and psychostimulant drugs in the urine at the screening visit;
11. Negative alcohol test at screening visit;
12. No history of severe post-vaccination reactions or post-vaccination complications after the use of immunobiological preparations;
13. Absence of acute infectious and / or respiratory diseases for at least 14 days before enrollment in the study.

5.2 Exclusion Criteria

Volunteers cannot be included in the study if at least one of the following exclusion criteria is met:

1. Any vaccination/immunization given within 30 days prior to enrollment in the study.
2. Therapy with steroids (with the exception of hormonal contraceptives) and / or immunoglobulins or other blood products that did not end 30 days before enrollment in the study;
3. Immunosuppressive therapy and systemic corticosteroid therapy completed in less than 3 months. prior to inclusion in the study.
4. Postponed less than one year prior to enrollment in the study, acute coronary syndrome or stroke
5. Any immunodeficiency (for example, hereditary immunodeficiency, acquired immunodeficiency syndrome [AIDS], etc.).
6. Infectious diseases:
   - history of HIV (antibodies against HIV type 1 or 2), hepatitis (positive test for HBsAg or antibodies against HCV), active syphilis;
   - Tuberculosis;
   - Active infection (excluding onychomycosis) or any major episode of infection requiring intravenous antibiotic treatment within 4 weeks prior to screening or oral within 2 weeks prior to screening;
   - History of a serious recurrent or chronic infection not listed above.
7. Major surgery within 4 weeks prior to screening.
8. Chronic autoimmune diseases or systemic collagenoses in history, requiring the appointment of immunosuppressive therapy.
9. Volunteers who have undergone organ transplantation, including bone marrow transplantation or peripheral blood stem cell (PBSC) transplantation, and are receiving immunosuppressive therapy.

10. Splenectomy in history.
11. Volunteers with a previous or concomitant history of neoplasms (ICD codes C00-D09).
12. Aggravated allergic history (history of anaphylactic shock, Quincke's edema and other life-threatening conditions), hypersensitivity or allergic reactions to the administration of immunobiological preparations, known allergic reactions to any of the components of the vaccine or a vaccine containing similar components, exacerbation of allergic diseases on the day of enrollment in the study.
13. Neutropenia (decrease in the absolute number of neutrophils less than 1000 cells / mm3), agranulocytosis, significant blood loss, severe anemia (hemoglobin concentration less than 80 g / l), thrombocytopenia (decrease in the absolute number of platelets less than 50,000 cells / mm3).
14. Anorexia, protein deficiency of any origin.
15. Volunteers with a BMI value in the range from -2 SD to +2 SD, according to age (Appendix 4).
16. Extensive tattoos at the sites of drug injection (deltoid muscle area), which do not allow assessing the local reaction to ED administration.
17. Chronic diseases of the cardiovascular, bronchopulmonary, neuroendocrine systems, as well as diseases of the gastrointestinal tract, liver, kidneys, muscle and connective tissue in the stage of exacerbation or decompensation.
18. The presence or suspicion of drug, alcohol or drug addiction and other mental disorders.
19. Medical conditions that, from the investigator's point of view, put the participant's health at risk if they participate in the study or potentially make it difficult to interpret the results of the study.
20. Family members of the staff of research centers directly involved in the conduct of the study and.
21. Participation in other clinical trials and use of other investigational drugs within 28 days prior to screening.
22. Planned vaccination against COVID-19 with any vaccine, both as part of other studies and as part of a civil circulation.
23. Female subjects during pregnancy or lactation
24. Inability to read Russian; inability or unwillingness to understand the essence of the study. Any other condition that limits the eligibility of obtaining informed consent or may affect a volunteer's ability to participate in the study will affect the volunteer's ability to participate in the study.

5.3 Exclusion Criteria

1. Refusal of the volunteer from further participation in the study

Non-compliance by the volunteer with the rules for participation in the study.

2. Occurrence of causes / occurrence during the study of situations that threaten the safety of the volunteer (for example, hypersensitivity reactions, etc.).

3. Volunteers selected to participate in the study in violation of the inclusion / non-inclusion criteria.

4. Vaccination with any vaccine for the prevention of coronavirus infection (COVID-19) both in clinical trials and in the framework of civil circulation

5. Positive pregnancy test in female subjects.

6. Occurrence in the course of the study of other reasons that impede the conduct of the study according to the protocol.

In the event of an SAE or pregnancy, volunteers are excluded from the continuation of the vaccination program, but they continue to be observed in accordance with the rules described in Section 8 of the current protocol. In case of infection with SARS-CoV-2 (laboratory and/or instrumentally confirmed diagnosis), the volunteer is excluded from the vaccination program, but not from the study. Once disease is identified, there are no further standard study visits, but at least one ad hoc visit for SARS-CoV-2 infections (Section 4.2.5.8).

5.4 Procedure and conditions for the inclusion of volunteers

Volunteers meeting the selection criteria in accordance with the study protocol will be sequentially included in the study of the first stage, first the oldest group of 15–17 years old (the first about 20-25 people) then the youngest group of 12–14 years old. The interval between inclusions of groups is 3 days.

The selection and recruitment of volunteers to participate in the study is carried out as follows:

1. The collection of information about those wishing to participate in a clinical trial is carried out in the children's polyclinics of the city of Moscow by posting information about the planned study in the polyclinics and informing the doctors of the polyclinics about the planned study.
2. Adolescents may be included in the study if they or their parents/adoptive parents apply directly to the N.F. The data in this case is transferred to research organizations.
3. On the official websites of clinics that plan to participate in a clinical trial, an announcement is posted on the recruitment of volunteers to participate in a clinical trial. Volunteers and their parents/adoptive parents can contact study coordinators or research physicians at the contacts listed in the announcements.

Volunteers will be included in the Phase 2 study as informed consents are signed.

Information materials, including announcement texts, for physicians, volunteers and their parents/adoptive parents should be submitted for approval to the local ethics committee (LEK) or the Moscow City Ethics Committee (MGEC).

5.5 Compliance control

Vaccination is carried out in the conditions of the research center, the procedure for of ED administration is carried out by medical personnel. In this regard, 100% compliance is assumed, compliance control is not provided.

5.6 Authorized concomitant therapy and contraception

In this study, non-steroidal anti-inflammatory drugs and antihistamines are allowed.
Medicines for the treatment of AE, as well as emergency medicines (if necessary) will be prescribed in accordance with the approved instructions for use of the medicinal product for medical use and the ATC pharmacotherapeutic group recommended by WHO, as well as taking into account the method of administration and use of the medicinal product. The regimen for prescribing other drugs will be selected according to the recommendations for the use and dosages of this drug. These drugs must be approved for use in the territory of the Russian Federation.
All therapy performed will be recorded in the eRCI and the primary documentation of the volunteer.
During the post-administration follow-up phase, volunteers are required to inform the investigator of any medications taken during the study. The use of concomitant medications should be documented in the eRCI, which specifies the substance, dose, timing, and reason for the use of concomitant medications.

The facility where the clinical trial is being conducted must have emergency medical facilities available. In the case of emergency medical care, all the measures taken and the necessary medications used must be recorded in the primary documentation and eRCF.
Contraception For sexually active adolescents, contraception should be provided by one of the following methods: abstinence, intrauterine device, oral/injectable contraception, subcutaneous implant, or a double barrier method (eg, condom with a local contraceptive). “Selected practice recommendations for contraceptive use” – 2nd ed., World Health Organization, 2005; ISBN 92 4 156284 6 (NLM classification: WP 630). The document was translated into Russian by the Reproductive Health and Research Program of the WHO Regional Office for Europe under the Strategic Cooperation Program between WHO and the United Nations Population Fund.

5.7 Concomitant and prior therapy, lifestyle

In this study, it is forbidden to use any immunotropic drugs (immunosuppressants, immunomodulators), steroids (with the exception of the use of corticosteroids for emergency care, contraceptives) and / or therapy with immunoglobulins or other blood products that has not been completed 30 days prior to study entry. It is not allowed to use any vaccination 30 days before enrollment in the study and within 30 days after the second vaccination, then vaccination is allowed in accordance with the regional vaccination schedule.

Transfusion of blood and its components, plasmapheresis and donation are not allowed during the entire study. The use of immunoglobulins, preparations of monoclonal antibodies, interferons, colony-stimulating factors, growth factors is not allowed. If the need arises for therapy with the above drugs and procedures, the volunteer should be withdrawn from the study.

In the event that it becomes necessary to prescribe such drugs to a volunteer for health reasons, the volunteer will be withdrawn from the study with subsequent follow-up.

It is not allowed to take phytopreparations and biologically active food supplements during the entire study.

Vaccination with any other COVID-19 vaccines is not permitted, including in other clinical trials.

6 Information about the medicinal products used in the clinical trial

6.1 Experimental drug

Experimental drug:
Gam-COVID-Vac M Combined vector vaccine for the prevention of coronavirus infection caused by the SARS-CoV-2 virus
Comparator: not provided

6.2 Measures to minimize subjectivity

A double-blind, placebo-controlled, multicenter, parallel-group clinical trial is planned. Randomization is provided in a ratio of 4:1.
The production and labeling of the vaccine and placebo is carried out in a way that eliminates confusion in production and does not allow to distinguish between the drug and placebo externally.

6.3 Experimental drug regimen

As part of this study, the drug will be administered at 1/10 and 1/5 of the immunizing dose ($1*10^{11}$ viral particles): 10^{10} and 2×10^{10}, respectively, intramuscularly into the deltoid muscle of the shoulder. The drug is administered twice with an interval of 21 days (first component 1, then component 2).

6.4 Dosage form, packaging and labeling of the investigational product

For the purposes of the clinical study, drugs from experimental batches will be used. In addition to the factory label containing information about the name, dosage form, route of administration, composition, batch number, expiration date and manufacturer of the medicinal product (name and contact information of the sponsor of the study), each package will be pasted with a sticker indicating the information required in accordance with the Order Ministry of Industry and Trade of Russia dated 14.06.2013 N 916 "On Approval of the Rules of Good Manufacturing Practice"

This study is a Phase III study, and the ED used is registered. The study is conducted using an open design to expand the population that is allowed to use the ED.

6.5 Investigational medicinal product accounting procedures

An ILS may only be located at a center approved for this study. The Principal Investigator must ensure that the drug is stored in appropriate conditions to prevent loss, theft and violation of storage conditions in accordance with the draft instructions for medical use, and the requirements of the Federal State Budgetary Institution “National Research Center for Epidemiology and Microbiology named after N.N. N.F. Gamaleya" of the Ministry of Health of Russia.

The Principal Investigator should appoint a responsible person to control the storage conditions of the drug and record its consumption.

Control and accounting of drug consumption should be documented. The ED can only be assigned to study participants.

6.6 Storage of randomization codes and procedures for their disclosure

6.6.1 Distribution by study group

Not applicable for the first stage. The vaccination scheme in the groups will be the same.

The second stage is envisaged as a double-blind, placebo-controlled study. All study subjects who meet the inclusion/exclusion criteria and successfully complete all screening procedures will be assigned to two study arms by stratified IWRS randomization in a 4:1 ratio to the study group (using Gam-COVID-Vac M Combined Vector Vaccine for Prevention coronavirus infection caused by the SARS-CoV-2 virus") and in the control group (using placebo). Randomization of study subjects will be carried out in two age strata: 12–14 and 15–17 years.
Study subjects will be assigned unique randomization numbers that will remain the same throughout the study.
Investigating physicians will maintain center-specific registers with screening and randomization numbers of study subjects, as well as a register of identification codes of study subjects with full names. At the same time, medical researchers are obliged to comply with all legal requirements for the protection of personal data. The study subject identification code log will only be kept at the study site and will not be shared with the study organizer.

6.6.2 Revealing randomization codes

The present study is a double-blind study: blinded for the study subject and the study physician.

At the initiation visit, the investigators will be informed about the randomization code disclosure and blinding procedure through the IWRS system. Code disclosure is recommended in a medical emergency for reasons relating to the safety of the research subject at the discretion of the researcher's physician. The reason for the disclosure of the code must be described in detail in the primary documentation of the research subject.

After the disclosure of randomization codes, the subject of the study can be informed about which group he was included in - vaccines or placebo.

The disclosure of codes will be made after the 28th day of the study.

7 Assessment of immunogenicity parameters

Assessment of immunogenicity:

The following indicators of immunogenicity will be assessed in volunteers:

a) Indicators of the intensity of the humoral immune response:

- Geometric mean titer of IgG antibodies against SARS-CoV-2 spike protein;
- Percentage of volunteers with detected specific IgGs;
- Geometric mean titer of neutralizing antibodies against the SARS-CoV-2 virus;
- Percentage of volunteers with detected neutralizing antibodies.

b) Indicators of the intensity of the cellular immune response:

- An increase in the concentration of interferon gamma in a culture of T-lymphocytes after restimulation with a specific SARS-CoV-2 antigen (glycoprotein S or RBD);
- Level of proliferative activity of CD4+ and CD8+ cells after restimulation with SARS-CoV-2 specific antigen (glycoprotein S or RBD).

Immunogenicity parameters can be compared with those of healthy volunteers in the Gam-COVID-Vak clinical trials at the same time.

8 Evaluation of safety parameters

8.1 List of safety parameters

The duration of participation of volunteers in the study will be 53 weeks, including 1 week of screening, 3 weeks of therapy and 49 weeks of follow-up. For placebo volunteers, the study will be extended by 28 days to unblind and the period of unblind procedures by a total of no more than 2 months.

The safety assessment will be based on the registration of AE within 42 days and SAE during the entire follow-up period.

In order to prevent the development of post-vaccination reactions and complications, volunteers are screened to determine the initial indicators of the state of the body. In addition, volunteers will undergo a medical examination. All baseline indicators (examinations before drug administration) are entered into the electronic individual registration card (eIRC) of volunteers.

Throughout the vaccination period and during the follow-up period, safety information will be collected on the following parameters:

- development, severity and relationship with administration of the two components of the drug Gam-COVID-Vak M Combined vector vaccine for the prevention of coronavirus infection caused by the SARS-CoV-2 virus of all SAE during the entire study;

- development, severity and relationship with administration of the two components of the drug Gam-COVID-Vac M Combined vector vaccine for the prevention of coronavirus infection caused by the SARS-CoV-2 virus of all AE during 42 days of the study
- the frequency and severity of local signs and symptoms, the relationship with the use of the drug of general signs and symptoms after drug administration;
- data of physical, laboratory and instrumental studies;
- reactogenic properties are assessed in terms of local (area of the injection site) and general (systemic reactions of the body to the administration of the drug) reactions.

Local reactions are evaluated on the following grounds:
- hyperemia at the injection site;
- edema (infiltration);
- pain
- increase in regional lymph nodes;
- rash;
- itching.

Common post-vaccination reactions include: fever, feeling unwell, headache, dizziness, decreased appetite, insomnia, nausea, vomiting, dyspepsia, weakness, sweating, muscle and joint pain, abdominal pain, convulsions, etc. In addition, allergic reactions are possible. reactions.

Accounting for local and general reactions is carried out by the researcher on the basis of body temperature measurement, examination and questioning of the volunteer, conducted in accordance with the schedule of visits and research procedures.

Changes in laboratory parameters (clinical and biochemical blood tests), changes in vital signs (blood pressure [BP], heart rate [HR], respiratory rate [RR], body temperature) will also be assessed.

When assessing, it should be borne in mind that there are no pathognomonic symptoms that would unambiguously consider each specific case of AE associated with vaccination. Such clinical symptoms as high fever, intoxication, neurological symptoms, various types of allergic reactions, including the immediate type, may not be due to immunization, but to a disease that coincided with the vaccination. Therefore, each case of the disease that developed in the post-vaccination period requires careful differential diagnosis with both infectious and non-infectious diseases using instrumental and laboratory research methods, based on the clinical symptoms of the disease.

One of the criteria is the time of occurrence. As a rule, the symptoms of post-vaccination reactions develop no later than 48 hours, while the duration of fever for more than 72 hours or its occurrence after this time indicates the possible presence (attachment) of infection.

8.2 Methods and timing for assessing, recording and analyzing safety parameters

The methodology for assessing key safety parameters (including physical examination, laboratory and instrumental examinations) is discussed in detail in Section 4.2.5. Safety assessment will be carried out throughout the study (see scheduled visit procedures).

It should be taken into account that adolescent volunteers are a group in which a significant number of endocrine and autoimmune diseases manifest. This should be taken into account when assessing diseases that coincided with vaccination and investigation of cases should be carried out with the involvement of narrow specialists to make a reasonable conclusion about the likelihood of a disease being associated with vaccination.

8.3 Requirements for reporting, registration and reporting of adverse events and intercurrent diseases

8.3.1 Definition of adverse events and serious adverse events

An adverse event (AE) is any adverse change in the state of health of a research subject who has been administered a medicinal product, regardless of the causal relationship with its use. An AE can be any adverse and unintended change (for example, abnormal laboratory value), symptom or disease, the timing of which does not preclude a causal relationship with the use of a medicinal product, regardless of the presence or absence of a relationship with the use of a medicinal product;

An SAE is any medically adverse event that:

- led to death;
- represents a threat to life;
- leads to persistent or severe disability or disability;
- represents a congenital anomaly or malformation;
- requires hospitalization or its extension;
- requires medical intervention to prevent the development of these conditions.
- is medically significant: deciding whether rapid reporting is appropriate in other situations (for example, in cases of medically important events that do not pose an immediate threat to the life of the research subject, do not result in death or hospitalization, but puts the subject at risk or requires intervention to prevent one of the outcomes listed above) is based on medical and scientific judgment. Such cases should usually also be considered SAE.

If a coronavirus infection is detected, this event will be assessed as an AE. If a positive PCR test is detected without clinical manifestations, the AE will be recorded as a laboratory deviation. If a volunteer is hospitalized for coronavirus infection, this event will be regarded as an SAE, the investigator should collect as much detailed information as possible about this SAE (examinations performed, disease severity, therapy received, outcome).

When describing an AE, the term "severity" is used to describe the intensity of a particular event and is not synonymous with the term "severity". Reports that add significant information about the specificity, increase in frequency or severity of known, already documented serious adverse reactions (side effects) are AE reports.

Information about side effects already known for this investigational product is contained in the Investigator Brochure. This information will be included in the volunteer information sheet and should be discussed with research subjects.

Life threat

“Life-threatening” means that the participant is at immediate risk of death from the resulting AE, or that the use or continued use of the medicinal product may be expected to result in death of the participant. “Life-threatening” does not mean that if the AE were to become more severe, it could cause death.

Hospitalization

Outpatient treatment in an emergency room setting is not in itself a serious AE, although the reasons for such treatment may be serious AE (eg, bronchospasm, laryngeal edema). Hospitalizations and/or surgical interventions planned prior to or during the study are not considered AE if the underlying disease was present prior to enrollment in the study, unless the condition worsened unexplained during the study.

Important medical event or medical intervention

Medical and scientific expertise should be used to assess whether a case is serious in situations where an important medical event may not pose an immediate threat to life or result in death, hospitalization, disability or incapacity for work, but may put the participant at risk or require medical intervention to prevent one or more of the outcomes listed in the severity definition. As a rule, such phenomena should be considered as serious.

8.3.2 Adverse events of particular interest

Of particular interest to researchers is the likelihood of developing allergic reactions, inflammatory and infectious diseases after vaccination, in particular infections caused by herpes viruses.

8.3.3 AE intensity

The term "severity" is used to describe the intensity of a particular event. It is important to distinguish between serious and severe AE. Severity is a measure of intensity, while severity is determined based on severity criteria (SAE criteria section 8.3.1). Throughout the study, the Investigator will identify AE and classify them according to intensity as follows:

- Mild AE: transient or mild discomfort that does not affect the subject's daily activities; no medical intervention/therapy required;
- Moderate AE: mild to moderate activity limitation - assistance may be required; no or minimal medical intervention/therapy required;
- Severe AE: severe limitation of activity, as a rule, outside assistance is required; medical intervention / therapy is required, hospitalization is possible; any SAE.

8.3.4 Causal relationship to investigational product

The association of AE with the study drug will be assessed using the WHO scale:

Certain	Clinical manifestations of AE, violations of laboratory parameters occur during the period of taking the drug, cannot be explained by the presence of existing diseases and the influence of other factors. AE manifestations regress after discontinuation of the drug and reappear upon re-appointment

Probable	Clinical manifestations of AE, violations of laboratory parameters are related in time to the drug intake, are unlikely to be related to concomitant diseases or other factors, regress with drug withdrawal. Response to re-administration of the drug is unknown
Possible	Clinical manifestations of AE, changes in laboratory parameters are time-related with taking the drug, but they can be explained by the presence of concomitant diseases or the use of other drugs and the influence of chemical compounds. Information about response to drug withdrawal is unclear
Doubtful	Clinical manifestations of AE, changes in laboratory parameters occur in the absence of a clear temporal relationship with the medication; there are other factors (drugs, diseases, chemicals) that may be causing them
Conditional	Clinical manifestations of AE, violations of laboratory parameters referred to as AE, are difficult to assess. Additional data needed for evaluation or this data is currently being analyzed
Indefinite	Suspected AE reports cannot be assessed as there is no sufficient information, or it is contradictory

Guidance on interpreting the question of causality

The following parameters should be analyzed and evaluated:

- Time dynamics. Does the occurrence of the AE have an acceptable temporal relationship with drug administration?

- Compliance with the known drug profile. Is the AE consistent with the available information about the suspect drug (pharmacology and toxicology) or about drugs in the same pharmacological class? Or can an AE be expected based on the pharmacological characteristics of this drug?
- No alternative cause. The AE cannot be adequately explained by other causes, such as the underlying disease, other medications, other factors related to the patient's health status, or environmental factors.
- Laboratory indicators. The presence of a connection is confirmed by a certain laboratory indicator (if the analysis was performed before administration and after the onset of the AE).

In complex cases, other factors may be considered, such as:

- Is this phenomenon consistent with an overdose of study drug?
- Is there a known mechanism for its development?

A "related" rating is assigned if, after analysis of the relevant data, it is determined that there is a "reasonable likelihood" of a causal relationship for a particular case. The wording "reasonable probability" for causation means that, in general, there are facts (evidence) or arguments that indicate the existence of a causal relationship.

An assessment of causality is made on the basis of available data, including sufficient information to form a conclusion. In the absence of facts or arguments to suggest a causal relationship, the event will be rated "not related".

8.3.5 Documentation of adverse events

Any AE (non-serious and serious) occurring from the time the subject provided study-specific informed consent to the completion of the study should be documented in the primary medical records and on the designated eRCF pages.

To identify AE, research subjects should be asked leading questions at each study visit. AE may also be identified if subjects self-report them during or between visits, and if they are detected during an examination, laboratory tests, or other assessments.

Actions taken to treat AE should be documented and categorized as "no action taken", "medication (concomitant therapy)", or "other treatment". In addition, the actions taken on the investigational product should be documented and classified into one of the following categories: "no change", "withdrawal", "dose reduction", "dose increase", "treatment suspension", "action unknown", and "not applicable". Concomitant therapy, other treatments, or changes in study drug use should also be documented.

Medical conditions/diseases that existed prior to the start of the study drugs are considered AE only if they worsen after the subject was included in the study. Abnormal laboratory values or other findings are AE only when they cause clinical signs or symptoms, are considered clinically significant, or require therapy.

AEs should be assessed at each visit (or more frequently if indicated) for any changes in severity, suspected association with study drug, interventions taken to treat it, and outcome.

During and after the subject's participation in the study, the investigator (health care facility) shall ensure that the study subject receives the necessary medical attention in the event of any study-related adverse events, including clinically significant changes in laboratory parameters. The researcher (medical institution) is obliged to inform the research subject about intercurrent diseases that have become known to the researcher and require medical attention.

AEs between informed consent and first dose of study drug

In the interval between the signing of informed consent and the use of the first dose of the drug,

registration of SAE is provided. A volunteer with an SAE prior to drug administration is excluded from the study.

AEs after first dose of study drug

The investigating physician should observe all AE that have occurred since the first dose of the drug and during the study, until the AE resolves or is regarded as chronic or stable.

SAE ongoing at the last visit

If the SAE continues at the last visit, the Investigating Physician will continue to follow up until the SAE resolves or stabilizes/ becomes chronic.

The investigator must submit SAE follow-up reports in accordance with Section 8.3.6 SAE Reports below.

Clinically significant abnormal laboratory values at the last visit

The Investigator should document all abnormal laboratory findings at the last visit as AE, even if no relationship to the specific study drug can be found.

8.3.6 Teports of serious adverse events

All SAE must be reported to the Sponsor immediately, except for those SAE that are identified in the protocol or other document (such as the Investigator's Brochure) as not requiring urgent reporting. After the initial urgent communication, a detailed written communication must be submitted as soon as possible. Initial and follow-up reports should identify study subjects by their assigned unique codes, and not by subject names, personal identification numbers, and/or addresses.

All adverse events and/or laboratory abnormalities identified by the protocol as critical for safety assessment must be reported to the Sponsor in accordance with the requirements for reporting adverse events within the timeframe specified by the Sponsor in the protocol.

It is critical that the Investigating Physician promptly (i.e., within 24 hours of notification of an SAE) report any serious AE, even if the Investigating Physician does not consider the SAE related to study medications.

To report an SAE, the Investigator must use the Sponsor's Serious AE Reporting Form as an initial or follow-up report. The form is faxed or emailed to the sponsor's representative with a copy to the study manager using the contact information provided in the table below.

The Investigating Physician should also forward any updates/new information in a new "SAE Reporting Form" as a follow-up to a previously reported SAE. Follow-up information should describe whether the event resolved or is ongoing, if a diagnosis was made, if treatment was given and, if so, what kind, and whether the subject is still in or out of the study.

Recurring episodes, complications, or progression of the initial SAE should be reported as a follow-up to the original episode, regardless of when the event occurs.

Any new SAE that is considered completely unrelated to a previously reported SAE should be reported as a new and separate occurrence.

Any questions from the Sponsor regarding SAE reports must be answered by the Investigator within 24 hours.

The Investigating Physician should keep notifications of delivery of information to all recipients in the Investigator's file for the study.

Reporting to health authorities

The Sponsor submits all reports by the due date to all concerned regulators. Serious unanticipated adverse reactions are subject to reporting to regulatory authorities.

Investigator training

By signing the study protocol, the Principal Investigator certifies that he/she has completed the training provided and is aware of the Sponsor's requirements and his/her obligation to report SAE/AEs and pregnancies as defined in the study protocol.

8.4 Pregnancy

The Investigating Physician must promptly (i.e., within 24 hours of reporting the pregnancy) to the Sponsor, with a copy, the manager in charge of the study, as described in Section 8.3.6, of all pregnancies in study participants and study participants' partners, that occurred during the clinical study. Reports of cases of pregnancy are recorded only from the moment of the first administration of the drug.

The investigating physician should immediately exclude the pregnant participant from further participation in the study and monitor the pregnancy to determine its outcome, including spontaneous or planned abortion, details of delivery and the presence or absence of any malformations, congenital anomalies in the newborn, or complications in the mother.

Pregnancies should be reported on the Clinical Trial Pregnancy Reporting Form. Additional information about pregnancy should be recorded on the same form and should include the possible relationship of any pregnancy outcome to study drug use.

Any SAE noted during pregnancy must be reported using the SAE Reporting Form.

8.5 Method and duration of follow-up of clinical study participants after the occurrence of adverse events

Once an AE is identified, it should be monitored until it resolves or is considered permanent. The outcome of the event should be documented and classified into one of the following categories: "the event did not resolve/has not changed", "the condition worsens", "the event resolved/the subject recovered", "the event improves/the subject recovers", "the event resolved/the subject recovered with complications ", "fatal" or "outcome unknown".

9 Statistical aspects of the clinical study

9.1 Description of statistical analysis methods

Statistical analysis will be carried out using specialized software, the final choice of which will be made at the stage of finalizing the statistical analysis plan.

Safety and immunogenicity analyzes will be conducted separately for the 12–14 and 15–17 age cohorts.

Continuous (quantitative) data will be presented as number of observations, arithmetic mean, standard deviation, median, interquartile range, minimum and maximum. For indicators representing the antibody titer, the geometric mean will be additionally calculated.

Qualitative data will be presented as absolute frequencies (number of observations), relative frequencies (percentages).

Sample estimation accuracy measures, i.e. 95% confidence intervals for the means calculated from the t-distribution (for measures representing antibody titer, the 95% confidence interval for the geometric mean will also be calculated using a log-normal transformation), and 95% confidence intervals for frequencies calculated using the exact binomial Clopper-Pearson method (for dichotomous data) will be presented for the immunogenicity and safety endpoints.

Concomitant and past diseases, allergic history, previous operations and injuries, as well as adverse events will be coded using the MedDRA classifier in the current version at the time of closing the database. For coding prior and concomitant therapy, the ATC classifier in the current version at the time of closing the database will be used, levels 4 and 5 (if available) of coding will be used.

9.1.1 Demographic and other background data (group comparability for analysis)

Volunteers will be included in the study as informed consents are signed. All data obtained in groups 1 and 2 prior to the start of the study drug or (demographic data, laboratory data, data on instrumental and physical examinations, vital signs, etc.) will be compared between groups in order to determine significant differences in the baseline characteristics of the groups in the analysis.

Subject distribution, demographics, and other baseline characteristics will be presented using descriptive statistics for all subjects included in the study (FAS population). Comparison of baseline scores between dose groups will be done using a t-test. For categorical data, a χ2-test or Fisher's exact test will be applied if the expected frequency in any of the cells is less than 5. Descriptive statistics will also be used to present data on comorbidities and concomitant therapy. Concomitant medications will be listed by their INN. Comorbidity data will be coded according to MedDRA.

9.1.2 Analysis of the primary security parameter

The primary goal of this clinical study is to evaluate the safety and tolerability of Gam-COVID-Vac M in healthy volunteers aged 12–17 years.
The primary parameter (endpoint) of safety was the proportion of volunteers with AE and serious adverse events (SAE) that occurred in volunteers within 6 and 12 months after receiving the first dose of the vaccine and that are potentially associated with vaccination.
This endpoint will be presented descriptively according to the descriptive statistics parameters above, and using the exact 95% confidence interval for the proportion. The results will be presented for the entire dataset for safety analysis.
Adverse events (AE) will be coded according to the MedDRA Regulatory Glossary. For each AE/SAE, the absolute number will be given, as well as the proportion of subjects with the development of one or another AE. AE data will be tabulated by organ system and class (SOC) and presented with preferred terms (PT) according to MedDRA in relation to study therapy and severity.
To analyze the frequency of local reactions and abnormalities according to the physical examination, descriptive statistics methods will be used, indicating the absolute number of subjects in n/N format and the proportion of subjects in each category. For intergroup analysis, the χ^2-test or Fisher's exact test will be used if the expected frequency in any of the cells is less than 5.

9.1.3 Analysis of immunogenicity parameters

In order to assess the immunogenicity of Gam-COVID-Vac M in healthy volunteers aged 12–17 years, the following secondary immunogenicity parameters will be analyzed:

1) Geometric mean titer of IgG antibodies against the SARS-CoV-2 spike protein in volunteers before (on the day of injection) and 42 ± 2, 180 ± 14 days after the first dose of the vaccine;
2) The proportion of volunteers with an increase in the titer of IgG antibodies against the SARS-CoV-2 spike protein, before (on the day of the injection), as well as 42 ± 2, 180 ± 14 days after the first dose of the vaccine.

In order to characterize the immunogenicity of the Gam-COVID-Vac M Combined vector vaccine for the prevention of coronavirus infection caused by the SARS-CoV-2 virus, the geometric mean titer of virus-neutralizing antibodies (VNA) in at least 100 study subjects will be analyzed before (on the day of execution injection), as well as 42 ± 2, 180 ± 14 days after the first dose of the vaccine.

In order to describe the state of tension of the cellular immune response when using the drug Gam-COVID-Vak M Combined vector vaccine for the prevention of coronavirus infection caused by the SARS-CoV-2 virus, the level of interferon (IFN) γ in the culture of T-lymphocytes after restimulation with the glycoprotein of the SARS virus will be analyzed -CoV-before administration of the 1st component, as well as 28 ± 2 days after administration of the first dose of the vaccine in volunteers of the 1st stage.

In order to describe the state of the antigen-specific cellular immune response when using the drug Gam-COVID-Vak M Combined vector vaccine for the prevention of coronavirus infection caused by the SARS-CoV-2 virus, the number of proliferating T-helper lymphocytes (CD4+) and cytotoxic lymphocytes (CD8+) will be presented.) in response to mitogen stimulation, determined in at least 50 study subjects on the day of vaccine administration before receiving the first dose of ED and 28 ± 2 days after receiving the first dose in stage 1 volunteers.

Data on IgG antibody titers to the SARS-CoV-2 spike protein within the same group will be analyzed using a paired t-test or Wilcoxon test, depending on the normality of the data distribution (comparison of data obtained at different time periods from the start of vaccination within the same group). An unpaired t-test or Mann-Whitney test will be used to compare antibody levels between different dose groups, depending on the normality of the data distribution. Similarly, the analysis of data on titers of neutralizing antibodies will be carried out. In addition, a mixed model can be used using antibody titer as dependent variable and dose group, evaluation visit, sex and age as fixed factors, and subject number as a random factor.

To analyze data on the concentration of interferon gamma in the culture of T-lymphocytes and the level of proliferative activity of CD4+ and CD8+ cells after restimulation with the specific SARS-CoV-2 antigen, descriptive statistics methods will be used. The data will be presented by dose groups and assessment points. Intergroup comparison will be carried out using a t-test or Mann-Whitney test (depending on the type of data distribution). Within-group comparisons at each assessment visit to baseline will be performed using a paired t-test or Wilcoxon test (depending on the type of data distribution). To assess the normality of the distribution, the Shapiro-Wilk test will be used.

Immunogenicity indicators, if necessary, can be compared with the indicators of healthy volunteers in the Gam-COVID-Vak clinical trials at the same time.

9.1.4 Analysis of other safety, tolerability and reactogenicity parameters

The security assessment will include the following parameters:

• Development, severity and relationship with administration of the two components of the drug Gam-COVID-Vak M Combined vector vaccine for the prevention of coronavirus infection caused by the SARS-CoV-2 virus of all AE during the entire study;

• Frequency and severity of local signs and symptoms, association with the use of the drug of general signs and symptoms after drug administration;

• Data from physical and laboratory studies, including dynamics (clinical and biochemical blood tests), dynamics of vital signs (blood pressure [BP], heart rate [HR], respiratory rate [RR], body temperature);

• Dependence of the severity of AE on the course of the underlying disease and the therapy received;

• Reactogenic properties are evaluated in terms of local (area of the site of drug administration) and general (systemic reactions of the body to drug administration) reactions.

To analyze the frequency of local reactions and abnormalities according to the physical examination, descriptive statistics methods will be used, indicating the absolute number of subjects in n/N format and the proportion of subjects in each category. For intergroup analysis, the χ^2-test or Fisher's exact test will be used if the expected frequency in any of the cells is less than 5.

Quantitative safety data variables (clinical and biochemical blood tests, dynamics of vital signs) will be presented using descriptive statistics methods:

- Number of non-missing values (N);
- Minimum (Min);
- Maximum (Max);
- Arithmetic mean (M);
- Standard deviation (SD);
- 95% confidence interval for the mean (CI);
- Average (Median);
- Interquarter range (IQR).

9.2 Timing of the interim review

An interim analysis will be carried out after data collection on day 28 from the last group of stage 1 and submitted to the Ministry of Health of Russia.

The Interim Report will be submitted to the Independent Data Monitoring Committee (IDMC) for review. After consideration of the report, a decision will be made on the transition to the second stage of the study.

In addition, the study plans to continuously collect safety data, which will be regularly submitted to the IDMC along with information on the progress of the study.

9.3 Planned number of clinical trial participants with justification for sample size

The main purpose of the study is to evaluate immunogenicity and safety based on the development of adverse events. At the first stage of the study, it is planned to select a dose that will have a good safety profile. However, injection site reactions and systemic reactions of mild to moderate severity are expected in this study, AE that prevent further study of the vaccine or severe AE are not expected. The calculation showed that vaccination of 50 subjects in each dose group will detect with a 90% probability at least one AE (reaction at the injection site or systemic reaction) with an expected frequency of at least 5%. At the same time, in total, in two dose groups (100 people), the probability of detecting at least 1 AE will be 90% with an expected frequency of AE of at least 2.5%:

Probability of seeing at least 1 AE based on the expected frequency of AE

Expected AE frequency	Probability of observing at least 1 AE in N volunteers						
	**N=50**	**N=100**	**N=200**	**N=250**	**N=350**	**N=2,400**	**N=3,000**
**0.05%**	2%	5%	10%	12%	16%	70%	78%
**0.1%**	5%	10%	18%	22%	30%	91%	95%
**0.5%**	22%	39%	63%	71%	83%	>99%	>99%
**1%**	39%	63%	87%	92%	97%	>99%	>99%
**1.5%**	53%	78%	95%	98%	99%	>99%	>99%
**2.5%**	72%	92%	99%	>99%	>99%	>99%	>99%
**5%**	92%	99%	>99%	>99%	>99%	>99%	>99%
**10%**	99%	>99%	>99%	>99%	>99%	>99%	>99%

At the second stage of the study, one of the main tasks is to evaluate the immunogenicity parameters for the selected vaccine dose. Therefore, it is planned to include in the second stage a sufficient number of subjects to estimate the proportion of subjects with the presence of seroconversion with an accuracy (90% confidence interval width) of at least 0.05, with an expected seroconversion rate of at least 0.95.
At the second stage of the study, one of the main tasks is to evaluate the immunogenicity parameters for the selected vaccine dose. Therefore, in the second step, it is planned to include a sufficient number of subjects who will donate blood to estimate the proportion of subjects with seroconversion with an accuracy (90% confidence interval width) of at least 0.05, with an expected seroconversion rate of at least 0.95.

At least 500 people (400 vaccine and 100 placebo) will be included in the immunogenicity study for a statistically significant assessment of immunogenicity:

$$N = 3{,}8416 * X * (1-X) / 0{,}0025$$

%	95	90	85	80	75	70	65	60	55	50
number	73	139	196	246	289	323	350	369	381	385

9.4 Applicable level of significance of the clinical investigation

For this study, the analysis is descriptive and will be conducted at a two-tailed significance level of 0.05 without the need for adjustment for multiplicity of comparison.

9.5 Criteria for termination of a clinical trial

The study may be terminated upon the occurrence of the conditions set out in section 4.4 of this protocol. Statistical criteria for termination of the study are not established by this protocol.

9.6 Procedures for handling missing, non-analyzable and doubtful data

During monitoring visits to the clinical site, clinical trial specialists (monitors), authorized by the sponsor, will analyze the eRCI of the study subjects for lack of necessary data. In the absence of data in the eRCF and the availability of relevant information in the primary documentation, requests to researchers and instructions to eliminate inconsistencies will be formulated. Sponsor-appointed statistician and data control managers, when reviewing the database of study results, will conduct an analysis for questionable, missing, and non-analyzable data, which may also result in requests to investigators.

Investigators will, if possible, correct the identified errors in the eRCF and inform the principal investigator and authorized representatives of the sponsor about this. If the identified data errors cannot be corrected after the completion of the participation of volunteers in the study, statistical analysis of the data will analyze the sensitivity of the resulting parameters to the presence of questionable data. Information about missing, doubtful and not subject to analysis data will be presented in the final report of the clinical study.

For all types of endpoints and other parameters, the analysis will be carried out only on actually available information, i.e. without filling in missing or invalid data.

9.7 Procedures for reporting any deviations from the original statistical plan

All deviations from the original statistical plan must be described and justified in an amendment to the protocol and / or the final study report (in the latter case, the statistical analysis plan developed before the start of the final statistical analysis should contain a list of these deviations with justification of the reasons for the deviation from statistical plan according to the protocol).

9.8 Procedures for selecting clinical trial participants for analysis

When conducting statistical analysis, it is planned to study three populations of subjects:

- All subjects included in the study (FAS, full analysis set). This population will be used to describe baseline characteristics, including demographics, anthropometrics, comorbidities, and all immunogenicity endpoints;
- An additional immunogenicity endpoint analysis will be performed on a population of subjects who completed study participation without major deviations from the study protocol and who have valid immunogenicity data at least at one time point after vaccine administration (PP population, Per Protocol);
- All subjects who received at least one component of the vaccine will be included in the analysis of safety parameters.

The PP population will include volunteers who did not experience any of the following events during the course of the study:

- The development of conditions or occurrence of events that meet the exclusion criteria from the study.
- Use of unauthorized concomitant therapy.
- Any other significant violation of the protocol, found to significantly violate the primary assessment of safety and efficacy in this study participant.

10 Description of quality control and quality assurance activities

10.1 Study monitoring and quality control

Regular visits by a clinical research specialist (monitor), on behalf of the sponsor and in accordance with the standard operating procedures (SOP), before the start, during the study and at the end of the study, contribute to the successful conduct of the study and serve as a guarantee of collecting accurate data, timely detection of possible errors, documenting the clinical trial process and ensuring the protection of the rights of the subjects of the study and the compliance of the study with the principles of good clinical practice (ICH GCP), the requirements of international law and the legislation of the Russian Federation.
Routine study monitoring includes:

- confirmation that the process of obtaining informed consent, as well as screening and enrolling subjects in the study, has been properly conducted and documented;
- verification of eIRK data and primary medical documentation of the subjects of the study;
- confirmation of documentation and timely reporting of information about HP during the study;
- confirmation of compliance by the clinical center staff with the requirements for the implementation of diagnostic and therapeutic procedures of the study protocol;
- confirmation of documentation of the supply, storage, distribution and destruction of the investigational product and study materials;
- confirmation of the competencies of the personnel of the clinical center, external laboratory, necessary for the study;
- confirmation of compliance of diagnostic and laboratory equipment with the requirements for safe and adequate use during the study;

- confirmation of the investigator's interactions with the local ethical committee regarding the safety of the study and the introduction of amendments to the study protocol agreed with the sponsor.

Ensuring the quality control of the results of the study is carried out by the employees of the sponsor/authorized representative of the sponsor, who maintain the electronic database of the study, which identify inconsistencies, erroneously entered data, missing data during the cross-check of all eRCF. If questions arise or the need for clarification arises, a special form (request for clarification of data) is sent to the researcher by e-mail/fax, the request, which must be satisfied in writing within 7 days after its delivery. In accordance with the requirements of the legislation, the sponsor or authorized state bodies have the right to inspect (audit) the material and technical support of the study and study documentation. The investigator must provide access to the documentation and all necessary information to persons authorized to conduct an audit or inspection.

10.2 Documentation

To conduct clinical trials at the Federal Government Budgetary Institution “National Research Center for Epidemiology and Microbiology n.a. N.F. Gamaleya” of the Ministry of Health of the Russian Federation has implemented a quality management system that includes written SOPs to ensure that clinical trials are conducted and data is obtained, documented and reported in accordance with the protocol, GCP principles and applicable regulatory requirements.

The investigator must maintain primary records for each study subject containing personal data (including hospital admissions and medical history) and records made at visits, including demographic and medical information, laboratory data and all other tests and procedures . Any information entered into the eIRC must have a primary source in the primary documentation. The investigator should also keep the original Informed Consent signed by the subject and the subject's parent/adoptive parent.

The investigator must ensure that the monitor has access to all primary documentation relevant to the subject to confirm the accuracy of the data entered in the eRCF. The monitoring standards of the Sponsor's company provide for a total check of informed consents, inclusion / non-inclusion criteria, documentation on AE/SAE, safety data, tolerability of the drug in the primary documentation. Additional checks on the compliance of primary information and records in the CRF are carried out in accordance with the monitoring plan for this study. No primary documentation identifying the identity of the subjects will be disclosed.

Regular visits of the study monitor, on behalf of the sponsoring organization, before the start, during the study and at its completion, contribute to the successful conduct of the study and serve as a guarantee of collecting accurate data and timely detection of possible errors, ensuring the protection of patients' rights and compliance of the study with the requirements of the legislation of the Russian Federation and good clinical practice.

In this study, during regular monitoring visits, continuous verification of eRCI data, monitoring of compliance with study procedures, rules for storing study drugs, reagents and consumables, as well as proper and timely maintenance of the main documents of a clinical trial, including reporting provided for by the rules of good clinical practice, are provided for. , the legislation of the Russian Federation and protocol-specific SOPs, as well as the SOPs of the clinical center, provided that the sponsor accepts this documentation as normative. In addition, the sponsor, an authorized representative of the Sponsor and the health authorities have the right to carry out an unscheduled inspection (audit) of the study logistics and study documentation.

Representatives of the Sponsor may visit the study site at any time during or after the study to conduct an audit of the study in accordance with regulatory requirements and company policy. Such audits will require access to all documents relating to the study, including primary documents, for inspection and comparison with the QFM. However, subject data must be kept confidential. At regular scheduled site audit visits by the Sponsor or its designees, the investigator and staff must be present to enable consultation.

The audit can also be carried out by representatives of any authorized body. The Investigator must notify the Sponsor immediately if contacted by a competent authority regarding an upcoming inspection.

To conduct clinical trials, the Gamaleya National Research Center for Epidemiology and Microbiology of the Russian Ministry of Health has introduced a quality management system.

Quality management in clinical trials is carried out by ensuring the control of relevant processes:

- carrying out all stages and operations of a clinical trial;
- development of the main documents of the clinical trial (Protocol, Brochure, data registration forms (eCRF);
- Protecting the rights, safety and well-being of volunteers (development of the Informed Consent Form and information leaflet);
- safety monitoring during the study;
- accounting procedures for the investigational product;
- methods for minimizing subjectivity in the course of the study;
- inspection and self-inspection;
- storage of study documentation.

10.3 Amendments to the protocol

Investigators' signatures on the Protocol Signatures page signify a written confirmation of consent to conduct research in accordance with this protocol. During the course of a clinical trial, changes and additions may be made to the study materials. Such changes and additions are considered as amendments.
Protocol Amendment A written description of changes or a formal explanation of the text of a clinical trial protocol. Amendments may be material or non-substantial. Any protocol amendment must be duly approved in accordance with the sponsoring company's internal SOPs, approved by the regulatory authorities, the NEC, and signed by the investigator before becoming effective.
The order of the Ministry of Health of the Russian Federation No. 775 dated August 31, 2010 “On approval of the procedure for considering a report on the need to make changes to the protocol for a clinical trial of a medicinal product for medical use” defines a list of significant / non-essential amendments and the procedure for submitting materials about them for examination.
Amendments to the materials of a clinical trial are considered significant if they can affect the objectives, forms of organization, methodology, statistical methods for processing the results of a clinical trial and measures to ensure the safety of volunteers participating in it.
Amendments to the materials of a clinical trial are considered insignificant if they do not affect the objectives, forms of organization, methodology, statistical methods for processing the results of a clinical trial and measures to ensure the safety of volunteers participating in it.
If it is necessary to make changes to this protocol, the research sponsor submits a message to the Ministry of Health of the Russian Federation about the need to make changes to the clinical trial protocol. The decision to make changes or to refuse to make changes is made by the Ministry of Health of the Russian Federation after an examination of the provided updated materials. Amendments to the minutes must be kept together with the original version of the minutes. The title page of the protocol must indicate the number and date of the amendment.

10.4 Protocol deviations

Protocol deviation is an unintentional deviation from the approved Study Protocol.
A major protocol deviation is one that, in the judgment of the investigator or the investigator's designee, may result in the withdrawal of the subject from the study or exclusion of the subject's data from the clinical and/or statistical portion of the study. Deviations that have not been classified as major are considered minor deviations from the Study Protocol.
Serious deviations from the Protocol should be reported to the sponsor as early as possible by the clinical center staff and/or the monitor (if present at the center). The Sponsor may propose to reclassify a Protocol deviation (minor to major or vice versa) based on the evaluation. In such a case, the classification made by the sponsor shall prevail and should be communicated to the clinical center staff along with written justification.
The sponsor must be informed of minor deviations from the Protocol within 10 working days, but before the start of the next study period/phase or before the start of the clinical phase/statistical phase.
Notifications and reports of deviations from the Protocol are submitted to the competent authorities and the relevant ethical committees in accordance with applicable requirements/guidelines/laws.

Procedure for documenting deviations from the Protocol

The investigator, or the investigator's designated responsible person, must document and explain any deviation from the approved Study Protocol.

The investigator may deviate from the protocol or make changes to it to eliminate the immediate threat to the subjects of the study without prior approval (issuance of a conclusion) by the IRB / IEC. As soon as possible, a description of the deviations or changes, indicating their reasons, and, if necessary, proposed amendments to the protocol should be sent to:

- ESO (NEC) for consideration and approval (issuance of an opinion);
- Sponsor for agreement with him;
- authorized bodies, if necessary.

The investigator must immediately report to the IRB/IEC:

- protocol deviations or protocol changes made to address an immediate threat to study subjects;
- about changes that increase the risk for subjects of the study and (or) significantly affect the conduct of the study;
- any unexpected serious adverse drug reactions;
- new data that may indicate an increased risk to study subjects or adversely affect the course of the study.

Sponsor's notification of a protocol deviation may, in exceptional cases, be given verbally (if immediate action/notice is required) followed by a written notice (e.g., by email; in a communication about the progress of the study period). All deviations from the protocol should be described in the final report of the study.

10.5 List of data recorded in an individual registration card (without prior written or electronic recording) and considered as primary data

All data obtained as part of this clinical trial will be pre-registered in writing and / or electronically (depending on what is applicable in a particular medical organization) in primary medical records, with subsequent entry of data into the eRCF. No data will be recorded directly in eIRK without prior registration in the primary documentation.

11 Description of the ethical aspects of the clinical trial

11.1 General

This study will be carried out in accordance with the Declaration of Helsinki of the World Medical Association "Ethical principles for conducting medical research involving a person as a research subject" of 1964 with subsequent changes and additions; Rules of Good Clinical Practice of the EAEU (approved by the Decision of the Council of the Eurasian Economic Commission dated November 3, 2016 No. 79); Order of the Ministry of Health of the Russian Federation of April 1, 2016 No. 200n "On Approval of the Rules of Good Clinical Practice"; Federal Law of the Russian Federation of April 12, 2010 N 61-FZ "On the Circulation of Medicines" (as amended), as well as ICH guidelines, including the E6 Good Clinical Practice Guide (ICH E6 GCP).

11.2 Procedure for obtaining an informed consent

Prior to inclusion in the study, the subject is provided with written information and oral explanation of the aims, objectives and methods of the study, as well as the expected benefits and possible risks associated with participation in the study. In addition, subjects should be made aware of the voluntary nature of participation in the study and that the subject has the right to refuse participation in the study at any time, and that this refusal will not affect the quality of care provided to him. Although the subject is not required to report the reasons for withdrawing from the study, the investigator should attempt to ascertain those reasons without violating the subject's rights. Consent from the subject must be obtained prior to any study procedures.

The processing of data collected during the study is carried out in compliance with the confidentiality of the data of the subjects. Subjects should be made aware of the purposes of the planned computer processing of data and the conditions for publishing these data (for example, for presentation at medical conferences, in journal articles and other open sources), presented only in an aggregated form that does not allow its identification.

Subjects must be made aware that authorized health officials and the sponsor will have access to their protected health information for monitoring, inspection and audit purposes. At the same time, subjects must be guaranteed strict confidentiality of all information that allows the identification of the subject of the study, and non-disclosure of such information.

The Informed Consent Form (Volunteer Information Sheet) must be completed in duplicate, signed and dated by the subject and the investigator in their own handwriting. The parent/adoptive parent informed consent form must be completed in duplicate, signed and dated by the parent/adoptive parent and the investigator in their own handwriting. The first copy of the signed informed consent forms must be kept by the investigator in the Investigator's File, the second copy must be issued to the research subject, parent/adoptive parent.

11.3 Confidentiality and identification of research subjects

The confidentiality of records that allow the identification of research subjects will be protected in accordance with the right to privacy and the protection of confidentiality in accordance with regulatory requirements. Volunteer identification records will be kept confidential and may only be disclosed to the extent permitted by law. When the results of the study are published, the confidentiality of the personal data of the subjects will be preserved. Confidentiality of the subjects is guaranteed by the Federal Law of July 27, 2006 No. 152-FZ "On Personal Data" (as amended).

11.4 Recruitment of subjects from vulnerable and special groups

The study will involve healthy volunteers - adolescents 12–17 years old, their rights will be protected, which is reflected in the information leaflet of the volunteer and is confirmed by the signing of the informed consent form of both the teenager himself and his parent / adoptive parent. Adolescents and their parents/adoptive parents will receive all necessary clarifications from the volunteer's information sheet and from the investigator's physician. Participation in the study requires an agreed voluntary decision by the participant and their parent/adoptive parent, after understanding all aspects of the study.

The participation of children with disabilities in the study is not provided. The participation of children left without guardianship is not provided.

Participation in the study implies the receipt of monetary compensation.

Remuneration is paid for participation in the first stage of the study. Its purpose is to compensate for inconvenience and lost time. According to the schedule, volunteers will undergo screening, be hospitalized twice for 3 days, and will be observed on an outpatient basis (5 visits that involve blood donation), will fill out diaries and interact with telemedicine center doctors to assess safety and effectiveness throughout the year. During this time, they must comply with protocol requirements, will be restricted from vaccinating with other vaccines, and will not be able to participate in other studies. Volunteers will receive 100,000 rubles before taxes, which is comparable to payments made by adults for participating in a similar study - 100,000 rubles after taxes, in which volunteers were hospitalized for 28 days and observed for six months, during which they donated blood twice.

The second phase of the study is outpatient, volunteers will visit the clinic and donate blood 5 times, for which they will receive a compensation of 15,000 rubles before taxes, which is comparable to similar payments for adult volunteers in the third phase of the Gam-Covid-Vac vaccine trials of 10,000 rubles before taxes for 3 blood donations.

Payments to the placebo group are intended to compensate for the inconvenience of extended follow-up, repeat injections, and the need to donate blood again. Volunteers from the placebo group will receive 15,000 rubles.

Structure of payments

Group	Number of volunteers	Amount, rub
stage 1		
Vaccine, hospitalization, donate blood	100	100,000
stage 2		

Vaccination, blood sampling	400	15,000
Placebo	600	15,000

Payment terms will be detailed in the contract.
The informed consent states that the parents/adoptive parents undertake to spend this amount on the needs of the child.
This amount of remuneration was assessed as fair, sufficiently motivating to comply with the protocol, and at the same time not too large for coercion to participate and inadequate risk assessment, both on the part of adolescents and their parents/adoptive parents. It should be noted that, in accordance with the current legislation, payments under a clinical trial are the basis for stopping the payment of subsidies, benefits or other social payments, if the volunteer receives them. The volunteer and his parent/adoptive parent are informed about this in advance - this information is included in the informed consent. This circumstance is also an obstacle to possible coercion to participate in the study for financial reasons.

12 Description of data handling and record keeping

All site records and documents related to the clinical investigation, including those on the investigator's file (including eRCF, informed consent forms, journals, subject records, etc.), as well as the subject's primary medical records, must be retained for 15 years after the end of the study. The sponsor of the study must control the security and availability of all clinical investigation materials throughout the entire life cycle of the investigational product. Archival data can be stored in the form of photocopies, as well as on optical and electronic media. The Principal Investigator is obliged to immediately inform the sponsor of the facts of unintentional damage/destruction, as well as changes in the storage location of the archived clinical trial materials. Purposeful destruction of archived research materials is possible only with the written permission of the Study Sponsor.
All information received, including information on AE/SAE, will be entered into the primary documentation and then transferred to the eIRC. eIRK will not contain data that is not reflected in the primary documents.
As subjects progress through their scheduled visits and the eRCF is completed by the investigator, the eRCF and primary documentation will be reconciled by the sponsor's authorized monitors. If the eRCF is filled in correctly and accurately in accordance with the data of the primary documentation, the monitor confirms in the eRCI the reconciliation of the primary documentation and eRCI data by setting the reconciliation flag. In the event that at the stage of data evaluation in the eRCF, the data control manager and/or biostatistician have questions about the data, all clarifications and changes in the eRCF data will be documented by creating electronic requests for data clarification in the eRCF. Responses to such requests given by the researcher in the eIRC are checked by the monitor, including for compliance of the corrected data with the text of the request (if applicable), and, if the answer is recognized as sufficient, the request will be closed. Otherwise, the request will be reopened with additional explanatory text for the researcher.

The investigator is required to provide information demonstrating that subjects can be recruited in a timely manner while meeting protocol criteria.

The study must be conducted in accordance with the sponsor's protocol and applicable standard operating procedures. If it becomes necessary to make changes to the protocol, the procedure outlined in section 10.3 of this protocol must be followed.

It is mandatory for investigators to complete the primary medical records and eRCI of all subjects included in the study.

The investigator is responsible for the complete and accurate completion of the eRCF. All data recorded in the eRCI must be reflected in the subject's primary medical records in hard copy or in the form of records made by the investigator or other authorized person of the clinical center.

In the eRCF, in accordance with the primary documentation, all significant information about the participation of the subject in the study is recorded. The eCRF should contain information about the completion of the subject's participation in the study. The eRCF must be completed no later than 7 days after the subject's visit to the study site.

eIRK must be filled in in accordance with the instructions for filling out eIRK. Errors made should be corrected by entering a new value in the eIRK, while the old value will be stored in the history of changes (audit trail). For all missing data, explanations must be provided in the eRCF, which will be implemented by the presence of a special marker field (“tick”) confirming the missing data. If necessary, the researcher can add a comment to the corresponding field in the eRCF with an explanation of the reasons for the omission of data. eRCI must be certified by the electronic signature of the researcher. These signatures certify that the information contained in the eRCF is correct.

For volunteers who prematurely terminate participation in the study, the “Study Termination” section of the eRCF is completed, where it is mandatory to indicate in detail the reason why the volunteer withdrew from the study.

All study information and data collected are strictly confidential. The investigator has the right to disclose information about the study to persons not directly involved in the conduct of the study, only with the permission of the sponsor.

The final report, consisting of a statistical and clinical report, is generated after the database is closed and the statistical processing of the study results is completed. The final report is signed by the principal investigator of the clinical center, who confirms the results and conclusions of the study, sealing the report with the seal of his institution.

12.1 Direct access to primary data/documentation

Primary data is all the information contained in the original medical records and their certified copies, about the results of clinical observations, examinations and other activities that allows you to recreate the course of a clinical trial and evaluate it. Primary data are contained in the primary documentation (originals or certified copies). The researcher provides the possibility of monitoring the study, audit(s), examination by the Ethics Council and regulatory authorities, provides direct access to primary data/records.

Primary Documents - source documents, data, and records (e.g., case histories, patient records, laboratory records, notes, study diaries, questionnaires, dispensing logs, automated device records, verified and certified copies or extracts, microfiches, photonegatives, microfilms, or magnetic media, x-rays, any records related to the volunteer, including those stored in a pharmacy, laboratories and instrumental diagnostic departments used in a clinical trial). Primary data should be stored in an appropriate quality for the entire period of time provided for by local and international legislation, as well as written agreements with the study sponsoring company. For each enrolled subject, the investigator shall indicate in the primary records that the subject is participating in the study, and also record at least the following information: individual identification code, personal data of volunteers (name, address), date of drug administration, vital signs, any AE, study completion dates, and main reasons for treatment discontinuation (if applicable).

The duty of the investigator is to provide direct access to primary data and documentation for the clinical research specialist of the sponsoring company, the auditor of the competent authorities, representatives of the insurance company, ethics committees.

13 Financing and insurance

13.1 Funding

Financing of the clinical trial is carried out by the organization-developer of the medicinal product of the Federal State Budgetary Institution "National Research Center for Epidemiology and Microbiology named after N.N. N.F. Gamaleya" of the Ministry of Health of Russia.

The financial aspects of the study will be documented in the form of an agreement between the drug developer (Sponsor) and the research organization (Sponsor's representative).

Neither the Sponsor nor the investigator is financially responsible for the study/treatment of medical conditions that may be identified during screening.

The sponsor of the study will provide the required amount of study drug for the study, all expenses for the development of the drug are borne by the Sponsor. Responsibility for the safety of the drug from the moment of transfer of the study drug by the Sponsor lies with the clinical center where the study will be conducted.

Volunteers participating in the immunological part of the study will receive monetary compensation for the time spent on donating blood and additional visits to the clinical center, the payment procedure will be described in the agreement with the volunteer.

13.2 Insurance

The Principal Investigator of the Research Center is responsible for the safety of volunteers during this study. In the event that a volunteer develops an AE, the Principal Investigator and his staff will provide him with medical care and will do everything possible to treat him.

If the volunteer agrees to participate in the study, his participation in this study will be insured by the company in accordance with the Federal Law of April 12, 2010 N 61-FZ "On the Circulation of Medicines", Decree of the Government of the Russian Federation No. 714 "On Approval of Model Rules for Compulsory Life Insurance and Health of a Patient Participating in Clinical Trials of a Medicinal Product" dated September 13, 2010 and Decree of the Government of the Russian Federation No. 393 "On Amendments to the Standard Rules for Compulsory Life and Health Insurance for a Patient Participating in Clinical Trials of a Medicinal Product" dated May 18, 2011. In accordance with the Decrees of the Government of the Russian Federation No. 714 dated September 13, 2010, No. 393 dated May 18, 2011 "On Approval of Model Rules for Compulsory Life and Health Insurance of a Patient Participating in Clinical Trials of a Medicinal Product".

Risks to the life and health of volunteers participating in the clinical trial will be insured in accordance with the established procedure by the insurance company Capital Life Life Insurance LLC.

The researcher will inform the subject about the availability of such insurance, each volunteer after signing the informed consent will receive an insurance policy. Subjects participating in this study are guaranteed insurance against the risk of harm to life and health in accordance with the requirements of the legislation of the Russian Federation. In order to maintain anonymity, the personal data of each volunteer in the policy will be replaced by an Individual Volunteer Identification Code, which is assigned to him during the study in the form established on the territory of the Russian Federation. In the event of harm to the health of a volunteer associated with a clinical trial, the insurance company undertakes to reimburse all costs for the necessary medical examination and treatment, the need for which will arise as a result of direct exposure to the investigational drug and / or medical manipulations applied according to the Study Protocol.

The study does not provide for any additional types of voluntary insurance or other options for providing treatment and / or compensation in case of harm to the health of a volunteer as part of the study.

Sponsor shall not be liable for any loss, harm and/or damage that may be caused to a volunteer if such loss, harm and/or damage is caused by:

- taking a prohibited drug during the study;
- deviation on the part of the volunteer from the study protocol, study requirements, and/or any instructions or directions that the investigator may give;
- the act or omission of a third party to adequately respond to an AE or response to an investigational drug.

14 Independent data monitoring committee

Data Monitoring Committee (IDMC; Data and Safety Monitoring Board, DSMB) is an independent committee (a group of experts not directly involved in the study) will be formed by the Study Sponsor to periodically review the progress of the clinical study, data on safety, as well as to make recommendations to the sponsor on the advisability of continuing, terminating the study or making changes to it.

Prior to being included in the committee, members of the IDMC must report in writing that there is no conflict of interest.

The main task of the IDMC is the ongoing independent assessment of the safety of the investigational drug and, accordingly, the risk to study participants.

The IDMC can consider such significant indicators of the current study as the number and nature of deviations from the protocol, premature withdrawal of patients from the study, etc.

The IDMC bases its recommendations (conclusions) on the results of an interim analysis of the obtained data.

The functions of the IDMC cannot be entrusted to the sponsor's staff, researchers or independent ethics committees. The independence of the IDMC should ensure that the recommendations of the IDMC are objective and prevent sponsor staff, CROs, and investigators involved in the conduct of the trial from accessing blinded data and intermediate results of the trial, thus ensuring that the validity of the trial is maintained.

The IDMC shall act in accordance with written procedures and document its meetings and decisions. In its work, the IDMC may hold closed and open meetings. Closed meetings are held only with members of the IDMC who have access to uncovered information. Public meetings are held with representatives of other groups involved in the conduct of the study

The expert opinion of the IDMC constitutes a recommendation; the final decision regarding the course of the study, taking into account the recommendations of the IDMC, is made by the sponsor of the study.

Information about the role of the IDMC and the decisions it makes should be included in the clinical trial report.

Table 5. IDMC composition.

No.	Full Name	Role	Qualification, place of work, position
1	Naigovzina Nelli Borisovna	IDMC chairman	Head of the Department of Public Health and Healthcare, Moscow State University of Medicine and Dentistry n.a. A.I. Evdokimov of the Ministry of Health of Russia, President of Evogen LLC, Doctor of Medical Sciences, Prof., Honored Doctor of the Russian Federation
2	Sychev Dmitry Alekseevich	Vice-chairman	Rector at Federal State-Funded Educational Institution “Russian Medical Academy of Continuous Professional Education” of the Ministry of Health of Russia, MD, Professor, Corresponding Member of the Russian Academy of Sciences
3	Bukhtin Kirill Mikhailovich	IDMC member, secretary,	Researcher at Federal Government Budgetary Institution “National Research Center for Epidemiology and Microbiology n.a. N.F. Gamaleya” of the Ministry of Health of the Russian Federation, Ph.D. (without the right to vote)

4	Namazova-Baranova Leyla Seymurovna	IDMC member	Head of the Department of Faculty Pediatrics, Faculty of Pediatrics, Federal State-Funded Educational Institution Russian National Research Medical University Education n.a. N.I. Pirogov of the Ministry of Health of Russia, Head of the Research Institute of Pediatrics and Children’s Health at the Central Clinical Hospital of the Russian Academy of Sciences of the Ministry of Science and Higher Education of the Russian Federation, chief freelance pediatric specialist in preventive medicine of the Ministry of Health of Russia, Doctor of Medical Sciences, Prof., Academician of the Russian Academy of Sciences

5	Kulichenko Tatyana Vladimirovna	IDMC member	Head of the Department for the Implementation of the Functions of the National Medical Research Center for Pediatrics, Federal State Autonomous Educational Institution of Higher Education n.a. N.I. Pirogov of the Ministry of Health of Russia, Chief freelance specialist pediatrician of the Central Federal District, MD, professor of the Russian Academy of Sciences
6	Ermolaeva Irina Igorevna	IDMC member	Deputy Director of the Department of Clinical Research, FSAEI First Moscow State Medical University of the Russian Academy of Medical Sciences n.a. I.M. Sechenov of the Ministry of Health of Russia, cybernetics doctor
7	Kharit Susanna Mikhailovna	IDMC member	Leading Researcher, Head of the Department for the Prevention of Infectious Diseases at FMBA Federal State Budgetary Institution "Children's Scientific and Clinical Center for Infectious Diseases"
8	Kostinov Mikhail Petrovich	IDMC member	Head of the Laboratory of Vaccine Prevention and Immunotherapy of Allergic Diseases, Research Institute of Vaccines and Serums n.a. I. I. Mechnikov of the Russian Academy of Medical Sciences, Honored Worker of Science of the Russian Federation, Doctor of Medical Sciences, Professor
9	Alekseeva Ekaterina Iosifovna	IDMC member	Director of the Clinical Institute of Children's Health n.a. N.F. Filatov, Head of the Department of Pediatrics and Pediatric Rheumatology at FSAEI First Moscow State Medical University of the Russian Academy of Medical Sciences n.a. I.M. Sechenov of the Ministry of Health of Russia, Doctor of Medical Sciences, Professor, Corresponding Member of the Russian Academy of Sciences
10	Pampura Alexander Nikolaevich	IDMC member	Head of the Department of Allergology and Clinical Immunology, National Research Institute of Pediatrics, State Budgetary Educational Institution of Higher Professional Education n.a. N.I. Pirogov of the Ministry of Health of Russia Federation, chief freelance pediatric specialist allergist-immunologist at Moscow Health Department, MD
11	Zinserling Vsevolod Aleksandrovich	IDMC member	Head of Research Institute of Pathology, Federal State Budgetary Institution National Medical Research Center n.a. V.A. Almazov of the Ministry of Health of Russian Federation, Doctor of Medical Sciences, Professor

15 Publications

The information contained in this document is the property of the Sponsor and disclosure to third parties is permitted only with the written permission of the Sponsor. The right to access this information is limited to investigators and research facility staff participating in the study, members of the NEC, and public health officials authorized to oversee the conduct of the clinical trial. Information about the study, to the extent necessary to make a decision on granting consent to participate, is provided to subjects who may participate in this study.

The results of this study are exclusively owned by the sponsor of the study. No data from this study may be presented or published without the prior written permission of the sponsor.

16 References

1. Yang F. et al. Clinical characteristics and outcomes of cancer patients with COVID-19 // J. Med. Virol. 2020. Vol. 92, № 10. P. 2067–2073.
2. COVID-19 vaccination: the VOICE for patients with cancer | Nature Medicine [Electronic resource]. URL: https://www.nature.com/articles/s41591-021-01240-w: (accessed: 19.03.2021).
3. Rojas M. et al. Convalescent plasma in Covid-19: Possible mechanisms of action // Autoimmun. Rev. 2020. Vol. 19, № 7. P. 102554.
4. Wang X. et al. Neutralizing Antibodies Responses to SARS-CoV-2 in COVID-19 Inpatients and Convalescent Patients: preprint. Infectious Diseases (except HIV/AIDS), 2020.
5. Chandrashekar A. et al. SARS-CoV-2 infection protects against rechallenge in rhesus macaques // Science. 2020. Vol. 369, № 6505. P. 812–817.
6. Yu J. et al. DNA vaccine protection against SARS-CoV-2 in rhesus macaques // Science. 2020. Vol. 369, № 6505. P. 806–811.
7. Naming the coronavirus disease (COVID-19) and the virus that causes it [Electronic resource]. URL: https://www.who.int/emergencies/diseases/novel-coronavirus-2019/technical-guidance/naming-the-coronavirus-disease-(covid-2019)-and-the-virus-that-causes-it (accessed: 16.03.2021).

Preston LE, Chevinsky JR, Kompaniyets L, et al. Characteristics and Disease Severity of US Children and Adolescents Diagnosed With COVID-19. *JAMA Netw Open.* 2021;4(4):e215298. doi:10.1001/jamanetworkopen.2021.5298

Appendix 1. Visual analog scale (VAS) for pain assessment

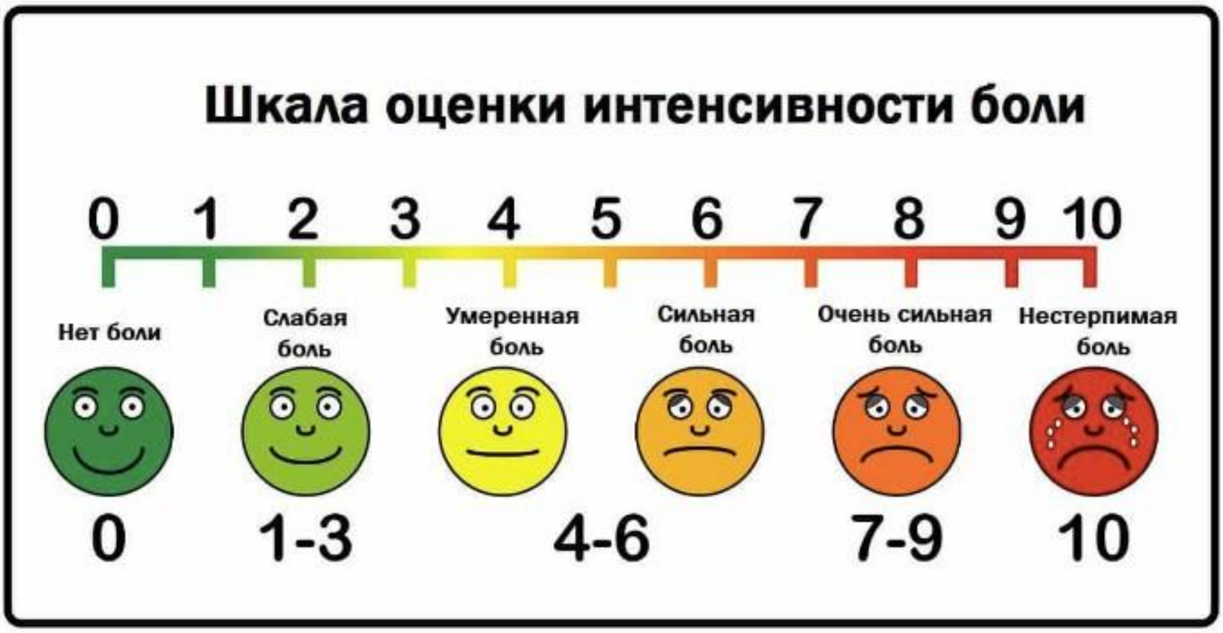

Comment: The VAS pain score is used to assess the subjective sensations of the volunteer. The doctor shows the volunteer a scale when questioning complaints and fixes the VAS scores in the primary documentation and CRF. Assessment of the severity of pain as an adverse event is made in accordance with Appendix 3 “Vaccination Adverse Events Rating Scale”.

Appendix 2 DIARY OF A VOLUNTEER. Template

Volunteer note:

Please complete this questionnaire if you have any complaints of feeling unwell after vaccination.

This is necessary so that you save this important information, and the doctors can correctly assess the situation. To do this, they may need to contact you for clarification.

Please list any complaints you have had since the vaccine was given. If you have additional complaints that were not previously listed, describe them separately in the last “Comments” section.

**I. PASSPORT PART ***
1. individual volunteer code
2. Gender (male, female):
3. Date of birth (month, year):
4. Age: ☐☐ (years)

** this information is not filled in by the volunteer in the mobile application*

**II. VACCINE INFORMATION ***
5. At which institution was the vaccine administered?:
6. Date of first vaccine administration:
7. Date of second administration (if the vaccine was administered twice):

** this information is not filled in by the volunteer in the mobile application*

**III. INFORMATION ABOUT COMPLAINTS AFTER VACCINATION**
8. Have you had any complaints about how you feel after getting the vaccine?<br>☐ Yes☐ No
9. Date of occurrence of complaints after vaccination:
10. Note any symptoms that occur:<br><br>10.1. GENERAL COMPLAINTS:<br>• absent<br>• temperature increase<br>• chills<br>• headache<br>• dizziness<br>• weakness<br>• malaise<br>• feeling of heat<br>• ache (pain) in the muscles<br>• ache (pain) in the joints<br>• pain in the chest area

- a sore throat
- difficulty breathing
- cough
- runny nose
- nasal congestion
- sweating
- decreased sense of smell
- loss of taste
- loss of appetite
- nausea
- vomit
- stool disorders
- abdominal pain

10.2. LOCAL SIGNS

- absent
- pain at the injection site
- rash
- itching
- swelling at the injection site
- swelling at the injection site
- redness of the skin at the injection site
- allergic reaction

in case of selecting “allergic reaction” field for free entry “how it manifests itself”

10.3.CARDIOVASCULAR DISORDERS

- absent
- pressure increase (with the possibility of entering a digital pressure value)
- pressure reduction (with the possibility of entering a digital pressure value)
- increased heart rate (with the ability to enter a digital value of the pulse)
- slow heart rate (with the ability to enter a digital heart rate value)
- pain in the heart
- fainting or loss of consciousness

10.4.DISORDERS ON THE PART OF THE ORGAN OF VISION

- absent
- flies flickering before the eyes
- pain in the eyeballs
- color perception disorders
- discomfort in bright light
- blurred vision

10.5. Do you have any other complaints: Yes No
If you select yes: free input field for complaints

10.6. When these complaints were resolved:

10.7. Have you had any other illnesses or ailments since the vaccination?

diseases	
date of onset	
end date	

with the possibility of multiple filling

11. What did you do when signs of illness (malaise) appeared?
- nothing
- call an ambulance
- hospital admission
- -calling the doctor of the polyclinic at home
- visiting a clinic doctor
- taking medications (name of the drug, dosage, duration of administration)
- additional treatment (with free entry field)

<u>Free entry field</u>

IV. IMPORTANT ADDITIONAL INFORMATION

12. Have you ever had a pregnancy after vaccination: Yes ☐ No ☐

13. Did you have contact with people who were later diagnosed with COVID-19 after the first vaccine injection: Yes☐ No ☐

14. Have you had contact with people who were later diagnosed with COVID-19 after the second vaccine injection: Yes ☐ No ☐

4. Have you ever traveled to other cities and countries after vaccination: Yes ☐ No ☐
If "Yes" is selected, fill in the additional fields
Departure date
Country
City

16. Have you tested for COVID-19 on your own (outside the study protocol) after vaccination: Yes *☐ No ☐

17. Describe what tests for COVID-19 and when you passed after vaccination, and what were the results of these tests:
17.1. PCR test
analysis date
analysis result (with the ability to indicate that the result of the analysis is not known)
17.2. SARS-CoV-2 antibody blood test
analysis date
analysis result (with the ability to indicate that the result of the analysis is not known)

18. Comments:

Thank you for your time.
Your information is very important for your and public health

Appendix 3 Vaccination Adverse Events Rating Scale[9]

Local reaction to the injectable product	Mild degree (class 1)	Moderate degree (class 2)	Severe degree (class 3)	Potential life hazard (class 4)
Pain	Does not affect activity	Repeated use of a non-narcotic pain reliever >24 hours or interferes with activity	Any use of narcotic pain medication or that prevents daily activities	Ambulance or hospitalization
Sensations	Mild discomfort to the touch	Discomfort when moving	Significant discomfort at rest	Ambulance or hospitalization
Erythema<br>Redness	2.5 – 5 cm	5.1 – 10 cm	> 10 cm	Necrosis or exfoliative dermatitis
Edema	2.5 – 5 cm and does not affect activity	5.1 – 10 cm or affects activity	> 10 cm or affects activity	Necrosis
Local reaction to the injectable product*	Severity is determined by the most pronounced reaction			

* – combines at least two local reactions (pain, subjective sensations, redness, swelling)

**Vital parameters**	Mild degree (class 1) [10]	Moderate degree (class 2)	Severe degree (class 3)	Potential life hazard (class 4)
Fever (°C)	38.0 – 38.4	38.5 – 38.9	39.0 – 40	> 40
Tachycardia, heart rate	101 – 115	116 – 130	> 130	Ambulance or hospitalization
Bradycardia, heart rate*	50 – 54	45 – 49	< 45	Emergency room visit or hospitalization for arrhythmia
Systolic hypertension, mmHg	141 – 150	151 – 155	> 155	Emergency room visit or hospitalization for malignant hypertension

[9] Guidance for Industry. Toxicity Grading Scale for Healthy Adult and Adolescent Volunteers Enrolled in Preventive Vaccine Clinical Trials. U.S. Department of Health and Human Services Food and Drug Administration Center for Biologics Evaluation and Research September 2007

[10] The undesirable phenomenon "fever" is fixed when the body temperature rises to 38°C and above. The temperature in the range of 37.0-37.9 ° C is recorded as hyperthermia, the severity is assigned "mild"

Diastolic hypertension, mmHg	91 – 95	96 – 100	> 100	Emergency room visit or hospitalization for malignant hypertension
Diastolic hypertension, mmHg	85 – 89	80 – 84	< 80	Emergency room visit or hospitalization for malignant hypertension
Heart rate	17 – 20	21 – 25	> 25	Intubation

* bradycardia is normally found in athletes and people engaged in heavy physical labor

Note: Vital sign abnormalities should only be reported as adverse events if they are clinically significant and change from baseline.

[a] Vital signs should be measured at rest.

[b] No previous consumption of hot or cold drinks or smoking.

[c] Assessment of bradycardia in certain populations of healthy volunteers, such as trained athletes, requires clinical judgment

mmHg. = millimeters of mercury.

**Systemic reaction (general)**	**Severity of the systemic reaction**			
	**Mild degree (class 1)**	**Moderate degree (class 2)**	**Severe degree (class 3)**	**Potential life hazard (class 4)**
Nausea/vomiting	No impact on daily activities, or 1-2 episodes in 24 hours	Some effect on daily activities, or >2 episodes in 24 hours	Event interfering with activities of daily living requiring ambulatory intravenous hydration	Emergency room visit or hospitalization for hypotensive shock
Chills	No impact on daily activities	Some impact on daily activities	Significant impact on daily activities	Seeking emergency care or hospitalization

**Systemic reaction (general)**	**Severity of the systemic reaction**			
	**Mild degree (class 1)**	**Moderate degree (class 2)**	**Severe degree (class 3)**	**Potential life hazard (class 4)**
Headache	No impact on daily activities	Multiple use of non-narcotic pain medications in 24 hours, or some effect on daily activities	Significant; any use of narcotic analgesics, or an occurrence that interferes with daily activities	Seeking emergency care or hospitalization
Fatigue	No impact on daily activities	Some impact on daily activities	Significant impact on daily activities	Seeking emergency care or hospitalization
Myalgia	No impact on daily activities	Some impact on daily activities	Significant impact on daily activities	Seeking emergency care or hospitalization

**General system indicators**	Mild degree (class 1)	Moderate degree (class 2)	Severe degree (class 3)	Potential life hazard (class 4)
Flu-like syndrome*	Mild symptoms No effect on activity	Some impact on activity*	Significant; prevents daily activity**	-

* (CTCAE) v5.0 / Definition: A disorder characterized by a group of symptoms similar to those seen in patients with influenza, including fever, chills, body aches, malaise, loss of appetite, and dry cough, combining at least two local reactions (headache, weakness, arthralgia, myalgia, hyperthermia or fever). Severity is determined by the most pronounced manifestation

Daily activity
* definition refers to cooking, shopping for food or clothes, using the phone, etc.
** definition means bathing, dressing and undressing, self-feeding, using the toilet, taking medication, not being bedridden.

**Systemic diseases**	Mild degree (class 1)	Moderate degree (class 2)	Severe degree (class 3)	Potential life hazard (class 4)
Illness or clinical adverse event (as defined in accordance with applicable regulations).	No effect on activity	Some effect on activity, does not require medical intervention	Significant; prevents daily activities and requires medical attention.	Ambulance or hospitalization

Classification of severity of lymph node involvement after vaccination (CTCAE v5.0)

	Mild degree (class 1)	Moderate degree (class 2)	Severe degree (class 3)	Potential life hazard (class 4)
lymphadenopathy after vaccination	local enlargement of the lymph nodes	local ulceration; generalized enlargement of the lymph nodes	-	-

– November 27, 2017, p. 43

Laboratory indicators

Blood analysis

Local reaction to the injectable product	Mild degree (class 1)	Moderate degree (class 2)	Severe degree (class 3)	Potential life hazard (class 4)
Hemoglobin g/dl Female	11.0 – 12.0	9.5 – 10.9	8.0 – 9.4	< 8.0
Hemoglobin g/dl Male	12.5 – 13.5	10.5 – 12.4	8.5 – 10.4	< 8.5
Increase in leukocyte cells/mm^3	10,800 – 15,000	15,001 – 20,000	20,001 – 25,000	> 25,000
Decrease in leukocyte cells/mm^3	2,500 – 3,500	1,500 – 2,499	1,000 – 1,499	< 1,000
Decrease in lymphocyte cells/mm^3	750 – 1,000	500 – 749	250 – 499	< 250
Decrease in neutrophils cells/mm^3	1,500 – 2 000	1 000 – 1,499	500 – 999	< 500
Eosinophils cells/mm^3	650 – 1,500	1,501 – 5,000	> 5,000	Hypereosinophilia

Decrease in platelets cells/mm^3	125,000 – 140,000	100,000 – 124,000	25,000 – 99,000	< 25,000
Prothrombin time	1.0 – 1.10 ×ULN**[11]	1.11 – 1.20 × ULN	1.21 – 1.25 × ULN	> 1.25 ULN

[11] "ULN" is the upper limit of the normal range

APPT	1.0 – 1.2 × ULN	1.21 – 1.4 × ULN	1.41 – 1.5 x ULN	> 1.5 × ULN
Hyperglycemia	65 – 69	55 – 64	45 – 54	< 45
Hyperglycemia				
Hyperglycemia<br>Fasting - mg/dl<br>Random - mg/dl	100 – 110<br>110 – 125	111 – 125<br>126 – 200	> 125<br>> 200	Insulin requirement or hyperosmolar coma
Creatinine concentration	1.5 – 1.7	1.8 – 2.0	2.1 – 2.5	> 2.5 or dialysis
Albumin	2.8 – 3.1	2.5 – 2.7	< 2.5	--
Bilirubin	1.1 – 1.25 × ULN	1.26 – 1.5 × ULN	1.51 – 1.75 × ULN	> 1.75 × ULN
The concentration of total cholesterol	201 – 210	211 – 225	> 226	---
Alkaline phosphatas e	1.1 – 2.0 × ULN	2.1 – 3.0 × ULN	3.1 – 10 × ULN	> 10 × ULN
ALT<br>AST	1.1 – 2.5 × ULN	2.6 – 5.0 × ULN	5.1 – 10 × ULN	> 10 × ULN

Urinalysis

Local reaction to the injectable product	Mild degree (class 1)	Moderate degree (class 2)	Severe degree (class 3)	Potential life hazard (class 4)
Protein	traces	1+	2+	Hospitalization or dialysis
Glucose	traces	1+	2+	Hospitalization for hyperglycemia
Red blood cells	1-10	11 – 50	> 50 or blood in macroscopic quantities	Hospitalization or transfusion of red blood cells (PRBC)

Annex 4 Body mass index

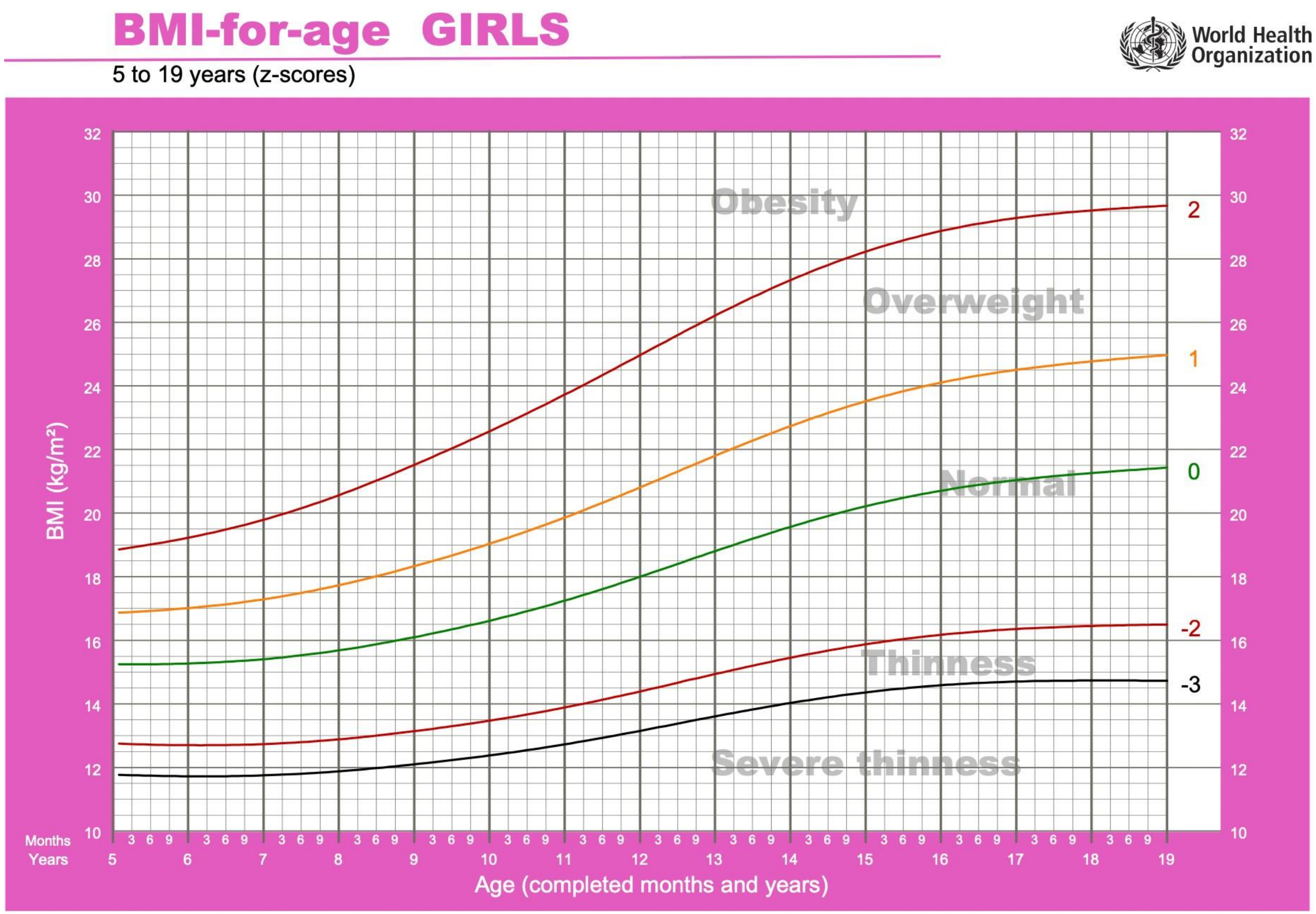

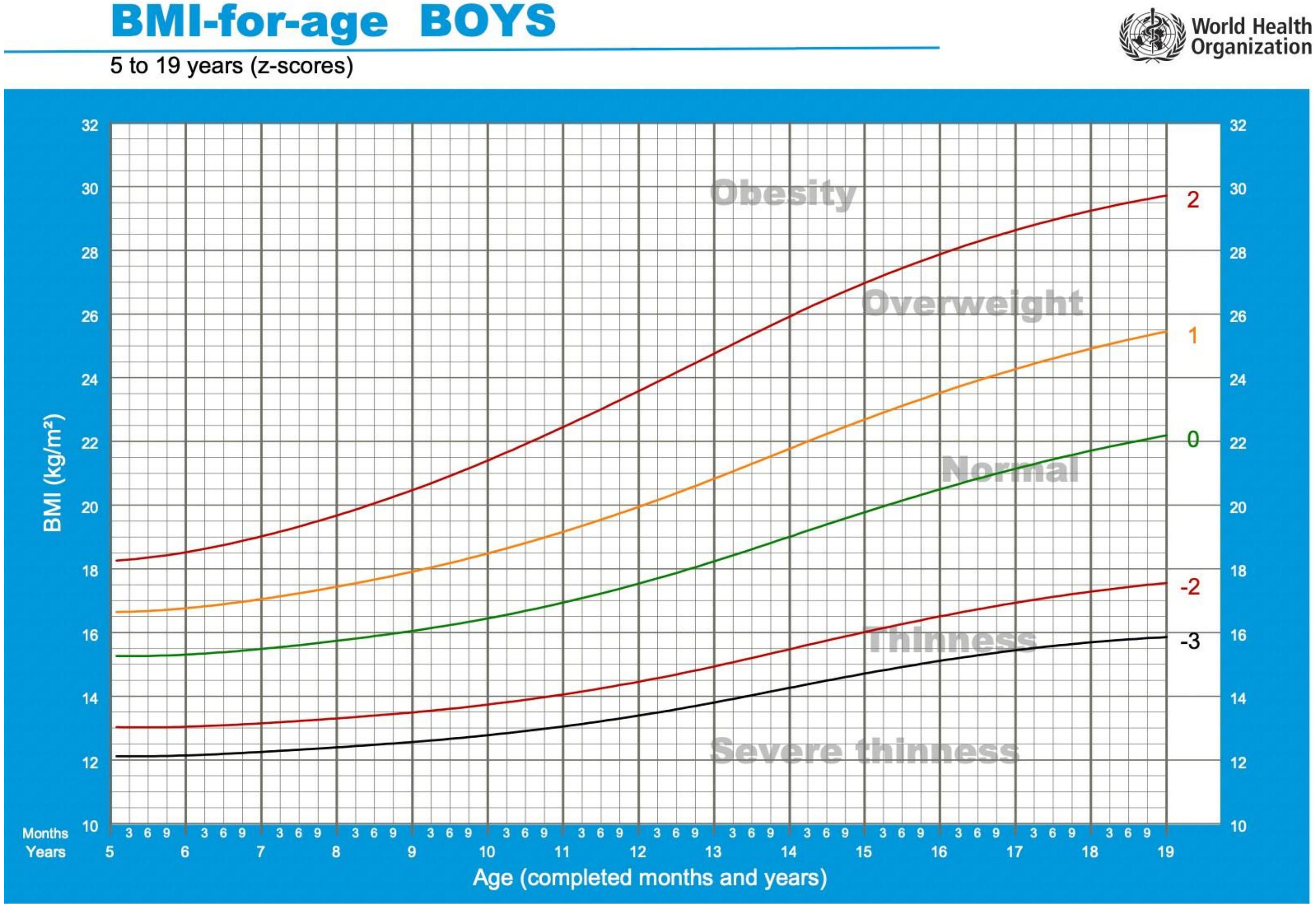

https://www.who.int/toolkits/growth-reference-data-for-5to19-years/indicators/bmi-for-age
